# Lung Cancers: Molecular Characterization, Clonal Heterogeneity and Evolution, and Cancer Stem Cells

**DOI:** 10.3390/cancers10080248

**Published:** 2018-07-27

**Authors:** Ugo Testa, Germana Castelli, Elvira Pelosi

**Affiliations:** Department of Hematology, Oncology and Molecular Medicine, Istituto Superiore di Sanità, 00161 Rome, Italy; germana.castelli@iss.it (G.C.); elvira.pelosi@iss.it (E.P.)

**Keywords:** non-small cell lung cancer, small cell lung cancer, cancer stem cells, genomic profiling, membrane cell markers, tumor xenotransplantation assay

## Abstract

Lung cancer causes the largest number of cancer-related deaths in the world. Most (85%) of lung cancers are classified as non-small-cell lung cancer (NSCLC) and small-cell lung cancer (15%) (SCLC). The 5-year survival rate for NSCLC patients remains very low (about 16% at 5 years). The two predominant NSCLC histological phenotypes are adenocarcinoma (ADC) and squamous cell carcinoma (LSQCC). ADCs display several recurrent genetic alterations, including: KRAS, BRAF and EGFR mutations; recurrent mutations and amplifications of several oncogenes, including ERBB2, MET, FGFR1 and FGFR2; fusion oncogenes involving ALK, ROS1, Neuregulin1 (NRG1) and RET. In LSQCC recurrent mutations of TP53, FGFR1, FGFR2, FGFR3, DDR2 and genes of the PI3K pathway have been detected, quantitative gene abnormalities of PTEN and CDKN2A. Developments in the characterization of lung cancer molecular abnormalities provided a strong rationale for new therapeutic options and for understanding the mechanisms of drug resistance. However, the complexity of lung cancer genomes is particularly high, as shown by deep-sequencing studies supporting the heterogeneity of lung tumors at cellular level, with sub-clones exhibiting different combinations of mutations. Molecular studies performed on lung tumors during treatment have shown the phenomenon of clonal evolution, thus supporting the occurrence of a temporal tumor heterogeneity.

## 1. Introduction

Lung cancer is one of the leading causes of cancer-related deaths in the world. The five-year lung cancer survival rate is only 15% and lung cancer alone is responsible for more deaths each year than breast, pancreas, colon and prostate cancers together. Lung cancer is the second most common cancer in both men and women: in men, prostate cancer is the most common, while in women, breast cancer is more common. About 14% of all new cancers in USA are lung cancers [1]. The American Cancer Society estimates for lung cancer in USA for 2018, about 234,000 new cases and about 154,000 deaths [1]. Lung cancer is the leading cause of cancer-related deaths among men (26% of all deaths for cancer) and women (25% of all deaths for cancer) [1]. Lung cancer preferentially occurs in older people since most people diagnosed with lung cancer are 65 or older, while only a minority of patients is diagnosed younger than 45; the average age at diagnosis is about 70 years [1]. The incidence of lung cancer dramatically increased from 1930 to 2000, peaking in its incidence around 1990 in men and 2000 in women; after 1990 to today lung cancer mortality in men is decreasing and this trend started in women only after 2000–2005 [1]. The fact that lung cancer incidence and mortality rates continue to decline about twice as fast as in men than in women largely reflects historical differences in tobacco uptake and cessation [1].

A recent epidemiologic analysis provided evidence that incidence and mortality of lung cancer is markedly affected by socioeconomic status of various countries. Thus, country-specific human development index was strongly correlated with lung cancer incidence and mortality and, at a lesser extent, with Gross Domestic Product [2]. Among men 22 out 38 and 30 out 36 countries in the world showed declining incidence and mortality trends, respectively; in contrast, among women, 19 out 38 and 16 out 36 countries showed increasing incidence and mortality, respectively [2]. Among women, Brazil, Spain and Cyprus displayed the greatest incidence increase and all countries in Western, Southern and Eastern Europe showed increased mortality [2]. These observations support the hypothesis that the incidence and mortality rates of lung cancer are positively correlated with higher socio-economic development and productivity across various countries and indicate that greater disparity in the temporal trends of lung cancer among various countries are observed [2].

The most important risk factor for lung cancer is tobacco smoking. Globally, cigarette smoking by itself is responsible for over 80% of all lung cancer cases. In USA, for men who were current smokers, as compared with men who had never smoked, the relative risks of death from lung cancer were 12.2, 23.8 and 24.9 in the 1960s, 1980s and 2000s cohorts, respectively; for women who were current smokers, as compared with women who had never smoked, the relative risks of death from lung cancer were 2.7, 12.6 and 25.6 in the 1960s, 1980s and 2000s cohorts [3]. These observations have supported the view that women who smoke like men die like men for lung cancer. Convergence of the relative risks for men and women results from the convergence of smoking patterns among men and women since the 1960s [3]. It is important to point out that it was estimated that smoking causes about 25% of deaths among women and men 35 to 70 years in the United States [4]. It was estimated for men and women comprised between 25 and 79 years of age that the rate of death from any cause among current smokers was about three times that among those who had never smoked; the increased mortality among smokers was mostly due to neoplastic, vascular respiratory diseases life expectancy was shortened by more than 10 years among the current smokers, as compared with those who had never smoked [4].

Other factors contribute to lung cancer development, such as air pollution, indoor emission of fuel burning, environmental exposure to radon, asbestos, some metals such as chromium, cadmium and arsenic and some organic chemicals [5,6]. Studies carried out both in Europe and China have provided evidence that particulate matter air pollution contributes to lung cancer incidence [5,6]. Since these risk factors are preventable by smoking cessation and clear air initiatives, it is possible to reduce lung cancer incidence and mortality by population-based appropriate preventive strategies.

It is evident therefore that lung cancer represents a main medical problem and there is hope that that progresses in clinical treatment of this group of cancers could be achieved through improvements in our understanding of the molecular basis and tumor biology, particularly at the level of the cells that initiate the tumoral process. The most common lung cancer type is represented by non-small cell lung cancer (NSCLC, corresponding to about 90% of cases of lung cancer, the remaining ones being small lung cancer or SLC) which comprises three histological subtypes: adenocarcinoma, squamous cell carcinoma and large cell carcinoma. The large majority, up to 90% of patients with NSCLC, have history of tobacco smoking. The majority of NSCLC patients are diagnosed at advanced stage, when the various treatments cannot be curative.

In 2015, a new World Health Organization (WHO) classification of lung tumors was proposed [7]. This classification incorporates relevant histopathological and immunohistochemical findings. This new classification is important because is not only applicable on resection specimens, but also on small biopsies and cytological material [7,8]. This is particularly important in view of the fact that about 70% of patients with lung cancer present with advanced stages of disease and are not suitable for tumor resection [7,8]. It is important to note that this new classification includes also precursor lesions for both adenocarcinomas and squamous cell carcinomas. For resected adenocarcinomas, the definition of adenocarcinoma in situ and minimally invasive adenocarcinoma is important because identifies patients whi, if undergo complete resection, have a 100% probability of disease-free survival [8]. The major histological subtypes of malignant lung cancers are represented by adenocarcinoma, squamous cell carcinoma, neuroendocrine tumors (small cell lung cancer and large cell neuroendocrine carcinoma), large cell carcinoma, adenosquamous carcinoma and sarcomatoid carcinoma (Table 1). 

The stage classification of lung cancer includes the evaluation of the degree of progression of the primary tumor (T), the invasion of regional lymph nodes (N) by the tumor cells and by the presence of distant metastases (M) (Table 2). Importantly, the eighth edition lung cancer stage classification involves also the evaluation of carcinoma in situ and of minimally invasive carcinoma [9].

In the last 20 years dramatic progresses have been made in the understanding of the molecular abnormalities underlying lung cancers. These progresses have led to the development of targeted therapies and of a new generation of immunotherapy, resulting in an improvement of clinical outcomes for some lung cancer subtypes. However, main challenges still remain unresolved, including: (a) the dientification of new driver mutations to expand the population of patients that can benefit from targeted therapies, a problem particularly relevant for LSQCC and NSCLC; (b) a better understanding of the cellular and molecular mechanisms underlying resistance to targeted therapies, to try to prevent and eventually to bypass these resistances with the identification of more active single or drug combinations; (c) the identification and study of new drugs and of combination therapies based on rational pharmacological associations; (d) the identification of new biomarkers, able to predict the clinical responses to immunotherapy. The progresses achieved during these last years and those that could be made in the future years will require an integrated view of various aspects of lung cancer biology at cellular and molecular level, involving the analysis of genetic and epigenetic abnormalities of tumor cells, analysis of clonal evolution of tumor cell populations during spontaneous disease progression and under effect of various treatments, identification of tumor cell populations capable of initiating and maintaining the tumors process and the development of suitable animal models, reproducing the features of human tumors.

In this review, we provide an overview of the recent progresses made in lung cancer biology and treatment, suggesting that only a multidisciplinary and integrated approach will allow to improve the understanding and the treatment of these tumors.

## 2. Genetic Abnormalities in Lung Adenocarcinoma

NSCLC is a highly heterogeneous disease from a genetic point of view. The numerous studies carried out during these last years have led to the identification of numerous somatic mutations occurring in NSCLC. As for other tumors, the main aim of these studies consisted in the identification of driver mutations, i.e., of oncogenic mutations capable of driving the tumoral process: these mutations have been observed at the level of genes encoding signaling proteins, such as protein kinases and also at the level of GTPases [10]. In NSCLC the more frequently mutated genes with known potential function of driver genes are the following: *EGFR* (10−30%), *FGFR1* (20%), *KRAS* (15−30%), *PIK3CA* (2−5%), *ERBB2* (*HER2*) (2−5%), *BRAF* (1−3%), *ALK* (3%), *ROS1* (1%), *MAP2K1*/*MEK1* (1%), *RET* (1%), *NRAS* (1%) and *AKT1* (<1%) (reviewed in [10]). It is important to note that these various mutations are mutually exclusive, with the exception of *PI3KCA* mutations.

The tumor genomic landscape of tumors occurring in non-smokers and in smokers was recently compared and many remarkable differences have been reported: (a) mutation frequencies were higher in smokers than in never smokers tumor samples; (b) a different mutation spectrum in smokers (predominant C:G↣A:T) and never-smokers (C:G↣T:A) was observed; (c) distinctive sets of mutated genes in never-smokers (*EGFR* mutations and *ALK* and *ROS1* fusions) and smokers (*KRAS*, *TP53*, *BRAF*, *JAK2* and *JAK3* and mismatch repair genes mutations). The combination of mutational and gene expression data allowed to identify several pathways that are affected in lung adenocarcinoma: genes involved in extracellular matrix interaction, focal and adhesion, cell-cycle and JAK-STAT (*JAK2* is mutated in about 1% of NSCLCs) pathways are significantly enriched in lung adenocarcinomas [11]. Finally, the analysis of the variant allele frequencies for somatic mutations found in each tumor sample allowed to predict the number of the size of the clonal population in each tumor: it was estimated that about 40% of tumors were monoclonal and 60% multiclonal [11].

A recent study compared the use of next-generation sequencing to sequence the exons and genomes of DNA from a large number of adenocarcinomas. This analysis confirmed a high mutation rate of *TP53* (50%), *KRAS* (27%), *EGFR* (17%), *STK11* (15%), *KEAP1* (12%), *NF1* (11%), *BRAF* (8%), *SMAD* (4%). Other genes frequently mutated are *U2AF1* (3%), *RBM10* (7%) and *ARID1A* (8%). On the other hand, frequent copy number alterations have been observed: gain of *TERT* (42%), *MYC* (31%), *MCL1* (34%), *EGFR* (22%), *ERBB2* (20%), *NKX2-1* (18%); losses of *TP53* (18%), *CDKN2A* (24%, 10% homozygous) [12]. The analysis of the prognostic impact of these mutations showed that *TP53* and *U2AF1* mutation had both a negative prognostic impact and are associated with a reduced survival [12]. Interestingly, the analysis of the frequency of mutated genes in the context of cancer hallmarks provided a very interesting outline: 74% of tumors displayed mutations conferring resistance to cell death: 65% deregulating cellular energetics; 55% sustaining cellular proliferation; 63% evading growth suppressors; 38% enabling replicative immortality; 28% activating invasion and metastasis; 15% inducing angiogenesis and 42% inducing genomic instability and mutations [12].

A recent study carried out on a large number (230) of adenocarcinoma lung cancer provided a comprehensive molecular profiling of lung adenocarcinoma. The analysis of gene mutations showed that eighteen genes were currently mutated: TP53 was the most frequently mutated (46%); *KRAS* mutations (33%) were mutually exclusive with *EGFR* mutations (14%); another group of genes frequently mutated is represented by *BRAF* (10%), *PIK3CA* (7%), *MET* (7%) and *RT1*, a small GTPase (2%); a group of tumor suppressor genes, including *STK11* (17%), *KEAP1* (17%), *NF1* (11%), *RB1* (4%) and *CDKN2A* (4%), was also frequently mutated; another group of frequent mutations involve a set of chromatin modifying genes, such as *SETD2* (9%), *ARID1A* (7%) and *SMARCA4* (6%) was frequently mutated, as well as the two RNA splicing genes *RBM10* (8%) and *U2AF1* (3%); finally, mutations of the Max-interacting gene *MGA*, mutationally exclusive with *MYC* focal amplifications, are observed in 8% of patients [13]. Somatic copy number alterations involve amplifications of the *NKX2-1*, *TERT*, *MDM2*, *KRAS*, *EGFR*, *MET*, *CCNE1*, *CCND1*, *TERC* and *MECOM*, while *CDKN2A* gene was the most frequently deleted [12]. Analysis of aberrant RNA transcripts detected fusions involving *ALK*, *ROS1* and *RET*; *MET* exon 14 skipping in RNA, resulting in stabilized MET protein and activation. An overall view of the mutational status of the 230 adenocarcinoma patients showed that 62% of them display activating mutations in known driver oncogenes (such as *EGFR*, *KRES*, *BRAF* mutations, *ALK*, *ROS1* and *RET* fusions), the remaining 38% of patients was without any apparent *RTK*/*RAS*/*RAF* oncogene mutation. However, a careful analysis showed that *TP53*, *KEAP1*, *NF1* and *RIT1* mutations are enriched in the oncogene-negative group of lung adenocarcinomas. Taking into account the various genes mutated in lung adenocarcinomas, the most frequent biochemical pathways showing key alterations were represented by: RTK/RAS/RAF pathway (76%), PI3K-mTOR pathway (25%), p53 pathway (63%), cell cycle (64%), chromatin and RNA splicing (22%) [13]. It is important to point out that MAPK activation score is higher among KRAS mutant lung adenocarcinomas, but it is present also among *KRAS* WT adenocarcinomas. mTOR pathway may be activated in lung adenocarcinomas through three different molecular mechanisms: *PI3KCA* mutations, *STK11* mutation associated or not with low levels of LKB1 [10]. The integrative analysis of transcriptional and epigenetic profiling allowed to propose a molecular classification of lung adenocarcinoma subtypes. Taking into account the various studies on this topic [13,14], it was now proposed a molecular classification identifying the following subtypes: the terminal respiratory unit, also known as bronchioid; the proximal-inflammatory, also known as squamoid; the proximal proliferative, also known as magoid. The subtypes were associated with genomic alterations: the proximal proliferative subtype was enriched for *KRAS* mutations, *KEAP1* mutations, and with inactivation of the tumor suppressor *STK11*; the proximal inflammatory subtype was characterized by mutations of *TP53* and NF1 and by solid histopathology; the terminal respiratory unit, associated with a more favorable prognosis, was associated with *EGFR* mutations and fusions [13,14].

The chromatin remodeling pathway is frequently altered in lung cancer and could play a role in disease development. The SWI/SNF multiprotein complex is a key component of the chromatin remodeling machinery and plays a relevant role in the control of genomic plasticity. Deregulation of this pathway and mutation of the SWI/SNF subunits are observed in many tumors. Relevant abnormalities of the SWI/SNF subunits have been recently described also in NSCLCs; in fact, BRG1 and BRM are downregulated in 15−20% primary NSCLCs; ARID2 gene was found altered in 73% of lung adenocarcinomas and in 5% of samples the mutations predicted a truncated or absent protein [15].

**Figure 1:**
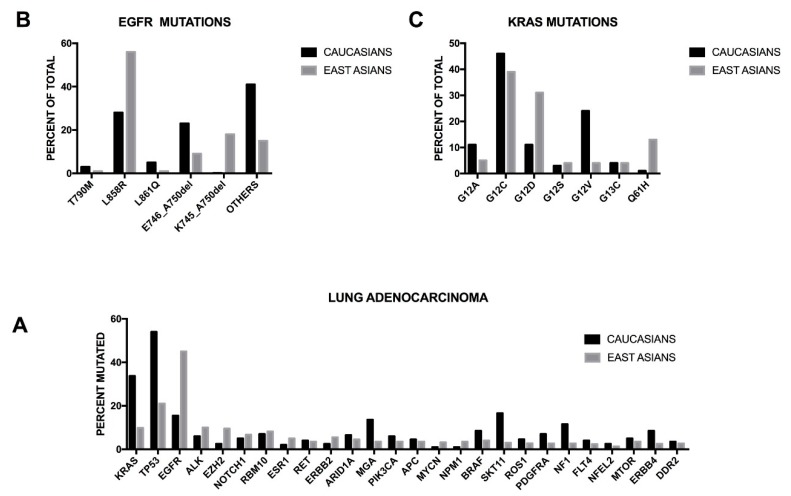
(**A**) Comparison of the most recurrent gene mutations in lung adenocarcinoma between Caucasians and EAST Asians; (**B**) Various gene mutations at the level of the EGFR gene in Caucasians and East Asians; (**C**) Various gene mutations at the level of the KRAS gene in Caucasians and East Asians. Data are reported in [13,17].

It is important to note that some remarkable differences have been observed in the incidence of some oncogenic mutations in lung adenocarcinomas of patients of different ethnical origin. Thus, East Asians display a higher rate of *EGFR* mutations and a lower rate of *KRAS* mutations than white populations. These differences have a considerable impact on the management of lung adenocarcinomas at the level of regional cancer centers and of the design of clinical trials in different countries. Recently, Sun and coworkers reported the mutational spectrum of lung adenocarcinomas occurring in East Asian non-smoker patients and showed that in a very high proportion (about 80%) of these patients *EGFR* mutations ae involved; in another 10% of these patients either *ELML-ALK* fusions or *HER2* insertions occur; finally, only 2% of these patients displayed KRAS mutations [16]. These conclusions were strongly supported by a recent study reporting the comprehensive genomic profiling of 255 Chinese lung adenocarcinomas, showing some remarkable differences in the frequency of mutation of several driver genes in comparison with the data reported in Caucasian patients [17]. Among the East Asian patients, the most frequently mutated genes are *EGFR* (46.7%, compared with 15% of TCGA data), *TP53* (21.2%, compared with 54.5% of TCGA data), *ALK* (12.1%, of whom 8.8% of mutation and 3.3% of rearrangement, compared with 5.8% of TCGA data), *KRAS* (9.8%, compared with 33.7% of TCGA data), *EZH2* (9.4%, compared 2.2 of TCGA data), *ERBB2* (5.5%, compared with 2.4% of TCGA data), *STK11* (3.1%, compared with 16.6% of TCGA data), *PDGFRA* (2.7%, compared with 7.0% of TCGA data), *NF1* (2.7%, compared with 11.6% of TCGA data) and *ERBB4* (2.4%, compared with 8.4% of TCGA data) (Figure 1) [17]. Clinically relevant genomic alterations were identified in 60.5% of East Asian patients [17].

Shi and coworkers have performed an integrative genomic analysis on primary NSCLC adenocarcinomas, corresponding to stage I to III tumors, almost exclusively (>90%) from smoker patients [18]. The results of this study were in large part confirmatory of those reported in the TCGA study; the most frequently mutated genes were: *TP53* (33.7%), *KRAS* (30.7%), *KEAP1* (23.8%), *STK11* (14.9%), *ARID1A* (7.9%), *SAMRCA4* (8.9%), *EGFR* (8.9%), *ATM* (7.9%) and *RBM10* (7.9%) [9]. They identified also two new putative driver genes: *POU4F2* (POU Class 4 Homeobox 2), mutated in 8.9% of cases and *2KSCAN1*, mutated in 5.9% of samples [18]. The analysis of set of mutually exclusive driver genes identified two sets of genes: *KRAS*, *EGFR*, *NF1*, *BRAF*, *MET* and *ZKSCAN1*; *STK11*, *EGFR*, *U2AF1* and *ERBB2* [18]. Finally, in the tumor evolution analysis, four driver genes had a significantly lower fraction of subclonal mutations, including *TP53*, *KEAP1*, *STK11* and *EGFR*, thus suggesting a tumor initiation role for these genes [9].

**Figure 2:**
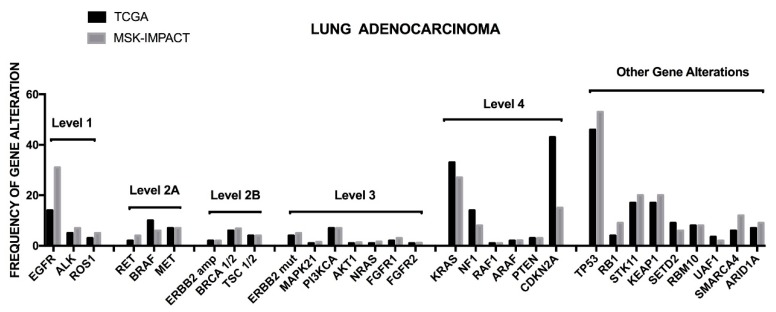
Frequency of main genetic alterations, subdivided into various levels according to the degree of therapeutic actionability of genetic events, in two groups of lung adenocarcinoma patients: TCGA data sets based on the analysis of non-metastatic patients and MSK-IMPAC data based on the analysis of recurrent/metastatic lung adenocarcinomas. Date are reported in [13,19].

The TCGA dataset was mainly based on the analysis of non-metastatic NSCLC adenocarcinomas, treatment naïve only. Recently, the Memorial Sloan Kettering Integrated Mutation Profiling of Actionable Cancer Targets (MSK-IMPACT) group reported the results of a large sequencing screening carried out on a large population of NSCLC adenocarcinomas with recurrent/metastatic disease, including analyses on tumors collected following treatment with at least one prior systemic therapy [19]. Basically, the profile of genetic alterations observed in the two studies was similar, with some remarkable differences concerning the frequency of some genetic alterations, such as *EGFR* mutations more frequent in the MSK-IMPACT cohort than in the TCGA cohort (27% vs. 11%); conversely, some drivers were present at higher rates in the TCGA cohort than in the MSK-IMPACT cohort, including *NF1* (8.3% vs. 2%) and *BRAF* mutations (7% vs. 3.6%). Interestingly, in this study potentially actionable genetic events were stratified into four different levels, from 1 to 4, classified according to clinical or laboratory evidence that the mutation confers increased sensitivity to specific targeted therapies [19]. The level 1 includes *EGFR* mutations, *ALK* fusions and *ROS1* fusions; the level 2A includes *RET* fusions, *BRAF^V600E^*, *MET* mutations and *MET* amplifications; the level 2B involves *ERBB2* amplifications, *BRCA 1–2* loss and *TCS 1–2* loss; the level 3 involves *ERBB2* mutations, *MAP2K1*, *FGFR3*, *PIK3CA*, *AKT1* and *ARAF1* mutations and *BRAF^V601E^* mutation; the level 4 involves *KRAS* mutations, NF1 loss, RAF1 mutations, *BRAF^non−V600E^*, *CDKN2A* loss, *MDM2* amplification and *EGFR*^exon20insertion^; in addition to these four level groups it is identified an unknown mitogenic driver set, characterized by frequent *TP53* (68%), *STK11* (37%), *KEAP1* (35%), *SMARCA4* (18%), *PTRD* (17%), *ARID1A* (16%) (Figure 2) [19]. While most of patients pertaining to the levels 1 and 2A were treated on a genotype-matched therapy, only a minority of patients of levels 2B, 3 and 4 were undergone to matched therapy [19]. A part of patients displays two or, more rarely, three targetable driver mutations.

The identification of these genetic abnormalities is important because it allowed to develop specific target treatments for some subsets of patients: phase II studies have shown that in some of these patients the response rate and progression-free survival are improved with targeted therapy compared with standard chemotherapy. Thus, targeted treatment is now approved for patients with *EGFR*-mutated and *ALK*-rearranged advanced lung adenocarcinomas. As above stated, these molecular studies have shown that in a significant proportion of lung adenocarcinomas recurrent mutations of driver genes have been identified, including *KRAS* and *NRAS* mutations, mutations in *ERBB2*, *BRAF*, *PIK3CA* and *AKT1*, recurrent gene fusions involving *ROS1* and *RET*, *MET* amplification, *MEK1* and *AKT1* mutations. A multiplexed assay of oncogenic drivers showed a mutation of these genes in 64% of lung adenocarcinomas [20]. However, this estimate is not based on the most sensitive next generation sequencing techniques. In fact, using a hybrid-capture-based next generation sequencing assay it was possible to show the presence of driver mutations in a group of lung adenocarcinoma patients resulted to be negative for driver mutations according to a standard, not-NGS assay: the most recurrent mutations observed in these patients were *TP53*, *EGFR*, *MDM2*, *KRAS*, *CDK4* and *SETD2* mutations [21]. Importantly, clinically-relevant mutations of *EGFR*, *BRAF* and *ALK*, *ROS1* and *RET* translocations have been observed in 26% of these “driver-negative” patients [21].

Interestingly and importantly, these therapeutic developments have modified the prognosis of many of these tumors. In this context, Lopez-Chevez and coworkers recently published the results of an interesting basket study describing the response to molecular target therapies of a large cohort of NSCLC patients. In this study, five biomarker-matched treatment groups have been evaluated: erlotinib for *EGFR*-mutated NSCLCs; seletinib for *KRAS*, *NRAS*, *HRAS* or *BRAF*-mutated NSCLCs; MK2206 for *PI3KCA*, *AKT* or *PTEN* mutations; lapatinib for *ERBB2* mutations or amplifications; sunitinib for *KIT* or *PDGFRA* mutations or amplifications [22]. Different survivals were observed for these different NSCLC subgroups: 3.51 years for *EGFR*-mutated patients; 2.9 years for *ALK* rearrangements; 2.3 years for *KRAS*-mutated patients; 2.2 years for those with other genetic abnormalities; 1.85 years for those without an actionable mutation.

Lung adenocarcinoma occurring in the young is not a well studied clinical entity, largely due to the fact that the median age of diagnosis is 70 years of age and less than 5% of patients are younger than 50 at diagnosis. However, recent studies have explored the mutational landscape of NSCLC occurring in the young, providing evidence that these tumors are enriched in targetable genetic alterations, such as *ALK* and *ROS1* rearrangements (whose frequency peak of incidence occurs in patients <40 years of age) and *HER* mutations (whose frequency peak of incidence occurs in patients of 41-50 years), while *KRAS* and *BRAF* mutations are more frequent among older patients [23]. Lung cancer of young patients is associated with a poor prognosis [23]. Tanaka and coworkers confirmed these findings in a group of 81 Japanese young lung adenocarcinomas, showing in these patients a very high frequency of *ALK* translocations (41%) and *EGFR* mutations (30%), but a low frequency of *KRAS* mutations (2%); furthermore, about 6% of these patients displayed *RET* or *ROS1* translocations [24].

The majority of studies to identify mutation hotspots in cancer, including lung adenocarcinoma, have focused on protein-coding regions in whole exome capture studies. Most of the genome not coding for proteins includes transcribed and not-translated exons of genes, introns, and noncoding regulatory genetic elements [25]. Lung adenocarcinomas harbor high burdens of neutral mutations [12]. The analysis of whole genome sequences has revealed the existence of rare noncoding mutation [13]. Whole genome sequencing analysis of lung adenocarcinomas revealed noncoding somatic mutational hotspots near *VMP1*/*MIR21* and insertion and deletion (indel) hotspots in surfactant protein genes (*SFTAB* and *STFPC*). These genes are the major transcriptional products of type II pneumocytes in the lung [25]. Through a statistical analysis of whole genome sequences across different types of cancers, it was provided evidence that other tumor types harbor similarly prevalent hotspots of noncoding somatic indel mutations, targeting a class of lineage-defining genes: albumin in liver carcinoma, gastric lipase in stomach carcinoma, thyroglobin in thyroid carcinoma [25]. These highly expressed genes define cell types that play key roles in the physiology of their respective organs and represent the precise cell of origin for the respective cancers [25].

Some of these mutations are clinically relevant and have been already translated into clinical applications (Table 3). For a detailed analysis of lung adenocarcinomas bearing these mutations and of the clinical implications see Supplementary File.

### 2.1. Abnormalities of DNA Methylation in Lung Adenocarcinoma

While numerous studies have analyzed the occurring structural somatic genetic abnormalities, few studies have analyzed the pattern of DNA methylation in lung tumors. The analysis of DNA methylation at the level of CpG island promoter regions allowed to identify three groups of lung adenocarcinomas according to the level of methylation: (i) a CIMP-H (high) group characterized by the hypomethylation of several genes (CDKN2A, GATA2, GATA4, GATA5, HOXA9, HOXD13, SOX17, WIF1), overexpression of WNT pathway genes and MYC overexpression; (ii) CIMP-I (intermediate); (iii) CIMP-L (low). No association was found between the level of DNA methylation and genomic alterations at the level of chromatin remodeling genes. However, it was observed an association between the expression of the chromatin modifying gene SETD2 and CDKN2A methylation. Tumors with low CDKN2A expression, as a consequence of gene promoter methylation were enriched for SETD2 mutations, have a lower ploidy and have a low mutation rate [4]. Recently, Karlsson and coworkers have reported a detailed analysis of the pattern of DNA methylation in lung cancers and through this analysis have identified clinically relevant subgroups of patients: one neuroendocrine and five adenocarcinoma epitypes [26]. The four epitypes of lung adenocarcinomas corresponded one to a global hypomethylation pattern (ES1), one resembling a methylation pattern of normal lung tissue (ES5), one displayed a promoter methylation pattern (ES4) and one displaying a pattern intermediate between ES1 and ES4 (ES2); the ES3 epitype corresponded to the neuroendocrine subtype and involved SCLC and LCNECs [26]. Important clinic-pathological differences have been observed between the various ES subgroups: the ES5 was enriched for never smokers, while never smokers were rarely classified among ES1; EGFR mutations were frequent in the ES5, but rare in the ES1 group; KRAS-mutated adenocarcinomas were enriched in the ES4 [26]. The number of mutations was decreasing from ES1 to ES5; the TP53, STK11 and KEAP1 mutations were most frequent among ES1 and ES2 subgroups [26]. ES2 and ES5 epitypes were associated with the best outcome, while ES1 and ES4 with the worst outcome [26].

The majority of studies on DNA methylation in lung adenocarcinoma lack mRNA expression data, mutation status and survival data. However, some recent studies addressed these issues. Thus, Bjaanoes and coworkers have reported a genome-wide DNA methylation in 164 lung adenocarcinomas; unsupervised hierarchical clustering of these tumors using the most variable gene regions allowed their separation into three clusters; the cluster 1 was enriched in TP53-mutated tumors and included 1641 differentially methylated gene regions, associated with TREM1 signaling pathway; the cluster 2 was enriched in tumors from never smokers and included 647 gene regions differentially methylated, associated with granulocyte and granulocyte adhesion; the cluster 3 was enriched in tumors from smokers, is scarcely EGFR-mutated, and included 1152 differentially methylate gene regions, associated with aryl hydrocarbon receptor signaling [27]. The progression-free survival of patients pertaining to cluster 2 is clearly better compared to that observed for patients pertaining to the clusters 1 or 3 [27]. Interestingly, lung adenocarcinomas associated with specific driver mutations, such as EGFR, ALK or ROS1 display a specific pattern of CpGs methylation [27]. A prognostic index based on DNA methylation levels of 33 CpGs was established and was found to be significantly associated with prognosis [27].

Epigenetic mechanisms greatly contribute to inter-tumoral and intra-tumoral heterogeneity of lung adenocarcinomas; through their contribution to intra-tumoral heterogeneity, epigenetic mechanisms play a key role in tumor progression and evolution [28]. Furthermore, the methylation of gene promoters of E-Cadherin, Snail1 and Twist1 plays a key role in the epithelial-to-mesenchymal transition of lung adenocarcinoma cells, an event strongly related to malignancy progression [28].

### 2.2. Genetic Abnormalities in Precursor Lesions of Lung Adenocarcinoma

Sequential premalignant lesions and consequent cellular and molecular changes have been poorly documented for lung adenocarcinomas. Atypical adenomatous hyperplasia (AAH) is the only sequence of morphologic change identified so far for the development of lung adenocarcinomas [29]. Microscopically, AAH dipslays localized proliferation of alveolar type II pneumocytes with mild to moderate cellular atypia. Some studies have suggested that in situ adenocarcinoma (AIS) could represent an intermediate lesion in the progression of AAH to lung adenocarcinoma [29]. AIS, formerly defined as bronchioalveolar carcinoma, is a non-invasive form of glandular neoplasia, exhibiting increased size, cellularity and morphology atypia [29]. Microscopically, this tumor is composed by atypical type II pneumocytes. Minimally invasive adenocarcinoma (MIA) represents a further step towards malignant adenocarcinoma and is defined as a small adenocarcinoma (≤3 cm) with a leptic pattern and invasion of 5mm or less in any one focus [29]. MIA was introduced as a new tumor entity between AIS and lepidic adenocarcinoma in the 2015 WHO classification of lung tumors. The link between AAH and invasive lung adenocarcinoma is supported by various observations: in fact, 5−20% of lungs resected for primary lung adenocarcinomas also harbor AAH, and AAHs harbor some of the genetic and epigenetic alterations observed in lung adenocarcinomas [29]. 

There is a lack of understanding of the molecular aberrations leading to the initiation and progression of AAH. Sivakumar et al. have molecularly analyzed 22 AAHs and showed that these lesions can be classified into three different subgroups with mutually exclusive and distinct driver gene mutation status: (i) BRAF-mutant; (ii) KRAS-mutant (ever-smokers only); (iii) KRAS/BRAF-WT AAHs. In this cohort of patients, BRAF oncogene was the most frequently mutated (four patients with K601E and one with N581S), followed by KRAS, predominantly mutated at the level of codon 12 [30]. Intriguingly and interestingly, no BRAF mutants were observed among the paired lung adenocarcinomas of BRAF-mutant AAHs; however, in four of the five BRAF-mutant AAHs, the paired lung adenocarcinomas exhibited driver EGFR mutations [30]. Conversely, lung adenocarcinomas of KRAS-mutant AAHs displayed several other driver mutations, including TP53, EGFR and KRAS [30]. The mutual exclusivity of BRAF and KRAS mutations in AAHs and the disparate pattern of mutations in the paired lung adenocarcinomas suggest divergent pathways in the pathogenesis of preneoplastic lesions of lung adenocarcinomas [30].

Izumchenko and coworkers have performed a targeted next-generation sequencing on multifocal AAHs and different zones of histologic progression within AISs and MIAs [31]. In this study, 25 distinct AAHs were discovered in the lung resection specimens derived from six different patients with invasive lung adenocarcinoma; the most frequently mutated genes were BRAF (16%) and ARID1A (16%); EGFR and MALM1 were mutated in three AAHs in two of the six patients; TP53 and KRAS were mutated in several AAHs within the same tumor; alterations in growth factors other than EGFR are observed in 28% of AAHs [31]. Multi-region sequencing of MIAs and AISs demonstrated different genetic drivers within the same tumor and showed also that clonal expansion is an early event of tumorigenesis [31]. BRAF was the most mutated gene in AAH lesions, but three of the four patients with mutated BRAF in AAHs had not mutated BRAF in the matched lung adenocarcinoma [31]. The most likely interpretation of these observations is that the invasive clone rarely develops from a lesion with a *BRAF* mutation, in line with the observation that the frequency of BRAF mutations in invasive lung adenocarcinomas is low (2−3%). *EGFR* and *TP53* are important gene drivers in the early stages of lung adenocarcinoma development, but their frequency increases with advancing steps of histologic progression and were always present at the MIA stage; interestingly, the fractional abundancy of *TP53* and *EGFR* mutations is higher in MIAs than in AISs [31]. *KRAS* mutations were seen in AAHs and paired adenocarcinomas, supporting a role for *KRAS* mutation as an early genetic event during lung tumorigenesis [31].

These studies have shown that the frequencies of *KRAS* and *BRAF* mutations are higher in preinvasive tumors than in invasive tumors, while the frequency of *EGFR* mutations in preinvasive lesions is like that observed in invasive tumors. These observations may be explained assuming that *BRAF* or *KRAS* mutations are able to induce cell proliferation and cellular atypia, but in the absence of additional genomic alterations are unable to induce invasive tumors.

## 3. Genetic Abnormalities of LSQCC

Lung squamous cell carcinoma (LSQCC) is characterized by a genomic signature of tobacco use, with the majority (>90%) of affected patients being smokers. LSQCCs exhibit a somatic mutation burden and pattern like that of patients with SCLC or other tobacco-related tumors [32]. As above mentioned, in lung adenocarcinoma patients the mutation burden is variable and is related to the smoking status, being much lower in non-smoker patients [32]. This link of LSQCC to a main causative event is also supported by the finding that the molecular abnormalities observed in this tumor are homogeneous at the level of various populations of different ethnical origin. Furthermore, there is a consistent similarity between LSQCC and other squamous tumors, such as head and neck squamous cancers.

LSQCC is a common type of NSCLC that seems to have a pattern of gene mutations in large part different from those observed in adenocarcinomas. Some recurrent and typical chromosomal alterations are observed in LSQCCs, responsible for allelic losses or allelic amplifications. Allelic losses are typically observed at the level of chromosomes 3, 5, 9, 13 and 17, while the most relevant allelic amplification involves 3q [32]. The most relevant events for the biology of LSQCCs occur at the level of amplification of 3q and allelic losses at the level of 3p and 9p chromosomal regions. The 3q region contains several potentially interesting genes, including EPHB3, PIK3CA, SOX2 and TP53 and its amplification is observed in the large majority of LSQCCs, but only in <20% of lung adenocarcinomas. It is very important to point out that the development of 3q amplification is associated with tumor progression, this abnormality being absent in low-grade LSQCCs and is present in virtually all high-grade LSQCCs [32]. The chromosomal region 3p contains various tumor suppressor genes, such as FHIT, FUS1 and VHL: allelic losses in this region have been observed frequently in preneoplastic lesions and virtually in all cancer lesions [32]. Deletions at the level of chromosome region 9p determine loss of heterozygosity at the level of the loci of two important tumor suppressor genes such as CDKN2A and PTPRD [32].

Activating EGFR mutations, as well as ALK fusions, are typically not present in LSQCC. Particularly, Rekhtman et al. have carefully assessed the occurrence of EGFR and KRAS mutations in LSQCC and have observed that mutations of both these genes were absent in typical cases of LSQCC; rare cases positive for EGFR or KRAS mutations can be identified as rare mixed squamous-adenocarcinomas [33]. Single platform genomic studies have identified some regions of somatic copy alterations, such as amplifications of FGFR1, PDGFRA and SOX2 and deletions of CDKN2A [34].

Recently, a comprehensive study of the genetic abnormalities occurring in LSQCC was performed in the context of the Cancer Genome Atlas Project, providing a fundamental integrated analysis of DNA copy number abnormalities, somatic exonic mutations, mRNA sequencing, mRNA expression and promoter methylation, microRNA analysis and whole genome sequencing [35]. The results of this complex analysis provided a comprehensive landscape of genomic and epigenomic alterations occurring in LSQCC. The most prominent findings of this analysis were the following: (a) 10 genes were found to be frequently mutated and they include TP53 (mutated in about 90% of cases), CDKN2A (15%), PTEN (8%), PIK3CA (16%), KEAP1 (12%), MLL2 (20%), HLA-A Class I Major Histocompatibility Gene (3%, loss of function mutations), NFE2L2 (15%), NOTCH1 (8%, truncating mutations seemingly associated with loss-of-function), RB1 (7%) [35]; (b) Many of the frequent somatic mutations found in LQSCC are drivers of important pathways involved in tumor initiation or in tumor progression: oxidative stress response is altered in 34% of cases through KEAP1, CUL3 and NFE2L2 mutations; cell differentiation genes (SOX2, NOTCH1, NOTCH2, TP63, FOXP1) are altered in 44% of samples; cell cycle genes such as CDKN2A and RB1 in 72% of tumors; PI3K pathway genes in 47% of tumors [35]. Recently, the mutational spectrum of LSQCC in East Asian patients was reported, showing a similar pattern to that observed in white populations [36]. The following rates of recurrent gene mutations were observed: TP53 (73%), MLL2 (24%), NFEL2 (17%), RB1 (15%), PTEN (11%), KEAP1 (16%), PI3KCA (9%), CD117 (13%), NF1 (12%), SWI/SNF (15%), NOTCH (15%). Frequent copy number alterations were observed at the level of: SOX2 (79%), PI3KCA (77%), TP63 (75%), FGFR1 (31%), CDKN2A (38%), PDGFRA (18%), PTEN (31%) [36].

Li and coworkers have reported the molecular analysis of 198 Chinese LSQCC by wide exome sequencing and basically showed a pattern of the most recurrently mutated genes in agreement with the two studies performed in Caucasian and Korean LSQCC patients [37]. At variance with the two previous studies, these authors reported in Chinese patients the frequent mutations of the CHD10 gene, mutated in 14.8% of these patients; a part of the CDH10-mutated LSQCCs have two or three independent mutations, seemingly inactivating the two alleles; CDH10 could act as a potential tumor-suppressor gene in LSQCC [37]. This study showed also that some biochemical pathways are frequently mutated in LSQCC and their consequent deregulation could play a relevant role in LSQCC pathogenesis: cell adhesion/Wnt/YAP in 76% of cases; oxidative stress response in 21% of cases; PI3K/RTK/RAS signaling pathway in 37% of cases [37]. Tao and coworkers have analyzed 157 Chinese LSQCC patients at the level of 50 oncological-relevant genes and observed that among these genes the most frequently mutated genes were TP53 (56%), CDKN2A (8.9%), PIK3CA (8.9%), KRAS (4.5%), EGFR (3.2%), FBXW7 (2.5%), PTEN (2.5%), FGFR3 (1.9%) AKT1 (1.3%) and KIT (0.6%). EGFR mutations were more frequent in non-smokers than in smokers (23.5% vs. 0.7%), while TP53 mutations were more frequent among smokers than in non-smokers (59% vs. 29%) [38]. 76% of samples displayed copy number alterations at the level of FGFR1 (16%), EGFR (14%) HER2 (9.6%), PDGFRA (7.6%), CCND1 (14%), SOX2 (31%), CDKN2A (21.7%) and PTEN (16.6%) [39]. Interestingly, the overall analysis of the results obtained in this screening revealed that about 93% of these patients harbored abnormalities (Mutations or amplification/deletion or deregulated expression) which are potentially druggable targets for anticancer therapy [39].

The majority of LSQCC are centrally located (c-LSQCC); a minority of these tumors is located in the peripheral lung (p-LSQCC). Interestingly, p-LSQCC have more mutations of EGFR than c-LSQCC (6.2% vs. 2.2%); furthermore, p-LSQCCs have the tendency to higher frequency of PIK3CA, KRAS and HER2 mutations than c-LSQCC (4.8% vs. 2.2%; 3.4% vs. 1.5%; 1.4% vs. 0%) [40].

About 5% of LSQCC patients are never smokers [41]. Lee and coworkers have explored the mutational spectrum in a population of never smokers LSQCC patients [41]. The frequency of the mutations at the level of the main driver genes was similar in never smoker and ever-smoker patients: TP53 (75% vs. 93%), RAS (66.7% vs. 71.4%), KIT (25% vs. 43%), EGFR (25% vs. 14.3%), PTEN (8.3% vs. 28.6%) [41]. The overall survival of never-smoker patients was similar to that observed in ever-smoker patients, matched for tumor stage and age [41].

Okamoto and coworkers have subdivided LSQCCs according to the number of their mutational load and observed that tumor with high mutation burden tended to be less differentiated and preferentially located in the upper or middle lobe [42]. SOX2 and TKNR2 amplifications are associated with high tumor burden [42].

Campbell and coworkers have performed a comparative analysis of the patterns of somatic genomic alterations observed in lung adenocarcinomas and LSQCCs. This comparison was based on the analysis of a large set of tumor samples. The median mutation rates are 8.7 mutations/Mb in lung adenocarcinomas and 9.7 mutations/Mb for LSQCCs [43]. At the level of gene mutations, 38 genes were found as recurrently mutated in lung adenocarcinomas and 20 in LSQCCs; only six genes, including TP53, RB1, ARID1A, CDKN2A, PI3KCA and NF1, were recurrently mutated in both types, with TP53, CDKN2A and PIK3CA being more frequently mutated in LSQCCs than in lung adenocarcinomas [43]. Mutated and amplified genes in LSQCC most closely resembled the genes mutated in hand and neck squamous cell carcinomas and in bladder cancers, tumors related to smoking, as well as LSQCC [43]. In contrast, significantly mutated genes in lung adenocarcinomas are more similar to those observed in glioblastoma and colon cancer [43]. Some genes significantly mutated were exclusively mutated in lung adenocarcinoma, such as STK11 (LKB1), RBM10, KEAP1, RIT1 and MET, while other genes such as NFE2L2, KDM6A, RASA1, NOTCH1 and HRAS are significantly mutated in LSQCC [43]. The most significantly focally amplified genes in lung adenocarcinomas are NKX2-1, MYC, TERT, MCL1 and MDM2, while in LSQCC the most amplified were SOX2, CCND1, FGFR1, MYC, YES1, MIR205 and EGFR [43]. In conclusion, both mutated genes and recurrent somatic copy number alterations are largely distinct for lung adenocarcinomas and LSQCCs [44]. These observations are consistent with gene expression studies showing that while lung adenocarcinoma makes part of a single tumor group composed only by this tumor, LSQCC makes part together with head and neck squamous cancers and with a bladder subtype of one tumor group characterized by TP53 alterations [45].

### 3.1. LSQCCs with FGFR Alterations

DNA sequencing studies have shown recurrent mutations at the level of some genes, including TP53, NFE2L2, BA13, KEAP1, MUC16, GRM8, FBXW7, RUNX1T1, STK11 and ERBB4. Some of these genetic abnormalities are druggable, i.e., they can be targeted with some drugs and may open the way to new, possible efficacious therapies for this lung cancer type. Thus, two studies have shown that more than 20% of squamous adenocarcinomas exhibit the amplification of chromosome segment 8p11-12 containing the FGFR1 gene [46,47]. Importantly, treatment of lung cancer cell lines bearing FGFR1 amplifications with FGFR inhibitors inhibits cell growth and survival [46,47]. These observations suggest that FGFR1 may represent a promising therapeutic target in non-small cell lung cancer [46,47].

In addition to FGFR1 amplification, Liao and coworkers reported oncogenic FGFR2 and FGFR3 mutations (each with a frequency of about 3%) [48]. These mutants were constitutively active and are sensitive to the inhibitory effects of drugs targeting FGFRs [48].

Flockerzi and coworkers have investigated FGFR1 amplifications in 101 LSQCCs and have reached the conclusion that FGFR1 is a frequent alteration (22%) in LSQCC and appears not to be a negative, but rather a favorable prognostic marker for women and particularly for patients with advanced LSQCC, stage III-IV [49].

Preclinical studies have shown that FGFR alterations predict sensitivity to FGFR inhibitors. Numerous clinical trials are in progress to evaluate selective and nonselective FGFR inhibitors, including Dovitinib (a FGFR1, FGFR2 and FGFR3 inhibitor), Lucitanib (a FGFR1/2/3 and VEGFR inhibitor), JNJ-42756493 (oral pan-FGFR inhibitor) and BGJ398 (a FGFR1/2/3 inhibitor). The FGFR 1–3 inhibitor BGJ398 was evaluated in patients with advanced solid tumors, including NSCLC patients, including LSQCCC patients [50]. Interestingly, anti-tumor response (partial responses) was observed in 11% of patients with amplified FGFR1 LSQCCs [50]. Importantly, this drug had a tolerable a manageable safety profile [50]. 

It is important to note that not all LSQCC cells displaying FGFR1 amplification are sensitive to FGFR inhibition, thus indicating that selection of patients exclusively based on gene amplification is not the best predictor of response to therapy [51]. Predictors of sensitivity of FGFR1^ampl^ LSQCCs to FGFR1 inhibitors are represented by autocrine FGF production and MYC overexpression [51].

The results of in vitro and in vivo clinical studies clearly indicated that not all FGFR1-amplified LSQCCs are sensitive to FGFR inhibitors and this observation suggests that some mechanisms of primary resistance are frequent in FGFR1-amplified lung cancer [52]. The analysis of these resistance mechanism showed that two different pathways that cause emergence of resistance, both pathways leading to MAPK reactivation: NRAS amplification and DUSP6 deletion; MET upregulation [52]. These resistance mechanisms can be bypassed by appropriate combination therapies [52].

Some recent studies have explored the mechanisms of resistance of LSQCC to FGFR1 inhibitors and have also suggested some strategies to bypass this resistance. Co-clinical trials showed that not all xenografts derived from LSQCCs with FGFR abnormalities are sensitive to Dovitinib, an FGFR inhibitor [53]. Transcriptional activation of 18 key signaling components of the FGFR pathway, but not the type of FGFR alteration present in tumor cells, predict sensitivity to Dovitinib [53]. The study of tumor cell lines chronically exposed to the FGFR inhibitor BGJ398 showed that AKT activation mediates the development of resistance to this inhibitor [54]. Weeden et al. have investigated xenograft models of FGFR1 overexpressing LSQCCs and observed that only the combined therapy, but not the single-drug therapy, with cisplatin and a FGFR inhibitor, elicited a marked anti-tumor cytotoxic effect and markedly prolonged animal survival [55].

FGFR1 controls glucose uptake and utilization by activating the AKT/mTOR pathway, which in turn in involved in the induction of GLUT-1 glucose transporter expression; FGFR inhibitors exert their anti-tumor activity also through the inhibition of glucose metabolism through AKT/mTOR inhibition [56]. The combination of FGFR inhibitors with agents targeting AKT/mTOR signaling pathway increases the anti-tumor effects induced by FGFR inhibition; this drug combination could represent a new therapeutic strategy for FGFR1-amplified LSQCCs [56].

### 3.2. LSQCCs with DDR2 Mutations

Another druggable genetic abnormality of LSQCCs represented by mutations of DDR, a receptor tyrosine kinase able to bind collagen as its endogen ligand, occurring in about 4% of cases [57]. DDR mutations were shown to be oncogenic and their transforming activity can be blocked by the tyrosine kinase inhibitor dasatinib [57]. Interestingly, in a clinical trial a subject with a LSQCC with a DDR2 kinase domain mutation, responded to treatment with dasatinib and erlotinib [57].

In the various studies of characterization of molecular alterations observed in LSQCCs, a rate of DDR2 mutations ranging from 1.1% to 4.6% was reported; this great variability may be related to a heterogeneity of LSQCCs, but also to differences in DNA sequencing methodology, not always covering all gene coding-sequences [57]. In a recent study carried out on French patients and based on the sequencing of 271 LSQCCs, a frequency of 4% of DDR2 mutations was observed; importantly, DDR2 mutations were not mutually exclusive with other driver alterations [58]. Lee and coworkers reported that a mutation in DDR2 occurs with a frequency of about 2% in Korean lung SQCC patients. Interestingly, these authors reported two novel DDR2 mutations, both located in a kinase domain and inducing an increased proliferation rate [59]. 29% of primary LSQCCs display an overexpression of DDR2 [60]. Interestingly, experiments of enforced expression of DDR2 enhanced lung metastases in animal models [60].

A DDR2 mutation (L63V) in combination with TP53 loss induced in mice poorly differentiated lung adenocarcinomas with a high penetrance (100% of animals). Importantly, mice with DDR2-WT and TP53 loss did not develop lung tumors [61]. Tumors generated in DDR2-mutant/TP53-loss mice display squamous cell markers, such as p63 and SOX2. These tumors were sensitive to Dasatanib and BET inhibitor JQ1 [61]. Adaptive responses to dasatinib treated LSQCCs harboring DDR2 mutations limit the response to this inhibitor [62]. A small-molecule chemical library screen showed that MET and insulin-like growth factor receptor inhibitors cooperated with Dasatinib in inducing death of DDR2-mutant LSQCCs [62].

### 3.3. LSQQ with SOX2 Amplification

Broad 3q chromosome amplification is widely recognized as the most common chromosome abnormality found in LSQCC, where SOX2, PIK3CA, ACK1, PRKC1, TP63, PDL1, ECT2 and other genes are located [36].

It is of interest to note that the SOX2 gene is much more frequently amplified in LSQCC (72%) than in lung adenocarcinomas (8%). The presence of SOX2 amplifications and high Sox2 protein expression was associated with a peculiar histological subtype characterized by basaloid differentiation and nuclear atypia [34]. Elevated SOX2 protein levels were associated with a better overall survival in LSQCC patients [63]. SOX2 amplifications are preferentially observed in LSQCCs exhibiting TP53 mutations; furthermore, it was observed in various LSQCC specimens a very good correlation between SOX2 mRNA and T53 levels [64]. Importantly, SOX2 gene amplifications are associated with FGFR1 and PIK3CA gene gain in LSQCC, and therefore it seems logical to assume that these gene gains occur concomitantly [65]. Another study showed that Sox2 is co-amplified with PRKCI (Protein Kinase C Iota), both genes being present on chromosome 3q26 [66]. As will be discussed later, these two amplified genes cooperate to drive a stem-like phenotype in LSQCC [66]. 

SOX2, as a transcription factor, mainly exerts its oncogenic activity by modulating gene expression. In this context, a recent study showed that SOX2 suppresses CDKN1A (a cell cycle inhibitor) expression and, through this mechanism, sustains the growth of lung squamous tumor cells [67].

Very importantly, a recent study showed that SOX2 could act as a master regulator of tumor squamous cell differentiation [68]. In fact, SOX2 overexpression in tracheobronchial cells combined with CDKN2A and PTEN loss results in the generation of LSQCC, closely resembling the human counterpart [68].

Importantly, amplification of distal 3q, with consequent SOX2 gene amplification, is an early genetic event during squamous lung cancerogenesis: in fact, this abnormality is observed in high-grade, but not in low-grade, bronchial dysplastic lesions [69]. SOX2 amplification is a common event in squamous cell carcinomas of various organ sites, including lung, esophagus, cervix uteri, skin and penis [70].

Various studies have shown that high levels of SOX2 amplification are associated with a better prognosis of LSQCC. Thus, Wibertz and coworkers have studied two cohorts of LSQCCs, for a total of 891 patients and have observed that 8% of these patients display a high level of SOX2 amplification, associated with lower tumor grade and a prolonged overall survival [71]. Yoon and coworkers showed that SOX2 overexpression is a positive prognostic factor in patients with stage III LSQCC undergoing adjuvant radiotherapy [72]. Zheng and coworkers analyzed a group of Chinese patients and observed an association between SOX2 amplification /overexpression and FGFR fusion genes and a better RFS and OS in these patients, compared to those not overexpressing SOX2 [73]. These conclusions were supported also through the meta-analysis of the literature data, thus indicating that SOX2 amplification is favorable for overall survival in LSQCC [74].

### 3.4. Gene Expression Classification of LSQCC

Whole-transcriptome expression profiles generated by RNA sequencing allowed to classify LSQCC into four different subgroup signatures that were defined as classical (36%), primitive (15%), basal (25%) and secretory (25%) [75]. Importantly, a consistent correlation between expression subtypes and genomic alterations in copy number, mutation and methylation was observed. The classical subtype was characterized by alterations in KEAP1, NFE2L2 and PTEN genes, overexpression of SOX2, TP63 and PI3KCA (all present on 3q), high PI3KCA expression, frequent chromosome instability; the primitive expression subtype was characterized by RB1 and PTEN alterations, frequent chromosome instability; the basal expression subtype typically showed NF1 alterations. CDKN2A alterations were frequent in all lung cancer expression subtypes [75]. The primitive subtype may derive from a later stage of differentiation, while the basal type derived from an earlier differentiation stage [75]. Among the four LSQCC subtypes, the primitive subtype has the worst prognosis and the basal subtype has better prognosis than the other subtypes [76]. Studies with cell lines corresponding to the various subtypes indicate a differential sensitivity to various anti-tumor drugs; the secretory-type cell lines are significantly less sensitive to anticancer drugs [76].

A recent study explored the tumor immune landscape in the various LSQCC subtypes. The secretory subtype showed consistently higher immune cell expression of both innate and adaptive immune cells; the classical subtype demonstrated the lowest immune cell expression of all LSQCC subtypes; CD271 (PD-L1) expression did not correlate with other immune cell expression in the various LSQCC subtypes; major histocompatibility complex II expression was higher in the secretory subtypes than in the other subtypes, while NRF2 expression was clearly higher in the classical subtype than in the other subtypes [77]. Increased immune cell expression is not consistently associated with improved survival and appears to be expression subtype-dependent [77].

## 4. Genetic Abnormalities in Adenosquamous Lung Carcinomas

A peculiar group of NSCLCs is represented by adenosquamous lung carcinomas, a tumor subgroup containing both adenocarcinoma and squamous cell carcinoma. A recent study reported the main molecular features of these tumors, showing in 56% of cases known mutant kinases, such as *EGFR* mutations (31.6%), *KRAS* mutations (10.5%), *AKT1* mutations (2.6%), *ERBB2* insertion mutation (1.3%), *PI3KCA* mutation (1.3%), *ALK* gene fusions (5.3%) and *KIF5B-RETR* fusions (4%); no *BRAF* mutations have been detected. The mutational profiles and clinicopathologic features of classical adenosquamous NSCLCs is very similar to that of poorly differentiated adenocarcinomas [78]. In selected patients, a microdissection analysis of the adenomatous and squamous components present in each tumor was performed, showing that both exhibit the same EGFR or KRAS mutations [79].

Shi and coworkers have characterized the molecular abnormalities in a group of 56 Chinese adenosquamous carcinomas, confirming in large part the results previously reported: particularly, they observed 55.4%, 7.1%, 1.8% and 5.4% for EGFR, KRAS, BRAF and PIK3CA mutations, respectively [80]. All PIK3CA mutations co-occurred with EGFR mutations [80]. No ALK and DDR2 mutations were observed. The clinicopathological characteristics of EGFR-mutated and KRAS-mutated adenosquamous cancers is comparable to those observed for their adenocarcinoma counterpart [80].

Shi and coworkers have explored 51 adenosquamous carcinomas for PD-L1 expression and showed that this check point ligand was preferentially expressed in the squamous component, compared to the adenocarcinoma component [81]. PD-L1 expression correlated with the lymphovascular invasion [81]. According to these findings, it was concluded that anti-PD-L1 is a promising treatment option in lung adenosquamous carcinomas in which PD-L1 expression is high and EGFR mutations are present [81].

As above mentioned, the two histological components of adenosquamous carcinomas share identical oncogenic mutations, suggestive of a potential transition, transdifferentiation from lung adenocarcinoma to squamous cell carcinoma [82]. Recent studies in experimental models support the transdifferentiation of adenocarcinoma cells to squamous carcinoma cells [82]. In this context, also clinical studies have provided evidence that EGFR-mutant lung adenocarcinoma can transdifferentiate to squamous cell carcinoma in relapsed cancer patients [82]. Thus, the adenocarcinoma to squamous cell carcinoma transdifferentiation may represent a mechanism involved in the pathogenesis of adenosquamous carcinomas and in the development of drug resistance [82].

## 5. Genetic Abnormalities in Basaloid Lung Carcinomas

In the 2015, the classification scheme of World Health Organization proposed a new classification of LSQCC subtypes, identifying three subtypes: keratinizing (the most frequent, 65−70%), nonkeratinizing (with intermediate frequency, 25−30%) and basaloid (the less frequent, about 5%). A recent study explored a large set of Chinese LSQCCs and showed that basaloid LSQCCs do not display any typical clinicopathologic feature compared to the other two subtypes [83]. It is commonly accepted that basaloid LSQCCs have a worse prognosis, but this finding was not confirmed in all studies.

The molecular features of these tumors were recently reported. There are two types of basaloid lung cancers, one is the pure basaloid type and the other one is the squamous variant of the basaloid carcinoma. Gene expression profiling studies showed a number of typical abnormalities in basaloid lung cancers, consisting in: (a) overexpression of genes related to TP53 mutation signature, transcription factors (such as SOX4, SOX9, SOX11 and MYB), methylation regulation (such as DNMT1 and DNMT3), cell cycle, survival (such as BCL2) and embryonic development (such as FGF3 and FGF9); (b) down-regulation of genes related to squamous cell differentiation, such as genes related to keratinocyte differentiation [84]. Concerning the quantitative and qualitative molecular abnormalities basaloid tumors more resemble SCC than adenocarcinoma [84].

### Common Genetic Abnormalities in Squamous Cell Carcinomas

Recently, the somatic genetic abnormalities observed in LSQCC have been compared to those observed in other squamous cancers (bladder, oral cavity) and this analysis led to the identification of some common features: all these tumors defined as C2-squamous-like tumors, arise from a cellular subtype shared between epithelial surfaces exposed to environmental influences and display common genetic features at the level of a set of dysregulated genes, mainly represented by SOX2 and ΔNp63 due to gene amplification [85].

**Figure 3:**
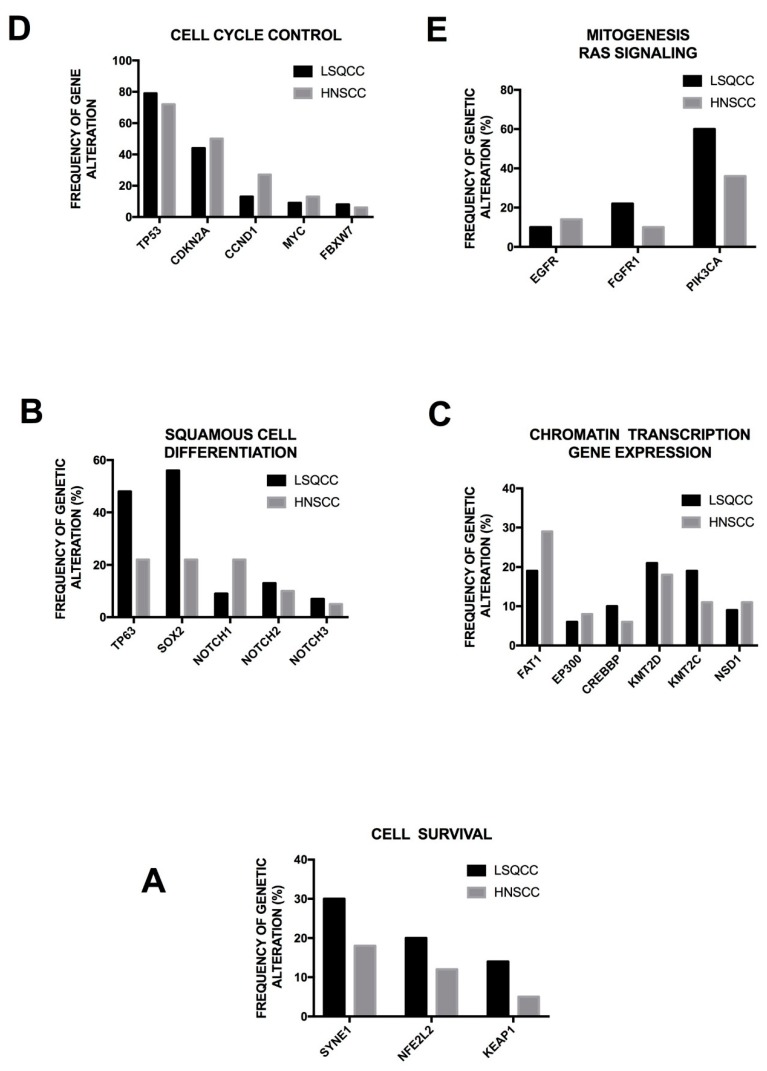
Pattern of frequently altered genes in LSQCC and HNSCC, subdivided according to their biologic function. A: Cell Survival; B: Squamous Cell Differentiation; C. Chromatin Transcription Gene Expression; D: Cell Cycle Control; E: Mitogenesis, RAS Signaling. Data are reported in [85].

The comparison of genetic abnormalities observed in the various squamous cell cancers show strong similarities between these tumors; particularly, the comparison of the genetic abnormalities observed in LSQCCs and HNSCCs shows a very high similarity in the type and also in the frequency of these various genetic abnormalities (Figure 3) [85]. Some peculiar features distinguish squamous cell cancers from other tumors, particularly related to specific determinants of squamous differentiation, such as NOTCH, TP63 and SOX2 [85]. These observations support a unified perspective on the biology of squamous cell cancers and support the translation into new common therapeutic approaches [85].

These molecular studies have led to a classification of NSCLCs biologically and clinically relevant. The ensemble of molecular studies has provided the recognition of the main somatic genetic lesions occurring in the two main tumor subtypes: thus, EGFR and KRAS mutations and ALK-EML4 fusions mainly occur in adenocarcinomas, while DDR2, FGFR2 and NFE212 mainly occur in squamous cell carcinomas. Taking into consideration the data in genomic alterations, linked to histomorphological and immunohistochemical data of the tumors and to patient outcome, clinically relevant lung tumor subgroups have been identified. Particularly, this study showed that: (a) the analysis of BRAF, EGFR, KRAS, PI3KCA mutations, ALK fusions and FGFR1 amplifications is clinically relevant and helps to define tumor subgroups that can take benefit from patient individualized therapies; thus, the EGFR-mutant lung cancers can be subdivided into two different subgroups according to the presence (poorer prognosis) or absence (better prognosis) of TP53 mutations; TP53-mutant tumors can be subdivided into two subgroups, one with RB1 no loss (with a better prognosis) and the other one with RB1 loss (with a poorer prognosis) [86]. However, it is important to point out that using the actual available therapies (surgical debulking, chemo-radiotherapy) the prognosis of the different NSCLC subgroups subdivided according to the presence of BRAF, EGFR, KRAS or PI3KCA mutations or FGFR1 amplifications or ALK-EML4 fusions is not prognostically relevant [87]. In spite this limitation, this classification is very important because these different NSCLC subtypes can be addressed, in some instances, to individualized treatments based on selective molecular targeting.

## 6. Intra-Tumor Heterogeneity and Tumor Evolution

Two recent studies provided very important informations about the phenomenon of tumor heterogeneity and clonal/subclonal evolution in NSCLC. These studies were based on multiregion sequencing of a certain number of lung cancers surgically resected [88,89]. This analysis allowed to define three types of genetic alterations in lung tumors: “trunk” mutations represent ubiquitous mutations present in all regions of the tumor; “branches” mutations representing heterogeneous mutations present only in some regions of the tumor; “private” mutations representing mutations present only in one region of the tumor. These different mutations underline a progressive mutational evolution of the tumor, with trunk mutations occurring early, before, branch mutations, occurring at later times during tumor progression [88,89]. Both these studies provided evidence about a consistent degree of spatial heterogeneity, being variable from one tumor to another: on the average, 60−70% of all mutations were detectable in all regions of the same tumor, while the remaining 30−40% were represented by spatially heterogeneous (branch or private) mutations [88,89]. In one of these two studies it was noted that there was a marked intra-tumor heterogeneity in translocations, mutations and copy number alterations associated with APOBEC cytidine deaminase activity (thus, driver genes PIK3CA, EP300, TGFBR1, PTPRD and AKAP9 harbored mutations in an APOBEC context), thus providing evidence about a possible functional impact of APOBEC activity on subclonal expansion [88]. In the other study it was noted that the early relapsing patients had significantly larger subpopulations of subclonal mutations in their primary tumors than patients without relapse [89].

Jamal-Hanjani and coworkers have performed a wide analysis of intra-tumor heterogeneity on 100 NSCLC patients who had not received previous systemic therapy; at least three different regions of each tumor were analyzed [90]. This analysis showed a consistent degree of intratumor heterogeneity, with a median of 30% of somatic mutations identified as subclonal and a median of 48% of copy-number alterations as subclonal; these findings support the existence of genetic instability occurring at mutational and chromosome level during tumor development [90]. Considerable variation in the extent of intra-tumor heterogeneity was observed among various tumors [90]. LSQCCs carried significantly more clonal mutations than did lung adenocarcinomas; however, there were no significant differences between LSQCCs and lung adenocarcinomas in the number or proportion of subclonal mutations [90]. In lung adenocarcinoma patients, tumor stage positively correlated with the burden of clonal and subclonal mutations with the proportion of subclonal copy-number alterations; furthermore, in these patients, a significantly higher clonal and subclonal mutational burden was observed in smokers, compared to patients who had never smoked [90]. Driver mutations in EGFR, MET, BRAF and TP53 were almost always clonal; heterogeneous driver alterations occurring later during tumor evolution were observed in 76% of the tumors and were more common in PIK3CA and NF1 genes and in genes involved in chromatin modification and DNA damage response and repair. Genomic and chromosomal instability paralleled the mutational intra-tumoral heterogeneity and resulted in evolution of driver copy number alterations, such as amplifications in CDK4, FOXA1 and BCL11A [90]. Importantly, elevated copy number heterogeneity was associated with an increased risk of recurrence and death [90]. These findings indicate that intra-tumor heterogeneity originated through chromosome instability is associated with an increased risk of recurrence or death [90].

Interestingly, a recent study provided evidence that lung tumor evolution is characterized also by acquisition of immune evasion mechanisms [91]. This conclusion is based on the analysis of the acquisition of HLA loss of heterozygosity; HLA loss of heterozygosity occurs in about 40% of NSCLCs and is associated with a high tumor burden, APOBEC-mediated mutagenesis, upregulation of cytolytic activity, and PD-L1 positivity [91]. In both lung adenocarcinomas and LSQCCs, subclones harboring HLA loss of heterozygosity are associated with a clearly elevated burden of non-synonimous mutations and of neoantigens, compared to subclones derived from the same ancestral cancer cells, but without HLA loss of heterozygosity [91]. The peculiar nature of HLA loss-of-heterozygosity alterations, their subclonal development with high frequencies, considerable enrichment in metastatic sites, and occurrence as parallel events no related to particular genotypes, suggests that HLA loss-of heterozygosity represents an immune escape mechanism, occurring later during tumor evolution, in consequence of a strong environmental selection pressure [91]. The selection of a reduced HLA expression provides NSCLC cells with the capacity of evading immune mechanisms [91].

## 7. Genomic Alterations in Small Cell Lung Cancer

Small-cell lung cancer (SCLC) is a distinct clinical and histological entity within the range of lung cancers. It accounts for 13% of all newly diagnosed cases of lung cancer worldwide [92]. It typically occurs in heavy smokers. It is characterized by aggressive growth (patients usually present with rapid-onset symptoms due to local intra-thoracic tumor growth), frequent metastases and early death [92]. SCLCs are positive for various markers of neuroendocrine differentiation, such as chromogranin A, neuron-specific enolase, Neuron adhesion molecular (NCAM or CD56) and synaptophysin. However, the positivity of these markers alone cannot be used as a demarcation criterion to distinguish SCLCs from NSCLCs since 10% of NSCLCs are positive for neuroendocrine markers. Initial studies on the genetic somatic abnormalities observed in SCLC have shown a consistent number of abnormalities, none of them being specific for this tumor: (a) frequent inactivation of tumor suppressor genes, including *TP 53* (about 90% of cases), *RB1* (60−90% of cases) and *PTEN* (13% of cases); (b) the deletion of 3p (14–23) in the region containing the tumor suppressor gene *FHIT* is seen in virtually all SCLC cases; (c) copy number gain in 7p 22.3, which contains *MAD1L1*, a mitotic checkpoint gene; (d) *MYC* amplification, occurring in 20% of cases; (e) infrequent activating mutations at the level of *PI3KCA*, *EGFR* and *KRAS* (all < 10%).

The advent of the massive parallel sequencing technology allowed a more detailed analysis of the mutational range of SCLC. In this context, initial studies have been carried out on single SCLC cell lines (due to the difficulty to obtain tumor specimens since the large majority of these patients do not undergo surgical resection of the tumor). Thus, using this technology, Pleasance and coworkers have revealed in a SCLC cell line, 22,910 somatic mutations, of which 134 were in the exome and provided evidence of gene signature typical of tobacco exposure [93]. Tobacco smoking induces the deposit in the lungs of hundreds of chemical carcinogens including mutations through molecular processes that imply chemical modification of a purine residue, failure to repair the mutation by genome repair pathways and incorrect nucleotide incorporation opposite the distorted base during DNA replication. G > T transversions are the more common substitutions observed in cells exposed to polycyclic aromatic hydrocarbons present in tobacco smoke: in line with this finding, enrichment of G > T mutations at CpG dinucleotides was observed in SCLC cells [93].

Recently, the results of integrative genome analyses carried out on 63 primary tumors have been reported. A first important result of these analyses was that, compared to other tumors in global sequencing studies, SCLC exhibits an extremely high mutation rate corresponding to 7.4 protein-changing mutations per million base pairs [94]. This high mutation rate is seemingly related to tobacco carcinogens, as supported by the finding of an elevated rate of C:G > A:T transversions compared to the neutral mutation rate commonly observed [94]. The analysis of the mutated genes has led to identify a list of likely driver genes in SCLC: *TP53*, *RB1*, *PTEN*, *CREBBP*, *EP300*, *SLIT2*, *MLL*, *COBL* and *EPHA7* [94]. Inactivating *TP53* and *RB1* mutations play a major role in SCLC development, as suggested by the observation that lung tumors formed in *TP53* and *RB1* double knockout mice exhibit many features similar to those observed in humans in SCLC tumors [94]. Another group of recurrent mutations occurs at the level of three genes, *CREBBP*, *EP300* and *MLL* that encode histone modifiers: considering global frequency of the genomic alteration of these three histone-modifying enzymes it becomes evident that they represent the second most frequently mutated class of genes in SCLC. Another group of mutations concerns three tumor suppressor genes *PTEN*, *SLIT2* and *EPHA7* [94]. It is of interest to note that PTEN mutations and *FGFR1* amplifications are potentially tractable genomic alterations [94]. As above stated, *MYC* amplifications are frequent in SCLC. These amplifications involve several *MYC* family genes such as *MYC*, *MYCL1* and *MYCN* [95]. Interestingly, in a screening of drug sensitivity of SCLC it was observed that SCLC cell lines bearing *MYC* amplification, are sensitive to growth arrest and apoptosis induction by Aurora Kinase inhibitors [96]. On the basis of these observations, it was suggested that this subtype of SCLC patients could benefit by treatment with Aurora Kinase inhibitors, a class of drugs recently introduced in cancer therapy [96].

A recent study provided evidence about mutations of several members of the *SOX* family [97]. Knockdown of *Sox2* expression in SCLC with *Sox2* amplification resulted in inhibition of tumor growth [97]. In addition to this abnormality, it was also reported the recurrent *RFL-MYCL1* fusion: silencing of *MYCL1* in cell lines harboring this fusion gene resulted in inhibition of tumor growth [97].

As above indicated, all members of the *MYC* family (*MYC*, *MYCN* and *MYCL*) were found to be focally amplified and overexpressed in SCLCs. MYC acts by binding to a DNA motif (the E-box) under form of a heterodimer complex with MAX protein. *MAX* inactivating mutations have been observed in pheochromocytomas, including germline mutations associated with a hereditary form of this neoplastic disease. Given these observations, it was important to verify the possible occurrence of MYC inactivating mutations in SCLC, tumors with neuroendocrine features. Thus, Romero and coworkers through the analysis of 98 SCLCs have identified homozygous somatic mutations of *MAX* in 6% of cases; importantly, MAX mutations were found to be mutually exclusive with *MYC* family gene amplifications and with mutations of BRG1 (a gene encoding an ATPase of the *SW1*/*SNF* complex, a regulator of MAX and of MGA, a protein involved in MAX dimerization) [98]. Interestingly, these authors observed that BRG1 depletion specifically inhibits the growth of *MAX*-deficient SCLC cells [98].

George and coworkers have performed a comprehensive characterization of genomic alterations occurring in SCLC. Very high mutation rates, corresponding to 8.62 nonsynonomous mutations per Mb have been reported; C:G > A:T transversions were found in 28% of all mutations, a pattern suggestive of heavy smoking [99]. A reconstruction of subclonality architecture supported a threefold lower subclonal diversity in SCLC, compared to lung adenocarcinoma [99]. The results of this study confirmed that *TP53* and *RB1* were altered in almost all tumor with biallelic inactivation, sometimes related to complex genomic rearrangements; interestingly, two SCLCs with wild-type *RB1* showed evidence of chromotripsis, leading to overexpression of Cyclin D1, thus indicating an alternative mechanism of RB1 deregulation [99]. These findings strongly support the view that *TP53* and *RB1* genes inactivation is an obligatory event in SCLC development [99]. Among the significantly mutated genes in SCLC there are *KIAA1211* (17%), *COL22A1* (17%), *RGS7* (10%) and *FPR1* (6%), involved in G-protein-coupled signaling [99]. Inactivating mutations of NOTCH family genes are observed in 25% of human SCLCs: *NOTCH1* (14%), *NOTCH2* (4%), *NOTCH3* (6%) and *NOTCH4* (2%) [99]. In line with the idea that NOTCH signaling inactivation may contribute to SCLC development, activation of NOTCH signaling in a SCLC mouse model markedly reduced tumor number and prolonged animal survival; furthermore, in these models, NOTCH activation inhibited neuroendocrine gene expression [99]. Somatic genomic rearrangements of *TP73*, creating an oncogenic version of this gene, TP73∆ex 2/3 are observed in about 12% of SCLC patients [100]. In rare cases (*KIT* 6%) SCLC tumors displayed receptor kinase mutations [99]. Among the copy-number alterations, *TP53*, *RB1*, *CDKN2A* homozygous losses and *FHIT* losses, as well as *FGFR1*, *IRS2*, *MYC* family genes (*MYC*, *MYCN* and *MYCL1*) amplifications are frequent events [99].

Augert and coworkers have analyzed the mutation spectrum of a small population of SCLC patients and showed a high frequency rate of mutations of the histone methyltransferase *KMTD2*/*MLL2* (8% of SCLCs) [101]. In addition, mutations in other genes associated with transcriptional enhancer control, including *CREB* binding protein gene (CREBBP), E1A binding protein *p300* gene (EP300), and chromodomain helicase DNA binding protein 7 gene (*CHD7*) [101].

Dowlati and coworkers have analyzed the possible correspondence between molecular abnormalities and response ton therapy in 39 SCLC patients and observed that patients with mutant *RB1* (observed in 58% of patients) had better overall survival and progression-free survival compared with patients with wild-type *RB1* [102].

As mentioned above, SCLC is a particularly lethal cancer, with a median 9–10 months survival for metastatic disease and 2 years for nonmetastatic patients. The standard treatment for primary SCLC consists in a chemotherapy regimen based on a platinum doublet, cisplatin or carboplatin, usually in combination with the topoisomerase II inhibitor etoposide. The standard treatment of relapsing SCLC implies chemotherapy regimens involving a topoisomerase I inhibitor, Topotecan or Irinotecan. Primary SCLC is considerably sensitive to first-line treatment with >50% of objective responses; however, these responses are only transient with a progression-free survival <5 months; the response rates to second-line therapy are observed in only <20% of patients. The mechanisms responsible for the rapid development of chemoresistance in SCLC patients are largely unknown. The study of patient-derived xenografts to generate paired chemosensitive and chemoresistant SCLC, provided evidence that chemoresistance was associated with suppression of SLFN11, a factor involved in DNA-damage repair deficiency [103]. SLFN11 expression was decreased in SCLC cells of patients pre-treated with chemotherapy. Importantly, *EZH2* silencing in SCLC cells restores SLFN11 expression and chemosensitivity in vitro. Specific experiments provided evidence that EZH2 is directly involved in suppressing SLFN11 expression in SCLC. Very importantly, in PDX models, pharmacologic treatment with EZH2 inhibitors prevents the emergence of chemoresistance and improves chemoresponsivity [103].

Numerous recent studies have attempted to identify new therapeutic targets in SCLC. In 2015, Saunders and coworkers reported the results of an interesting study showing a high expression of the NOTCH inhibitory ligand Delta-like 3 (DLL3) in SCLC cell lines and primary tumors [104]. Interestingly, these authors have developed a DLL3-targetd antibody-drug conjugate capable of exerting a target-specific cytotoxic effect; this drug-conjugated antibody was able to exert a potent anti-tumor effect against neuroendocrine lung tumor xenografts [104]. A phase I study evaluated the response of 82 patients, including 74 SCLC and eight large-cell neuroendocrine carcinomas, to the anti-tumor effect of rovalpituzumab tesirine. At active doses of this drug, 18% of 60 assessable patients displayed a confirmed objective response; importantly, the rate of response to the treatment was 38% among patients displaying high DLL3 expression [105]. A phase III study enrolling patients with ongoing clinical benefit from 1st line platinum-based therapy will enroll in a large multinational trial more than 700 SCLC patients [106]. A recent study showed that at the immunohistochemical level 83% of SCLCs display DLL3 expression, with 32% of cases showing >50% of DLL3-positive cells (DLL3-high); the survival of DLL3-high and DLL3-low SCLCs was similar [107]. The control of the level of DLL3 expression on SCLC cells is of key importance in the context of ongoing phase III clinical studies [107]. Sharma and coworkers have developed ^89^Zr-labeled SC16 anti-DLL3 antibody as a companion diagnostic agent to facilitate selection of patients for treatment with Rova-T based on a noninvasive exploration of the in vivo status of DLL3 expression using PET imaging [108]. The introduction of this radiolabeled anti-DLL3 antibody could represent a precious tool for both selection of patients suitable for therapy with Rovalpituzumab teserine and for evaluating response to therapy [108].

Two studies suggest that Checkpoint Kinase 1 (CHK1) could represent a therapeutic target in SCLC cells. Thus, Sen and coworkers showed that the frequent loss of *TP53* and *RB1* in SCLCs results in loss of *E2F1* repression and is associated with increased expression of several mediators of DNA damage repair, including PARP1 and CHK1 protein. CHK1 is a serine threonine protein kinase that, in cells possessing aberrant *TP53*, becomes the main mediator of DNA damage-dependent cell-cycle arrest. Interestingly, targeting of CHK1 in SCLC cells with the specific inhibitor prexasertib increases the sensitivity of these cells to cisplatin and to PARP inhibitor olaparib [109]. Doerr and coworkers have performed a transcriptomic analysis of SCLC samples, providing evidence on the higher expression in these tumors of genes involved in cell cycle regulation, DNA damage signaling and DNA repair. One of the most striking finding concerns the high expression of CHK1. Importantly, ATR and CHK1 inhibitors displayed a selective toxicity for SCLC tumor cells, but not for NSCLC tumor cells [110]. These observations suggest that SCLC displays an actionable dependency on ATR/CHK1-mediated cell cycle checkpoints [110].

Preclinical studies have shown that the BCL-2/BCL-X_L_ inhibitor navitoclax is able to decrease the proliferation of SCLC cells; however, this promising preclinical activity failed to translate into a successful clinical activity in SCLC [111]. However, a recent study showed that only SCLCs bearing high BCL-2 are markedly inhibited by ventoclax, a BCL-2 inhibitor approved for clinical use [112]. This study suggested rationale for biomarker-guided clinical trials of ventoclax in high BCL-2-expresssing SCLCs [112].

Various studies have shown that the transcription factors ASCL1 and NEUROD1 play a key role in promoting the survival and malignant phenotype of SCLC. ASCL1 and NEUROD1 expression in SCLCs help to define tumor heterogeneity. These two transcription factors bind distinct genomic loci and regulate distinct genes: *ASCL1* targets oncogenic genes, including *MYCL1*, *RET*, *SOX2* and *NFIB; NEUROD1* targets *MYC* [113]. Importantly, ASCL1 regulates also many genes involved in NOTCH pathway, including DLL3 [113]. A recent study showed that MYC drives a neuroendocrine-low variant subset of SCLC with high NEUROD1 expression, corresponding to transcriptional profiles of SCLC characterized by low expression of neuroendocrine genes, including *ASCL1* [114]. MYC-driven SCLCs are sensitive to chemotherapy, but rapidly relapse; a drug-screening provided evidence that these tumors are uniquely sensitive to Aurora Kinase inhibitors, which dramatically improves chemotherapy response in vivo [114]. These observations are important because show criteria for patient stratification and reveal a potential targeted treatment approach for MYC-driven SCLCs [114].

ASCL1 is essential for both the proper development of neuroendocrine cells and is essential for the growth and survival of neuroendocrine lung cancers. Analysis of downstream targets of ASCL1 has revealed a number of potential molecular targets that represent molecular vulnerabilities that can be exploited for future therapeutic use [115]. One of this target is represented by the epithelial sodium channel, a membrane transporter that can be inhibited by orally effective potassium-sparing diuretics such as amiloride and its derivatives [116].

During normal lung development, NOTCH pathway activation inhibits the differentiation of lung progenitor cells to a neuroendocrine cell fate. NOTCH signaling results in a tumor suppressive effect. A recent study explored the effects of NOTCh signaling In SCLCs, showing that NOTCH signaling can be both tumor suppressive (intrinsically to neuroendocrine cells) or pro-tumorigenic (through the generation of non-neuroendocrine cells that are more chemoresistant and promote neuroendocrine tumor cell growth) in SCLC [117]. These observations provided a rationale for combining chemotherapy with NOTCH inhibition as a therapy for patients with SCLC, whose tumors display NOTCH-active tumor cells [117]. However, the results of a clinical trial with Tarextumab, an antibody inhibiting NOTCH 2/3, although initially showed a trend towards an improvement of progression-free survival in patients whose tumors expressed elevated levels of *NOTCH* target genes, failed to confirm this trend in in a larger cohort of patients in the context of a phase II study [117].

In addition to SCLCs the neuroendocrine tumors (NET) of the lung englobe also other, more rare, heterogeneous populations of tumors, including low-grade well-differentiated bronchial carcinoids (also known as typical carcinoids), intermediate grade atypical carcinoids and highly malignant large cells neuroendocrine carcinomas (LCNECs). However, recent molecular and clinical data indicate that these four neuroendocrine lung tumors can be grouped into two groups: high-grade neuroendocrine cancers, including SCLCs and LCNECs and low-grade carcinoids, involving typical and atypical carcinoids [118]. Thus, lung neuroendocrine tumors are subdivided into four different histological subtypes: Typical Carcinoid (TC), Atypical Carcinoid (AC), Large Cell Neuroendocrine Carcinoma (LCNEC) and Small-Cell Lung Carcinoma (SCLC). The pathological classification of these tumors is based on cytological criteria, positivity for neuroendocrine immunohistochemical markers, the mitotic activity and the presence of necrotic areas. From a clinical point of view, TCs are low-grade tumors associated with a good prognosis, ACs are intermediate-grade tumors and SCLCs and LCNECs are high-grade tumors, associated with a poor prognosis. The treatment of these tumors is related to their prognostic categorization, with TCs being treated with surgery alone, and ACs and LCNECs being treated with either surgery and/or systemic therapy and SCLCs being treated with systemic therapy. 

According to many biologic and molecular features carcinoid lung tumors (CLT) are distinguished from high-grade neuroendocrine carcinomas (HGNC): TP53 mutations are frequent in HGNCs, but are rare in CLTs; smokers are very frequent among HGNCs, but are rare in CLTs; 39 and 17p chromosome deletions are frequent in HGNs, but absent in CLTs, while 11q chromosome deletions are common to all neuroendocrine lung tumors; downregulation of E-cadherin expression, aberrations of the Rb pathways and abnormalities of the extrinsic and intrinsic apoptotic pathway are present in HGNECs, but absent in LCTs; the expression of the FHIT tumor suppressor is lost in HGNECs, but is maintained in LCTs [118]. A recent study provided a first detailed analysis of the somatic genetic abnormalities observed in LCTs. This analysis provided evidence that mutations in chromatin remodeling genes are frequent in these tumors [118]. Particularly, covalent histone modifiers are mutated in 40% of cases, while the SWI/SNF complex is mutated in 22% of cases [119]. Concerning the genes involved in chromatin remodeling processes, frequent are the mutations of *MEN1* and *ARID1A* genes [119]. The presence of only these mutations affecting few biochemical pathways and the virtual absence of other cancer-associated mutations argues in favor of an independent origin of these tumors from HGNECs [119]. Interestingly, gene expression signatures derived from the analysis of large numbers of lung adenocarcinoma patients indicate that about 10% of tumors with a neuroendocrine profile are present within tumors pathologically diagnosed as adenocarcinomas [120]. This subgroup of neuroendocrine carcinomas is characterized by expression of ASCL1 (Achaete-scute homolog 1 is a member of the bHLH transcription factors and is required for proper development of pulmonary neuroendocrine cells) and RET, is associated with a poor prognosis and occurs predominantly in smoker patients [121].

Asiedu and coworkers have performed a sequencing analysis of Atypical and Typical Carcinoids and have found recurrent mutations in several cancer genes, including *ATP1A2*, *CNNM1*, *MACF1*, *RAB38*, *NF1*, *RAD51C*, *TAF1L*, *EPHB2*, *POLR3B* and *AGFG1* [122]. These mutated genes are involved in important biological processes, such as cell division cycle, cellular metabolism, cell death, apoptosis, immune regulation [122]. The top most significantly mutated genes were *TMEM41B*, *DEFB127*, *WDYHV1*, and *TBPL1* [122]. Pathway analysis of significantly mutated and cancer driver genes involved MAPK/ERK and Amyloid Beta Precursor Protein (APP) pathways, whereas analysis of copy n umber alterations and gene expression data indicated a deregulation of the MAPK/ERK and NF-kB pathways [122]. The genetic alterations observed in ACs and TCs were similar [122].

**Figure 4:**
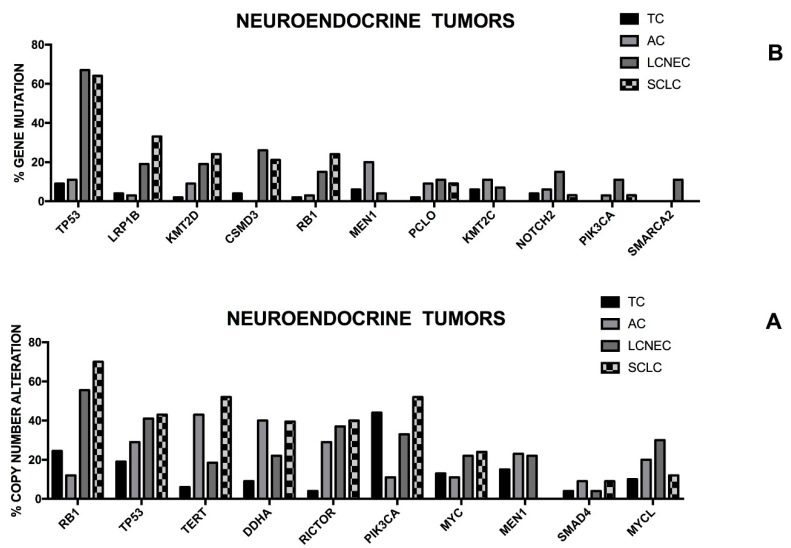
Genetic abnormalities observed in neuroendocrine lung cancers classified into four subtypes: TC (Typical Carcinoid); AC (Atypical Carcinoid); LCNEC (Large Cell Neuro Endocrine Carcinoma); SCLC (Small Cell Lung Carcinoma). A: Copy Number Alterations; B: Gene Mutations The data plotted in this figure were reported by Simbolo et al. [123].

More recent molecular studies were focused to better define the unique molecular characteristics of these four subtypes and to define the heterogeneity of some of these tumors. Thus, Simbolo and coworkers have recently reported a deep sequencing analysis of the four subtypes of lung neuroendocrine tumors, showing their peculiar molecular properties (Figure 4) [123]. Carcinomas had more mutations that carcinoids: TCs 0.8/Mb, ACs 1.6/Mb, LCNECs 3.0/Mb and SCLCs 2.9/Mb [123]. The most frequently mutated genes were: TP53 in carcinomas (TCs 9%, ACs 11%, LCNECs 67% and SCLCs 64%) and MEN1 in carcinoids (TCs 11% and ACs 20%) [123]. *KMT2D* displayed a trend on mutation rate in the four subtypes similar to that observed for TP53 (TCs 2%, ACs 9%, LCNECs 18% and SCLCs 24%) (Figure 4). It is interesting to note that chromatin-remodelling genes (*KMT2A*, *KMT2C*, *KMT2D*, *ARID1A*, *ARID1B*, *ARID2*) were similarly mutated in carcinoids (45.5%) and in carcinomas (55%) (Figure 4). In contrast, genes involved in cell-cycle control, such as *TP53*, *RB1* and *ATM*, were much more frequently mutated in carcinomas (70%) than in carcinoids (10%).

Finally, PI3K/AKT/mTOR pathway mutations were similarly higher in carcinomas (12%) than in carcinoids (2%) [123]. Copy number alterations were frequently observed at the level of the *RB1* and *TP53* genes, determining gene losses, more frequent in carcinomas than in carcinoids [123]. *TERT*, *SDHA* and *RICTOR*, as well as *PIK3CA* copy number gains were much more frequent in carcinomas than in carcinoids [123]. 

Other studies were focused to characterize the genetic alterations occurring in LCNECs. Miyoshi and coworkers have analyzed the genome profiling of 78 LCNECs by targeted capture sequencing of all the coding exons of 244 cancer-related genes and observed: frequent inactivating mutations in *TP53* (71%) and *RB1* (26%); genetic alterations in *PI3K*/*AKT*/*mTOR* pathway [15%, including *RICTOR* (5%), *PI3KCA* (3%), *PTEN* (4%), *AKT2* (4%) and *mTOR* (1%)]; activating mutations at the level of RTKs [*FGFR1* (5%), *KIT* (4%), *ERBB2* (4%), *EGFR* (1%)] and RAS pathways [*KRAS* (6%) and *HRAS* (1%)]. According to these findings it was concluded that LCNECs have a similar genomic profile to SCLC [124].

Next generation sequencing of 248 cancer-related genes in 48 LCNEC patients revealed a consistent heterogeneity and, according to genomic profiles, these tumors were subdivided into three subtypes, two major and one minor subsets: (a) a SCLC-like subset, characterized by *TP53* and *RB1* co-mutation/loss and other SCLC-type alterations, such as *MYCL* and *SOX2* amplification; (b) a NSCLC-like subtype, characterized by the lack of *TP53* and *RB1* co-alteration, *TP53* mutations and very frequent occurrence of NSCLC-type mutations, such as *STK11*, *KRAS* and *KEAP1*; (c) a rare carcinoid-like subtype, characterized by *MEN1* mutations and low mutational burden [125]. SCLC-like tumors were characterized by a high proliferation rate and the NSCLC-like subtype by adenocarcinoma-type differentiation marker expression [125]. In spite the similarities with lung adenocarcinoma, the NSCLC-like subtype exhibited peculiar genomic alterations, such as the frequent mutations in NOTCH family genes (28%) [125].

Chemotherapy treatment for LCNEC is matter of debate, due to its limited chemosensitivity. Usually these patients were treated either with a platinum-etoposide chemotherapy or with the same chemotherapy regimen used for lung adenocarcinoma. In a cohort of 79 LCNEC patients analyzed for their genomic profile by next generation sequencing, it was analyzed the impact of chemotherapy choice on disease outcome [126]. Patients with NSCLC-like subtype have better overall survival when treated with NSCLC chemotherapy, compared with SCLC chemotherapy (platinum-etoposide) [127]. This observation supports the clinical relevance of molecular profiling Of LCNECs [127].

George and coworkers have reported a detailed analysis of the genome profiling of LCNECs by whole exome sequencing and have also performed a transcriptional analysis of these tumors [128]. This analysis provided evidence about the existence of two molecular subgroups, similar to those described above. Type I LCNECs with bi-allelic *TP53* and *STK11*/*KEAP1* alterations; type II LCNECs with bi-allelic inactivation of *TP53* and *RB1* [128]. Despite sharing genomic alterations with lung adenocarcinomas and squamous cell carcinomas, type I LCNECs display a neuroendocrine profile, with similarities to SCLCs; type II LCNECs exhibit genetic similarities to SCLCs but are different from these tumors for the low level of neuroendocrine markers and high activity of the NOTCH pathway [128]. This study provided evidence that eight genes were recurrently mutated in LCNECs. *TP53* was the most frequently mutated gene, followed by RB1 (inactivating somatic events); bi-allelic alterations of these two genes co-occur in about 40% of cases and are the hallmark of type II LCNECs [128]. Interestingly, at pathological levels, tumors with RB1 alterations frequently display LCNECs with admixtures of other histological components. Somatic alterations occurring at the level of functionally important domains of *STK11* and *KEAP1* are observed in 30% and 22% of cases, respectively; importantly, these alterations are mutually exclusive with *RB1* alterations and represent the hallmark of type I LCNECs [128]. Mutations at the level of the genes encoding the metalloproteinases *ADAMTS2* (15%) and *ADAMTS12* (20%) and of the genes encoding *GAS7* (12%) and *NTM* (10%) are also frequent and occur at the level of both the LCNEC molecular subtypes. Interestingly, LCNECs harbor also alterations of oncogenes which are commonly found in lung adenocarcinomas, but usually absent in neuroendocrine tumors like SCLCs, such as *RAF* family genes, *BRAF* and *NFE2L2* mutations [128]. The analysis of transcriptomic profiling provided evidence about the existence of two main subtypes: type I LCNECs with high neuroendocrine expression and, similar to SCLC, a profile of ASCL1^high^/DLL3^high^/NOTCH^low^ (corresponding to the type I at the level of the genomic profile); type II LCNECs with reduced expression of neuroendocrine genes and a pattern of ASCL1^low^/DLL3^low^/NOTCH^high^ [128].

## 8. Normal Lung Stem Cells

Before analyzing the evidence provided until now in favor of the existence of lung cancer stem cells it is important to have an idea about normal lung stem cells. The lungs, together with trachea, arise from the anterior foregut endoderm, a tissue generating multiple organs in addition to the respiratory system. During the various stages of development, the endoderm and mesoderm components of the lung interact, promoting cell differentiation and progressive development of the lung through branching and patterning: these processes are regulated by multiple signaling pathways, including FGF, BMP and the Wnt pathways reviewed in [129]. The normal lungs have a complex anatomical and histological structure and are composed by a multitude of cell types. In the section of the respiratory system commonly called cartilagineous airways, the luminal epithelium recovering the trachea and the main bronchi is composed by terminally differentiated ciliated cells and Clara-like cells producing secretoglobins. In addition to these cell types, some neuroendocrine cells are also present. In addition to the surface epithelial cells there are also basal cells, characterized by the expression of cytokeratin 5 and 14, NGFR and p63. In the small bronchi and bronchioles, the surface epithelium becomes columnar and is composed by more Clara-like cells than ciliated cells. The most distal region of the lung is composed by the alveoli; two types of alveoli have been described, classified according to the cell type of epithelial cells: alveoli type I flat cells involved in gas exchanges and composed by this epithelial cells and alveoli type II composed by cuboidal cells, with cells containing secretory vesicles filled with lung surfactant.

Recent studies have shown an essential role for basal p63^+^ cells in airway development and in the generation of distinct stem cell pools in adult airways [130]. P63^+^ cells are abundant in the developing embryonic lung; p63^+^ cells arising from the lung primordium are initially multipotenmt progenitors of airways and alveolar lineages, but later become restricted to generate the tracheal stem cell pool. In the intrapulmonary airway, these cells are maintained in the adult life as immature cells in bronchi, serving as a are p63^+^KRT5^−^ progenitor cell pool that responds to influenza virus (HIN1)-induced injury [130]; particularly, a subpopulation of CC10 lineage-labeled p63^+^KRT5^−^ cell population is required for a full response to HN1 infection [130]. The p63^+^KRT5^−^ cell population is contained within the SOX2 lineage labeled pool previously identified and similarly involved in response to influenza virus [131].

Many studies carried out in mouse lungs have suggested the existence of putative stem cells, exhibiting the capacity to differentiate into the bronchiolar, alveolar type I and alveolar type II cells. Very few studies have been performed in the human system to try to identify and define lung stem cells. A putative population of human lung stem cells has been recently proposed by Kajtsura and coworkers, as being c-kit^+^ [132]. These cells were claimed to have a multi-tissutal differentiation potential, being endowed with the potential to generate lung epithelial cells, mesenchymal and endothelial tissues [132]. Particularly, these cells were shown to be capable of self-renewal in vitro, expressed the pluripotency markers OCT4, NANOG, SOX2, and KLF4, and to regenerate both the endodermal and mesodermal (vascular) components of the cryo-injured mouse lung [132]. However, this cell population was the object of a considerable controversy and its definition and existence remains uncertain.

There is now consistent evidence that distinct stem cell populations are responsible for the maintenance and reparation of these different lung epithelial regions. Lineage tracing experiments have allowed to define the identity of the stem cell populations responsible for the maintenance of lung epithelium: basal cells, Clara cells and ATII alveolar cells. Thus, at the level of the pseudostratified epithelium, basal cells as stem cells of the mouse trachea and human airway epithelium. Particularly, it was shown that both in mouse and human there is a basal cell population expressing p63 and cytokeratins 5 and 14, able to self-renew and generate luminal daughters in the sphere-forming assay [133]. In a subsequent study, it was provided evidence that NOTCH signaling is required for basal stem cell differentiation: in fact, NOTCH activity is low in airway epithelium under steady-state conditions and greatly increases during lung epithelial regeneration and enhanced luminal progenitor generation [134]. Loss of function experiments have shown that NOTCH activity is required for the differentiation, but not for the self-renewal of basal cells [134]. Clara cells are also able to self-renew and give rise to ciliate cells, but not to alveolar cells, and meet in part the stem cell criteria [135]. At the level of the alveolar epithelium, ATII cells act as stem cells through their capacity to both self-renew and generate ATI cells [136]. Desai and coworkers have recently proposed, and supported by a number of experimental evidences, that during the development the lung alveolar stem cells are bipotent progenitors expressing a subset of ATI and ATII markers and able to give rise to either ATI or ATII cells by shutting off inappropriate cell markers either in early or late stages of differentiation, then turning on cell-type-specific late markers, as they complete the maturation process [137]. The co-expression of ATI and AT II markers by these progenitors suggests that they have evolved from a primordial pneumocyte with features of both these cell types [137]. In contrast, after birth new ATI cells are originated from rare, self-renewing, mature ATII cells that produce clonal foci of alveolar renewal. This stem cell function of ATII cells is promoted by ATI injury. The self-renewal of ATII cells is induced in vitro by activation of the EGFR and in vivo by oncogenic KRas^G12D^, eliciting the formation of multifocal clonal adenomas [137]. According to these findings it was proposed a model of alveolar lung homeostasis and tumorigenesis, indicating that: during lung development, bipotent progenitors differentiate into ATI and ATII cells; after birth, mature ATII cells function as stem cells intermittently activated for alveolar renewal and repair by signals originated by dying ATI cells; activating mutations of EGFR or KRAS in ATII cells induce constitutive self-renewal of ATII cells, giving rise to the generation of tumors [137].

As above mentioned, the alveolar epithelium is maintained by rare type 2 (ATII) cells, cuboidal epithelial cells that retain the surfactant function of standard ATII cells, but also serve as stem cells. Their activation and differentiation give rise to the generation of ATI cells and generates clones of expanding renewal foci generating each year about 7% new alveoli [137]. It remains to understand how stem cells are selected from ATII cells, how their number is maintained and the mechanisms that control their symmetric or asymmetric cell divisions, underlying their self-renewal or differentiation to ATI cells. A recent study showed that WNT signaling is essential to maintain these cells (a WNT signaling in these cells is provided by autocrine mechanisms and by juxtacrine mechanisms mediated by fibroblasts present in the microenvironment); abrogating WNT signaling deplete these stem cells [138]. When daughter cells leave the WNT niche where they live, usually undergo a process of transdifferentiation into ATI cells [138]. Lung injury induced autocrine WNT signaling that promotes ATII progenitor cell expansion [138].

ATI and ATII cells receive signals from adjoining mesenchymal tissue, important for both tissue homeostasis and regeneration after injury. In this context, different epithelial-mesenchymal interactions, occur in the airway system, and particularly those intercurring between basal stem cells, secretory cells and mesenchymal cells present in these microenvironments. A recent study provided about a heterogeneity of lung epithelial mesenchymal lineages, providing evidence about a mesenchymal lineage (PDGFRα^+^) that participate in and provide preferential signals to promote alveolar self-renewal and regeneration and a mesenchymal lineage mediating signals that promote an ineffective injury response, such as myofibroblast expansion [139]. Another study supported the heterogeneity of lung mesenchymal cell populations in their phenotype and in their capacity to sustain the proliferation and differentiation of lung epithelial progenitors. Thus, using genetic lineage tracing, single-cell sequencing and organoid culture approaches, it was shown that LGR5 and LGR6, two well-known markers of stem cells in epithelial tissues, are markers of and help to define subpopulations of lung mesenchymal cells [140]. Particularly, LGR6 identifies a subpopulation of mesenchymal cells composed by smooth muscle cells surrounding airway epithelial and functionally involved in the promotion of airway differentiation of epithelial progenitors via WNT-FGF10 cooperation; genetic ablation of these mesenchymal cells impairs lung regeneration and airway repair after lung injury. LGR5^+^ cells are located at the level of alveolar microenvironment and are physiologically involved in the promotion of alveolar differentiation of epithelial progenitors through WNT pathway activation [140].

Various models of lung injury have shown that following an injury of the proximal airway epithelium basal stem cells are the main stem cell population (in part with Clara cells) contributing to the reparation of the damaged pseudostratified epithelium, while following an injury of the distal alveolar epithelium, ATII cells are the main stem cell population contributing to the reparation of the damaged alveolar epithelium [141]. The behavior of lung epithelial stem cells is regulated by both intrinsic and extrinsic mechanisms; the extrinsic mechanisms are provided by the microenvironment, represented by the tissutal stem cell niches generating signals mediated by the secretion of growth factors or by the interaction of stem cells with components of the extracellular matrix or with other cell types. Although the signals originated at the level of the epithelial stem cell niches involve complex regulatory networks, among the various growth factors secreted at the level of the microenvironment, a major role is played by FGF10 which gives signals allowing the maintenance of the distal airway stem cells and drives the differentiation of proximal airway basal stem cells reviewed in [141]. FGF10 contributes to the development of some main constituents of the lung stem cell epithelial niches, such as airway smooth muscle cells and lipofibroblasts [142]: in fact, FGF10-positive cells act as progenitors of these cells during development [142].

Recently, a new population of putative lung stem cells was isolated from human lung. These cells were identified according their positivity for E-Cad and LGR6: E-Cad^+^/LGR6^+^ cells can be indefinitely expanded from human lungs, harbouring both self-renewal capacity and the potency to differentiate in vitro and in vivo [143]. Importantly, E-Cad^+^/LGR6^+^ cells when inoculated into the kidney capsule produce a progeny of differentiated bronchoalveolar tissue, while retaining their self-renewal capacity [143].

Two studies on a murine model of lung damage and subsequent regeneration allowed to identify a population of distal lung alveolar stem cells [144,145]. Both these studies showed that a rare population of distal alveolar stem cells (DASCs), lineage-negative, are responsible and indispensable for lung regeneration following influenza virus or bleomycin-mediated lung damage [144,145]. Particularly, it was shown that a population of DASCs, expressing Trp63 and Keratin5 undergo a proliferative expansion following influenza virus infection and assemble into nascent alveoli at the level of the sites of interstitial lung inflammation [144,145]. Genetic ablation of these cells impairs lung regeneration. Cell tracking experiments provided evidence that these cells are individually able to differentiate, generating a progeny of alveolar type I and II cells and bronchial secretory cells [144]. The injection of these cells minimizes in vivo the consequences of endogenous lung stem cell loss [144,145].

The studies carried out in these last years have supported the existence of facultative progenitors that are recruited after injury to contribute to the process of lung regeneration. In this context, a recent study reported the isolation and characterization of a WNT-responsive alveolar epithelial progenitor (AEP) lineage within the ATII cell population, acting as a major facultative progenitor cell in the distal lung [146]. AEPs exhibit a distinct trascriptomic profiling and respond to WNT and FGF stimulation. Importantly, human AEP cells can be identified and isolated through the expression of the membrane marker TM4SF1 and act as alveolar epithelial progenitor cells in organoid cultures [146]. The comparison of these AEP^+^ progenitors with KRT5^+^ progenitors suggest a different role after lung injury: AEPs could represent a major lineage contributing to functional alveolar regeneration though the production of ATI and ATII cells after lung injury; KRT5^+^ progenitors act rapidly after lung injury to prevent loss of epithelial barrier [146]. Thus, these two progenitors should cooperate in promoting tissue regeneration after lung injury. 

The large majority of the studies on tissutal niches have been carried out in mice and few studies have been performed in humans. However, a recent study characterized the human alveolar epithelial stem cell niche organized to permit the survival and self-renewal of LGR6^+^ alveolar epithelial stem cells [147]. This important function is mediated by a paracrine regulatory circuitry involving SDF-1alpha secreted at low levels by LGR6^+^ stem cells and acting by promoting the recruitment of fibroblasts which in turn release TNF-alpha [147]; this TNF-alpha leads to the activation of a TGF-beta/p38-alpha-mediated autocrine loop at the level of LGR6^+^ epithelial stem cells, inducing an increase in the SDF-1alpha production by these cells [147]. The high SDF-1alpha released then induces the release of angiogenic growth factors by fibroblasts, promoting tissutal angiogenesis [147].

Given the peculiar physiological function of lung it is not surprising that reactive oxygen species (ROS) may act as important regulators of lung stem cell homeostasis, proliferation and self-renewal. In fact, it was shown in a recent study that not the absolute low or high ROS level in a lung stem cell, but rather the dynamic intracellular change from a low ROS to a relatively high ROS within a lung stem cell is a key determinant of cell self-renewal after injury [148]. The increase of ROS levels activates the transcription factor Nrf2, which in turn induces the activation of the NOTCH pathway to stimulate lung stem cell self-renewal after injury and an antioxidant molecular program able to scavenger intracellular ROS and thus able to protect from the toxic effects of ROS and to restore low ROS levels [148].

A defective function of the reparative functions of lung stem cells may have important consequences in the development of some human pathologies. Thus, recent studies suggest that alveolar stem cell senescence could represent a major driver of pulmonary pathologies, such as idiopathic pulmonary fibrosis and emphysema. In fact, recent studies have shown that mutations in telomerase (TERT and TR) represent the most commonly observed risk factor predisposing to the development of idiopathic pulmonary fibrosis; furthermore, telomerase mutations predispose to emphysema development in smoker patients, as well as alpha-1 antitrypsin deficiency [149]. Telomeres are DNA-protein structures that exert the important biological function of protecting chromosome ends; these structures shorten progressively with cell divisions and with aging. A short telomere originates a signal of DNA damage to the cell, determining a consequent induction of cellular senescence and apoptosis. This effect of telomerase shortening was observed at the level of alveolar stem cells, in mice, resulting in a defective repopulating and reparing capacity of these cells. In fact, in mice short telomeres result in a defective function of type 2 alveolar epithelial cells, due to their premature senescence and to the induction of a reactive inflammatory response [150]. These observations are important because suggest that alveolar stem cell failure is the main driver of telomere-mediated lung disease and efforts must be made at therapeutic level to try to reverse these negative effects on stem cell function [150]. 

As above mentioned, the identification of human lung stem cells remains at a large extent elusive. Particularly, whether there are lung cells with regenerative capacity in vivo remains to be demonstrated. A recent study by Ma and coworkers provided a first evidence that human SOX9^+^ airway basal cells could act as regenerative stem cells [151]. The entire lung epithelium arises from SOX9-positive progenitors that for the respiratory tree and differentiate into airway and alveolar cells. A recent study showed that more SOX9^+^ multipotent progenitors can be isolated and expanded in long-term cultures; these cultured SOX9^+^ progenitors generate both in vitro and in vivo both airway and alveolar cell types [152]. According to these findings, it was suggested that a single expandable SOX9^+^ progenitor cell population could be used as an alternative to region-restricted stem cells [152]. Ma and coworkers have identified putative adult human lung progenitor cells located at airway epithelium rugae with a SOX9 marker to distinguish them from other SOX9^−^/p63^+^/KTR5^+^ airway basal cells [151]. Human SOX9^+^ cells can be isolated by bronchoscopic brushing and indefinitely expanded in feeder-free culture conditions, expanded human SOX9^+^ cells are able to generate both alveolar and bronchiolar epithelium and, after transplantation into injured mouse lung allowed the reconstitution of air-blood exchange system and improved lung function [151,152]. Taking advantage on these observations, for the first time the transplantation of in vitro expanded autologous SOX9^+^ cells were evaluated in two bronchiectasis patients; lung tissue repair and pulmonary function enhancement was observed 3-12 months after cell transplantation [151]. These observations may represent the basis for future large-scale clinical study aiming to demonstrate the lung regenerative potential of expanded SOX9^+^ progenitors [151].

In conclusion, the studies on normal lung stem cells, mostly based on studies carried out in mice, support the view that the regeneration of the respiratory epithelium is ensured by the proliferation and differentiation of region-specific epithelial stem and progenitor cells resident at the level of specific tissutal niches, located in various areas of the airway tree. The analysis of various lung injury models in mice and some pathological conditions in humans, supports physiological role for these stem/progenitor cells in lung tissue regeneration.

## 9. Lung Cancer Stem Cells

Studies carried out during the last years have attempted to isolate and to characterize cells endowed with the property to initiate and to maintain lung cancers. In lung cancers, three different cancer stem cell populations have been identified according to the expression of some selected markers (CD133) [153,154] or high expression of the ALDH isozymes [155,156] or the capacity to extrude cytotoxic drugs (drug-resistant side-population) [157,158].

Various studies have characterized CD133^+^ cells in NSCLCs. In this context, Eramo and coworkers have shown that CD133 was expressed in a variable, but small percentage of NSCLCs, usually restricted to <1% of cells [153]. CD133^+^ cells were able in about 30% of cases to form tumor spheres in vitro when grown in serum-free medium with EGF and bFGF; CD133^+^ cells derived from tumor spheres are able to generate tumors when inoculated into immunodeficient mice with histological features similar to those of the original tumor [153]. Bertolini and coworkers confirmed the low expression of CD133^+^ cells in NSCLCs and showed that the frequency of these cells is higher in cancer than in normal lung tissue; these CD133^+^ cells were shown to be tumorigenic and to express several stemness genes [159]. Importantly, the frequency of CD133^+^ cells increased after in vitro and in vivo treatment of lung cancer cells with cisplatin [159]. These observations suggest that lung cancer CD133^+^ cells are chemoresistant. Drug resistance of CD133^+^ lung cancer cells was confirmed in another study, showing that drug-resistant cells derived from NSCLC cell lines were enriched for CD133^+^ cells and displayed elevated cytokine expression [154]. A recent study further explored the cisplatin resistance of lung cancer cells. Thus, Liu and coworkers showed that lung cancer cells surviving to cisplatin treatment are enriched in CD133^+^ cells, displaying multiple drug resistance mainly related to expression of the drug transporter ABCG2 [160]. Additional experiments have provided evidence that NOTCH1 activation induced by cisplatin was required for cisplatin-mediated CD133 enrichment and induction of multidrug resistance [160]. Analysis of tumor specimens of patients undergoing cisplatin-based chemotherapy showed a considerable enrichment of CD133^+^ cells following drug treatment and evidenced NOTCH1 activation into these cells [160]. Finally, a recent study suggested a possible link between the level of CD133 expression in NSCLCs and the phenomenon of tumor vasculogenic mimicry, a phenomenon in which aggressive tumor cells mimic endothelial cells and form vascular channel-like structures to convey blood without the participation of endothelial cells [161]. However, a great variability in the detection and abundance of CD133^+^ cells in NSCLCs and in their tumorigenicity was observed in the various reports and some doubts were raised about the prognostic value of the level of CD133 expression in NSCLC [162].

Several studies have characterized the expression of aldehyde dehydrogenase enzymes (ALDH) in lung cancers and have analyzed the tumorigenic potential of cells overexpressing ALDH. In this context, initial studies have shown that the ALDH isozymes, ALDH1A1 and ALDH3A1, are expressed in putative epithelial stem cell niches, overexpressed in NSCLCs (both adenocarcinomas and squamous cell carcinomas) compared to normal lung tissue [163]. Jiang and coworkers observed that ALDH activity can be used as a marker to isolate cancer stem cells in two NSCLC cell lines and showed also that ALDH1 levels in primary tumors are associated with poor survival in a group of stage 1 patients [164]. These observations were supported by Sullivan and coworkers. These authors have shown through flow cytometric analysis of lung cancer cell lines and primary tumor cells that most of NSCLCs contain a subpopulation of cells with high ALDH activity, mainly related to the ALDH1A isozyme [156]. ALDH^+^ lung cancer cells were shown to be tumorigenic and possess clonogenic activity [156]. Expression analysis of sorted cell populations showed elevated NOTCH transcript expression in ALDH^+^ cells: inhibition of NOTCH activity using a gamma-secretase inhibitor greatly increased ALDH^+^ cells in lung cancers and concomitantly reduced the proliferation and clonogenicity of tumors [156]. In a recent study, Shao and coworkers have explored the various genes preferentially expressed in lung ALDH^+^ cells, compared to the corresponding ALDH^−^ cells: the gene most expressed in ADH^+^ cells wasALDH1A3. Knockdown of ALH1A3 into NSCLCs resulted in a dramatic decrease of ALDH activity, clonogenicity and tumorigenicity [165]. In this study, it was also observed that ALDH^+^ cells preferentially express STAT3, whose inhibition resulted in a decrease of ALDH^+^ cells and tumor clonogenicity [165]. Surprisingly, the inspection of ALDH1A3 levels into primary lung tumors preferential expression in well-differentiated adenocarcinomas, in newer-smokers and females [165]. 

As above mentioned, NOTCH signaling was required for the self-renewal of airway basal stem cells. The role of NOTCH signaling for maintaining lung cancer stem cells was supported by several recent studies. Thus, Hassan and coworkers have explored the effect of NOTCH overexpression on the development of cancer stem cell properties by lung cancer cells. Transduction with NOTCH gene constructs resulted in increased tumorigenicity both in vitro (tumor-sphere formation in serum-free medium) and in vivo (xenotransplantation in nude mice) [166]. NOTCH-overexpressing cells were resistant to chemotherapy and, in line with this observation, a statistically significant correlation between poor clinical outcome and NOTCH activity was observed in patients with lung adenocarcinoma [166]. This last observation was further supported by the finding that high expression of HES-1, a NOTCH target gene, in lung adenocarcinoma patients was associated with poor prognosis [166]. A rare population of CD24^+^/ITGB4^+^/NOTCH^high^ cells was shown to be capable of propagating tumors in both clonogenic and orthoptic serial transplantation assays [167]. This population is enriched after chemotherapy. All four NOTCH receptors mark this cell population, and, among them, NOTCH3 plays a nonredundant role in tumor propagation [167]. Growing evidence indicates that NOTCH signaling, in cooperation with several transcription factors, consistently contributes to the initiation and progression of NSCLC and to the epithelial-mesenchymal transition [168]. Zhang and coworkers have isolated a subpopulation of cancer stem-like cells (CD166^+^CD49f^high^CD104^−^Lin^−^) from primary NSCLC specimens and have shown that these cells express much more NOTCH1 than the CD166^−^ counterpart [169]. NOTCH1 signaling is required for the self-renewal of these cells via the transcription factor HES1 and for platinum resistance [169]. 

The Hoechst 33342 dye efflux assay and flow cytometry were used in many studies to isolate and characterize the side population (SP) cells from human lung cancer cells. SP cells isolated from NSCLC cell lines exhibit higher expression of ABC transporters and were resistant to chemotherapeutic drugs [157]. Furthermore, SP cells isolated from NSCLCs were more metastatic in xenograft models and their invasive properties are driven by high Rac1 activity [158].

Additional studies based on the isolation of SP cells from lung cancer cell lines have shown that these cells preferentially express SMO, a key mediator of the Hedgehog (HH) signaling pathways: blockade of the HH pathway using cyclopamine inhibited cell-cycle progression of SP cells [170]. Other studies have explored the expression of stemness transcription factors in lung cancer SP cells. Among the transcription factors, SOX2 seems to play a relevant role in lung cancer stem cells; in fact, high SOX2 levels are required to sustain self-renewal and expansion of lung cancer stem cells [171]. EGFR-Src-AKT signaling is required to maintain high Sox2 levels in lung cancer stem cells [171]. Other studies have addressed the functional role of SOX2 in the biological properties of SP LC cells. Through silencing and overexpression of SOX2 in lung cancer cells it was provided evidence that SOX2 expression is required for the maintenance of stemness and tumorigenic activity of human lung cancer SP cells [172]. As above reported, the amplification of chromosome 3q26 results in the co-amplification and co-expression of protein kinase C iota (PKCι) and SOX2 in LSQCC which cooperate to trigger and maintain cell-autonomous HH signaling in LSQCC cancer stem cells [172].

In a recent study, Akunuru and coworkers have compared the lung cancer stem cells identified according to these three different markers and reached the conclusion that SPO cells represent a peculiar subpopulation different from other cancer stem cells populations identified according to CD133 positivity or to the high ALDH expression [173]. On the basis of the results obtained on cancer cell lines and on primary tumors these three different cancer stem cell subpopulations were demonstrated to be not overlapping [173]. SP^+^ cancer stem cells were characterized by high expression of the transcription factors Sox2, Nanog and Oct4, high metastasizing capacity and high production of some cytokines, including IL-6 and VEGFA [173]. Importantly, they have shown that, under appropriate cell culture conditions, SP^−^ cells are able to generate SP^+^ cells: therefore, the non-cancer stem cell and the cancer stem cell populations were interconvertible [173].

More recently, Zhang and coworkers reported that the selection of CD166^+^ cells from primary NSCLC, pertaining to adenocarcinomas or squamous cell carcinomas or luminal cell cancers, gives rise to an enrichment of about 100-fold in cells endowed with tumor-initiating capacities, while CD166^−^ cells were unable to generate lung tumors when inoculated into immunodeficient mice [174]. Furthermore, only CD166^+^, but not CD166^−^ cells, were able to generate tumorspheres in vitro [174]. The comparative analysis of the transcriptome into the CD166^+^ and CD166^−^ tumor cell populations showed the existence of some cancer stem cell-associated genes, such as the oncogenic stem cell factor LIN28B and the embryonic lung transcription factors PEA3 and NPAS1; this analysis showed also that the glycine/serine metabolism enzymes are highly enriched in CD166^+^ cells [174]. Among the glycine metabolism enzymes, glycine decarboxylase (GLDC), a key component of the glycine cleavage system, was highly expressed in CD166^+^ cells [174]. Functional experiments have shown that GLDC is essential to lung cancer stem cells: particularly, both GLDC and LIN28B are required for cancer stem cell growth and tumorigenesis; GLDC overexpression was able to promote cell proliferation and transformation [174]. At the level of the primary tumors, lung cancers expressing high GLDC expression are associated to a reduced survival [174]. However, experiments of CD166 knockdown in patient-derived cell lines failed to show a significant effect on tumor growth. Furthermore, immunohistochemical results in 143 NSCLC patients failed to show any significant association between CD166 expression and patients’ survival [174]. According to these findings, Zhang and coworkers concluded that CD166 is an inert membrane marker of NSCLC stem cells [174]. The immunochemistry study of a large pane of NCSLC patients showed that CD166 expression was inversely associated with tumor size and lymph node status and was not correlated with patient survival [175]. According to these findings, the theory of CD166 as a CSC marker for NSCLC was questioned [175]. GLDC expression in NSCLC is a prognostic factor. In fact, high GLDH expression in NSCLC was associated with a negative prognosis. In contrast, low GLDC expression in combination with absent HIF-1α expression was associated with a good prognosis [176].

Other studies have provided some evidence that the membrane receptor glycoprotein, CD44, able to bind hyaluronic acid, could represent a marker of lung cancer initiating cells. In this context, Leung et al. using ten human NSCLC cell lines have shown that the CD44^+^ cell fraction possesses enhanced cancer stem cell properties, compared to CD44^−^ cells, including in vitro tumor-spheroid forming capacity. Furthermore, CD44^+^ cells isolated from some of these cell lines display increased cisplatin resistance; finally, in vivo tumors isolated from lung tumors generate from inoculation of CD44^+^ cells into immunodeficient mice exhibit expression of pluripotency/stemness-associated genes, such as OCT4, NANOG and Sox2 [177]. However, it is important to underline that CD44 expression was observed only in 60% of the cell lines examined by these authors [178]. The possible link between CD44 expression by lung cancer cells and chemoresistance was further reinforced by a recent study showing enhanced CD44 and CD133 expression on cisplatin-resistant lung cancer cells [178]. These cells displayed enhanced expression of other stem cell-related markers/functional properties [178].

Other studies suggest that c-kit, also known as CD117, the receptor of Stem Cell Factor (SCF) is a potential membrane marker of lung cancer stem cells. Levina and coworkers showed that tumor spheroids grown from lung cancer cell lines exhibit a markedly increased c-kit expression, compared to the expression observed in parental cell lines [179]. Interestingly, tumor spheroids exhibited also the property to produce and release SCF: this cytokine triggers an autocrine growth stimulation pathway [179]. Imatinib, a tyrosine kinase inhibitor, able to inhibit various receptor tyrosine kinases, including c-kit, inhibits tumor growth [179]. In line with these observations, a recent study reported an increased CD117 expression occurring in 22% of NSCLC [180]. In these tumors, a part of tumor cells co-express other tumor stem cell markers, such as CD133 and CD44. Importantly, the growth and survival of c-kit^+^ lung tumor cells are inhibited by the tyrosine kinase inhibitor imatinib [180].

As above mentioned, drug-selected lung cancer stem cells show high expression of CXCR4, in addition to CD133. A recent study addressed the possible functional role of CXCR4 in the maintenance of stemness of lung cancer stem cells. Experiments carried out on cancer stem cells purified from lung cancer cell lines have suggested that CXCR4 expression on these cells is required for self-renewal activity, resistance to ionizing radiations and for tumorigenicity in vivo [181]. Mancini and coworkers have shown sphere growth in 11 out 15 lung carcinoma malignant pleural effusion patient samples [182]. Compared to the control, fresh adherent cells, the tumor spheroids exhibited enhanced ALDH1 expression, together with enhanced expression of Stem Cell-associated markers [182].

The study of lung cancer stem cells was extended also to metastatic sites of this tumor. One of the most frequent metastatic sites of this tumor is represented by the brain. Thus, Nolte et al. have grown patient-derived brain metastases in serum-free stem-enriching tumorsphere conditions [183]. They observed that metastases-derived tumorspheres had a mean sphere-forming capacity and a median stem cell frequency comparable to that observed for primary lung cancer-derived tumorspheres [183]. Metastasis tumorspheres resembled for their phenotype and tumor-forming capacity in xenotransplantation assays to the primary tumors from which they were derived [183]. Attempts to identify a subpopulation of TICs within the brain metastases-derived tumorspheres were unsuccessful; however, some genes particularly overexpressed and associated to poor prognosis have been observed at the level of brain metastases tumorspheres [183].

Studies on tumorspheres isolated from lung cancer cell lines suggest that Musashi 1 (Msi1) is a potential marker of lung CDSCs. Msi1 is an RNA-binding protein involved in a series of processes related to the post-transcriptional regulation of gene expression. An important target of Msi1 is Numb, a negative regulator of NOTCH, whose translation is repressed by Msi1. Msi1 is well expressed in lung cancer cells and, particularly, at the level of tumorspheres isolated from lung cancer cell lines [184]. Inhibition of Msi1 expression by shRNA reduced spheroid proliferation [184]. Msi1 was shown to be expressed at elevated levels in both lung adenocarcinomas and LSQCCs [184].

As above mentioned, 80% of lung cancers can be attributed to cigarette smoking. Currently, it is estimated that more than 1.3 billion people smoke and, therefore, it is not surprising that the global burden of tobacco-associated thoracic malignancies (lung and esophageal cancers) will continue to increase, with very devastating consequences, particularly at the level of developing countries. Recent studies suggest that in addition to its numerous oncogenetic effects, tobacco smoking may have also several deleterious effects at the level of the cancer stem cell population. Thus, Ooi and coworkers have provided evidence that cytokeratin 14^+^ (K14^+^) lung cells represent a reparative cell population involved in tissue repair after injury, such as ischemic hypoxic injury, naphthalene injections (naphthalene is one of the carcinogens produced by cigarette smoking). Persistent of K14^+^ cells are observed in aberrant repair of premalignant repair and, particularly, at the level of NSCLCs associated with injury from smoking [185]. In fact, tobacco smoking causes continuous and repeated cycles of injury and repair of the airway. A subset of NSCLC patients associated with tobacco smoking display a clearly increased expression of K14^+^ cells and the presence of these K14^+^ progenitors in NSCLC predicted a poor prognosis and this predictive value was very strong in smokers [185]. According to these findings it was suggested that K14^+^ cells may represent tumor-initiating cells in a subset of smokers with NSCLC [185]. In another study, it was evaluated the effect of cigarette smoke condensate on drug resistance and on the SP population of lung cancer cell lines [185]. Cigarette smoke condensate enhanced drug resistance of lung cancer cells, a phenomenon mainly related to enhanced expression of the drug exporter ABCG2 [186]. In parallel, it was shown that cigarette smoke condensate induced a consistent increase of the number of SP cancer stem cell-like cells [186]. According to these findings it was concluded that cigarette smoking promotes lung cancer cell chemoresistance, through ABCG2 upregulation mediated by AKY activation, and expands the cancer stem-like cell population, further contributing to drug resistance [186]. Other studies in this area were focused to better define the mechanism through which cigarette smoking, in addition to be involved in cancerogenesis, diminishes responses to chemotherapy and radiation therapy, enhances the metastatic potential and decreases the survival of lung cancer patients. These effects are in part related to some effects of tobacco smoking at the level of the modulation of genes involved in stem cell function. Thus, it was reported that under clinically relevant exposure conditions, tobacco smoking causes a tumorigenic effect mediated through polycomb-induced repression of the protein Dickkopf1 (DKK1) which encodes a Wnt antagonist, thus resulting in an activation of Wnt signaling [187]. In a more recent study the same authors showed that tobacco smoking-induced epigenetic downregulation of miR487b results in overexpression of various polycomb group proteins, including BMI1 and SUZ12, as well as Wnt5a, KRas, c-Myc, all involved in the modulation of stem cell properties [187]. Finally, in another recent study the same authors have explored the molecular mechanisms through which cigarette smoking stimulates ABCG2 expression in lung cancer cells. Thus, they demonstrated that ABCG2 upregulation by cigarette smoke condensate was dependent upon ABCG2 gene promoter occupancy by aryl hydrocarbon receptor, Sp1 and NRF2 [188]. Mithramycin treatment inhibited cigarette smoke-induced ABCG2 upregulation and inhibited multiple stem cell-related pathways both in vitro and in vivo [188]. Finally, other recent studies have shown that cigarette smoke favors the induction of the epithelial to mesenchymal transition (EMT). Particularly, it was shown that cigarette smoking induces the repression of E-Cadherin, a hallmark of EMT, by regulating transcription factors Lef-1 and Slug, which leads to EMT [189]. In line with these observations, E-Cadherin levels in lung cancers of smokers are significantly lower than those observed in lung tumors of never-smokers [189]. Interestingly, histone deacetylase inhibitors are able to reverse cigarette smoking-induced migration and invasion through restoration of E-Cadherin expression [189].

Some studies have explored the possible link between cancer stem cells and EMT in NSCLC. In this context, Kumar and coworkers have developed a three-dimensional culture system in which NSCLC spheroid cultures were co-stimulated with TNF-alpha and TGF-beta: these cultures displayed elevated expression of EMT master-switch transcription factors TWIST1, SNAIL1, SLUG and SIP1 and high tumor invasivity [190]. These “mesenchymal” tumorspheres displayed elevated expression of stem cell markers, thus suggesting that EMT sustains the development of cancer-initiating cells [190]. In a subsequent study the same authors have shown that EMT induction promotes the secretion of soluble factors by NSCLC acting in an autocrine way and promoting NF-kB activity, which in turn upregulates INHBA/Activin, a morphogen of the TGF-beta superfamily, responsible for the maintenance of the EMY phenotype and for the stimulation of cancer-initiating cell self-renewal [191]. The responsiveness of NSCLC cells to TGF-beta is related to the stage of tumor progression. In fact, a peculiar molecular mechanism operating during NSCLC progression upregulates TGF-betaR3 expression: this mechanism is driven by overexpression of HMGA2, a non-histone chromosomal high-mobility group protein; the high expression of HMGA2 mRNA determines a marked binding of let-7 family micro-RNA; this blocking of let-7 micro-RNA determines a derepression of typical let-7 targets and particularly of TGF-betaR3, that, through this mechanism, is derepressed and overexpressed and TGF-beta signaling is greatly potentiated in NSCLC [192].

Studies carried out on murine lung cancer stem cells have led to identify Matrix Metalloproteinase 10 (Mmp 10) as a gene highly expressed in these cells and playing an important role in tumor initiation and maintenance. Mmp 10 is highly expressed in NSCLC tumors, but not in surrounding tumor stromal cells. Mmp10 is required for the growth of human lung cancer cells and for their inversion properties in vitro and in vivo [193]. In mouse lung cancer models Mmp10 was shown to be induced in bronchio-alveolar stem cells transformed by oncogenic KRas mutant [194] and to promote KRas-mediated bronchio-alveolar stem cell expansion and lung cancer formation [195]. According to these observations it was suggested a direct role of Mmp10 in the maintenance of lung cancer stem cells. This hypothesis was directly supported by a recent study showing that Mmp10 was highly expressed in murine oncosphere cultures enriched in CSCs and its knockdown by siRNA markedly inhibited oncosphere expansion and expression of stem cell markers [196].

The possible prognostic impact of CSC markers CD133 and ALDH1 was explored. As reviewed by Alamgeer et al., the prognostic significance of CD133 expression in NSCLC is uncertain, with highly contradictory evidences originated by various reports reviewed in [197]. In contrast, the majority of studies involving the detection of ALDH1 in lung cancer tissues have shown a negative impact of the level of this enzyme on patients’ outcome [198,199,200]. Only one report, in which tumor ALDH1 levels were measured by immunofluorescence and not by immunochemistry (with subdivision of patients into positive and negative groups) provided an opposite evidence [201]. A panel of putative stem cell makers was studies in a large group of different NSCLCs at histopathological level, showing a different pattern of positivity in three groups of tumors: CD44, ABC45, ALDH1 and Nestin were associated with poorly differentiated tumors; ALDH1, CD44 and SOX2 were frequently expressed in LSQCC; CD24, CD126 and epithelial cell adhesion molecule markers were expressed in adenocarcinomas [202].

Few studies have tried to characterize cancer stem cells in adenosquamous carcinomas. These tumors contain a mixture of squamous (cytokeratin 5^+^) and adenocarcinoma (cytokeratin 7^+^) features. The origin of this mixture of cells is unclear since squamous and adenocarcinoma cells seem to originate from the malignant transformation of different cell types. To try to better understand the cellular origin of adenosquamous tumors, Mather and coworkers have first grown in vitro adenosquamous tumor cells in cell culture medium used to grow human lung cells: this cell culture procedure allowed to enrich for cancer stem/progenitor cells able to engraft immunodeficient mice, generating adenosquamous tumors [203]. Important, while the cancer stem like cells coexpressed cytokeratin-5 and -7, the large majority of tumor cells generated in xenografts are either cytokeratin-5^+^ or cytokeratin-7^+^, with <10% of the cells remaining double positive [203]. According to these findings it was proposed that adenosquamous carcinomas are originated from a multipotent lung progenitor.

Given the difficulty to obtain tumoral tissue from SCLCs it is not surprising that only few studies have attempted to characterize CSCs from these tumors. In this context, a recent study by Sarvi and coworkers provided evidence that CD133^+^ cells isolated from SCLC cell lines exhibit properties of CSCs in that they form colonies in vitro and tumors in vivo, are resistant to anti-tumor agents such as etoposide and remain undifferentiated when grown in serum-free medium, while they differentiated in serum-rich medium [204]. Interestingly, these CD133^+^ cells express neuropeptide receptors [204]. The frequency of cells expressing CD133 is increased in human SCLC lung biopsy samples derived from patients undergoing chemotherapy [204]. Interestingly, the ASCL1 transcription factor, required for the differentiation of lung neuroendocrine cells and expressed in lung neuroendocrine tumors, is required for CD133 and ALDH1A1 expression in SCLCs, as shown by experiments of intereference knockdown [205]. Importantly, the CD133^+^ cell population isolated from SCLCs was markedly more capable of initiating tumors in nude mice than the CD133^low/-^ cell population: ASCL1 was shown to be critical for the tumor-initiating capacity of CD133^+^ SCLCs [205]. Other investigators have provided evidence that in SCLC cell lines a small population of urokinase plasminogen activator receptor (CD87)-positive is chemoresistant and displays enhanced clonogenic activity [206]. These observations were further supported by an additional study showing that in SCLC SBC-7 cell line the expression of either CD133 or CD87, mutually exclusive, was associated to resistance to chemotherapy; however, the higher tumorigenicity was displayed by cells negative for CD133 and CD87 expression [207]. Jahchan and coworkers have isolated and characterized a population of long-term tumor-propagating cells in a mouse model of SCLC; this population is characterized by high levels of CD24 and EpCAM expression and was identified also in primary human SCLCs [208]. These cells are proliferative and display a peculiar transcriptional profile characterized by elevated MYC activity; reduction of MYC activity in these cells inhibits long-term propagation of these tumor progenitor cells but does not affect their short-term growth [208].

## 10. Lung Cancer Xenotransplantation Assays

In parallel to the studies on the characterization of lung cancer stem cells, some investigators have explored xenotransplantation models generated using freshly resected patient tumor cells immediately transplanted into immunocompromised mice without an intermediate in vitro culture step. Using this approach, Cutz and coworkers reported the successful growth of primary lung xenotransplants implanted at the level of the subrenal capsule: particularly, they obtained the growth in the immunocompromised mice (NOD/SCID) of various types of primary lung cancers [209]. The xenografts retained the major histologic features and karyotypic features of the original cancers from which they were derived [209].

John and coworkers used a different methodology based on the subcutaneous xenotransplantation of tissue fragments derived from primary lung cancers; in this study based on the analysis of xenotransplantation of 157 primary lung cancers, these authors observed that 40% of transplanted tumors engrafted [210]. Some factors clearly influenced the rate of engraftment: squamous histology, poor differentiation and larger tumor size favored engraftment; importantly, the engraftment was preferential for KRAS-mutated tumors, compared to EGFR-mutated tumors [210]. A comparable methodology was used by Fichtner and coworkers and on 102 transplanted lung cancer fragments growth of xenotransplants was observed in about 24% of cases [211]. Zheng and coworkers have used this methodology of xenotransplantation and have obtained the development of 10 patient-derived NSCLC xenograft models, representative of various genetic abnormalities observed in these tumors, such as EGFR mutations, KRAS mutations, FGFR1 amplifications and MET amplifications [212]. It was suggested that the use of these xenotransplantation models could be used as an experimental platform for the development of personalized therapies for NSCLC patients [213]. Ilie and coworkers have subcutaneously xenografted 100 NSCLC tumor specimens into immunodeficient mice: the engraftment rate was 60% for squamous carcinoma and only 13% for adenocarcinomas [213]. Patients for whom xenografts were obtained had a significantly shorter disease-free survival compared to patients whose tumors failed to generate xenografts [213]. These xenografts allowed the isolation of a collection of cell lines, largely reflecting at molecular and histological level the tumors from which they were derived [213]. Lee and coworkers have established patient-derived xenografts using NSCLC brain metastasis surgical samples and have explored their utility for the development of personalized treatments. Interestingly, NSCLC brain metastases were more suitable than primary tumor specimens (74% vs. 23%) for xenograft development [214]. Xenografts derived from NSCLC brain metastases recapitulated the histologic, genetic and functional properties of the corresponding parental tumors. Tumor spheres isolated in vitro from these xenografts conserved their brain metastatic potential and can be used to screen drug sensitivity to a large spectrum of drugs [214]. Russo and coworkers have recently reported a methodology for obtaining lung tumor xenografts using either fresh tumor sections or tumor fragments cultured for 24h in organotypic cultures [215]. The cellular and molecular properties of tumor xenograft obtained with the two procedures are highly comparable [215].

Moro and coworkers reported the establishment of a large panel of tumor xenografts derived from NSCLC patients, recapitulating and maintaining tumor features over 10 passages in mouse [216]. Significant correlation rates between PDX rate and patient’s prognosis, tumor stage and stem cell markers (CD133^+^CXCR4^+^EpCAM^−^) was observed [216]. The findings of this study further supported the relevance of PDXs as suitable preclinical models reflecting parental patient tumors and tumor aggressiveness and may represent a tool for the development of personalized therapies [216]. Interestingly, some studies on xenotransplantation of NSCLC cells have shown the existence of heterogeneous tumor cell populations. In this context, particularly relevant was the study of Tiran and coworkers reporting the isolation from an adenocarcinoma lung patient resistant to chemotherapy of two tumorigenic cell populations, one initiated in spheroid cell cultures, with epithelial phenotype, expression of CSC markers (ALDH1, CD133) and the other one growing as adherent cells and characterized by positive expression for mesenchymal markers and negative expression for CSC markers [217].

The development of xenotransplantation models of SCLC is particularly important since surgically resected tissue of this tumor is rarely available. In 2009 Daniel and coworkers reported the development of a primary xenograft model of SCLC in which endobronchial tumor specimens obtained from chemo-naïve patients are transplanted and propagated in vivo into immunodeficient mice [218]. Tumor cell lines have been obtained from the tumor xenotranplants [218]. More recently, Leong and coworkers have shown that SCLC cells obtained from endobronchial ultrasound-guided transbronchial aspiration are successfully xenotransplanted into immunodeficient mice (about 80% of engraftment) [219]. Stable cell lines were obtained from these xenografts that maintained in 90% of cases a SCSL phenotype and exhibited genotypic properties similar to those of the parental tumor cells [219].

Importantly, a recent study provided evidence that xenotransplants can be obtained from the implantation in immunocompromised mice of circulating tumor cells isolated from the blood of SCLC patients. This study was originated from the observation that circulating tumor cells are frequently present in the blood of patients with SCLC and their number is inversely associated with survival [220]. The circulating tumor cells in these patients have been defined as cells co-expressing EpCAM and cytoketarins (8, 18 and 19), without expression of the CD45 hematopoietic marker [220]. Based on this observation, Hodgkinnson et have proposed to verify the presence within circulating cells of tumor cells capable of generating tumors in immunodeficient mice. To this end, these investigators have isolated Ficoll fraction from PB containing circulating tumor cells and have injected them subcutaneously to immunodeficient mice and have observed that about 4 months after injection these cells are able to induce the formation of tumors, exhibiting typical features of SCLCs [41]. The circulating tumor cells were higher in patients with chemorefractory disease, compared to their number observed in chemosensitive patients [221]. Genomic analysis of tumors growing into immunodeficient mice showed consistent similarity with primary tumors [221]. The tumor xenotransplants originated from circulating tumor cells can be serially passaged and exhibited a sensitivity to cisplatin and etposide similar to that observed in clinical setting [221]. In conclusion, these observations clearly indicate that circulating tumor cells in SCLC patients possess tumor-initiating capacity and represent a source of cells for the tumor cells for the development of experimental tumor models [221]. Drapkin and coworkers recently reported the efficient generation of PDX models from patients with SCLC, obtained either starting from tumor biopsies or circulating tumor cells [222]. Whole-exome sequencing studies showed that genomic alterations are maintained between patient tumors and PDXs [222]. In vivo treatment with etoposide and platinum in 30 PDX models showed greater sensitivity in PDXs from chemotherapy-naïve patients, and resistance to chemotherapy was associated to increased expression of a MYC gene signature [222]. Importantly, serial CTC-derived PDXs generated from individual patients at different time points recapitulated the changing drug sensitivities of these patients’s disease, thus supporting the potential translational implications of this strategy [222].

## 11. Mouse Models of Lung Carcinoma

Various mouse models of lung cancer have been developed to try to identify lung tumor-initiating cells. In two murine models of lung cancer adenocarcinoma, tumors are initiated with conditional activation of oncogenic KRas^G12D^ or with conditional p53 deficiency in p53^Flow/Flow^ mice. In both these models, lung adenocarcinomas develop, recapitulating the main features of the corresponding human tumors. In contrast, mice bearing a mutant human EGFR transgene, EGFR^T790M-L485R^, develop adenocarcinomas representative of lung adenocarcinomas occurring in non-smoker patients. Initial studies based on the analysis of KRasG12D activation by inhaled adenoviral Cre have led to propose that the initiating cells for lung adenocarcinoma are putative bronchioalveolar stem cells (BASCs), located at the bronchioalveolar duct junction [223]. In this lung cancer mouse model oncogenic KRAS induces an expansion of BASCs, a phenomenon requiring the activation and the integrity of the Polycomb protein Bmi 1 [224]. This conclusion is directly supported by the observation that Bmi 1 deficiency inhibits mutant-induced BASC expansion and lung oncogenesis [224]. 

Cho and coworkers have explored in detail the KRas^G12D^-driven lung adenocarcinoma model and have identified the cell types that seem to be responsible for the origin of these tumors. To better define this initiating cancer cell population those authors have used three membrane markers, EpCAM, MHCII and ICAMI to enrich bronchiolar ciliated, Clara and alveolar type II cells, respectively. The EpCAM^+^MHCII^−^ cell fraction, highly enriched in bronchioalveolar Clara cells, is more tumorigenic than the EpCAM^+^MHCII^+^ cell fraction enriched in alveolar cells [225]. Furthermore, EpCAM^+^MHCII^−^ cells were able to generate, when injected in immunodeficient mice, tumor heterogeneity [225]. According to these observations, it was concluded that bronchioalveolar Clara cells are the origin of the cellular elements generating lung adenocarcinomas in KRas^G12D^ mice [225]. 

The problem of identifying the cell of origin of KRas^G12D^-induced lung adenocarcinomas was re-explored in a recent study where a peculiar strategy was used to express the mutant KRAS oncogene at the level of two different cellular compartments of epithelial bronchioalveolar cells. The first strategy allowed oncogene expression in the secretory Clara cells, in the bronchioalveolar duct junction (BADJ) and in some alveolar cells: these CC10-positive cells do not give rise to tumors in the airways, undergo hyperplasia at the BADJ and give rise to tumors in the alveoli. The second strategy allowed oncogene expression at the level of alveolar type II cells, Clara cells in the terminal bronchioles and putative bronchioalveolar-stem cells as cells of origin for KRAS induced lung hyperplasia [226]. However, only type II cells seemed able to progress to adenocarcinoma [226]. Another study reinforced the concept that alveolar type II cells, which are able to generate type I alveolar cells, could represent the tumor-initiating cell in the KRas-driven model of lung adenocarcinoma. Thus, Lin and coworkers have developed new mouse models of lung adenocarcinoma allowing selective manipulation of gene activity in surfactant associated protein C (SPC)-expressing cells, including type II alveolar cells and bronchioalveolar stem cells that reside at the bronchioalveolar duct junction [227]. Activating KRas alone or in combination with p53 removal in murine SPC^+^ cells lead to NSCLC development in alveoli [185]. To further stress the notion that alveolar type II cells can be the originating cells for NSCLCs, these authors have introduced and expressed mutant EGFR into SPC^+^ cells and have observed tumor formation in alveoli [227]. According to these findings it was proposed a model in which lung adenocarcinoma can initiate in type II alveolar cells [227].

Curtis and coworkers have addressed a very important problem because they have explored whether in different mouse lung carcinoma models the tumor genotype could represent an important determinant of the lung cancer initiating cells [228]. Thus, they have explored the three most common mouse lung cancer models: KRas^G12D^; KRas^G12D^in mice with conditional p53 deficiency in p53^flox/flox^; EGFR mutant. They have shown that, although the tumors formed in these adenocarcinoma models possess similar proportions of putative BASC-like population Sca-1^+^/CD45^−^/CD31^−^, they exhibit considerable differences in the phenotype of cells responsible for tumor initiation: thus, in adenocarcinomas induced by KRas alone, Sca-1 expression was not associated with tumor initiating/propagating activity; in KRas/p53flox lung adenocarcinomas, Sca-1 expression was associated with a considerable enrichment in tumor-initiating cells; in the case of EGFR-driven lung adenocarcinomas, the selection of Sca-1^−^ tumor cells exhibited a considerable enrichment in tumor-initiating cells [228]. These results indicate that heterogeneity of cells capable of driving tumor development in murine lung adenocarcinoma models, in function of the tumor genotype [228].

Pacheco-Pinedo and coworkers have explored the effect of the activation of Wnt/beta-catenin signaling in the context of KRas^G12D^-induced mouse lung carcinogenesis [229]. Activation of Wnt/β-catenin in the bronchiolar epithelium of the adult mouse lung does not itself promote tumor development [229]. However, the concomitant activation of Wnt/β-catenin signaling and expression of a constitutively active KRAS mutant, such as KRAS^G12D^, led to a marked increase of tumor formation, in terms of both tumor size and number of tumor lesions, compared with KRAS^G12D^ alone. Interestingly, activation of the Wnt/β-catenin pathway determined a change also of the tumor phenotype, inducing a phenotypic switch from bronchiolar epithelium to highly proliferative distal progenitors found in the embryonic lung [229]. This finding implies that Wnt/β-catenin signaling is able to induce transdifferentiation of bronchiolar epithelium (Clara cells) to distal epithelium progenitors and subsequently to distal epithelial and alveolar differentiation [229]. In conclusion, these observations have provided evidence that Wnt/beta-catenin activation in combination with KRAS mutations leads to a more aggressive form of lung cancer with an embryonic progenitor phenotype [229].

The study of KRAS-induced mouse adenocarcinomas allowed to define the various Ras-induced pathways acting downstream this mediator, that, can be potentially targeted in view of novel therapeutic strategies. In this context, particularly interesting were the results obtained by Kumar and coworkers who have shown that the GATA2 transcriptional network is strictly required for KRAS-oncogene-driven NSCLC [230]. This conclusion was supported by various lines of evidence: (i) loss of GATA2 reduced the viability of Ras-mutated NSCLCs, but not of WT NSCLCs; (ii) in KRAS-driven NSCLC mouse model, GATA2 loss strongly reduced tumor development. The study of the mechanisms by which GATA2 maintains Ras-pathway mutant NSCLC survival showed an important role of the regulation of proteasome activity mediated via control of the transcription factor NRF1 of the transcriptional activation of the IL-1 and NF-kB signaling pathways [230]. Furthermore, GATA2 regulates Rho target genes via STAT activation and this function is essential for the Ras-mediated survival of KRAS-mutant NSCLSs. Although GATA2 itself is not druggable, the combined proteasome and Rho signaling inhibition resulted in a robust suppression of KRAS-mutant tumor growth [230]. Another recent study provided evidence about a key role of the NOTCH pathway in KRAS-driven lung tumorigenesis. In fact, Maraver and coworkers have found that the NOTCH pathway is hyperactive in murine KRas^G12V^-driven NSCLC. Additional experiments have shown that the γ-secretase complex is required for KRAS^G12V^-NSCLCs and, conversely, pharmacological inhibition of γ-secretase arrests these tumors [231]. The activated NOTCH pathway upregulated pERK levels in KRAS-mutant NSCLCs through a mechanism involving HES1 which directly binds to and repress the promoter of DUSP1, encoding a dual phosphatase that dephosphorylates phospho-ERK [231]. According to this mechanistic interpretation, gamma secretase inhibitors upregulate DUSP1 and decrease phosphor-ERK levels [231]. These observations support a therapeutic potential for gamma secretase inhibitors in NSCLCs with KRAS mutations. In line with the above-mentioned observations, it was found that a subset of NSCLC patients displays a hyperactivated NOTCH pathway; these patients have high HES1 and low DUSP1 levels and are associated with a poor clinical outcome [231]. Corcoran and coworkers reported the results of a pooled shRNA-drug screen designated to identify MEK inhibitor-based targeted therapy for KRAS mutant lung cancers. Using this strategy, they identified the anti-apoptotic BH3 gene family gene BCL-XL as a top hit [232]. A chemical inhibitor of BCL-XL (ABT-263), in combination with a MEK inhibitor led to a marked apoptosis of KRAS mutant NSCLC cell lines [232]. Importantly, this drug combination induced regression of tumors in a KRAS-driven lung cancer mouse model [232]. These observations support combined MEK/BCL-XL inhibition as a potential therapeutic approach for KRAS mutant lung cancers [232]. The role of NOTCH pathway in KRAS-driven mouse lung tumorigenesis is further supported by another recent study showing that the cell population of KRAS-driven lung tumor is heterogeneous and within this cell population CD24^+^ITGB4^+^NOTCH^high^ cells are capable of propagating tumor growth in a clonogenic and orthoptic serial transplantation assay [167]. All four NOTCH receptors are expressed in these tumor propagating cells, but among them NOTCH3 is the receptor more relevant from a functional point of view [167]. Importantly, the tumor propagating cell population is enriched after chemotherapy and the gene signature of mouse tumor propagating cells correlates with poor prognosis in human NSCLC [230]. Inhibition of NOTCH signaling in tumorspheres isolated from NSCLC patients reduced their proliferation in vitro and in vivo [167]. 

Another important model of mouse lung cancerogenesis was based on the expression of clinically relevant EGFR mutants into mouse pneumocytes. As mentioned above, somatic mutations in exons encoding the tyrosine kinase domain of the EGFR gene are frequently observed in human lung adenocarcinomas and are associated with sensitivity to some TK inhibitors. The large majority of EGFR mutations or short deletions occur at the level of exon 19. To evaluate the oncogenic potential of EGFR mutations, transgenic mice expressing either EGFR^ΔL747-S752^ or EGFR^L858R^ in type II pneumocytes have been developed [233]. Although with different latencies, these mice developed lung adenocarcinomas highly reminiscent of human bronchio-alveolar carcinoma [233]. The same authors have developed another mouse model using another EGFR mutant [233]. In addition, animals with double EGFR^L858R+T790M^ were also developed [38]. Both the transgenic mice develop lung tumors that are resistant to EGFR TK inhibitors [38]. Particularly interesting both for the understanding of the mechanisms of EGFR-mediated lung oncogenesis and for the identification of therapeutic strategies to bypass TKI resistance was the observation of the key role of the FOXO1/KLF6 axis in EGFR action. In fact, Sangodkar and coworkers have shown that in EGFR-mutant lung adenocarcinoma cells the KLF6 transcription factor is completely downmodulated via a transcriptional repressive effect mediated by the transcription factor FOXO1 [234]. Interestingly, the drug trifluoroperazine hydrochloride, which inhibits the FOXO1 nuclear export, restored sensitivity of EGFR TK resistant lung cancer cells in vitro and in vivo [234]. These observations suggest that the KLF6/FOXO1 transcriptional network may represent an important EGFR oncogenic signaling pathway that can be targeted for the treatment of metastatic lung adenocarcinoma.

KRAS^G12D^, KRAS^G12C^, KRAS^G12V^, KRAS^G12A^ were enriched in lung adenocarcinomas in mice, as they were in humans; KRAS^G12C^ mutations were less frequent than in human disease, while KRAS^G12R^ and KRAS^G13R^ were more frequent in murine disease [235]. KRAS^G12C^ is the most frequent KRAS mutation observed in current/former-smokers and it is approximately three times less prevalent in never-smokers in which KRAS^G12D^ is the dominant mutation. The prevalence of various KRAS mutants in lung cancer is largely mediated by the mutational processes that lead to lung cancer initiation. Consistent variability in growth rate, survival and drug response has been observed in KRAS mouse lung cancer models, despite the shared initiating event; therefore, it is difficult to translate the observations made in these models into clinical application [236]. Many factors may contribute to the heterogeneity of KRAS-mutant mouse lung cancer models, but a predominant role is played by the acquisition of cooperating genetic alterations spontaneously acquired during tumor development [237]. Interestingly, the mutational landscape of murine lung adenocarcinomas originated by carcinogens [methyl-nitrosourea (MNU) and urethane] or by genetic activation of KRAS (KRAS^LA2^) was compared [44]. Although the MNU-induced tumors carried exactly the same initiating KRAS mutation as seen in the KRAS^LA2^ model (KRAS^G12D^), MNU tumors display a much higher number of single nucleotide mutations than tumors observed in KRAS^LA2^ mice; in contrast, KRAS^LA2^ tumors exhibited a significantly higher aneuploidy and copy number alterations comparted to the carcinogen-induced tumors [44]. This finding suggests that the type of somatic alteration and selection during tumor progression depends on genetic events underlying initiation of tumorigenesis [44]. It is important to point out that the carcinogen-initiated tumors acquire clonal KRAS oncogenic mutations: KRAS^G12D^ in MNU-initiated tumors. Interestingly, a comparison of all validated mouse mutations observed in carcinogen-induced and in genetic-induced mouse lung tumors with those observed in human lung adenocarcinomas revealed substantial overlap in driver genes harboring consequential mutations [44]. Interestingly, a recent study compared the genomic abnormalities observed in lung adenocarcinomas driven by mutant KRAS or EGFR and by overexpression of MYC; tumors from models driven by strong cancer drivers, such as mutant EGFR and KRAS, harbored few mutations in known cancer genes, whereas tumors driven by MYC acquired recurrent clonal KRAS mutations. It is important to note that the number of point mutations observed in murine EGFR- and KRA-driven tumors are much lower than those observed in corresponding human tumors [238]. 

It is very well established that chronic cigarette smoke is a major determinant in lung cancer development, being directly responsible for the majority of these tumors occurring worldwide. The contribute of cigarette smoke and its components to genetic abnormalities and to epigenetic changes was well documented in many recent studies, above mentioned. However, only few studies have addressed the analysis of the role of smoke-induced changes in the genesis of lung cancer in animal experimental models. In this context, particularly interesting was a recent study showing that long-term exposure of human bronchial epithelial cells to cigarette smoke condensate induces various epigenetic changes, particularly at the level of DNA methylation, comparable to those observed in smoking-related NSCLC, that sensitize the cells to malignant transformation with a single KRAS mutation [239].

The study of animal models of lung adenocarcinoma helped to define the role of many other genes that cooperates with KRAS in inducing tumor development. Thus, the study of KRAS-mutant mouse lung models helped to better define the role of normal and mutant TP53 in lung tumorigenesis. The study of these models showed that low levels of oncogenic KRAS fail to engage TP53-mediated tumor suppression; only when oncogenic RAS signal flux exceeds a critical threshold, TP53-mediated tumor suppression is triggered [240]. Importantly, TP53 restoration in mouse models of progressing NSCLCs, initiated by sporadic oncogenic activation of endogenous KRAS, failed to induce significant regression of established tumors, although it was able to decrease the proportion of high-grade tumors [240]. Turell and coworkers characterized the transcriptional and functional phenotypes of murine lung tumors that lack TP53 or express a contact (R270H) or a conformational (R172H) TP53 mutant. These mutants were evaluated either in the absence or in the presence of wild-type TP53 functionality. Wild-type TP53 exerts a dominant-suppressive effect on mutant tumors, since all genoptypes are equally sensitive to its restoration in vivo [241]. This observation indicates that TP53 restoration is a potential therapeutic approach suitable for all TP53 genotypes and helps to explain the high incidence of TP53 loss-of-heterozygosity observed in mutant TP53-mutant lung carcinomas. In contrast, TP53 gain-of-function and their vulnerabilities vary according to the mutation type: thus, TP53^R270H^ mutant displayed a sensitivity to Simvastatin, a drug sensitivity observed also in human tumors with this type of TP53 mutation (R273H).

Another molecular event cooperating with KRAS in driving lung adenocarcinoma development is represented by MYC activation. Recent studies have provided evidence that MYC activation contributes to lung adenocarcinoma progression in KRAS^G12D^-driven lung tumors in vivo, showing that MYC drives the conversion of indolent adenomas to aggressive, inflammatory, and immune-suppressed adenocarcinomas; MYC deactivation in these tumors causes tumor regression [242]. Importantly, it was shown a major role for MYC in re-programming the tumor microenvironment, particularly for that concerns the inflammatory and immune components of tumor stroma [242]. Given this important contribution of MYC to KRAS-driven lung tumorigenesis, it is not surprising that a pharmacological treatment based on combining DNA demethylating agents (azacytidine) and histone deacetylase inhibitors, able to inhibit MYC signaling, reduces MYC-driven cell proliferation, associated with an enhancement of immune signaling [242]. This dual epigenetic therapy could synergize with immune checkpoint blockade and could drive a potent anti-tumor response [243].

Other genetic alterations cooperate with KRAS mutation in the development of lung adenocarcinomas. NFkB transcription factor is a key regulator of inflammatory and immune responses, as well as of cell survival. The IkB kinase (IKK) complex, composed of IKKα, IKKβ and IKKγ (NEMO) is essential for the activation of NFkB; particularly, IKKα regulates both canonical and noncanonical NFkB signaling, as well as NFkB-independent functions. Initial studies in animal models provided evidence NFkB activity is required for KRAS-initiated lung adenocarcinoma development because it supports cell survival [244]. Recent studies have addressed a role for IKKα in cooperation with KRAS in the induction of lung adenocarcinomas. Inactivating mutations (2.2% of cases) and hemizygous deletions (22% of cases) of CHUK, the locus encoding IKKα, have been observed in human lung adenocarcinomas [240]. In these patients, CHUK deletion was associated with a reduced survival, thus suggesting that IKKα deletion may contribute to the development of a more malignant phenotype of lung adenocarcinomas. This hypothesis was directly supported by studies in a KRAS-mutant mouse model of lung adenocarcinoma; in fact, lung-specific IKKα deletion promotes KRAS^G12D^-mediated lung adenocarcinoma development, in association with elevated NOX2, down-regulated NRF2, accumulated ROS and attenuated cell senescence [240]. These observations led to conclude that IKKα loss favors lung adenocarcinoma development protecting lung epithelial cells from the anti-tumorigenic effect promoted by KRAS activation and related to ROS production and induction of cell senescence [240]. 

Animal models were fundamental to better define the role of the methyltransferase EZH2 in lung cancer development. EZH2 acts in the context of the Polycomb Repressive Complex 2 (PRC2), trimethylating Histone 3 at Lysine 23 and, through this mechanism, elicits gene silencing. Studies using EGFR-mutant lung cancer models showed that EZH2 inhibition increases the sensitivity to topoisomerase II G12D/adenocarcinomas [245]. More recently, it was shown that PRC2 is a critical regulator of KRAS-driven NSCLC progression: in fact, EZH2 overexpression enhances KRAS-induced lung adenomagenesis and inflammation [245]. Additional TP53 inactivation activates an epithelial-to-mesenchymal transition program, leading to invasive mucinous adenocarcinoma [246]. The CRISP technology allowed to identify genes that act as tumor suppressors and may inhibit the KRAS-driven lung cancer development. This approach allowed to identify the histone demethylase, whose expression is down-regulated in NSCLC, as a tumor suppressor in the KRAS-driven mouse model: in fact, UTX knockout promotes lung tumor progression in KRAS^G12D/+^ mouse model [247]. The tumor-promoting effect induced by ATX loss is mainly mediated via EZH2 upmodulation; in line with this finding, these tumors are sensitive to EZH2 inhibitors [247].

A single endogenous mutant KRAS allele is sufficient to promote lung cancerogenesis in mice, but malignant progression requires additional genetic alterations; interestingly, advanced lung tumors from KRAS^G12D/+^; p53^null^ mice frequently display KRAS^G12D^ allele enrichment [248]. The increased expression of the mutant allele implies a metabolic reprogramming of the tumors, with a glycolytic switch and an increased channeling of glucose-derived metabolites into TCA cycle and glutathione biosynthesis [248]. This finding is important because shows that mutant KRAS copy gain creates unique metabolic dependencies that can be exploited for the selective targeting of these tumors [248]. 

Other studies have shown that KRAS mutations and other cooperating mutations determine a metabolic reprogrammation of lung cancer cells, determining some vulnerabilities in these cells, potentially amenable to selective targeting. As above repeatedly mentioned, about 20% of KRAS mutant lung adenocarcinomas display KEAP1 loss, often associated with KRAS mutations. Using a CRISPR/Cas-9-based approach in a mouse model of KRAS-driven lung adenocarcinoma, it was examined the effect of KEAP1 loss in lung adenocarcinoma development and hyperactivates NFE2L2 expression [249]. KEAP1 is a negative regulator of Nuclear Factor Erythroid 2-Like 2 (NFE2L2), a master regulator of oxidative metabolism. Importantly, KEAP1/KRAS-mutant lung adenocarcinomas are dependent upon increased glutaminolysis and are therapeutically sensitive to pharmacologic inhibition of glutaminase [249]. These observations provide a strong rationale for stratification of lung adenocarcinoma patients harboring KRAS/KEAP- or KRAS/NFE2L2-mutant tumors as potential candidates to respond to glutaminase inhibition [249]. KEAP1^−^/KRAS mutant cells undergo apoptosis when grown in low glutamine conditions through a pathway requiring the activation of Activating Transcription Factor 4 (ATF4) [250]. Knockdown of ATF4 in KRASmut/KEAP1^−^ cells inhibited the apoptosis triggered by low glutamine levels [250]. In line with these observations, the levels of ATF4 targets are much higher in KRASmut/KEAP1^−^ lung cancer cells, compared with tumor cells wild-type for KEAP1 or KRAS [250]. The ATF4 modulation exerted by oncogenic KRAS is required for apoptosis suppression via control of asparaginase biosynthesis: inhibition of AKT suppresses asparaginase expression and, combined with low extracellular asparagine, decreased the growth of KRAS-mutant lung cancers [250].

As above shown, the study of KRAS-mutant mouse lung cancer was of fundamental importance to try to define some vulnerabilities of these tumor cells and to define potential therapeutic approaches. Using the KRAS^G12V^/TRP53 mutations model, it was shown that: systemic ablation of MEK or ERK kinases in adult mice prevent tumor development but results in an unacceptable toxicity; ablation of c-RAF expression in advanced tumors results in tumor regression, with no development of resistance mechanisms [251]. It is particularly important to underline that systemic abrogation of c-RAF expression does not inhibit canonical MAPK signaling, thus resulting in limited toxicities [251]. The therapeutic effect observed upon c-RAF inhibition is seemingly related to the inability of c-RAF protein to interact with other partners. In this context, there is evidence that c-RAF inhibits apoptosis in a kinase-independent manner, apparently thorough Bcl-2 and pro-apoptotic kinases ASK1 and MST2; in line with this hypothesis, c-RAF inhibition in KRAS-mutant tumor cells induces activation of Caspase-3 [251]. According to these findings and to numerous previous studies, it was concluded that MAPK signaling pathway has shown three classes of targets, related to their effects as KRAS effectors: those that have no effect in preventing the development of KRAS-driven lung adenocarcinoma (A-RAF, B-RAF, CDK2 and CDK6); those that prevent tumor development, but cause unacceptable toxicities if completely ablated (MEK1-2, ERK1-2 and CDK1); those that prevented tumor development (c-RAF and CDK4) when ablated concomitantly with KRAS oncogene expression and do no not induce significant toxicities when targeted systematically [251].

KRas^G12D^ fails to induce the formation of LSQCC in mice; however, Ji and coworkers have reported that a significant proportion of KRas^G12D^ mice lacking LKB1 in the lungs develop mixed lung tumors of ADC and LSQCC types [209]. The loss of LKB1 alone did not induce lung cancer formation in mice. Recently, a procedure was reported allowing the development of a mouse model for LSQCC. This model was based on the development of kinase-dead IKKalpha knockin mice: these mice spontaneously develop LSQCC, associated with IKKalpha downregulation and marked pulmonary inflammation. IKKalpha downregulation upregulated the expression of p63, keratin 5 and Trin 29 at the level of pulmonary epithelial cells: the expansion of population is followed by LSQCC formation, accompanied by inflammation-associated deregulation of oncogenes, tumor suppressors and stem cell regulators [252]. Additional experiments carried out in these animals allowed to determine that *IKKalpha* mutant macrophages promote the tumoral transition of IKKalpha^low^K5^+^p63^+^ cells to tumor squamous cells [252]. The tumors developing in this mouse model resemble human LSQCC [252]. In a subsequent study the same authors showed that biallelic inactivation of *LXB1* and *PTEN* in the mouse lung induces the development of squamous adenocarcinoma, recapitulating the biology, the gene expression profile and the microenvironment observed in human disease [253]. These squamous tumors expressed markers p63, SOX2 and KRT5 and exhibited a transcriptome resembling that observed in human SCCs [253]. The isolation of Sca1^+^NGFR^+^ cells leads to an enrichment of cells with properties of tumor-initiating cells able to reproduce the disease in serial xenotransplantation assays [253]. *LKB1* loss is an important mechanism involved in the control of the cellular identity of lung adenocarcinoma cells, determining their transdifferentiation to squamous cell carcinoma. In fact, it was shown that LKB1 loss in lung adenocarcinoma activates *YAP* (a major effector of the Hippo pathway) which in turn upregulates *ZEB2* expression and represses *DNp63* transcription which inhibits squamous cell transdifferentiation; during transdifferentiation, YAP is inactivated, thus relieving the ZB2-mediated repression of DNp63 and thus inducing the program of squamous cell differentiation [254]. Thus, YAP represents an important barrier for lung cell fate conversion. Ectopic expression of YAP in type II alveolar epithelial cells led to hyperplasia in mouse lungs. *YAP* overexpression in the KRAS^G12D^ lung cancer mouse model accelerates lung adenocarcinoma progression; conversely, *YAP* deletion markedly delayed tumor progression in *KRAS*-mutant mice [255]. The antiapoptotic protein surviving could be the downstream mediator of YAP, responsible for promoting malignant progression of *LBK1*-deficient lung adenocarcinomas [254]. Other studies have shown that YAP deletion completely blocks *KRAS*^G12D^ and *TP53* loss-driven adenocarcinoma initiation and progression, whereas heterozygosity for *YAP* partially suppresses lung cancer growth and progression [256]. In a more recent study it was shown that *LKB1* deletion in mouse cells that had *KRAS* activation long before was sufficient to induce the generation of tumors with squamous characteristics [257]. This observation suggests the existence of a transition from an adenocarcinoma *KRAS*-mutant to a squamous tumor *KRAS*-mu/LKB1-null [257]. Using this mouse model, it was provided evidence that de-repression of squamous genes through loss of PCR2 accompanies the squamous transition [257]. Club cells and basal stem cells were identified are the most fit populations involved in the generation of adenosquamous tumors [257].

A considerable controversy surrounds the identity of the cell of origin of *KRAS*^G12D^-induced lung cancers. For many years it was believed that these tumors derive from transformed alveolar type II cells, characterized by the expression of the Surfactant Protein C (SPC) [223]. Some studies have, however, suggested the cellular origin of these tumors from a rare cell population, co-expressing SPC and Clara cell antigen 10 and localized at the level of bronchio-alveolar duct junction [223]. However, using various genetic approaches, Xu et al. have reached the conclusion that AT2 cells, but not Clara cells, are the predominant cancer-initiating cells of *KRAS*^G12D^-induced lung adenocarcinoma [226]. Finally, a recent study by Sutherland provided evidence conciliating these different studies [258]. In fact, these authors have used two mouse models of human lung adenocacrcinoma, based on activation of *KRAS*^G12D^ alone or in combination with *TP53* loss in various lung epithelial cells using cell-type-restricted Adeno 5-cre viruses [258]. Using this technology, it was provided clear evidence that both CC10^+^ Clara cells and type II alveolar SPC^+^ cells are able to generate adenocarcinoma in response to *KRAS*^G12D^ activation [258].

Other recent studies have attempted to define the genetic factors that may modify the cellular origin of KRAS-driven cancers. Using the KRas^G12V^ mutant to transform murine lung cells Mainardi and coworkers have reached the conclusion that only SPC^+^ type II cells are permissive to the transforming activity of this KRAS mutant, first forming hyperplastic lesions, progressing first to adenomas and then to adenocarcinomas [259]. However, induction of *KRAS*^G12V^ expression in lung cells by intratracheal infection with adenoviral particles generated only bronchiolar and bronchioalveolar duct junction hyperplasias, composed by CC10^+^ Clara cells, progressing to adenomas and not to adenocarcinomas. According to these observations it was concluded that various lung cancer cell types generate benign neoplasms following *KRAS*^G12V^ expression, but only SPC^+^ ATII cells were able to generate adenocarcinomas [259]. 

Inhibition on NOTCH signaling markedly reduced *KRAS*-induced adenocarcinoma formation, while NOTCH activation in bronchiolar cells rnhanced *KRAS*-induced lung adenocarcinomas [260]. Given these observations, these investigators have explored the molecular mechanisms that could modulate NOTCH expression during lung tumorigenesis, showing that *SOX2* overexpression determines transcriptional repression of *NOTCH1* and *NOTCH2* [260]. A partial inhibition of *SOX2* in *KRAS*^G12D^ mice allows bronchiolar tumor formation (papillary adenocarcinoma). These results suggest that the cell of origin of *KRAS*-induced lung tumors depends on the level of *SOX2* expression, modulating NOTCH signaling [260].

Recently, a mouse model of squamous lung carcinoma based on *SOX2* overexpression was described. As above mentioned, *SOX2* is amplified in about 20% and is overexpressed in 60−90% of LSQCC; furthermore, *SOX2* is frequently expressed in early LSQCC, thus suggesting that it may represent one of the molecular initiation events. Using lentiviral transfer of *SOX2* specifically to the mouse lung, it was tested its capacity to promote tumorigenesis in cooperation with various tumor suppressor genes, showing that SOX2 overexpression cooperates with *LKB1* loss to promote LSQCC formation [261]. Importantly, the tumors developed in these animals resemble the human counterpart of LSQCC for their histopathological features, biomarker expression and activation of relevant signaling pathways, such as STAT and mTOR [261].

As above mentioned, molecular studies have led to the identification of *SOX2* and *PRKCI*, as two genes frequently co-amplified in LSQCC and as potential regulators of lung CSCs. *PRKCI* encodes the Tumor Protein Kinase C iota (PKCι) that is overexpressed in LSQCC and its level of expression is predictive of poor clinical outcome. PKCι was able to drive LSQCC invasion and growth in vitro and in vivo and Mmp10 is a critical effector in these biological effects [66]. Genetic disruption of *PRKCI* in the LSL-*KRAS*^G12D^ mouse LAC model inhibits tumor initiation by blocking the expression of lung cancer initiating cells [66]. PRKCI was shown to be required for the maintenance of a tumorigenic phenotype in lung cancer cells possessing *PRKCI* amplification and in LSQCC [66]. Particularly, PRKCI phosphorylates SOX2, thus inducing its recruitment at the level of the promoter of Hedgehog (Hh) acyltransferase (HHAT), catalyzing a key step in Hh ligand generation; this PKCι-mediated phosphorylation of SOX2 is essential for the maintenance of the stem cell-like phenotype [66].

## 12. Cellular Origin of Small Cell Lung Cancer

The cells that upon oncogenic transformation initiate and maintain the small cell lung cancer (SCLC) are largely unknown. Some indirect arguments have historically suggested a cell origin different from NSCLC. In fact, human SCLCs usually localize at the level of middle bronchioles and express a battery of neuroendocrine cell markers, including calcitonin-related peptide and other neuropeptides expressed within neuroendocrine pulmonary cells. According to these findings, neuroendocrine pulmonary cells have been proposed as the cells of origin of NSCLCs [262]. Alternatively, it was proposed that SCLCs, given their multiple differentiation capacities (epidermoid carcinoma, adenocarcinoma and/or large cells carcinoma-like), could derive from a “common” undifferentiated progenitor.

The search for a cell of origin of SCLC is greatly complicated by the consistent difficulty to obtain primary tumor specimens, a starting biological material to isolate these cells. In fact, given the aggressive clinical development and the advanced status of diagnosis, patients with SCLC usually do not undergo tumor resection. In spite this major constraint, numerous cell lines have been isolated and established from SCLC patients and currently grown in vitro. These cell lines have represented the biological material for many studies of characterization of SCLC stem cells.

Side population fraction of cells was isolated from SCLC cell lines and was found to correspond to <1% of total cells: these side population cells displayed many properties of stem cells, including high proliferative capacity in vitro, high self-renewal capacity and reduced expression of neuronal differentiation markers, such as CD56 and CD90 [263]. Importantly, as few as 50 SP cells from H146 and H526 SCLC cell lines were able to induce tumor formation in recipient mice [263]. Purified SP SCLC cells were also characterized by the high expression of genes involved in drug resistance and by activation of NOTCH and Hedgehog signaling pathways [263]. 

Another study characterized sphere-forming cells (grown in serum-free medium) with stem-like properties from the SCLC cell line H446: these cells contained a higher proportion of cells with stem cell markers, such as CD133 and uPAR (urokinase plasminogen activator receptor, CD87) [264]. Both CD133^+^ and CD133^−^ cells are able to induce the formation of spheres, while uPAR^+^ but not uPAR^−^ cells are able to generate spheres [207]. In line with these observations, experiments on other small cell lung cancer cell lines have shown that both distinct subpopulations of CD133^+^ and CD87^+^ cells have the property of tumor-initiating cells and are chemoresistant [207].

The most important indications about the cellular origin of SCLCs derive from the study of some animal models of SCLC. Among these models, the most relevant was the model of the double knockout for both *TP53* and *RB1* in the lung epithelium of adult mice [265]. Nearly all these *RB1*/*TP53* double mutant mice develop SCLC; these cells express markers of neuroendocrine cells and have the capacity to metastasize. In order to evaluate the origin of SCLCs, inactivation of *RB1* and *TP53* in different cell compartments of the lung using cell-specific promoter adenoviral cre [calcitonin gene-related peptide (CGRP), specific for neuroendocrine cells; surfactant protein-C (SP-C), specific for AECII cells; secretoglobin CC10, specific for Clara cells] administration in *RB1^f^*^/*f*^*;p53^f^*^/*f*^ mice was performed [266]. These experiments provided evidence that neuroendocrine cells were the most probable cells of origin of SCXLC: in fact, CGRP-cre-driven *RB1*/*TP53* loss resulted in tumor development in all animals, with a mean tumor latency time of one year. SP-C-cre-driven deletion of *TP53*/*RB1* resulted in the generation of neuroendocrine tumors in about 50% of animals, with a more long tumor latency period. Inactivation of *RB1* and *TP53* in CC10-positive Clara cells induced the generation of only rare tumors with a very long latency period [266]. According to these observations it was concluded that neuroendocrine cells contribute to SCLCs, while Clara cells do not seem to be involved in SCLC formation [266].

Using the mouse model with *TP53* and *RB1* deletion, in the mouse lung epithelium evidence was provided about a crucial role of Hedgehog (HH) signaling in the genesis of SCLC [267]. In fact, Park and coworkers have provided evidence that the HH signaling pathway is constitutively activated in SCLC cells. The constitutive activation of the HH signaling molecule Smo promoted the clonogenetic activity of SCLC cells in vitro and tumor initiation and progression in vivo. Importantly, SMO deletion in mouse lung epithelial cells *TP53*/*RB1*-deleted abrogated SCLC tumor initiation [267], thus indicating an essential role for HH signaling in tumor initiation [267]. Pharmacological inhibition of HH signaling using cyclopamine, a Smo inhibitor, greatly decreased SCLC cell survival [267].

Using genome sequencing, the somatic evolution of mutations of the *TP53*/*RB1*-deleted mouse model of SCLC was investigated. According to this analysis, alterations in DNA copy number and complex genomic rearrangements have been detected, in the context of a low somatic point frequency in the absence of the mutagenic effects of tobacco [268]. Interestingly, alterations of the tumor suppressor *PTEN* have been observed in the majority of murine SCLC analyzed [268]. According to these findings it was proposed a model of progressive development of murine SCLCs, starting from Pulmonary Neuroendocrine Progenitors through intermediate Neuroendocrine Hyperplasia to SCLC through the progressive acquisition of driver mutations mainly represented by *RB1* deletion, *TP53* deletion, *MYCL* amplification and PTEN mutation [268].

MYCL acts as an oncogene in SCLC; recurrent amplification of the *MYC* family of oncogenes, including *MYCL* (7−9% of cases) was frequently observed in SCLC. Targeted overexpression of *MYCL* in a mouse lung cancer model markedly accelerated tumor development [269]. Mollaoglu and coworkers [114] generated a model of SCLC with elevated *MYC* expression and loss of *RB1* and *TRL53* [114]. MYC expression cooperates with *RB1* and *TRP53* loss in the mouse lung to promote aggressive, highly metastatic tumors, initially sensitive to chemotherapy and then relapsing, similar to human SCLC. *MYC* expression drives the induction of SCLC corresponding to the neuroendocrine-low variant, associated with high NEUROD1 expression [114]. Interestingly, targeted drug screening, showed that high MYC-expressing SCLCs are vulnerable to Aurora kinase inhibition, which, combined with chemotherapy, strongly suppresses tumor progression [114].

The study of genetically engineered mouse model systems of SCLC, in combination with the analysis of primary tumors, has contributed to the identification of *NFIB* as a key oncogene in SCLC. *NFIB* pertains to the *NFI* family of site-specific DNA binding proteins acting as key regulators of the expression of many cellular genes and playing a key role as master cell regulators of cell differentiation [270]. Dooley et al. identified *NFIB* amplification/overexpression within murine tissue of mice genetically engineered by TRP53 and RB1 conditional deletion, showing that NFIB expression was essential for proliferation and viability during transformation of murine SCLC and reported also recurrent NFIB amplification in about 15% of primary human SCLC [271]. More recent studies have strongly supported the oncogenic role of NFIB. Thus, Denny and coworkers provided evidence that NFIB is both necessary and sufficient to promote metastasis in vivo of SCLC tumors, through the reconfiguration of chromatin accessibility in SCLC cells [272]. Another study, based on a different mouse model of SCLC, further supported a role for NFIB in SCLC metastasis, showing also that this transcription factor is highly overexpressed in human metastatic high-grade neuroendocrine lung tumors [273]. Finally, Wu and coworkers showed that NFIB overexpression cooperates with *p53*/*Rb1* deletion to promote SCLC generation [274]. Transcriptional analysis showed that NFIB regulates the expression of genes related to axon guidance, focal adhesion and extracellular matrix-receptor interactions [274].

The definition of new potential therapeutic approaches in SCLC is of crucial importance. The study of *KRAS*^G12D^-induced or carcinogen-initiated mouse lung cancer models, showed that *PKM1* overexpression promoted tumor development, while PKM2 expression inhibited tumorigenesis [275]. Greater pyruvate kinase enzyme activity, a higher glycolytic rate and increased anabolic metabolism were observed in the PKM1-expressing tumors. Through the analysis of a large panel of SCLC cell lines, it was shown that these tumors express PKM1, whose expression is required for tumor cell proliferation [275]. Importantly, neuroendocrine lung cancers express PKM1 at levels much higher than NSCLC; particularly, the PKM1/PKM2 ratio was much higher in SCLCs than in NSCLCs [275]. Targeting of PKM1 may represent a potential target in SCLC tumor cells. 

### Lung Cancer Immunotherapy

The studies carried out in the last years have provided evidence that immunotherapy represents a new important therapeutic tool for NSCLC. These studies were largely based on immune check inhibitors. The immune checkpoint proteins Programmed cell Death protein 1 (PD-1) and Cytotoxic T Lymphocyte Associated 4 (CTLA-4) are receptors expressed on the surface of cytotoxic T-lymphocytes that interact with their ligands Programmed Death Ligand 1 (PD-L1) and Cluster Differentiated 86 (CD86) on antigen presenting cells, which helps the cancer cells evade T cell-mediated death. Immune check inhibitors prevent the receptors and ligands from binding each other, thereby disrupting signaling and restoring an anti-tumor immune response. Approved immune check point inhibitors include the anti-CTLA-4 agent ipilimumab; anti-PD-1 agents, Nivolumab and pembrolizumab; anti-PD-L1 agents atezolizumab and durvalumab. PD-L1 is expressed at variable levels on some tumor cells, including NSCLC cells and when expressed contributes to block the immune anti-tumor response.

Two phase III trials demonstrated improved OS and a favorable safety profile with the anti-PD-1 antibody nivolumab versus docetaxel in patients with previously treated advanced squamous (CheckMate 017) and non-squamous (CheckMate 057) NSCLC. Two-year overall survival rates with nivolumab versus docetaxel were 23% vs. 8% in squamous NSCLC and 29% and 16% in non-squamous NSCLC [126]. After 3 years, the estimated OS rates were 17% in the nivolumab group vs. 8% in the docetaxel group in the pooled population with squamous and non-squamous NSCLC [276].

Gettinger and coworkers reported 5-year follow-up results from an early-phase I study of nivolumab in a population of patients with advanced, pre-treated NSCLC [277]. The estimated 5-year OS rate was 16% for squamous and 15% for non-squamous NSCLC; 75% of the survivor patients received no subsequent therapy and were without evidence of disease progression at last follow-up [277].

Hellmann and coworkers have explored the safety profile and the therapeutic impact of the combined administration of nivolumab and ipilimumab in 78 NSCLC patients, randomized to receive two different schedules of these drugs [278]. Objective responses were observed, ranging between 38% and 47%, following the schedule of ipilimumab administration [278]. The safety profile of the drug co-administration was considered as manageable [278]. After this promising phase I trial, very recently a phase III trial evaluated PFS of stage IV or recurrent NSCLC patients with a high mutational burden (≥10 mutations per megabase), not previously treated with chemotherapy [279]. The objective response rate was 45% with nivolumab and 27% with chemotherapy; the PFS rate after 1 year was 13.2% with chemotherapy, compared with 42.5% with nivolumab plus ipilimumab [279]. Importantly, the benefit of nivolumab plus ipilimumab over chemotherapy, was consistent within subgroups, including patients with <1% or >1% of PD-L1 expression [279].

These studies have shown that nivolumab was more efficacious than chemotherapy in SCLC patients with metastatic NSCLC who had progressed during or after platinum-based chemotherapy. Carbone and coworkers compared nivolumab as first-line therapy in NSCLC patients with PD-L1 expression levels of 5% or more [280]. These investigators randomized 541 patients with expression levels of 1% or more to receive either nivolumab or platinum-based chemotherapy and the primary efficacy analysis included the 423 patients who met the 5% expression cut-off. After a media follow-up of 13.5 months, PFS was 5.9 months among patients treated with chemotherapy and 4.2 months among patients treated with nivolumab; median overall survival was 13.3 months with chemotherapy and 14.4 months with nivolumab [280]. 60% of chemotherapy patients subsequently received nivolumab. A subgroup analysis limited to patients with PD-L1 expression of 50% or more confirmed the absence of differences between therapies [280]. Treatment-related adverse events of grade 3 or 4 occurred in 18% of the patients who received nivolumab and 51% of those who received chemotherapy [280]. According to these findings, the authors of this study concluded that significant longer PFS than chemotherapy among patients with previously untreated stage IV or recurrent NSCLC with a PD-L1 expression level of 5% or more [280]. Some biases of this study, such as gender differences between the two groups of treatment and higher percentages of PD-L1 expressing tumors among the chemotherapy group of the study may have contributed to the absence of effects of nivolumab in this study.

In a phase III trial, 305 patients who had previously untreated advanced NSCLC with PD-L1 expression on at least 50% of tumor cells and no sensitizing mutation of the EGFR or translocation of the ALK gene, were randomly assigned to receive either pembrolizumab or the investigator’s choice of platinum-based chemotherapy. The PFS was 10.3 months in the pembrolizumab group versus 6.0 months in the chemotherapy group; The estimated rate of OS at 6 months was 80% in the pembrolizumab groups versus 72% in the chemotherapy group; the response rate was higher in the pembrolizumab groups than in the chemotherapy group (45% vs. 28%) [281]. Pembrolizumab was explored as first-line therapy in patients with advanced NSCLC, treatment-naïve. Interestingly, patients with ≥50% PD-L1 expression on tumor cells exhibited at 12 months a PFS of 52% and an OS of 85% [282]. These results were considered as promising for a long-term OS benefit in a part of treated patients. Importantly, pembrolizumab improved or maintained quality of life compared with that of chemotherapy [283]. Recently, a double-blind, phase III trial, randomly assigned 616 NSCLC patients with metastatic non-squamous NSCLC without *EGFR* and *ALK* mutations who were chemotherapy-naïve to receive pemetrexed and a platinum-based drug plus either placebo or pembrolizumab [284]. The estimated rate of overall survival at 12 months was 69% in the pembrolizumab-chemotherapy group and 49% in the chemotherapy-placebo group [284]. Improvement in overall survival was observed in both low and high PD-L1 expressing tumors [283]. Median progression-free survival was 4.9 months is the chemotherapy-placebo group and 8.8 months in the chemotherapy-pembrolizumab group [283]. The conclusion was that pembrolizumab administration may improve the survival of non-squamous *EGFR* and *ALK*-WT NSCLC with untreated metastatic disease. 

Patients who had received one to two previous cytotoxic chemotherapy regimens (one or more platinum-based combination therapies) for stage IIIB or IV non-small-cell lung cancer were randomized either to receive atezolizumab or to receive docetaxel [285]. Overall survival was significantly longer with atezolizumab than with chemotherapy in the PD-L1 expressing patient population: 13.8 months vs. 9.6 months. Patients in the PD-L1 low or undetectable subgroup also had improved survival with atezolizumab (12.6 months vs. 8.89 months). Overall survival improvement was similar in squamous or non-squamous histology [285]. Another study evaluated the anti-PD-L1 monoclonal antibody atezolizumab as first-line or subsequent therapy for patients with advanced NSCLC, with a PD-L1 expression ≥5% [286]. The objective response rate was 20% and compared favorably with historical controls; the median overall survival was from 15–23 months in three different cohorts of patients; PD-L1 status was a predictive marker in that patients with higher PD-L1 expression tended to have better responses; responses to treatment occurred regardless of *EGFR* or *KRAS* mutation status [286].

Other studies were focused to evaluate another anti-PD-L1 agent, durvalumab. Garassino and coworkers recently reported the results of a phase II (ATLANTIC study) involving the administration of durvalumab (anti-PD-L1) as third-line o later treatment for advanced NSCLC subdivided into three subgroups: (a) *EGFR*/*ALK*-mutants; (b) non-*EGFR*/*ALK* mutants with <25% or at least 25% PD-L1 expression; (c) non-*EGFR*/*ALK*-mutants with at least 90% of tumor cells with PD-L1 expression [287]. An objective response was observed in 12% of patients of cohort a, 16% in cohort b and 31% in cohort c [287]. These observations indicate that the non-*EGFR*/*ALK*-mutant NSCLCs respond better than EGFR/ALK^+^ tumors to durvalumab [287]. Antonia and coworkers recently reported the results from an interim analysis of the randomized, double-blind, phase III PACIFIC study comparing durvalumab as consolidation therapy with placebo in patients with stage III, locally, unresectable NSCLC that had no progressed after platinum-based chemoradiotherapy [288]. Both the median PFS (16.8 months vs. 5.6 months), the response rate (28.4% vs. 16%) and the median time to death (23.2 months vs. 14.6 months) were significantly longer among patients randomized to receive durvalumab than in those receiving placebo [288]. The results of this study are very important because suggest a potential clinical utility of immune check inhibitors for NSCLC patients at earlier stages of tumor development [288].

Other studies have evaluated the anti-tumor effect of ipilimumab administered in combination with chemotherapy (paclitaxel and carboplatin) to stage IV or recurrent chemotherapy-naïve SQNSCLC patients [289]. The conclusions of this randomized trial were negative, showing that the addition of ipilimumab to first-line chemotherapy did not prolong OS and did not improve PFS in patients with advanced SQNSCLC [289].

Lee and coworkers have performed a meta-analysis on five randomized trials, involving 3025 patients with advanced NSCLC; the patients analyzed in this meta-analysis were randomized to receive a checkpoint inhibitor (nivolumab, pembrolizumab, atezolizumab or docetaxel). Checkpoint inhibitors administration was associated with prolonged overall survival, compared with docetaxel; prolonged overall survival was observed in the EGFR-wild-type group, but not in the EGFR-mutant subgroup; prolonged survival was observed in the KRAS-mutant subgroup, but not in the *KRAS* wild-type subgroup; the relative treatment benefits were similar according to the smoking status, histology and performance status [290]. These observations indicate that checkpoint inhibitors, compared with docetaxel, prolong overall survival in second-line therapy in NSCLC; the absence of benefit in OS in EGFR-mutant patients suggest that in these patients these agents may be considered only after the failure of other effective therapies.

Another recent meta-analysis was based on the analysis of single-arm non-comparative clinical studies involving a total of 3404 patients with advanced NSCLC. The results showed an objective response rate corresponding to 18%, a 1-year overall survival of 45% and a PFS at 24 weeks of 42%; adverse events of grade 3-4 were observed in 12% of treated patients [100]. PD-L1 expression resulted predictive of the outcome of nivolumab immunotherapy [100].

As above repeatedly reported not all NSCLC patients are responsive to immunotherapy with immune check inhibitors (only 20% of NSCLC patients are responsive). Therefore, it is of fundamental importance to define biomarkers capable of predicting clinical response to immune checkpoint inhibitors. As reported in various clinical studies, the level of expression of PD-L1 on tumor cells seems to represent one parameter correlating with clinical response to immunotherapy. Particularly, the PD-L1 expression on tumor cells is regarded as the best available biomarker to predict the efficacy of checkpoint inhibitors in NSCLC; although there is a linear relationship between the size of the benefit of immune check-point inhibitors and the level of PD-L1 expression in NSCLC, tumor responses have been observed also in patients with low or undetectable PD-L1 expression [291]. However, PD-L1 protein expression, as assessed by immunohistochemistry on tumor cells, is neither prognostic nor predictive of benefit deriving from adjuvant chemotherapy in resected non-small cell lung cancer [292]. Positive PD-L1 expression was correlated with squamous histology, lymphocytic infiltrate, and *KRAS*, but not *TP53* mutation; EGFR-mutated tumors showed statistically non-significant lower PD-L1 expression [292]. A classification of NSCLCs according to PD-L1 expression on tumor cells and tumor-infiltrating lymphocytes was proposed: tumors characterized by high PD-L1 and high tumor-infiltrating lymphocytes were associated with a poor prognosis [293]. The expression of PD-1 in LSQCC is associated with a poor prognosis: in fact, univariate and multivariate survival analyses have shown that the PD-L1-positive patients have a poorer prognosis than the PD-L1-negative LSQCC patients, using the 1% cut-off value [294]. PD-L1 positivity in LSQCC is associated with a high Ki-67 labeling index [294]. Interestingly, PD-L1 is expressed in the large majority of pleomorphic carcinomas of the lung, a rare and aggressive subtype of sarcomatoid carcinomas of the lung; high PD-L1 expression in these tumors may predict a favorable prognosis in resected tumors [295,296].

The observations above reported indicate that the evaluation of PD-L1 expression on tumor cells represents an important biomarker in patients undergoing treatment with immune checkpoint inhibitors. Thus, it is not surprising that treatment with pembrolizumab in NSCLC required PD-L1 expression evaluation by immunohistochemistry assay; nivolumab and atezolizumab were approved without PD-L1 testing, although tests are available and recommended for both. Various PD-L1 immunohistochemistry assays (28-8, 22C3, SP142 and SP263) were used to assess PD-L1 expression in patients treated with PD-1/LD-L1 inhibitors [297]. The analysis of published studies indicates high concordance among 28-8, 22C3 and SP263 when assessing PD-L1 expression on tumor cell membranes; however, no concordant results were observed when these assays were used to detect PD-L1 expression on immune cells [297].

Mezquita et al. have evaluated the impact of lung immune prognostic index (LIPI) on the immune checkpoint inhibitor outcomes in patients with advanced NSCLC [298]. LIPI was based on the evaluation of the derived neutrophils/ (leukocyte minus neutrophils) ratio (dNLR) and lactate dehydrogenase level. The overall survival observed in a large set of NSCLC patients treated with anti-PD-1 or PD-L1 inhibitors or with chemotherapy showed that for patients treated with immune check inhibitors, but not with chemotherapy, there is a good correlation with the LIPI index: 3 months, 10 months and 34 months of OS associated with a poor, intermediate and good LIPI, respectively [298]. Therefore, the evaluation of LIPI might be useful for identifying patients unlikely to benefit from therapy with immune checkpoint inhibitor [298]. Many recent studies have highlighted the key role of cancer-specific neoantigens in determining cytolytic and T cell activity and, particularly, in predicting efficacy of immune checkpoint inhibition. A critical biochemical step in neoantigen presentation and cytolytic T cell responses is regulated by class I HLA, responsible for the presentation of intracellular peptides on the cell surface for recognition by T cell receptors. A loss of heterozygosity in HLA may facilitate tumor immune evasion. HLA LOH loss occurs in 40% of NSCLCs and is associated with PD-L1 positivity, high subclonal neoantigen burden, APOBEC-mediated subclonal frequencies, enrichment in metastatic sites suggests that HLA LKOH is an immune escape mechanism subjected to strong environmental selection pressures [91].

Rivzi and coworkers have recently published an analysis of the molecular determinants correlating with and influencing the outcome of therapy with immune checkpoint inhibitors in a large set of NSCLC patients. These studies raised many interesting findings and underlined the need of carefully exploring at molecular level NSCLC patients undergoing treatment with immune checkpoint inhibitors [299]. The major findings of this study were: Tumor mutation burden was greater in patients with durable clinical benefit than in those with non-durable clinical benefit; Durable clinical benefit was more common, and PFS was longer in patients at increasing tumor mutation burden; the fraction of copy number-altered genome was highest in patients with non-durable clinical benefit; tumor mutation burden and PD-L1 expression level were independent variables, and a combined tumor mutation burden and PD-L1 expression enriched for benefit to treatment with immune checkpoint inhibitors [299].

Another recent study explored the molecular features associated with response in patients with NSCLC treated with the combination of PD-1 plus CTLA-4 blockade. The main findings of this study were that: Tumor mutation burden (TMB) is significantly associated with improved efficacy of combination immunotherapy: TMB was higher in patients with objective response compared with those with no response; computational predicted neoantigen burden correlates with response; TMB is independent of PD-L1 expression, but patients with high TMB and positive PD-L1 expression had significantly improved rates of objective responses and PFS, compared with those tumors with only one or neither variable [300]. Importantly, the low response rate in TMB low suggests that combination immunotherapies do not overcome the negative predictive impact of low TMB; furthermore, TMB is predictive marker of response to immunotherapy much more that tumor PD-L1 expression [300]. 

The clinical activity of checkpoint blockade immunotherapies has been correlated with the activated T-cell recognition of neoantigens, which are tumor-specific antigens, mutated peptides presented by tumor cells on their surface. In order to trigger an immune response, neoantigens must be recognized by and must activate immune cells. Two main factors determine the fitness of neoantigens: the like hood of neoantigen presentation by the major histocompatibility complex and subsequent recognition by T cells [301].

The addition of chemotherapy to surgery as adjuvant or neoadjuvant treatment can improve survival rates of no more than 5% at 5 years. This finding underlines the need to define more efficacious treatments to be combined with surgery for NSCLC patients amenable to surgical resection of their tumors. Immunotherapy with immune checkpoint inhibitors is an obvious candidate for such an association in view of the efficacy demonstrated by these drugs for some NSCLC patients. These findings have supported the idea of designing clinical trials incorporating immunotherapy in earlier stages of lung cancer development. This strategy is supported by recent studies analyzing the immune microenvironment of lung adenocarcinomas compared to normal lungs and peripheral blood using paired single-cell analysis for 32 patients undergoing surgical resection for early stage tumors [302]. The data supported that tumors early developed an immunosuppressive microenvironment, as evidenced on the basis of various findings; (a) strongly reduced CD8^+^ T effector/Treg ratio compared to normal lung; (b) expansion of CD39^hi^CD38^hi^PD-1^hi^CTLA4^hi^Foxp3^hi^ Tregs at the tumor site; (c) strong reduction in the tumor microenvironment of CD16^+^NKcells, with reduced content of granzyme B; (d) depletion of CD141^+^ dendritic cells and enrichment in PPARγ^hi^ macrophages; (e) reduction of CD16^+^ monocytes, but not of CD14^+^ monocytes at the tumor site [302]. The identification of significant immunosuppressive changes in early lung adenocarcinoma lesions suggests that adjuvant or neo-adjuvant immunotherapy strategies tailored to restore immune changes occurring in the tumor microenvironment and to improve tumor response to immune checkpoint blockade [302]. 

Another important advantage of immunotherapy in early stages of tumor development could consist in generating a long-lasting anti-tumor immunity able to block micrometastatic disease, preventing tumor progression and relapse.

Numerous clinical trials involving the use of immune checkpoint inhibitors in NSCLC in a neoadjuvant or adjuvant setting at early stage of tumor development are underway [303]. Among the neoadjuvant studies, promising are the preliminary results observed in the ongoing NCT 02259621 and NCT 02927301 clinical trials. The first study evaluated the PD-1 inhibitor nivolumab for patients with early stage NSCLC and preliminary results presented at the 2017 ASCO Meeting showed that 43% of treated patients displayed a major pathological response, correlating with tumor mutation burden pre-treatment and associated with the influx of PD-1^+^ CD8^+^ T lymphocytes into responding tumors [304]. The results of this study were recently published showing that: the administration of Nivolumab in the neoadjuvant setting was safe, with an acceptable side-effect and was not associated with dalay in surgery; of the 21 tumors that were removed, 20 tumors were completely resected, showing a major response in 45% of tumors, with responses occurring in both PD-L1-positive and PD-L1 negative tumors; there was a significant correlation between the pathological response and the pre-treatment tumor mutational burden; treatment induced expansion of mutation-associated, neoantigen-specific T-cell clones in peripheral blood [305]. The follow-up of these patients is too short to have a conclusive view on the clinical efficacy and benefit of this treatment. However, it is important to point out that the extent of response to neoadjuvant chemotherapy at the time of surgical resection may be of prognostic value. In studies of neoadjuvant chemotherapy for NSCLC, tumors that had a major pathological response to therapy were associated with long-term survival [306,307]. Long-term follow-up of this study and of similar studies will be necessary to define the role of neoadjuvant PD-1 blockade in reducing recurrences and, eventually, curing early-treated NSCLCs.

The multicenter open-label, single-arm, phase II LCMC3 trial conducted by the Lung Cancer Mutation Consortium is evaluating the safety and efficacy of neoadjuvant and adjuvant treatment with atezolizumab in patients with resectable NSCLC. Neodjuvant therapy will consist of two 21-days cycles of atezolizumab and, following tumor resection, patients demonstrating clinical benefit with neoadjuvant therapy with atezolizumab for up to 12 months. The primary endpoint of the study consists in the evaluation of the major pathologic response. A similarly designed multicenter, phase II trial is being conducted with pembrolizumab; for patients with stage IB to IIIA NSCLC, 2 doses of neoadjuvant pembrolizumab and four doses administered as adjuvant therapy will be evaluated.

Recent preclinical data have suggested a rationale for combining radiotherapy with immunotherapy, basically showing that the combination of the two treatments is more efficacious than radiotherapy alone. Preclinical studies have provided evidence that PD-L1 expression on tumor cells is upregulated by radiotherapy, resulting in an enhanced antitumor effect of radiotherapy combined with PD-1/PD-L1 blockade [308]. Studies in models of NSCLC have shown that radiotherapy stimulates PD-L1 expression on *KRAS*-mutant tumor cells and synergizes with anti-PD-1 agent to induce regression of *KRAS*-driven genetically engineered mouse models of NSCLC [309]. Other studies by Gong and coworkers have shown that conventionally fractionated radiotherapy in combination with anti-PD-L1 antibody shows a synergistic anti-tumor effect in a mouse model of NSCLC [310]. Furthermore, down-regulating PD-L1 expression on lung tumor cells could alleviate resistance by promoting apoptosis. Interestingly, patients with negative PD-L1 expression had a significantly higher objective response rate that those with positive PD-L1 expression [310]. Similarly, Tokito et al. showed that in patients with stage II NSCLC treated with concurrent chemo-radiotherapy patients with positive PD-L1 expression tended to have poorer overall survival [311]. Interestingly, a recent study provided evidence that the combination of PD-L1 expression with CD8 TILs density had a strong prognostic impact with PD-L1^+^ CD8^low^ patients having the poorest prognosis [312]. Other experimental studies support the combination of radiotherapy with immunotherapy. In fact, Dovedi and coworkers have shown that fractionated radiotherapy leads to T-cell infiltration, dominated by expansion of preexisting T-cell clones; adoptive resistance via PD-1/PD-L1 pathway restricts the generation of systemic anticancer immunity following radiotherapy and can be overcome through combination with anti-PD-1 administration [313].

Several ongoing clinical trials are attempting to evaluate the safety and efficacy profiles of the combination radiotherapy and anti-PD-1/PD-L1 treatment. Interestingly, secondary analysis of the phase I KEYNOTE-001 study found that patients who previously received radiotherapy for NSCLC had significantly longer PFS and OS with pembrolizumab treatment versus patients who did not receive radiotherapy [314]. Furthermore, as above mentioned, Antonia and coworkers reported a significant prolongation of PFS in stage III, NSCLC patients receiving darvulumab after chemoradiotherapy treatment, compared to those receiving a placebo [311].

Recently, the results of a phase I study involving radiotherapy and pembrolizumab administration were reported [315]. NSCLC patients progressing on standard therapy received stereotactic body radiotherapy (SBRT) to two to four metastases and within 7 days from radiotherapy, pembrolizumab [315]. The results of the study showed that multisite SBRT in combination with pembrolizumab was well tolerated, with a median OS of 9.6 months [315].

Some studies have explored the quality of life of NSCLC patients with advanced disease either undergoing chemotherapy or immunotherapy treatment. In the phase 3 KEYNOTE-024 trial, treatment with pembrolizumab elicited longer PFS than did platinum-based chemotherapy in patients with treatment-naïve, advanced non-small-cell lung cancer with a PD-L1 tumor proportion score of ≥50%. Pembrolizumab administration improved or at least maintained health-related quality-of-life compared with that for chemotherapy [283].

In addition to the studies performed combining immunotherapy with chemotherapy or radiotherapy, other studies have attempted to combine immune checkpoint inhibitors with other types of immunotherapeutic agents, such as IL-2 or IL-15, that act via a different mechanism of action, involving activation of immune anti-tumor effector functions. Both IL-2 and IL-15 based treatments target the shared IL-2Rβγ pathway in lymphocytes. Particularly, IL-15 is a cytokine that primarily stimulates the proliferation and cytotoxic functions of CD8 T lymphocytes and NK cells, resulting in enhanced anti-tumor responses. Preclinical studies have shown that combining IL-2 or IL-15Rβγ agonists with anti-PD-1 monoclonal antibodies can improve anti-tumor responses. Given this background, a phase I clinical study evaluated the safety, tolerability, and activity of ALT-803, and OIL-15 superagonist, in combination with nivolumab in stage IIIB or IV NSCLC patients [316]. This study showed that the toxicity profile of this drug combination is acceptable and that the recommended dose of ALT-803 for phase II studies was 20 μg/Kg once per week [316]. 

In parallel to the studies of immunotherapy on NSCLC, other studies have evaluated immune checkpoint inhibitors in SCLC. The initial studies of immune checkpoint inhibition in SCLC using ipilimumab, in combination with chemotherapy was ineffective in the frontline therapy setting [317]. However, some therapeutic effects were observed using the PD-1 targeting alone or in combination with ipilimumab in SCLC patients who have progressed following standard chemotherapy. The phase I/II Check Mate 032 trial showed a rate of objective response of 10% in patients treated with nivolumab alone and 19−33% of objective responses in patients treated with nivolumab and ipilimumab (at various doses) [318]. Updated results of the Check Mate 032 trial, including data from randomized cohorts, confirmed a higher rate of responses among patients treated with the immunotherapy combination (22%), compared to those treated with nivolumab alone (11%) [319]. Whole exome sequencing was used to evaluate the impact of of tumor mutational burden on efficacy of nivolumab monotherapy or combined with ipilimumab in patients with SCLC from the randomized and nonrandomized cohorts of CheckMate 032 [320]. Within each treatment group, subdivided according to tumor burden, the estimated 1-year overall survival rate was higher in the high tumor tumor mutational burden group (35% and 62% for nivolumab monotherapy and nivolumab plus ipilimumab, respectively) than in the low (22% and 23%, respectively) or medium (26% and 20%, respectively) tumor mutational burden groups [320]. In an independent cohort of patients with SCLC who did not receive immunotherapy there was no prognostic difference in survival based on differences in the tumor burden [320].

Pembrolizumab was evaluated in the phase I study KEYNOTE 028, showing a response rate of 33% in previously treated SCLC patients, with median duration of response of 19.4 months and 1-year survival of 37.7%; 8.3% of the patients participating to this study had grade 3-4 adverse events [321,322]. Immune checkpoint inhibitors are also being evaluated as maintenance therapy in SCLC. In this context, a multicenter phase II trial of maintenance therapy failed to show any significant improvement of PFS when pembrolizumab was administered after chemotherapy, as a maintenance treatment [323]. Nivolumab alone and in combination with ipilimumab are currently under evaluation as maintenance therapy after standard therapy.

## 13. Conclusions

Lung cancer is the most common cause of cancer-related death in the world, with an estimated 1.6 million deaths each year. About 85% of patients have a group of histological types regrouped as NSCLC and including lung adenocarcinoma and LSQCC. The most common etiological factor involved in lung cancer development is tobacco smoking, accounting for about 80% of cases, particularly for LSQCC and SCLC. A part of lung cancers is observed in never smokers. Thus, the eradication of the use of all tobacco-related products represents a main and fundamental goal in the fight against cancer, but represents an objective difficult to reach for numerous problems. Thus, the war against lung cancer requires not only tobacco prevention strategies, but also an improvement of the current therapies.

The improvement of the therapies of lung cancers necessarily involves a better understanding of the molecular defects underlying these tumors. In the last two decades, tremendous progresses have been made in the understanding of the molecular abnormalities occurring in various lung cancer subtypes, offering the opportunity to better understand the pathogenesis of these diseases, providing also in some cases the possibility to identify potential therapeutic targets.

The most commonly mutated genes in in lung adenocarcinoma include *KRAS* and *EGFR* and the tumor suppressor genes *TP53*, *KEAP1*, *STK11* and *NF1*. *KRAS* and *EGFR* mutations are usually mutually exclusive, but when they co-exist, *KRAS* mutations confer resistance to EGFR inhibitors. Lung adenocarcinomas harboring oncogenic driver mutations at the level of *EGFR* and *ROS1* and *ALK* rearrangements have a lower mutational load, often occur in never smokers and are sensitive to targeting with specific inhibitors and this offered new therapeutic perspectives for these patients. *KRAS* and *EGFR* mutations are usually present in the founder clones of lung adenocarcinoma, while *TP53* mutations are frequently acquired during advanced stages of tumor development, thus suggesting a role during tumor progression [324]. Lung adenocarcinomas bearing *ERBB2*/*3* alterations, *ROS* mutations and *KEAP1* inactivation are also susceptible of targeting therapies.

LSQCCs are characterized by frequent mutations of *TP53* and *CDKN2A* alterations; *EGFR* alterations are less frequent in LSCCC than in lung adenocarcinoma and actionable mutations in receptor tyrosine kinases are rarely observed in LSQCC. 

The genomic landscape of lung cancers is markedly different between never smokers and smokers, with: the non-smokers showing a lower tumor mutational burden, a predominant transition of cytosine to thymine (C > T) and a higher frequency of actionable driving gene alterations, such as *EGFR* mutations and *ALK* and *ROS1* fusions; the smokers exhibiting a clearly higher mutation burden, predominantly cytosine to adenine (C > A) nucleotide transversions and non-actionable mutations, such as *KRAS* and *TP53* mutations.

Considerable progresses have been achieved in the therapy of NSCLCs in the last three decades. Surgical resection is the optimal therapy for patients with stage I to II and for a part of patients with stage IIIA disease; however, a part of these patients relapses after surgery and adjuvant chemotherapy with a cisplatin-based doublet improves the survival of patients with stage II and IIIA tumors completely resected. For patients with advanced stage and good performance status the platinum-based doublet therapy represents the standard therapy; patients achieving disease control after 4–6 cycles of therapy have the additional option of a maintenance therapy. Finally, for patients with unresectable locally advanced NSCLC, the standard therapy consists in the combination of chemotherapy with thoracic irradiation. However, in spite these treatments, a high mortality rate is observed among patients with advanced stage disease due to the presence of metastatic disease at diagnosis.

However, starting from the late 1990s, targeted therapies have been introduced in the treatment of some subtypes of NSCLCs and, particularly, of lung adenocarcinomas. 

**Figure 5:**
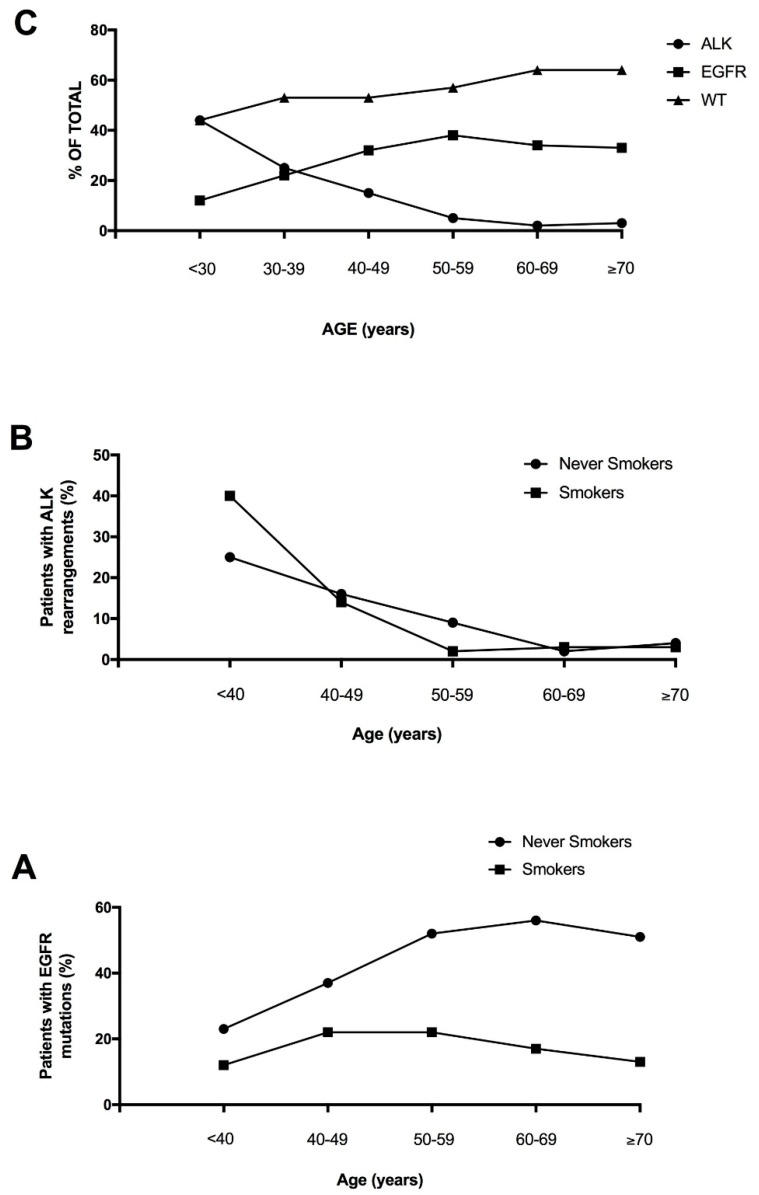
(**A**) Age distribution at diagnosis of Chinese NSCLC patients with ALK-EML4 rearrangements, EGFR mutations and wild type (without ALK or EGFR alterations) tumors; (**B**) age distribution of ALK-rearranged NSCLCs stratified by smoking status; (**C**) age distribution of EGFR-mutant NSCLCs stratified by smoking status.

The analysis of thousands lung cancer patients screened at molecular level allowed to define peculiar clinicopathologic features of some molecular subtypes of NSCLC. In this context, particularly relevant were the studies carried out in lung adenocarcinomas with EGFR mutations and ALK rearrangements. Particularly interesting were the results of a study performed by Hong and coworkers based on the screening of a total 1160 Chinese NSCLC patients: 78% lung adenocarcinomas, 54% never-smokers, 33.8% with EGFR mutations and 8.1% with EML4-ALK rearrangements [325]. The study of these patients showed that: (i) ALK-rearrangement was strongly associated with younger age at diagnosis (i.e., the frequency of ALK-rearrangements in NSCLC occurring before 40 years was very high (25−50%), while it was markedly lower in patients after 50 years (2−8%)); (ii) EGFR mutations were clearly associated with lower tobacco exposure and moderate to high differentiation [325] (Figure 5).

The first targeted therapy was developed for the treatment of EGFR-mutated lung adenocarcinomas. The most common mutations include exon 19 deletions and a missense mutation on exon 21 (L858R); these mutations confer sensitivity to EGFR TKIs. In order to inhibit the growth of EGFR-mutant lung adenocarcinomas, three generations of EGFR TKIs have been developed. First-generation EGFR TKIs, reversible, competitive ATP inhibitors targeting only EGFR, such as erlotinib and gefitinib, have shown higher response rates and improved PFS, compared to standard chemotherapy in previously untreated patients with *EGFR* mutations. However, patients treated with these inhibitors ineluctably relapse, most frequently due to a second *EGFR* mutation in exon 20 (T790M). To bypass these limitations, a second generation of EGFR TKIs was developed, mainly represented by Afatinib and Dacomitinib, acting as irreversible EGFR TKIs, targeting also HER2 and HER4. Both Afatinib and Dacomitinib showed an increased PFS compared to a first-generation EGFR TKI, such as gefitinib [21,326,327]. On the other hand, it was clearly demonstrated that the administration of gefitinib was detrimental to NSCLC patients with *EGFR*-mutant tumors with disease progression; in these patients, treatment with the first-generation EGFR TKI must be discontinued [328].

Third generation EGFR TKIs, such as osimertinib, are selective inhibitors of both the original sensitizing and T790M mutations but spar the normal EGFR. Osimertinib had significantly greater efficacy than platinum therapy plus pemetrexed in lung adenocarcinoma with T790M-positive advanced NSCLC in whom disease had progressed during first-line EGFR-TKI therapy [329]. Importantly, in a randomized trial comparing Osimertinib to first generation EGFR TKIs (erlotinib or gefitinib) in previously untreated NSCLC patients with *EGFR*-mutant tumors, osimertinib administration was associated with a significant improvement of PFS (17 vs. 8 months) and a trend towards an improved survival (after 18 months, 83% in the osimertinib arm, compared to 71% in standard EGF-TKIs arm) [330]. This important study represents the basis for establishing osimertinib as a first line EGFR TKI option. More mature data on the overall survival rate derived from this study are greatly waited. Another recent study performed on *EGFR*-mutant NSCLC patients treated in first-line with osimertinib confirmed a high-rate of response to treatment: in the group of patients treated with 160 mg osimertinib, the ORR was 87%, with a PFS of 19.3 months [331]. In this study, an attempt was made to try to understand the mechanisms of resistance to osimertinib in patients with disease progression, investigating circulating tumor DNA: 50% of these patients had no-detectable ctDNA and in the remaining patients the molecular mechanisms of tumor resistance were heterogeneous (*MET* amplification, *EGFR* and *KRAS* amplification, *MEK1*, *KRAS* or *PI3KC* mutations, *EGFR* C797S mutations, *JAK2* mutations, *HER2* exon 20 mutation); importantly, there was no evidence of acquired *EGFR* T790M mutation in progressing patients treated with osimertinib [331]. In the context of the clinical study AURA, it was evaluated the clinical efficacy of osimertinib on a group of NSCLC patients experimenting disease progression after one or more treatments with first and second- generation EGFR TKIs, related to the development of Thr790Met mutation; median PFS was 11.1 months and median OS was 16.9 months [332]. These patients were evaluated by analysis of plasma tumoral DNA: patients without detectable *EGFR*-activating mutations in plasma before treatment had the best overall and post-progression survival; loss of the Thr790Met mutation, but presence of EGFR-activating mutations in plasma were associated with the shortest progression-free survival; Thr790Met mutations were observed in 50% of cases, Cys797Ser in 17% of cases, *c-MET* amplification in 50% of cases, *BRAF* mutation in 8% and *KRAS* mutation in 8% of cases [332].

Interestingly, the Chinese patients show an increased incidence of NSCLC with EGFR mutations compared to Caucasian patients [333]. These tumors are characterized by a mutational MS3 signature that is associated with inflammatory tumor infiltrating B lymphocytes: as a consequence, the frequency of EGFR mutations is very high among lung cancers with a MS3 signature [333]. These observations were also confirmed through the analysis of TCGA data. MS3-high NSCLCs display a high contribution of distant chromosomal rearrangements, some of which result in fusions, involving genes such as ALK and RET [333]. According to these observations, it was suggested that inflammatory infiltration may contribute to the accumulation of EGFR mutations, particularly in never-smoker patients.

As above discussed, unfortunately mechanisms of resistance develop also in response to osimertinib, such as the C797S mutation. The various combinations of *EGFR* mutants determine a spectrum of sensitivity/resistance to the various EGFR TKIs and studies are in progress to define EGFR TKIs able to inhibit NSCLCs bearing triple mutants (sensitizing mutations + T790M + C797S): these new mutants, such as EA1045, could represent the fourth-generation of EGFR TKIs [334].

The history of treatments with ALK-rearranged NSCLCs is very similar to that described for EGFR mutant patients, with progressive identification and introduction in clinical use of first, second and third-generation ALK inhibitors. The first generation ALK inhibitor crizotinib improved overall response rate and PFS compared to cytotoxic therapy in untreated and previously treated ALK-mutant NSCLCs. Unfortunately, all patients with ALK-rearranged NSCLC develop drug resistant disease, mostly due to the development /selection of new ALK mutations or ALK copy number gain. Second- and third-generation ALK inhibitors (ceritinib, alectinib, brigatinib, lorlatinib) are structurally different and characterized by a higher affinity for ALK and different selectivity for various ALK mutants. Using these inhibitors, overall response rates and progression-free survival rates range from 39% to 56% and 5.4 to 12.9 months after crizotinib treatment failure, respectively. Two randomized studies have shown that alectinib compared to crizotinib elicited increased overall response rates and PFS in patients with previously untreated ALK-mutated NSCLC, thus indicating that Alectinib could represent a first-line treatment option [335,336]. In the alectinib arm the PFS was 25.7 months, compared to 10.4 months in the crizotinib group; the progression in the CNS as the first site of disease progression was 12% in the alectinib group and 45% in the crizotinib group [335,336]. The rationale of treating ALK-rearranged NSCLC patients with ALK inhibitors is supported by data on the overall survival. In fact, patients undergoing crizotinib treatment showed an overall survival of about 16–20 months [337], while patients sequentially treated with crizotinib and ceritinib displayed a survival of 49 months [337]. Furthermore. There is evidence about an improvement of overall survival among NSCLC patients who experienced progressive disease after crizotinib treatment and who were treated with next-generation-guided ALK inhibitor [338]. The development of the technology to detect circulating tumor DNA offers the unique opportunity to detect longitudinally during patients’ treatment the mutational evolution of ALK and to serve as a guide for definition of optimal ALK inhibitor therapy [339]. Thus, the longitudinal analysis of plasma specimens from ALK-positive patients with acquired resistance to ALK TKIs allowed to track the evolution of resistance during treatment and to define an optimized treatment [339]. It is important to underline that only half of the tumors that develop resistance to secondary-generation ALK inhibitors harbor resistance mutations in *ALK*, while the other half have other mechanisms underlying resistance. A recent study showed that non-receptor protein tyrosine phosphatase SHP2 inhibitor SHP099 halted the growth of ALK-resistant NSCLC cells [340].

Importantly, two recent studies support a real impact of ALK TKIs on the overall survival of ALK-positive NSCLCs. First, the final results of the Profile 1014 study provided evidence that crizotinib treatment elicited an improvement of overall survival compared to chemotherapy in patients with advanced ALK^+^ lung adenocarcinoma [341]. The longest OS was observed in crizotinib-treated patients who received a subsequent ALK TKI [341]. Second, in the ALTA trial, patients with ALK-positive NSCLCs, relapsing after crizotinib therapy, were assigned to receive either 90 (arm A) or 180 (arm B) mg of brigantinib; about 70% of these patients displayed brain metastases: the intracranial ORR was 46% and 67% in arm A and arm B, respectively; among patients with brain metastases, PFS was 15.6 months in arm A and 18.3 months in arm B [342].

ROS1 is a receptor tyrosine kinase constitutively activated in about 2% of lung adenocarcinomas as a consequence of fusion gene events. The kinase domain of ROS1 displays a great homology with those of ALK and this explains the inhibitory activity against ROS1-positive tumors of drugs such as crizotinib, ceritinib or lorlatinib active against *ALK*-mutant tumors. All these three drugs have shown a significant anti-tumor activity in *ROS1*-mutated lung adenocarcinomas [343]. The consistent activity of crizotinib was confined also in East Asian patients with ROS1-positive advanced NSCLC, with a median progression-free survival of 15.9 months and with a duration of response of 19.7 months [344]. The main mechanisms of crizotinib resistance is related to the presence of ROS1resistance mutations, particularly of the G2031R *ROS1* mutation, activation of the wild-type EGFR signaling, *KRAS* and *KIT* mutations. 

Other potential targetable gene alterations include *BRAF*, *MET* and *HER2* mutations, rearrangements in the proto-oncogene *RET* and fusions of the neurotrophic tyrosine receptor kinase (*NTRK*) genes.

As above repeatedly reported, the molecular profile of LSQCC is complex and associated with a high mutational load; the genetic profile of LSQCC is unlikely to offer many actionable molecular targets, since the main dominant molecular changes are not addictive oncogenes. The lack of identifiable oncogenic drivers in LSQCC represents a great limitation and targeting single genetic alterations seems to achieve only modest clinical benefits in advanced LSQCC. Given these consistent difficulties, novel study designs have been developed aiming to discover and evaluate additional potential target treatments for LSQCC. In this context, some protocols have been developed, such as the Lung Cancer Master Protocol (LUNG-MAP) study (SWOG S1400) aiming to identify potentially actionable molecular alterations in advanced LSQCC through the broad and comprehensive screening of patients through a next generation sequencing platform, capable of detecting base substitutions, short insertions and deletions, copy number alterations and gene fusions [345]. This approach enables the patients to be pre-screened with molecular testing prior to disease progression during first-line therapy, allowing the efficient assignment of eligible patients to a specific substudy, based on biomarker positivity [345]. However, three phase II studies performed by LUNG-MAP in advanced LSQCC, that included the FGFR inhibitor AZD4547, the cyclin-dependent kinase 4/6 inhibitor palbociclic, and the PI3K inhibitor taselisib failed to meet their primary end points in their respective biomarker-enriched cohorts [346,347,348].

In spite the lack of results until now of targeted therapy in LSQCC, recent studies have provided evidence that a part of these patients may have some benefit from immunotherapy. Interestingly, the immunotherapy studies have shown similar therapeutic benefits for LSQCC and lung adenocarcinoma patients. The anti-PD1 agent pembrolizumab was approved for first-line treatment of patients with advanced NSCLC, including LSQCC, in patients with high PD-L1 expression (tumor proportion score 50%). Second-line treatment of patients with advanced NSCLC, including LSQCC, with anti-PD1 agents pembrolizumab and nivolumab and anti-PD-L1 agent atezolizumab prolonged survival compared to standard chemotherapy. Importantly, the proportion of patients surviving after 5 years if nivolumab treatment were equally represented among LSQCCs and lung adenocarcinomas [278]. The best two biomarkers to predict the sensitivity of NSCLC patients to immunotherapy with immune check inhibitors based on the evaluation of PD-L1 expression and tumor mutational load. Thus, it is not surprising that PD-L1 testing at diagnosis for metastatic NSCLC disease has been included into guidelines, such as NCNN guidelines [349] or ASCO guidelines [350].

Given the high number of lung cancer patients treated with immune check inhibitors, it was possible to perform meta-analysis studies to evaluate the role of some biological parameters influencing the therapeutic response to these agents. A recent study investigated whether the patients’ sex influences cancer immunotherapy efficacy, showing that both for NSCLC patients and melanoma patients the relative reduction of the risk of death that is achievable with immune check inhibitors, compared with standard therapies is significantly higher in male patients than in female patients [351]. According to the findings of this study, it was suggested that a patient’s sex should be considered in the assessment of risk versus benefit when making decisions about treatment strategies.

While the large majority of studies explored immunotherapy with immune check inhibitors in the adjuvant setting, in patients with unresectable lung cancers, a recent study initially explored a possible role of immunotherapy in patients with resectable NSCLC in the neoadjuvant setting [305]. In this study, the patients were allocated to receive two doses on the anti-PD1 antibody nivolumab about 4 weeks before resection of stage I, II or IIIA NSCLC: the immunotherapy treatment did not delay surgery and of the 21 primary tumors removed, 20 were completely resected [310]. The anatomopathological analysis of the resected tumors showed that 45% of patients had a major pathological response occurring irrespective of the tumor PD-L1 status, but directly correlated with the pretreatment tumor burden [305]. These observations support the feasibility of the immunotherapy also in the neoadjuvant setting and suggest a promising therapeutic activity, to be evaluated in dedicated clinical trials.

The rapid development of targeted therapy for a part of patients with NSCLC had supported a redefinition of the guidelines concerning the definition of molecular analyses of lung cancer samples and the definition of the optimal treatment options for NSCLC patients with advanced disease. The guidelines on molecular testing indicate which lung cancer patients and samples should be tested, which genes should be tested, and how these tests should be designed, performed and validated [352,353]. Following these guidelines, the genes whose alterations have to be tested have been grouped into three different categories: (a) a set of genes that must be offered by all laboratories, that test lung cancers, including EGFR, ALK and ROS1; (b) a second set of genes that should be included in any expanded set of genes, including BRAF, MET, RET, ERBB2 and KRAS; (c) KRAS testing may be performed also as a single-gene test to exclude patients from expanded panel testing [352,353]_._

The development of immunomodulatory therapies based on immune check inhibitors requires the investigation of some biomarkers whose positivity correlates with a major probability of response: PD-L1 expression on tumor cells assessed by immunohistochemistry and the evaluation of tumor mutational load. Furthermore, some molecular markers predict a low response to immune check inhibitors. Thus, recent clinical studies have shown that LKB1 genetic alterations are associated with lack fo benefit from PD-1 blockade (7% overall response) in KRAS-mutant lung adenocarcinomas, a phenomenon that cannot be related to *KRAS* mutations in that *KRAS*-mutant lung adenocarcinomas without LKB1 mutations are responsive (about 35% of overall responses) to PD-1 blocking [354].

In parallel, evidence-based recommendations for the treatment of advanced of advanced NSCL have been updated and recently proposed [350,355]. The first-line therapeutic recommendations for patients with lung adenocarcinoma without an EGFR-sensitizing mutation or ALK or ROS1 gene rearrangement with a good performance status are the following: (a) with high PD-L1 expression and non-contraindications, single-agent pembrolizumab is recommended; (b) with low PD-L1 expression, a variety of combination cytotoxic chemotherapies (platinum doublet with pemetrexed ± bacivuzumab or carboplatin/pemetreexed/pembrolizumab). For patients with LSQCC without EGFR, ALK or ROS1 alterations the first-line therapeutic recommendations are the following: (a) with high PD-L1 expression single-agent pembrolizumab is recommended; (b) with low PD-L1 expression, a variety of combination cytotoxic chemotherapies are recommended (platinum doublet). The second-line therapeutic treatment for lung adenocarcinoma patients without a tumor with EGFR, ALK and ROS1 alterations implies: (a) in both patients with high PD-L1 expression who received first-line chemotherapy and have not received prior immunotherapy and patients with low or unknown tumor PD-L1 expression who received first-line chemotherapy, single-agent nivolumab, atezolizumab or pembrolizumab is recommended; (b) in patients who received an immune checkpoint inhibitor as first-line therapy, a variety of cytotoxic chemotherapies are recommended; (c) in patients who received cytotoxic chemotherapy and pembrolizumab as first-line treatment, docetaxel ± ramicirumab or gemcitabine is recommended. The same applies to patients with LSQCC without EGFR, ALK or ROS1 alterations for the second-line treatment. For both lung adenocarcinoma and LSQCC patients, the third-line treatments usually imply chemotherapy regimen based on docetaxel ± gemcitabine.

In conclusion, the treatment of NSCLC has undergone dramatic changes in the past 10 years, as a result of the better understanding of lung cancer heterogeneity and of the molecular abnormalities underlying this heterogeneity, with subsequent and consequent development of targeted therapies that started the era of personalized medicine and of immunotherapies. The development of these therapies represents the basis for the hope for prolonged survival in a part of patients with NSCLC, raising this this possibility of an efficacious therapy also for patients with metastatic disease. Nertheless, this improvement in therapy outcome is still limited to a minority of patients, with targeted therapies being limited only to adenocarcinoma patients and durable responses to immunotherapy occurring only in a minority of patients. Future studies have to try to: (i) to improve the number of lung cancer patients responding to targeted therapies or immunotherapies, particularly among SCLC and LSQCC patients, through the identification of appropriate tumor-specific targets and biomarkers; (ii) to better understand the mechanisms of acquired resistance to targeted therapies to block/reduce their occurrence or to develop an effective treatment at the time of their emergence; (iii) to better define the biomarkers predicting the response to immunotherapies. 

## Figures and Tables

**Table 1:** Classification of the main types of lung cancers following WHO criteria.

**Adenocarcinoma (Early Lesions)**	Adenocarcinoma in situ; Minimal Invasive Carcinoma (<0.5 cm); Atypical Adenomatous Hyperplasia; Adenocarcinoma in situ
**Advanced Adenocarcinoma**	Lepidic Adenocarcinoma; Acinar Adenocarcinoma; Papillary Adenocarcinoma; Micropapillary Adenocarcinoma
**Variants of Adenocarcinoma**	Invasive mucinous adenocarcinoma (mixed non-mucinous and mucinous Adenocarcinoma); Colloid Adenocarcinoma; Fetal Adenocarcinoma; Enteric Adenocarcinoma
**Squamous Cell Carcinoma** **Early Lesions**	Preinvasove Lersion; Squamous cell carcinoma in situ
**Squamous Cell Carcinoma** **Advanced Tumors**	Keratinizing; Non-Keratinizing; Basaloid Squamous Cell Carcinoma
**Neuroendocrine Tumors**	Small Cell Carcinoma; Large Cell Neuroendocrine Carcinoma; Carcinoid Tumor; Diffuse Idiopathic Pulmonary Neuroendocrine Cell Hyperplasia
**Large Cell Carcinoma**	
**Adenosquamous Carcinoma**	
**Sarcomatoid Carcinoma**	Pleomorphic, Spindle Cell, and Giant Cell Carcinoma; Carcinosarcoma; Pulmonary Blastoma
**Salivary gland-type Carcinoma**	Mucoepidermoid Carcinoma; Adenoid Cystic Carcinoma; Epithelial-Myoepithelial Carcinoma

**Table 2:** Classification of tumor, node and metastasis of lung cancers.

**T:Tumor**	**Tumor Characteristics**
T0	Absence of primary tumor
Tis	Carcinoma in situ
T1	Tumor ≤ 3 cm in greatest diameter
T1a	Tumor ≤ 2 cm in greatest diameter
T1b	Tumor ≥ 2 cm but ≤ 3 cm in greatest diameter
T2	Tumor ≥ 3 cm but ≤ 7 cm in greatest diameter
T2a	Tumor ≥ 3 cm but ≤ 5 cm in greatest diameter
T2b	Tumor ≥ 5 cm but ≤ 7 cm in greatest diameter
T3	Tumor >7 cm Tumor invading chest wall, diaphragm, phrenic nerve, mediastinal pleura, pericardium Tumor in the main bronchus Separate tumor nodules in in the same lobe
T4	Tumor of any size invading heart, great vessels, mediastinum, trachea, recurrent laryngeal nerve, esophagus, vertebral body, carina
**N:Nodes**	
N0	Absence of regional lymph node metastasis
N1	Metastasis in ipsilateral hilar and/or peribronchial lymph nodes and intra-Pulmonary lymph nodes
N2	Metastasis in ipsilateral mediastinal and/or subcarinal lymph nodes
N3	Metastasis in contralateral hilar or mediastinal lymph nodes, in ipsilateral or contralateral scalene lymph nodes, or in supraclavicular lymph nodes.
**M:Metastases**	
M0	Absence of distant metastasis
M1	Distant metastasis
M1a	One or more separate tumor nodules in contralateral lobe Tumor with pleural nodules or tumor pleural/pericardial effusion
M1b	Distant metastasis

**Table 3:** Druggable mutations mainly observed in lung adenocarcinomas (LUAD).

Gene	Alteration	Frequency in Lung Cancer	Targeted Therapy
EGFR	Exo 19 deletion L858R point mutation in exon 21 L861Q point mutation in exon 21 G719X point mutation in exon 18 T790M point mutation exon 20	NSCLC: ≅15% in Western populations NSCLC: ≅35−50% in Asian populations LUAD: 27% LSQCC: <9% LUAD never smoker: 42% LUAD former smoker: 13.5% LUAD current smoker: 5%	Gefitinib (EGFR del 19 or L858R mutation) Erlotinib (EGFR del 19 or L858R mutation) Afatinib (EGFR del 19 or L858R mutation) Dacomitinib (EGFR del 19 or L858R mutation and T790M mutation) Osimertinib (EGFR T790M mutation)
ALK	ALK rearrangement EML4-ALK (the most frequent) KIF5B-ALK KLC1-ALK	NSCLC: 3−5% LUAD: 3−7% LSQCC: 0.2−1% LUAD never smoker: 5−11% LUAD smoker: 0–0.8%	Crizotinib Ceritinib Alectinib Brigantinib Lorlatinib (active also against the resistance mutation G1202R)
ROS1	ROS1 rearrangement CD74-ROS1 SDC4-ROS1 SLC34A2-ROS1 EZR-ROS1	NSCLC: 0.5−2% LUAD: 2.6% LSQCC: 0% LUAD never smoker: 5.8% LUAD smoker: 0.4%	Crizotinib Ceritinib Brigatinib (it inhibits the resistance mutation L2026M, but not D2033N and L1951R) Lorlatinib (partially active against G2032R resistance mutation) Entrectinib Cabozantinib (active against G2032R, but with an elevated profile of toxicity)
RET	RET rearrangement	NSCLC: 1−2% LUAD: 1.4% LSQCC: 0% LUAD never-smoker: 1.8% LUAD smoker: 0.7%	Vandetinibv Cabozantinib Lenvatinib BLU-667
BRAF	Point mutations BRAF^V600E^ (30−40% of cases) BRAF^nonV600E^ (60−70% of cases): BRAF^V469A^, BRAF^D594G^, BRAF^G466A^ Class I, II and III BRAF mutants	NSCLC: 2.6% LUAD: 1.4% LSQCC: 0.2−1% LUAD never-smoker: 5−11% LUAD smoker: 0–0.8%	Dabrafenib (BRAF inhibitor) + Tramafenib (MEK inhibitor): BRAF^V600E^–mutant NSCLC
HER2	Point mutations HER2 amplifications	NSCLC: 1.8% LUYAD: 2.3% LSQCC: 0% LUAD never smoker: 4.5% LUAD smoker: 0.8%	Trastuzumab Afatinib Ado-trastuzumab emtansine
MET	MET mutations (exon 14 skipping mutations) MET amplifications MET translocations	Met mutations, METex 14 NSCLC: 2.5−3% LUAD: 3% LSQCC: 0% LUAD never smoker: 4% LUAD smoker: 2.2%	Capmatinib Tepotinib Crizotinib Cabozantinib
LKB1/STK11	LKB1 mutations LKB1 homozygous deletion	LUAD: LKB1 mutations 8% LUAD: LKB1 homozygous deletion 30%	Loss of LKB1 expression caused by mutation or gene deletion is associated with resistance to immune check inhibitors
KEAP1	Point mutation	LUAD: 15% LSQCC: 14% LUAD never smoker: 7% LUAD previous smoker: 18% LUAD current smoker: 27%	Clabetasol propionate SW 157765
NFE2L2	Point mutation	LUAD: 2% LSQCC: 14.5% LUAD never smoker: 3.5% LUAD previous smoker: 4% LUAD current smoker: 9.5%	Clabetasol propionate SW 157765

## Supplementary Materials

The following are available online at http://www.mdpi.com/2072-6694/10/8/248/s1.
