# Supplementary material for: Lung Cancers: Molecular Characterization, Clonal Heterogeneity and Evolution, and Cancer Stem Cells
Source: Cancers (Basel). 2018 Jul 27;10(8):248. doi: 10.3390/cancers10080248 (PMC6116004; doi:10.3390/cancers10080248)
Supplement: Supplementary file 1 [file cancers-10-00248-s001.docx]

**Supplementary Materials: Lung Cancers: Molecular Characterization, Clonal Heterogeneity and Evolution, and Cancer Stem Cells**

Ugo Testa *, Germana Castelli and Elvira Pelosi

Department of Hematology, Oncology and Molecular Medicine, Istituto Superiore di Sanità ,00161 Rome, Italy; germana.castelli@iss.it (G.C.); elvira.pelosi@iss.it (E.P.)

***** Correspondence: ugo.testa@iss.it

1. EGFR-Mutant Lung Adenocarcinoma

Some of these mutations are clinically relevant and have been already translated into clinical applications. In this context, particularly relevant are the studies and the results obtained using EGFR TK inhibitors. Overexpression of *EGFR* and activation of a downstream signaling activation pathway are commonly observed in NSCLC. The identification of activating mutations in *EGFR*, mostly seen in exon 19 (deletion) or in exon 21 (L858R) mutation, together with an increased sensitivity to EGFR tyrosine kinase inhibitors, has been the first and most important step toward molecular-guided precision therapy of lung cancer. According to these findings two small-molecule EGFR TK inhibitors (gefitinib and erlotinib), that inhibit the binding of ATP and prevents downstream signaling have been introduced in the therapy of NSCLC patients. Using both types of inhibitors it was observed that not all patients responded to treatment: the analysis of the *EGFR* mutational status in these patients showed that patients with mutated *EGFR* responded to treatment, while *EGFR* mutations were absent among patients who do not respond to this treatment. The *EGFR* mutations observed in these responder patients are *EGFR* activating mutations that promote cell survival and occur at the level of exon 19 (deletion) or L858R point mutations [1]. Both these mutations are located in the kinase domain of the receptor leading to ligand-independent, constitutive kinase activation. The meta-analysis of various clinical studies showed that the presence of *EGFR* mutations was associated with a high response rate and with an improvement of the progression-free survival compared to that achievable with standard treatments. Higher activity of EGFR tyrosine kinase inhibitors was seen in patients with exon 19 *EGFR* mutations than in patients who had exon 21 *EGFR* mutations. None of these trials showed a clear and significant difference in overall survival. In fact, in spite impressive responses to these EGFR TKIs, the disease progresses in most patients after 9 to 12 months of treatment.

It is of interest to note that *EGFR* TK domain mutations are present more often in the tumor specimens from lifelong never-smoker than from smokers. In fact, Kerr and coworkers have analyzed 1017 lung adenocarcinomas and reported a frequency of *EGFR* mutations of 6.0% among smokers, compared with 28.4% among never smokers [2]. Similar observations have been made by Aisner and coworkers who screened 907 lung adenocarcinomas for *EGFR* mutations subdivided into s*EGFR* mutations (including L858R mutant, Exon 19 in-frame deletions and insertions, G719S/C/A and L861Q) and o*EGFR* (E709A, Exon 20 in-frame insertion or deletion, T790M): both s*EGFR* and o*EGFR* showed a gradient of positivity from current smokers to Former Smokers and to Never Smokers (2.9%, 9.9% and 28.7%, respectively; 1.0%, 1.3% and 4.8%, respectively) [3].

At the moment, three generations of EGFR TKIs have been developed. The first generation of EGFR TKIs, composed by gefitinib and erlotinib, is effective in the treatment of NSCLCs with activating *EGFR* mutations, such as deletion in 19 and exon 21 L858R mutation. The second generation of EGFR-TKIs, afatinib and dacomitinib, is able to bind irreversibly the tyrosine kinase domain of EGFR and of other ERBB-family members. The third generation of EGFR-TKIs, composed by CO-1686, HM61713 and AZD9291 (osimertinib), inhibits both activating EGFR mutations and resistance *EGFR* mutations, such as T790M [4]. Importantly the results of the clinical trials LUX-Lung3 and LUX-Lung6 suggest that afatinib significantly prolonged the overall survival of del19-positive *EGFR*-mutant NSCLC patients compared to a standard chemotherapy regimen [30]. In contrast, the effect of this drug was not significant on the survival of NSCLC patients with EGFR Leu858Arg-positive tumors [5]. Another recent clinical trial showed the improved capacity of a second-generation EGFR TKI, dacomitinib, to prolong progression-free survival compared to a first-generation inhibitor, gefitinib: PFS was 14.7 months in the dacotinib group and 9.2 months in the gefitinib group [6].

The occurrence of a secondary exon 20 T790M missense mutation is the most frequent alteration occurring in about 40% to 60% of the treated patients and is the most important mechanism from a clinical point of view for its resistance to first and second-generation EGFR TKIs. Osimertinib is a third-generation, irreversible EGFR TKI able to target both primary activating *EGFR* mutations and the secondary T790M mutation; osimertinib showed a response rate of 61%, with a PFS of 9.6 months, in lung cancer patients with T790M mutations, whose disease progressed during treatment with an EGFR TKI [7]. More recently, the efficacy of osimertinib was evaluated in two randomized clinical trials: in one of these studies, AURA3, osimertinib was compared with standard chemotherapy in 419 NSCLC patients pretreated, with T790M mutations and it was provided evidence that osimertinib prolonged PFS from 4.4 to 10.1 months and increased the response rate from 31 to 71% [8]; an extension of the results of the AURA study showed that the median duration of response in 122 patients responding to the treatment with osimertinib was 15.2 months, indicating that this drug treatment may induce durable responses [9]; in the other study, FLAURA, 556 patients with previously untreated EGFR-mutation-positive NSCLC were randomly assigned either to osimertinib or gefitinib therapy: PFS was 18.9 months witho vs 10.2 months with gefitinib, with a duration of response of 17.2 months vs 8.5 months and with a survival rate at 18 months of 83% vs 71% [10]. This study strongly supports the use of a third-generation EGFR-TKI as a first-line therapy; it would be extremely important to have an evaluation of the effect on overall survival of this type of EGFR TKI at a longer time of follow-up. Unfortunately, also following treatment with osimertinib, some patients develop acquired resistance to this TKI, through the emergence of inhibitor-resistant tumor cell clones bearing *EGFR* C797S or G722S mutations [11].

Some studies explored the therapeutic effect of EGFR TKIs in the adjuvant setting. Thus, Zhong and coworkers have recently reported the results of the ADJUVANT randomized study, involving 222 patients with completely resected, stage II-IIIA *EGFR*-mutant lung adenocarcinoma: the patients enrolled in this study were randomly assigned to either four cycles of adjuvant chemotherapy (vinarelbine plus cisplatin) or 24 months of gefitinib. Disease-free survival was significantly longer for patients assigned gefitinib than for those assigned chemotherapy (28.7 months vs 18.0 months) [12]. However, after 30 months the benefit deriving from gefinitib is considerably reduced and overall survival data are not mature for evaluation [12]. Interestingly, similar conclusions were also reached in a subgroup of 161 patients with *EGFR*-mutant disease identified retrospectively in the RADIANT trial in which stage IB-IIIA disease were randomly assigned to erlotinib or placebo for 24 months after adjuvant chemotherapy; disease-free survival was longer with EGFR inhibitor (46.4 months vs 28.5 months), but the survival curves start to converge after 36 months and, after 48 months meet [13]. The role of EGFR-TKIs in adjuvant therapy of *EGFR*-mutant lung adenocarcinomas will require additional studies and, particularly, the evaluation of the effects on overall survival. However, these studies establish an important initial point for future studies, eventually implemented through the monitoring of the residual disease through analysis of circulating tumor DNA. In this context, it is interesting to note that in a study on 40 lung cancer patients with stage I-III disease, 93% of patients negative for circulating tumor DNA before treatment and four months after treatment remained free from progression at 36 months vs 0% among patients with detectable circulating tumor DNA [14].

*EGFR* mutations are heterogeneous since in addition to the most common mutations, other mutations have been described, either involving an EGFR kinase domain duplication [15] or a fusion of *EGFR* with other genes, such as RAD51 [16]. These findings further support the need of a careful molecular characterization of the *EGFR* mutational status before to start a therapy with an EGFR TKI.

A subset of patients with lung adenocarcinoma, corresponding to about 2.5% to 4.5%, carry germline variants that have been linked to cancer risk in Mendelian syndromes. These genes include *ATM, TP53, BRCA2, EGFR* and *PARK2* [17]. Interestingly, some families with multiple cases of NSCLC associated with a germline transmission of the T790M mutation have been described; these observations have implicated altered EGFR signaling in genetic susceptibility to lung cancer [18]. In a recent screening on >31,000 patients, germline *EGFR* T790M mutations were detected in 48 cases, 43 being adenocarcinomas [19]. A trial is evaluating the possible impact of germline T790M mutations on lung cancer development. The first results of this study showed that the penetrance of germline T790M *EGFR* is variable, but in some individuals, germline T790M is associated with lung nodules or advanced NSCLC at a young age [20].

Insertions in exon 20 are rare activating mutations in the TK domain of *EGFR* [21]. The mutations of exon20 comprise a combination of in-frame insertions and/or duplications of 3 to 21 base pairs, usually clustered around codons 767 and 774. *EGFR* exon 20 of EGFR contains two major regions, the α-C helix (residues 762-766) and the loop following the α-C helix; crystallographic studies suggest that these mutants have a stabilized and rigid conformation inducing resistance to EGFR TKIs [46]. Arcila and coworkers have estimated a frequency of *EGFR* exon 20 insertion mutations of about 9% of all *EGFR* mutations occurring in lung adenocarcinomas [21]. In contrast to the more classic *EGFR* activating mutations, these *EGFR* mutations have been associated with de novo resistance to EGFR-TKIs, such as erlotinib, gefitinib, afatinib and dacomitinib [21]. At the clinical level, only a part of patients with EGFR exon 20 insertion mutations are sensitive to EGFR inhibitors, such as afatinib or dacomitinib [21]. In a recent study, Kosaka et al. evaluated the molecular basis of this heterogeneity of EGFR exon 20 insertion mutant lung adenocarcinomas and showed that patients achieving a partial response after dacomitinib treatment showed Gly770 as a common mutation; structural modeling suggested that may facilitate inhibitor binding to EGFR; furthermore, the introduction of Gly770 mutation into the EGFR molecule conferred sensitivity to dacomitinib [22]. Very recently, Robichaux and coworkers used in silico, in vitro, and in vivo testing to model structural alterations induced in the EGFR molecule by exon 20 *EGFR* mutations and identify effective inhibitors. Modeling studies have shown that *EGFR* exon 20 insertion mutations restrict the size of the drug-binding pocket, thus limiting the binding of large size inhibitors; to bypass the key limitations, it was discovered that the small molecule poziotinib, was able to circumvent these steric impediments and to exert a potent TKI effect [23]. Pre-clinical models of *EGFR* exon 20 insertion mutant lung carcinomas supported the potential clinical use of poziotinib. In a phase II clinical trial, the first 11 patients with lung adenocarcinomas with EGFR exon 20 insertion mutations receiving poziotinib exhibits an objective response rate of 64% [23]. These observations identified poziotinib as a potent, clinically active inhibitor of lung adenocarcinomas with EGFR exon 20 insertion mutations [23].

Recent studies have addressed the problem of the molecular characterization of EGFR-mutated lung adenocarcinomas to understand the spectrum of co-occurring genetic alterations present in these tumors and their possible impact in the biology and response of these tumors to treatment with EGFR TKIs. A recent study reported the genomic analysis of 1,122 *EGFR*-mutant lung cancer using cell-free DNA samples; this study allowed to identify critical co-occurring oncogenic events present in most advanced-stage *EGFR*-mutant lung cancers [24]. The data showed that canonical *EGFR* driver mutations co-occurred with oncogenic alterations in several other genes, including *PIK3CA, BRAF, MET, MYC, CDK6* and *CTNNB1* [24]. Interestingly, some co-occurring genetic alterations were more frequent among EGFR-mutated than in EGFR mutation negative cases: *CDK6* (7% vs 3.1%), AR (5.1% vs 2.6%), *TP53* (54.6% vs 50.3%) [24]. About 40% of the *EGFR*-mutant lung adenocarcinomas displayed a Thr790Met mutation, thus indicating they were pre-treated with a first generation or second generation EGF TKI; these patients displayed some quantitative differences compared to *EGFR*-mutated Thr790Met-negative cases, including a higher number of detectable genetic alterations and more frequent alterations of *CDK6, AR, MYC, CCNE1, KRAS, PDGFRA, CNG* and *BRCA1* [24]. This analysis allowed to identify several genetic abnormalities as biomarkers predicting a poor response to EGFR TKI treatment (osimertinib), such as the presence of genetic alterations at the level of *CDK4, CDK6, NF1, PI3KCA, MET, APC* and *CCNE1* ; patients with *CDK4/CDK6* and with alterations in cell-cycle genes have a reduced overall survival, compared to those without these alterations [24].The longitudinal analysis of some patients in first, second or third line of treatment with EGFR TKIs indicated that tumor genomic complexity increases with EGFR-inhibitor treatment, and co-occurring alterations in *CTNNB1* and *PIK3CA* exhibit non-redundant functions cooperating with *EGFR* driver mutations to promote tumor metastasis or to limit EGFR-inhibitor response [24]. Other recent studies have addressed the importance of co-occurring mutations. Thus, Vanderlaan and coworkers investigated 1712 *EGFR*-mutated tumor-patient cases and observed that 50%, 10% and 5% of these cases displayed *TP53, PIK3CA* and *PTEN* mutations, respectively; interestingly, the presence of the co-occurrent *TP53* mutations were associated with a reduced PFS [25]. These conclusions were confirmed and extended by Canale et al, who have investigated in detail the co-occurrence of *TP53* mutations in *EGFR*-mutated lung adenocarcinomas treated with TKI in first-line setting [26]. Patients with TP53 mutations showed a worse prognosis than TP53 wild-type patients; particularly, *TP53* exon 8 mutations were associated with a significantly lower disease control rate and shorter PFS and these results were particularly significant in a subgroup of patients with *EGFR* exon 19 deletion [26]. Aisner and coworkers have investigated 35 *EGFR*-mutated lung adenocarcinomas and have observed that 40% of these tumors have co-mutated *TP53*; patients harboring a *TP53* mutation treated with targeted therapy displayed a trend toward shorter survival compared to those without a TP53 mutation [3]. However, when *TP53* mutations were subdivided into disruptive and non-disruptive types, it appeared evident that disruptive *TP53* mutations were associated with a reduction in survival, in comparison with no *TP53* mutations in patients with EGFR mutations [3].

Nathar and coworkers have reported the description of the genomic architecture of *EGFR*-mutant lung adenocarcinomas through multiregional exome sequencing of 16 lung adenocarcinomas of Asian patients [27]. These tumors although were usually observed in non-smoker patients and have a low mutation burdens, displayed a considerable degree of genetic complexity related to a high-degree of intra-tumor heterogeneity and to the presence of copy number alterations [27]. The multi-region sequencing of 16 surgically resected stage I-II *EGFR*-mutated lung adenocarcinomas, treatment-naïve, showed that: *EGFR* mutations are truncal events (i.e.; the mutations are present in all sectors of a tumor), in line with the early origin of these mutations in tumor history; *TP53* mutations, present in 9 of 16 of these tumors, are in most of cases truncal events; only other 17 lung adenocarcinoma driver genes were found to be mutated and seven of them were featured as truncal events; the analysis of the ratio branch/private mutations was used as a measure of intra-tumor heterogeneity and showed a median heterogeneity corresponding at 62.3%; the analysis of copy number alterations showed that they are frequent events in lung adenocarcinomas, with the majority of tumors showing moderate to high genomic instability and with 12/15 tumors displaying whole genome doubling, a truncal genetic event [27]. The analysis of the timing of these genetic alterations suggests that *EGFR* and *TP53* mutations occurred prior to wide genomic doubling and local single copy number alterations [52]. Early-occurring single copy number alterations converge on disruption of the cell cycle control [27]. According to all these observations, it was suggested that a dominant truncal driver event represented by *EGFR* mutations, in the context of low genomic mutation rates and high genomic instability, results in early clonal selection and subsequent development of high intra-tumor heterogeneity [27]. Finally, the occurrence or not of co-occurring *TP53* mutations during the natural history of development of *EGFR*-mutant lung adenocarcinoma induces different clinical trajectories of tumor development. Another study evaluated the occurrence of concurrent genomic alterations in a group of Japanese patients with EGFR-mutated lung adenocarcinoma [28]. Japanese *EGFR*-mutant (exon 19 deletion and an L858R mutation) displayed more *CDKN2B1* and *RB1* alterations than TCGA patients [28]. Concurrent mutations in CDKN2B1 or RN1 were associated with negative clinical outcome in Japanese patients with *EGFR*-active mutations [28].

It is important to note that EGFR activation induces both the MAPK and the PI3K signaling pathways: in spite the activation of both these pathways in *EGFR*-mutant lung cancer cells, these cells exhibited higher sensitivity to PI3K inhibitors than to MAPK inhibitors. This observation may be explained by the finding that activated EGFR preferentially dimerizes with ERBB3, a strong PI3K activator.

Although the clinical trials using EGFR TK inhibitors have provided an improvement of life expectancy of NSCLC patients, development of acquired resistance to these agents remains a challenging problem. In fact, more than 50% of these patients develop secondary point mutations located in the kinase domain of the *EGFR* at the gatekeeper position T790. Recent studies carried out using sensitive technologies (MALDI-TOF) have shown that T790M mutations at the level of the EGFR are more frequent than previously reported, being detectable in 25-30% of lung adenocarcinoma patients with EGFR mutations pre-treatment and in >80% of these patients after therapy with tyrosine kinase inhibitors [29]. These observations are compatible with the hypothesis that populations of T790M cells within the tumor are scarcely represented before treatment and, gradually, increase after EGFR TKI therapy because of selection pressure [29]. A recent study explored the evolution of resistance caused by the T790M gatekeeper mutation in *EGFR* [30]. Through the monitoring of the development of a number of resistant clones in parallel, it was possible to define different temporal patterns of emergence, reflecting emergence from pre-existing resistant T790M clones and emergence from cells originally T790M negative and *de novo* acquiring the T790M mutation [30]. Importantly, the clones that evolved from drug-tolerant cells bear epigenetic hallmarks of drug-tolerant cells and have only a limited response to third-generation EGFR inhibitors that target T790M EGFR [30]. These observations indicate that drug-resistant cells bearing the same EGFR mutation can either pre-exist or evolve from drug-tolerant cells [30].

In addition to the secondary gatekeeper mutation, NSCLC patients whose tumors harbor sensitizing mutations and who respond to EGFR TK inhibitors may also acquire resistance through activation of MET, via HGF ligand and *MET* gene amplification, which serves to re-activate the PI3K signaling pathway. Sequist and coworkers have investigated into detail the mechanism of acquired resistance to EGFR TKIs and have made a number of interesting and intriguing observations. First, they showed that some resistant cancers displayed unexpected genetic changes, including *EGFR* amplification and mutations at the level of the *PI3KCA* gene, while other patients exhibited clear phenotypic changes underlying epithelial-to-mesenchymal transition; furthermore, very intriguingly, 14% of TKI resistant tumors undergo a transformation in SCLCs and acquired sensitivity to standard treatments for these last tumors [31]. The same authors have recently analyzed the molecular changes associated with transformation from NSCLC to SCLC observed in a subset of EGFR-resistant tumors. Importantly, it was observed that 100% of these transformed SCLC cases display RB loss, together with increased expression of neuroendocrine markers, decreased EGFR expression and increased sensitivity to BCL2 inhibitors [32]. The analysis of the available literature has indicated that the histological transformation from NSCLC to SCLC and the existence of combined histology lung tumors is more common in cancers that have *EGFR*-activating mutations than in *EGFR*-wild-type tumors [33]. To explain these findings, it was hypothesized that cells of origin of some *EGFR*-mutant adenocarcinomas, type II alveolar cells, have the potential of neuroendocrine differentiation and to become SCLC [58]. However, the capacity to transform into SCLC and transformation from adenocarcinomas to SCLCs has occasionally been observed in lung cancers that do not have *EGFR* mutations [33].

Disparate EGFR TKI resistance mechanisms converge on AKT activation: combined drug treatment with an EGFR TKI and AKT inhibitors causes apoptosis and synergistic growth inhibition in multiple models of EGFR TKI-resistant lung adenocarcinomas [34]. In line with these findings, AKT levels are increased in clinical specimens derived from *EGFR*-mutant lung adenocarcinomas patients with acquired EGFR TKI resistance [34]. Karachaliou and coworkers have performed a gene expression analysis in two cohorts of lung adenocarcinoma patients treated with EGFR TKIs (gefitinib or osimertinib) and observed that elevated expression of *CDCP1* and *AXL* was an independent negative prognostic factor for both PFS and OS [35].

Recent studies have in part clarified the molecular mechanisms through which activated EGFR may induce lung cancer development and have also allowed the identification of molecules induced by EGFR activation that can be therapeutically targeted. Gene expression studies have shown that the transcription factor NKX2-1, a homeodomain transcription factor, is a reliable marker for “TRU-type” adenocarcinomas, associated with *EGFR* mutations. Importantly, functional studies have shown a role for NKX2-1 as a lineage survival oncogene in lung adenocarcinoma. In was shown that NKX2-1 induces the expression of Receptor tyrosine kinase Orfan Receptor 1 (ROR1) which favors PI3K-AKT survival pathway at the expenses of the pro-apoptotic p38 signaling, through c-Src activation, and favoring the EGFR-ERBB3 association, and the consequent ERBB3 activation and consequential PI3K activation [36]. Importantly, knockdown of *ROR1* expression induced an inhibition of both *EGFR*-mutant and *EGFR*-WT NSCLC cell lines: in this context, it is important to note that ROR1 inhibition markedly inhibited the growth also of *EGFR*-mutant NSCLC cell lines resistant to the EGFR inhibitor gefitinib [36]. Sangodkar and coworkers have shown that in *EGFR*-mutant lung adenocarcinoma cells the KLF6 transcription factor is completely downmodulated via a transcriptional repressive effect mediated by the transcription factor FOXO1 [37]. Interestingly, the drug trifluoperazine hydrochloride, which inhibits the FOXO1 nuclear export, restored sensitivity of EGFR TK resistant lung cancer cells *in vitro* and *in vivo* [37]. These observations may represent the basis for the identification of an important EGFR oncogenic signaling pathway that can be targeted for the treatment of metastatic lung adenocarcinoma.

The identification of specific druggable genetic abnormalities of NSCLCs offers the opportunity for development of new therapeutic approaches. In this context, most NSCLC patients exhibit only a mixed response: some tumor lesions regress upon treatment, while other one’s progress upon treatment with EGFR TKIs. These mixed responses are related to the tumor heterogeneity of *EGFR* mutations between the primary lung tumors and their metastases [38].

2. ALK-Rearranged Lung Adenocarcinomas

A recent study provided a detailed molecular characterization of *ALK*-rearranged NSCLCs, based on the detailed analysis of 158 cases. The large majority of these tumors exhibited an adenocarcinoma morphology. The analysis of these patients showed the absence of *EGFR* and *KRAS* mutations or rearranged *ROS1* or *RET, TP53* was the most frequent (11%) mutated gene, followed by *ARID1A* (7%), *ATRX* (6%), *NF1* (6%) *NOTCH1* (5%) and *ROS1* (5%); furthermore, these tumors displayed fewer mutations in *PIK3CA, CDKN2A* and *RB1* compared with those present in the TCGA data set [39]. *ALK-EML4* is the most frequent translocation observed in these tumors and is present in two different forms: *ALK-EML4* short forms have lower sensitivity to ALK inhibitors and their expression in tumors is associated with a more advanced stage of disease, more metastases, and poor outcome [39].

ALK-positive patients are usually younger than those with *EGFR* mutations and are usually non-smokers or slight smokers [40]. A meta-analysis on data collected from the study of 6950 patients showed an incidence rate of *AKT-EML4* fusion in NSCLC patients corresponding to 6.8% [66]. The incidence of *ALK-EML4* fusion was prevalent in adenocarcinomas, non-smoking NSCLC patients and was completely exclusive of *KRAS* mutation genes and partially exclusive of *EGFR* mutation (2% of these patients displayed EGFR mutations) [41]. Usually these patients present in late stages of disease and are not amenable for surgical resection [41]. *ALK* has three reported fusion partners: *EML4*, *KLC1* and *KIF5B*. *ALK* rearrangement-positive lung cancers have been effectively treated with ALK inhibitors: however, the magnitude and duration of the response was heterogeneous in function of the various *ALK-EML4* fusion variants, thus indicating that targeted therapy of these tumors should take into account the precise *ALK* genotype [67]. Recently, in a cohort of 488 patients, the prognosis of *ALK/EML4* fusion-positive NSCLC patients has been evaluated [42]. In stage I patients, *ALK/EML4*-positive patients had better prognosis than *ALK/EML4* negative patients. In contrast, in stage III patients, *ALK/EML4*-positive patients had poorer disease-free survival than *ALK/EML4*-negative patients [43]. *ALK/EML4* positive patients express elevated levels of the ERCC1 protein, a molecule playing a key role in mediating platinum sensitivity, thus suggesting that this tumor should be candidate to platinum-based regimens [43].

Other more recent studies have shown a possible link between the type of ALK molecular abnormality and the response to therapy. Thus, Rosenbaum and coworkers have shown that about 88% of ALK^+^ NSCLC have ALK rearrangements involving *EML4, KIF5B*, or non-canonical partners involving *ASXL2, ATP6V1B1, PRKAR1A*, and *SPDYA* [44]. Survival analysis of patients treated with targeted ALK inhibitors showed a significant difference between patients bearing *ALK-EML4* rearrangements, and those without (20.6 months vs 5.4) [44]. The most frequent *EML4-ALK* variant 1 (exon 13 of EML4 fused to exon 20 of ALK) and variant 3 (exon 6 of EML4 fused to exon 20 of ALK): ALK resistance mutations were significantly more common in variant 3 than in variant 1 [45].

*ALK/ELM4*-positive NSCLCs are markedly sensitive to the ALK inhibitor crizotinib. Progression-free survival was significantly longer with crizotinib than with chemotherapy (premetrexed+cisplatin) for *ALK/EML4*-positive patients [46]. However, these patients after an initial response, develop resistance to this kinase inhibitor. Various mechanisms seem to be responsible for the development of resistance and, particularly, the occurrence of secondary *ALK* mutations, as well as *ALK* copy number gain; in some patients the development of *EGFR* mutations was observed [47]. A recent study showed an additional mechanism of resistance related to some P2Y purigenic receptors acting via protein kinase C activation: crizotinib-resistant tumors treatment combined with an ALK and a PKC inhibitor restored drug sensitivity [48]. The analysis of some *ALK* fusion-positive lung cancer patients exceptionally responding to drug treatment suggested a sensitivity of some of them to IGF-1R inhibitors [74]. In models of ALK TKIs resistance, as well as in tumor biopsies of patients progressing on crizotinib monotherapy, the IGF-1R pathway was found to be activated and the combined inhibition of both ALK and IGF-1R improves therapeutic efficacy [49]. The majority of patients treated with crizotinib, a first generation ALK inhibitor, relapse within 12 months. 56% of patients resistant to crizotinib respond to ceritinib, a second generation ALK inhibitor and recently this drug was approved for the treatment of crizotinib-resistant patients [50]. The overall survival of ALK-rearranged NSCLC patients treated first with crizotinib and then with ceritinib was about 50 months [51].

At variance with EGFR, the mutations that confer resistance to the ALK inhibitor crizotinib are more heterogeneous and there is not the equivalent of *T790M* for *EGFR*. The gatekeeper mutation *ALK-L1196M* was observed in about 6% of patients treated with first-generation ALK1 inhibitors; the *G1202R, D1203N* and S1206 solvent-front mutations in *ALK* have been related to resistance to crizotinib [52]. The *G1202R* mutation conferring resistance to all approved inhibitors is observed in about 2% of patients at resistance with first-generation ALK inhibitors, but it is the most frequent common mutation in patients treated with later-generation ALK TKIs; this mutation is sensitive to lorlatinib, a third-generation ALK TKI under clinical evaluation [52]. In addition to these mutations, other mutations associated with resistance are *ALK-G1269A* (a mutation at the level of the ATP binding pocket hindering drug binding) and a series of mutations, such as *ALK-1151T* insertion, *ALK-F1174C, ALK-1152R, ALK-1156Y*, inducing conformational changes of ALK [52].

After the initial studies with crizotinib, subsequent studies have explored new ALK inhibitors with the specific aim of improving the anti-tumor effects, of bypassing crizotinib resistance and of preventing brain metastases. Basically, three new ALK inhibitors have been evaluated, including ceritinib, alectinib and brigantinib. The studies carried out using ceritinib have shown that this ALK inhibitor, when used in first line, showed a statistically significant and clinically meaningful improvement in progression-free survival versus chemotherapy in patients with advanced *ALK*-rearranged NSCLC [53]; when used in lung adenocarcinoma, *ALK*-rearranged patients who have failed after crizotinib showed a more potent anti-tumor effect, compared to chemotherapy [54]. Alectinib is a highly selective and potent inhibitor of ALK, displaying systemic and central nervous efficacy in the treatment of *ALK*-positive NSCLCX in phase I/II studies. Given these promising results, alectinib was evaluated in comparison to crizotinib in previously untreated, advanced ALK-positive NSCLC, including those with asymptomatic central nervous involvement: as compared to crizotinib, alectinib showed superior efficacy and lower toxicity in primary treatment of *ALK*-rearranged NSCLC [55, 56]. Alectinib significantly improved systemic and central nervous system efficacy versus chemotherapy for crizotinib-pretreated *ALK*-positive NSCLC patients [57]. Brigantinib is a novel and selective ALK inhibitor able to inhibit ALK with a 12-fold higher potency than crizotinib, but inhibited also EGFR mutants. The clinical studies showed a remarkable efficacy of brigantinib in crizotinib-resistant, *ALK*-rearranged NSCLC [58]. In these studies, brigantinib showed a remarkable anti-tumor activity, not only at the level of the whole body, but also at the level of the central nervous system, eliciting a remarkable improvement of intracranial outcomes, both in terms of objective responses and progression-free survival [58, 59].

Recently, it was tested the clinical activity of a third generation ALK inhibitor, lorlatinib. As above stated, pre-clinical studies supported the use of lorlatinib in lung adenocarcinomas resistant to first- and second-generation ALK TKIs [58]. 46% of lung adenocarcinoma patients who had already received two or more ALK TKIs displayed an objective response to lorlatinib [60]. These observations support the use of lorlatinib in lung cancer patients who had become resistant to other available TKIs, including second-generation ALK TKIs, and is actually under investigation in a phase III randomized controlled trial comparing lorlatinib to crizotinib. Unfortunately, as with other ALK inhibitors, acquired resistance to lorlatinib develops in all treated patients. Molecular analysis of a single patient treated sequentially with crizotinib, ceratinib and lorlatinib and the analyzed at the stage of lorlatinib resistance: at this stage, the patient displayed a novel double compound mutation *ALKC1156Y/L1198F*, with both mutations present on the same allele; paradoxically, this mutation conferred resistance to lorlatinib, but re-sensitized the tumor to crizotinib [52]. A recent study provided evidence that the acquisition of compound mutations is a common mechanism (35% of cases) of developing resistance to lorlatinib [61].

Other ALK TKIs are under evaluation. Ensartinib is a potent ALK TKI, recently explored in ALK-positive NSCLC patients in the context of a phase I/II clinical trial. Among the ALK-positive efficacy evaluable patients treated at optimal doses, the response rate was 60% and median progression-free survival was 9.2 months for the whole population of ALK^+^ patients and the response rate 80% and the progression-free survival 26.2 months for ALK TK naïve patients [62].

McCoach and coworkers have explored the molecular mechanisms of resistance of ALK-positive lung adenocarcinomas to ALK TKIs [63]. The results of this study provided evidence about two different mechanisms of resistance: one, related to alterations that restore signaling through the oncogene driver in the presence of the drug (occurring in about 35% of patients and represented by ALK kinase domain mutations) and the other one related to alterations that switch dependence to other signaling pathways (*NRG1* gene fusion, *RET* fusion, *EGFR* or *KRAS* mutations, *IDH1*, *RIT1*, *NOTCH* and *NF1* mutations) [63]. These observations strongly support the need of an extensive characterization of abnormalities occurring in ALK^+^ lung adenocarcinomas who have developed resistance to treatment with ALK TKIs [63]. Another recent study explored the mechanisms of ALK TKIs resistance, not associated with resistance mutations in *ALK*. Thus, Dardaei and coworkers have characterized this type of ALK-resistant tumors first showing that they are resistant to Lorlatinib, the third-generation inhibitor targeting ALK, and then, through a shRNA screen of 1,000 genes, showing that these tumors are sensitive to inhibition of SHP2 inhibitor, a non-receptor protein tyrosine phosphatase [64]. Thus, the SHP099 SHP2 inhibitor together with ceritinib, inhibited the growth of ALK-resistant lung adenocarcinoma cells, preventing compensatory RAS and ERK1/ERK2 reactivation [64]. Finally, a recent study provided evidence that also enhancer remodeling and altered expression of miRNAs may play a role in the development of ALK TKI resistance and suggest also the strategies targeting epigenetic pathways may represent a potentially effective strategy for overcoming acquired resistance to ALK TKIs [65].

The analysis of the genomic mutations in lung adenocarcinoma patients developing resistance to ALK TKIs requires tumor biopsies. However, the recently developed analysis of cell-free circulating tumor DNA may provide a non-invasive way to identify *ALK* fusions and actionable resistance mechanisms without the need of biopsy [66, 67]. These studies provided evidence that in most of cases it is possible to obtain tumor DNA from plasma and is possible to perform a comprehensive molecular characterization whose results are in line with those obtained on tumor biopsies [66, 67]. Longitudinal analysis of plasma specimens from ALK-positive patients with acquired resistance to ALK TKIs was suitable to track the evolution of resistance mechanisms during treatment [67].

3. ROS1-Rearranged Lung Adenocarcinoma

ROS1, a receptor tyrosine kinase of the insulin receptor family, is rearranged in about 2% of NSCLC patients. Translocations leading to *ROS1* fusion transcripts were shown to lead to constitutive tyrosine kinase activity and sensitivity to tyrosine kinase inhibitors. Patients with ROS1 rearrangements are frequently never/slight smokers. Analysis of the main features of these patients showed that ROS1 rearrangements identify a molecular subset of NSCLC patients with distinct clinical characteristics that are at large extent similar to those observed in patients with *ALK*-rearranged NSCLC [68]. In a recent study based on the molecular screening of 447 NSCLCs, *ROS1* rearrangements have been observed in 1.2% of cases: the five patients with *ROS1* rearrangements displayed in one case *SDC4-ROS1* fusion; in two cases *CD74-ROS1* fusion and the remaining two cases *SLC34A2-ROS1* fusion [69]. The analysis of 36 lung adenocarcinoma patients displaying *ROS1* fusion events showed in about 55% of cases a *CD74-ROS1* fusion transcript, and in the remaining 45% *EZR-ROS1* (19% of cases), *SDC4-ROS1* (11% of cases) and more rarely *SLC34A2-ROS1* or *TMP3-ROS1* fusion transcripts were observed [70]. Cell lines with *ROS1* rearrangements were inhibited in their proliferation and survival by ROS1 inhibitors [72]. ROS-rearranged lung cancers are more frequent among non-smokers; at the histological level these tumors display the solid signet-ring cells and mucinous cribiform pattern, observed also in *ALK* fusion tumors [71].

36% of *ROS1*-rearranged lung adenocarcinomas display concomitant driver oncogenic mutations involving either *EGFR* or *KRAS* or *BRAF* [72]. This heterogeneity may offer additional therapeutic opportunities in these patients [72]. However, in another study based on the analysis of 62 patients with *ROS1*-rearranged lung adenocarcinoma, concomitant driver oncogenic mutations were observed very rarely, with absent *EGFR, BRAF, ERBB2, AKT1, PIK3CA* and M*AP2K21* mutations and *KRAS* mutations present in 3.2% of cases [98]. The analysis of a dataset on 166 *ROS1*-rearranged lung adenocarcinomas confirmed the absence of concomitant mutations of driver oncogenes, with the exception of K*RAS*, mutated in about 2% of patients [73].

Lung cancers with ROS fusions are highly sensitive to treatment with ALK inhibitors: in fact, 72% of patients responded to crizotinib, with 5% of complete responses and about 50% of partial responses; the median duration of response was 17.6 months [74]. Although *ROS1*-rearranged NSCLC is sensitive to crizotinib, development of resistance frequently occurs. Analysis of crizotinib-resistant tumors from NSCLC patients with the *CD74-ROS1* rearrangement showed that molecular changes associated with this acquired resistance are heterogeneous, including ROS1 tyrosine kinase domain mutations, EGFR activation and epithelial-to-mesenchymal transition [75]. The cMET/RET/VEGFR inhibitor cabozantinib was able to overcome the resistance of all the identified ROS1 kinase domain mutants resistant to crizotinib [76]. Another promising new ROS1 inhibitor is PF-06463922, an ATP-competitive ALK/ROS1 inhibitor, inhibiting at sub-nanomolar concentrations ROS1 fusions and the crizotinib-resistant *ROS1*^G2013R^ mutation and the ROS1^G2026M^ gatekeeper mutation [77]. This activity compound exhibited a pronounced anti-tumor activity against suitable tumor mouse models [77].

Recently, crizotinib was evaluated in a large group of 127 Asian NSCLC patients with *ROS1*-rearranged tumors who had received three or fewer lines of prior systemic therapies [103]. An objective response was observed in about 72% of patients, with 12% of complete responses and 60% of partial responses, with a median progression free survival of 15.9 months [78].

Given the sensitivity of ROS1 rearrangements to crizotinib, this drug was approved in 2016 by the FDA for use in patients with metastatic NSCLC harboring a ROS1 fusion. The study of *ROS1*-positive NSCLCs showed that these tumors, compared with *ALK* rearrangements, are associated with lower rates of extra thoracic metastases, including fewer brain metastases, at initial diagnosis [79]. In these patients, ROS1 resistance mutations, particularly G2032R, appear to be the predominant mechanism of resistance to crizotinib [104]. Patients with NSCLC and *ROS1* rearrangements treated with crizotinib have a longer survival, regardless of prior smoking history; co-existing TP53 mutations were associated with shorter survival among these patients [79].

The G2032R [80] and D2033N [81] *ROS1* mutations represent the most recurrent mechanism of crizotinib resistance in ROS1-rearranged NCSLCs. The *ROS1-G2032R* mutation, analogous to the ALK-G1202R mutation, is highly potent and challenging to overcome and is the most common mutation conferring resistance to crizotinib in ROS1-driven lung adenocarcinomas [80]. The *ROS1-D2033N* mutation, analogous to the *ALK-D1203N* mutation, is located to the ATP binding site and alters electrostatic interaction with crizotinib [81]. Other *ROS1* mutations are analogous to *ALK* mutations and this finding is not very surprising given the structural similarities between *ROS1* and *ALK1*. Thus, the *ROS1* mutation S1986Y/F is analogous to the *ALK-C1556Y* mutation: both these mutations alter the position of the αC helix, and thorough this mechanism, inhibit crizotinib binding [82]. Another mechanism of resistance to crizotinib involves activation of EGFR: EGFR enabled bypass signaling to critical downstream pathways, such as MAPK [108]. These observations indicate that EGFR signaling can provide a fundamental adaptive mechanism that allows cancer cells to evade oncogene-specific inhibition [83].

Other ROS inhibitors are currently under clinical investigation. In this context, entrectinib, a multikinase inhibitor with activity against ROS1, elicited an objective response in 86% of patients with advanced ROS1 rearranged solid tumors [84]; interestingly, the majority of responding patients are NSCLC patients with ROS1 rearrangements, with a median duration of response of 17.4 months [84]. Particularly promising were the results obtained using another multikinase inhibitor, ceritinib: in a phase II study carried out on 32 patients with ROS1-rearranged NSCLC, 62% of objective responses were observed, with a median overall survival of 24 months [85]. Importantly, among the responding patients there were also patients pretreated with crizotinib and control of brain metastases was observed in >50% of patients with this type of metastases [85].

Although crizotinib is recommended as first-line therapy in *ROS1*-driving lung adenocarcinoma, the optimal first-line therapy for this group of lung cancer is still controversial. A number of studies have suggested that *ROS1*-rearranged NSCLCs are sensitive to pemetrexed-containing chemotherapy [86-88].

A third-generation, brain penetrant, tyrosine kinase inhibitor lorlatinib induced 50% of objective responses in a group of SCLC patients with *ROS1* rearrangement (some of them pre-treated with crizotinib) [60]. Lorlatinib may be a therapeutic strategy for patients with ROS1-rearranged NSCLC who have become resistant to currently available TKIs [60].

Lin and coworkers have proposed a therapeutic algorithm for ROS1-mutated NSCLCs. Crizotinib is the standard of care fist-line TKI for the treatment of advanced ROS1-rearranged lung adenocarcinomas; once patients progress during or after crizotinib treatment, it is recommended a repeat tumor biopsy (or a liquid biopsy) to determine the mechanisms of resistance and to identify the presence of any *ROS1* resistance mutations; the presence of a ROS1-resistance mutation suggests a certain degree of ROS1 dependency and suggests that the patient must be addressed toward clinical trials based on new ROS1 inhibitors; the absence of ROS1 resistance mutations indicates that the patient must be addressed to chemotherapy or to combination treatments [89].

4. RET Kinase-Rearranged Lung Adenocarcinoma

In addition to *ROS1* and *ALK* gene fusions, lung adenocarcinomas have been found to harbor gene fusions involving the *RET* tyrosine kinase gene and the partner genes *KIF5B* or *CCDC6* [115]. Of these patients, the majority had *KIF5B-RET* and *CCDC6-RET* fusions, while a minority of them had *NCOA4-RET* fusions [90]. At least 12 fusions *RET* partner gene have been identified to date. Particularly, *RET* fusions have been observed in 1.4% of NSCLCs and in 1.7% of lung adenocarcinomas, characterized by selected clinic-pathologic characteristics [91]. Some of these features correspond to a poor differentiation status and to a predominant occurrence among non-smoker patients and to early lymph node metastases [91]. A peculiarity of RET-rearranged lung adenocarcinomas is their poor differentiation status, with frequent signet-cell morphology.

*RET* fusion genes have been considered mutually exclusive with other molecular alterations. However, the molecular characterization of 22 patients showed in 8/22 TP53 mutations, in 1/22 *MET* amplification or *CTNNB1* mutation or *EGFR* mutation [92]; in another study, a retrospective analysis on 28 patients showed *EGFR* mutations in 6 cases [93].

Although RET-selective inhibitors have not yet been developed, clinically available tyrosine kinase inhibitors such as sunitinib, sorafenib, and vandetanib target RET kinase activity and this suggests that this NSCLC subtype could be treatable with a kinase inhibitor [94]. Given these observations, several clinical trials are undergoing and are evaluating the clinical response of patients with KIF5B-Ret associated NSCLC to targeted therapy using RET-tyrosine Kinase Inhibitors, such as levantinib, vandetanib, sunitinib, ponatinib, cobozantinib and AUY922. A preliminary report on three *RET*-rearranged lung adenocarcinoma patients showed a significant anti-tumor activity of cobanzatinib [94].

In a phase II study, 28% of patients with lung cancer harboring a *RET* rearrangement showed partial response to treatment with Cobazantinib [95]. A recent retrospective analysis on 165 patients with *RET*-rearranged lung adenocarcinoma showed that, among various mulitikinase tested, cabozantinib was the most active drug with 37% of overall responses [43]. However, the median overall survival was limited, thus suggesting that currently available multikinase inhibitors have limited activity in patients with *RET*-rearranged NSCLC [96]. The conclusion is that response rates to RET-directed therapy with currently available multikinase inhibitors are modest compared with those achieved using targeted therapies matched to other lung oncogenic drivers, such as *EGFR, ALK* or *ROS1* rearrangements [97] and this may be due either to the limited tractability of RET lung adenocarcinomas or to the lack, until now, of highly specific inhibitors of their oncoprotein in the clinic.

A recent study explored in detail a patient with *RET*-rearranged lung adenocarcinoma harboring *CCDC6-RET*, initially responding to vandetanib and then relapsing with a secondary mutation resulting in a serine-to-phenylalanine substitution at codon 904 in the activation loop of RET kinase domain: the S90F mutation confers resistance to vandetanib by increasing the ATP affinity and auto-phosphorylation activity of RET kinase [70].Thus, a missense mutation in the RET kinase domain is able to increase kinase activity and to confer drug resistance by an allosteric effect [98].

A recent study reporting the identification of AD80 and ponatinib as two drugs able inhibit RET downstream signaling in a 1000 to 1000-fold more effectively than cabozantinib or vandetanib [99]. Phosphoproteomic, biochemical and structural data indicated that AD80 and ponatinib have an optimal RET-specific profile, distinguishing them from currently available anti-RET drugs [124]. Importantly, only these two RET inhibitors displayed high activity against gatekeeper RET mutants *KIF5B-RET^V804M^* or *CCD6-RET^V804M^* [124]. Finally, AD80 potently shrinks RET-rearranged tumors in patient-derived xenografts [99].

Very recently, it was reported BLU-667, a next generation small-molecule RET inhibitor, specifically designed for highly potent and selective targeting of oncogenic RET alterations, including RET fusions and RET activation mutations; this compound displayed an increased potency and selectivity compared to compounds previously investigated for their RET kinase inhibitory activity [100]. In initial clinical studies, BLU-667 showed the expected capacity to reduce RET signaling and induced clinical responses in patients with *RET*-rearranged NSCLCs [100]. The clinical evaluation for this compound is ongoing in phase I clinical studies [100].

5. KRAS-Mutated Lung Adenocarcinoma

Activating mutations at the level of codons (G12C is the more frequent mutation and G12D and G12V the less frequent mutations) 12 and 13 of the *KRAS* oncogene occur in about 25% of lung adenocarcinomas and are mutually exclusive to *EGFR, HER2* and *BRAF* mutation and to *ALK* rearrangements. KRAS mutations seem to be an early event during the development of smoking-related NSCLC. These tumors are scarcely responsive to standard treatments and no drugs are available capable of directly and specifically inhibiting KRAS. 30% of *KRAS*-mutant adenocarcinomas display a loss of *LKB1*: *LKB1* loss is more common in smokers and is associated with a more aggressive clinical phenotype in *KRAS*-mutant NSCLC patients [101].

The frequency of occurrence of these three major driver mutations at the level of *EGFR, KRAS* and *ALK*, is in large part responsible for the differences in survival observed between smokers and never-smokers lung adenocarcinoma patients. Particularly, patients who had *KRAS* mutations had the poorest survival and since they are frequent among smoker patients, this explains the shorter survival of smoker versus non-smoker lung adenocarcinoma patients [102]. For these patients targeted therapies have been largely unsuccessful and chemotherapy remains the only standard of therapy. At the moment, the prolonged strategy of treatment of these tumors involve the use of docetaxol in combination with a MEK inhibitor; alternatively, a MEK inhibitor may be used in combination with an AKT inhibitor. As above mentioned, *KRAS* mutations are more frequent among smoker (about 30%) than never-smoker (about 10%) patients. The sequence of the mutated KRAS in smokers and non-smokers patients indicated different mutations in these two different types of patients suggest that most *KRAS*-mutant lung cancers in never smokers are not due to second hand smoke exposure [103]. The type of *KRAS* mutations (either at the level of codon 12 or of codon 13) does not seem to have a prognostic impact in NSCLC patients. In addition to KRAS mutations, more rarely lung cancer patients display *NRAS* mutations. In a large series of lung cancer patients, *NRAS* mutations have been observed on 0.7% of patients: 83% of these patients had adenocarcinoma and the large majority of them (>90%) do not have other driver mutations; furthermore, 95% of these patients are or were smokers [104]. *NRAS*-mutant lung cancer cell lines are sensitive to MEK inhibitors [104].

Therapeutic targeting of KRAS is a large and unmet goal of clinical oncology. As discussed above, KRAS itself has proved difficult to inhibit and the effectiveness of agents that target key RAS effectors has been considerably limited by the activation of parallel or compensatory pathways markedly reducing the anti-tumor effects of these drugs. In this context, a typical example is given by the MEK inhibitor trametinib, whose efficacy as single drug is strongly limited for the treatment of *KRAS*-mutant lung adenocarcinomas. A recent study identified an important mechanism hampering response of *KRAS*-mutant lung cancers to trimetinib: in fact, this drug induces a compensatory response involving FGFR1 that leads to signaling rebound and adaptive drug resistance [105]. In line with this observation, genetic and pharmacologic FGFR1 inhibition, together with trametinib, markedly potentiates anti-tumor effects *in vitro* and *in vivo* [86]. It is important to note that KRAS-mutant lung adenocarcinoma cells and patients’ tumor cells treated with trametinib show increased expression of biomarkers of FGFR activation [130]. The study of animal models of *KRAS*-driven lung cancers indicated a new potential therapeutic strategy. In fact, Sanclemente and coworkers have generated oncogenic *KRAS* mice that allow inducible deletion of *RAF1*, encoding *c-RAF* or *BRAF* in established *KRas^G12V^* or *KRas^G12V;TP53-/-^* lung tumors [106]. The systemic *c-RAF* ablation determines a significant tumor regression, without having an impact on MAPK activity and with a limited toxicity [131]. These results suggest that c-RAF inhibition may represent a therapeutic strategy for the treatment of *KRAS*-mutant lung adenocarcinomas [106]. Another experimental study suggested an unexpected strategy to inhibit *KRAS*-driven lung cancers [107]. Interestingly, blocking of KRAS dimerization between *KRAS*-WT and *KRAS*-mutant suppresses downstream signaling and the growth of *KRAS*-mutant lung cancers [107]. Therefore, blocking KRAS dimerization may represent a new strategy to block *KRAS*-mutant lung adenocarcinomas [107].

6. RASA1/NF1-Mutant Lung Adenocarcinoma

A *RASA1* (a Ras-GTPase-activating protein) inactivating mutation is observed in 1% to 3% of patients with lung adenocarcinoma; the *RASA1* mutation is highly enriched in patients with NF1-mutant lung cancer adenocarcinomas, and co-mutation of *RASA1/NF1* exhibited complete mutual exclusivity with *KRAS* and *EGFR* mutations, compared with single mutation of either the *RASA1* or *NF1* gene [108]. Co-occurring loss-of-function mutations of *RASA1/NF1* cause a marked dysfunction of GTP hydrolysis, with consequent accumulation of GTP-bound RAS proteins, activating MAPK, PI3K/AKT and Ral GDS pathways, thus inducing oncogenic proliferation. Interestingly, lung cancer cells with concomitant *RASA1* and *NF1* mutations are exquisitely sensitive to MEK inhibition [133]. These observations support the treatment of the rare cases of *RASA1/NF1* co-mutated lung adenocarcinomas with the MEK inhibitor trametinib [108].

7. FGFR Fusion Lung Adenocarcinoma

FGFR1/3 fusions have been observed in 1.3% of NSCLCs [134]. The most frequent fusions were represented by *FGFR3-TACC3* fusion (1.1%) and, less frequently, *FGFR1-BAG4* fusions [109]. These lung cancers seem to form a unique molecular subtype, with some peculiar features, such as: very high frequency (94%) of smokers; presence of large tumors at diagnosis and poor differentiation [80]. These patients could beneficiate from FGFR-targeted therapy [109]. In another study including both Asian and Caucasian patients, *FGFR-TACC3* fusion was observed in 0.5% of lung adenocarcinomas [110].

Interestingly, Chandrani et al have recently reported a study investigating actionable mutations in a group of 363 lung adenocarcinoma patients of Indian origin and reported the occurrence of *FGFR3* mutations in 5% of these patients [111]. These *FGFR3* mutations determine constitutive activation of the receptor and are oncogenic in experimental models [111]. In the majority of cases, *FGFR3* mutations are mutually exclusive with other major driver mutations, with the exception of few cases displaying either *EGFR* or *KRAS* co-mutations [111]. *FGFR3* mutations are more frequent among younger patients and show a trend toward a better overall survival [111]. It is of interest to note that in other studies in Caucasian patients *FGFR3* mutations were found to be absent in lung adenocarcinomas and only Imielinski et al reported *FGFR3* mutations of unknown functional significance in 3 out 183 lung adenocarcinoma patients [112].

8. Lung Adenocarcinoma Associated with PIK3CA Mutations

*PI3KCA* gene is frequently (2.5%) mutated in NSCLC. There are five hot-spot activating point mutations in *PI3KCA*, three in exon 9 and two in exon 20. As a result of these activating mutations, the downstream AKT pathway is deregulated, leading to enhanced MAPK (ERK1/ERK2) activity, promoting in turn cell proliferation.

*PI3KCA* mutations are heterogeneous in lung adenocarcinoma, involving most frequently E545K or H1047R and are frequently associated with other recurrent driver mutations, such as *KRAS* (in the majority of cases) or *EGFR* or, more rarely, MEK1, ALK and BRAF [113]. In lung adenocarcinoma patients, the presence of *PI3KCA* mutations in the absence of *EGFR* or *KRAS* co-mutations is associated with a poor prognosis [114]. As above mentioned, PI3KCA mutation co-exists in some patients with *EGFR* mutations. The study of a large set of *EGFR*-mutant lung adenocarcinomas allowed to conclude that the presence of *PI3KCA* mutations is not associated with primary resistance to EGFR TKIs; furthermore, acquired *PIK3CA* mutation related to EGFR TKI treatment is a rare event [115].

9. BRAF-Mutant Lung Adenocarcinoma

Mutations of BRAF, a kinase linking RAS GTPase to downstream MAPK pathway, occur in about 1-5% NSCLC. It is important to note that almost all these patients were current or former smokers. The *BRAF* mutations occurring in NSCLC are different from those occurring in melanoma in that 50% of these patients harbor non-V600E mutations: these mutations occurred in exons 11 and 15 (G469A and D594G) [116]. However, a recent screening on 951 patients with lung adenocarcinoma showed that *BRAF* mutations, observed in 2.2% of cases, were most commonly V600E [116]. According to the analysis of a large set of NSCLCs it was concluded that approximately 30% of patients display V600E mutations and in the remaining part, non-V600E the mutations often occur in the phosphate-binding loop (P-loop) at G466 and G469 [140]. *BRAF^V600E^* mutant displays a several hundred fold elevation of BRAF kinase over wild-type BRAF; the biochemistry of BRAF^non-V600E^ mutants is variable: while some of the mutants, such as *BRAF^G469A^* and *BRAF^L597V^* have high kinase activity, other mutants, such as *BRAF^G466V^* and *BRAF^D594G^*, have impaired BRAF kinase activity, but are still able to activate the MAPK pathway in alternative routes, acting in heterodimerization with CRAF [116]. Another remarkable difference between *BRAF^V600E^* and *BRAF^non-V600E^* is that the latter ones, but not the former ones, are associated in some cases with concomitant KRAS mutations; furthermore, particularly *BRAF^non-V600E^* mutants with impaired kinase activity are associated with concomitant KRAS mutations [116]. Salimian and coworkers have reported the analysis of 36 cases of NSCLC harboring *BRAF* mutations; only 28% of cases displayed the actionable V600E mutation [117]. Concurrent mutations were identified in *KRAS* (11% of cases), *PIK3CA* (6%) and *AKT1* (6%) [118]; the tumor grading varied from well to poorly differentiated, and morphology assumed various patterns [119].

Recent studies support an important role on hypoactive *BRAF^non-V600E^* mutants in lung adenocarcinoma development. Mutational analysis of different human cancers has provided evidence that among the BRAF hotspots in lung adenocarcinoma, those resulting in inactivating mutations predominate over the V600E activating mutation [120]. A retrospective analysis identified *BRAF^D594G^* as the most frequent BRAF alteration occurring in lung adenocarcinoma patients [121]. The analysis of BRAF inactivating mutants in lung adenocarcinoma patients showed a limited co-existence with RAS upstream mutations (11%), but more frequent mutations at the level of genes encoding RTK antagonists, determining a cooperation in sustaining MAPK activity [121]. The oncogenic role of *BRAF*-inactivating/hypoactive mutations is directly supported by mouse models of lung tumorigenesis: these *BRAF* mutants alone, or in cooperation with *KRAS^G12V^* induce lung adenocarcinoma formation in mice [122].

A recent study showed that, based on their mechanism of activation, BRAF mutants occurring in human tumors, including lung adenocarcinoma, can be subdivided into three different groups: class 1 includes BRAF mutations, such as *BRAF^V600E^*, that are RAS-independent, signal as monomers and are sensitive to RAF monomer inhibitors, such as Vemurafenib; class 2 includes *BRAF* mutants, RAS-independent, that signal as constitutive dimers and are resistant to Vemurafenib, but are sensitive to RAF dimer inhibitors or MEK inhibitors; class3 includes *BRAF* mutants with low or absent kinase activity, RAS-dependent and sensitive to ERK-dependent feedback of RAS: these mutants activate ERK by increasing their binding to RAS and, therefore, do not act as independent drivers, but amplify RAS signaling induced by RAS mutation, NF1 loss or activation of receptors (RTKs) [123]. Interestingly, while in melanoma and colorectal cancer class 1 mutants are clearly predominant over the other two types, in lung adenocarcinomas, the three classes of *BRAF* mutants are similarly represented [123]. An optimal strategy for inhibition of class3 BRAF mutants implies the use of MEK inhibitors when these mutations are associated with concomitant *KRAS* mutations or *NF1* loss or drug combinations including inhibitors of RTKs responsible for driving RAS activation when the mutants are not associated with *KRAS* mutations or *NF1* loss [123].

*BRAF*-mutant patients have a prognosis similar to that observed in patients with other genomic abnormalities [124]. However, other studies have indicated that patients with BRAF^V600E^ mutation have shorter overall survival and are resistant to platinum-based chemotherapy [124]. Preclinical data suggest that *BRAF*-mutant NSCLCs are scarcely sensitive to BRAF inhibitors, but are sensitive to MEK inhibitors. Clinical studies have been carried out to evaluate a targeted therapy of *BRAF*-mutant lung cancers. These studies have taken advantage on analogous studies carried out in *BRAF*-mutant melanomas. The clinical studies of targeted therapy of *BRAF*-mutant NSCLC have been performed in BRAF^V600E^-mutant tumors, since this mutation is sensitive to clinically available BRAF inhibitors vemurafenib or dabrafenib. In a phase II study of dabrafenib (2^nd^ generation BRAF inhibitor) plus tramafenib (2^nd^ generation MEK inhibitor) in the treatment of NSCLC patients harboring *BRAF^V600E^* mutations, the objective response rate was 64% and the responses were durable (6 months or longer) and has represented the support for the approval of this treatment regimen in *BRAF^V600E^*-mutant NSCLCs [125-127].

At the moment, the specific therapy of *BRAF^non-V600E^*-mutant NSCLC remains elusive. A recent study showed a distinct dependence on receptor tyrosine kinase signaling between *BRAF^V600E^* and *BRAF^non-V600E^* mutant lung cancer. In *BRAF^V600E^*-mutant lung cancer cells, MEK inhibition achieved sustained suppression of p-ERK, thus indicating that RTKs have a limited role in MAPK activation. In contrast, MEK inhibition leads to feedback activation of RTKs, resulting only in transient suppression of p-ERK in *BRAF^non-V600E^* mutant lung cancers. Particularly, EGFR is the dominant regulator of MAPK signaling in *BRAF^non-V600E^* mutant cells with impaired kinase activity; in these tumors, combined inhibition of MEK and EGFR resulted in tumor shrinkage *in vivo* [128].

The mechanisms of tumor evolution and resistance under effect of treatment with BRAF inhibitors are largely unkown. A recent study based a on the analysis of patient-derived tumor xenografts allowed to conclude that *BRAF* amplification represents an important mechanism of evolution and resistance that can be prevented though an intermittent schedule of administration of RAF, MEK and ERK inhibitors [129].

10. MEK1-Mutated Lung Adenocarcinoma

*MEK1* mutations occur in less than 1% (0.6%) of NSCLCs. The large majority (95%) of these patients are smokers [130]. The most common mutations were K57N (64%) and Q56P (19%), all mutually exclusive with other driver mutations [28]. Transversions G:C>T:A are predominant, in line with smoking-associated DNA damage [130].

As above mentioned, mutationally activated *KRAS* remains an intractable pharmacologic target and *BRAF*-mutant lung adenocarcinomas do not respond to inhibitors of RAF, the definition of effector pathways in these tumors is of crucial importance. In this context, a recent study, using genetically engineered mouse models of *KRAS^G12D^* or *BRAF^V600E^*-induced lung cancer, has provided evidence that in tumor cells developed in these mice a marked MAPK activation was observed [131]. Interestingly, pharmacologic inhibition of MEK1/2 provided evidence that both tumor initiation and maintenance in these models require MAPK activation [131]. The treatment of established tumors with MEK1/2 inhibitors induced regression of the tumors, which, however, regrew after cessation of treatment [131]. These observations suggest that MEK1/2 inhibition used alone or in combination with chemotherapy, may represent a potentially important strategy for targeting *KRAS* and *BRAF* mutant lung cancers [131]. On the basis of these observations a phase 2 randomised study evaluated the impact of selumetinib, an inhibitor of MEK1/MEK2, administered together with docetaxel on the therapy of *KRAS*-mutant advanced NSCLC [132]. Albeit the drug combination increased the number of adverse events, it slightly improved the progression-free survival and resulted in 37% of objective responses [132]. Unfortunately, a recent evaluation of this trial on a very large number of *KRAS*-mutated lung adenocarcinomas showed that selumetinib administered in combination with docetaxel was unable to improve progression free survival compared to docetaxel alone [133].

11. HER2-Mutant Lung Adenocarcinoma

The frequency of *HER2* mutations was assessed in a large cohort of NSCLC, showing that 1.75% of these patients displayed activating mutations of this gene by insertional mutagenesis (insertion of 12 bp at the level of codon 775): these mutations were mutually exclusive with *HER2* gene amplifications [134]. These mutations were more frequent in non-smokers than in smokers and are associated with the development of tumors corresponding to poorly differentiated adenocarcinomas [134]. The overall survival of this molecular subtype is in the same order of magnitude of that of patients with *KRAS* mutations [134]. A second study carried out on a very large cohort of 1275 NSCLCs showed that *HER2* mutations were detected in 4.3% of these patients, mutations being present in adenocarcinomas, but not in squamous cell carcinomas [135]. All these *HER2*-mutated tumors were shown to be negative for other driver mutations [135]. These tumors were particularly observed in younger patients, with no history of smoking and with small tumors [159]. *HER2* amplifications have been observed in about half of the patients with *HER2* mutations [136]. Patients with both *HER2* mutations and amplifications have a negative prognosis [136]. Kim et al explored 321 lung adenocarcinomas and have observed *HER2* mutations in about 2% of patients and *HER2* amplifications in 14% of patients. High *HER2* amplification was a poor prognostic factor for disease-free survival [137]. The Lung Cancer Mutation Consortium screened 920 lung adenocarcinoma patients and observed the occurrence of *HER2* exon 20 insertion mutations in 3% of these patients; one of these patients had a concurrent MET amplification; the majority of HER2-mutated patients were never-smokers and presented with advanced disease at diagnosis [137]. Patients with *HER2* mutations have inferior survival compared with the rest of the cohort of patients with other mutations [137]. Li and coworkers have carefully explored *HER2* mutations and amplifications on lung adenocarcinoma and reached the conclusion that these two abnormalities occur with the same frequency (3%) and do not overlap in the same patients, thus indicating that these two molecular abnormalities are distinct molecular targets [138].

The optimal treatment of *HER2*-mutant lung adenocarcinoma is still an unresolved issue. Case reports and clinical trials have been reported for their population of lung adenocarcinoma patients and suggested that anti-HER2 drugs, such as afatinib, dacomitinib, neratinib and trastuzumab can be associated with anti-tumor response [139, 140]. Recently, a European Network reported the results on the treatment of 101 patients with *HER2*-mutant lung adenocarcinoma: the progression-free survival was comparable for patients treated with chemotherapy compared to those who received *HER2*-targeted therapies (6 months vs 4.8 months) [141]. Wang and coworkers reported in a group of 29 HER2-mutant lung adenocarcinomas a PFS of 5.1 months in patients undergoing treatment with pemetrexed-based chemotherapy [142].

Recently, the oncogenic impact of ERBB2 mutations S310F, S310Y, G309E and insYVMA has been assessed after transfection in NIH3T3 cells. These extracellular mutations determine increased receptor phosphorylation and increased receptor dimerization [167]. The survival of transduced cells was inhibited by small-molecule inhibitors of ERBB2, raising the possibility that patients harboring these mutations could benefit for ERBB2-directed therapy [143].

Particularly interesting are the results of a recent study, the basket trial using the pan-HER kinase inhibitor neratinib (SUMMIT clinical trial). In this trial, patients with different types of *HER2*-mutant tumors were enrolled and the results obtained were exploited to improve our understanding of the factors limiting or favoring the tumor response to HER2-targeting agents [144]. The results of this study showed that the efficacy of this HER2 inhibitor varied as a function of the tumor type and mutant allele to a degree not predictable by preclinical models, with a greatest therapeutic activity observed in breast, cervical and biliary cancers and with tumors that contain kinase domain missense mutations [144]. Concerning lung adenocarcinomas, these tumors are scarcely responsive to the treatment with neratinib, with one objective response on 26 patients: among these patients, insertions in exon 20 predominate and these mutations are largely resistant [145]. Interestingly, the only patients responding to the treatment had a kinase missense mutation L755S [144]. Surprisingly, despite the low response rate, the median PFS was 5.5 months, with six patients remaining under treatment for more than 1 year, a finding which compares favorably with other treatments [144].

Some recent phase I/II clinical studies preliminary evaluated the anti-tumor activity of other HER2 inhibitors. Thus, Ren and coworkers reported the preliminary results on pyrotinib on 11 HER2 mutated NSCLC patients, showing 54% of partial responses [145]. In another study, Li and coworkers reported 33% of objective response rate among 18 *HER2*-mutataed NSCLC patients using ado-trastuzumab emtansine, a HER2 targeted antibody drug conjugate linking trastuzumab with the anti-microtubule agent emtansine [146].

Recently, genetically modified mouse models mimicking individual *HER2* alterations observed in NSCLC were generated and used to identify new drugs active in inhibiting these mutations. Interestingly, this approach showed that osimertinib, a third-generation EGFR inhibitor, showed marked efficacy in HER2-overexpressing and *EGFRdel19/HER2* models, but not in *HER2* exon 20 insertion tumors [147]. However, interestingly, combined treatment with osimertinib and the BET inhibitor JQI significantly inhibited HER2-mutant NSCLCs, while treatment with JQI alone was without efficacy [171]. These observations support the rationale for future clinical trials involving osimertinib either alone or in combination with epigenetic drugs to treat NSCLCs with *HER2* alterations [147].

Finally, a recent study provided evidence that poziotinib is a potent inhibitor of both *EGFR* and *HER2* exon 20 mutations [23]. This compound exerted a marked anti-tumor activity in mouse models driven by *HER2* exon 20 mutants [23]. The clinical activity of poziotinib against *HER2*-mutant NSCLC is under evaluation.

12. Lung Adenocarcinoma with FGFR1 Gene Amplification

Amplifications of the *FGFR1* gene are frequently observed in lung cancer, their frequency being markedly higher in smoker than never smoker patients and were clearly more frequent in squamous cell adenocarcinoma (41.5%) than in adenocarcinoma (14.5%). Importantly, SQCLC cell lines bearing FGFR1 amplification are sensitive to the inhibitory effect of the FGFR inhibitor PD173074 [148]. In line with this observation, FGFR1 knockdown in these cells resulted in cell cytotoxicity, showing that the survival of these tumor cells is dependent upon FGFR1-mediated signaling [148]. Other studies confirmed the lower frequency of FGFR1 amplification in lung adenocarcinoma (15%) than in squamous lung carcinomas [149]; however, surprisingly, FGFR1 expression at protein level was higher in lung adenocarcinoma than in squamous lung carcinoma [150].

13. Lung Adenocarcinoma with Abnormalities of MET Gene

Various mechanisms are responsible for MET activation in lung adenocarcinoma, including *MET* overexpression and genetic aberrations of *MET*, including mutations, amplification and translocation.

Mutations that result in skipping of exon 14 occur in 3-4% of lung adenocarcinomas and 2% of squamous lung carcinomas [151]. *MET*ex14 alterations result in constitutive activity of the mutated receptor and oncogenic transformation activity *in vitro* and *in vivo*. *In vitro* studies showed sensitivity to MET inhibitors in lung tumor cells harboring *MET*ex14 alterations [151]. Awad and coworkers have explored 933 lung adenocarcinoma patients and have reported that 3% of them have a *MET* exon 14 mutation; these patients have a median age of 72.5 years, were predominantly smokers [152]. Patients with exon14 mutations correspond to all four tumor stages: stage IV *MET*-mutated tumors were significantly more likely to have concurrent *MET* genomic amplification and strong c-Met immunocytochemical expression than *MET*-mutated stage I-III tumors or *MET*-WT stage IV tumors [152]. The analysis of concurrent mutations showed that none of the *MET*-mutated tumors displayed activating mutations in *KRAS, EGFR* or *HER2* or rearrangements in *ALK*, *ROS1* or *RET*; inactivating mutations were observed in 29% of cases and amplification of *MDM2* in 46% of cases; *EGFR* copy gain was observed in 29% of cases [152]. Interestingly, *MDM2* amplification was much more frequently associated with *MET*-mutated (46%) than with *KRAS* (0.3%) or *EGFR* (3.4%)-mutated lung adenocarcinomas [175]. Tong et al have explored *MET* abnormalities in 687 treatment-naïve NSCLC patients and observed 2.6% od *MET* mutations in lung adenocarcinomas, while these mutations were absent in squamous lung cell carcinomas; high was the frequency of *MET* mutations in adenosquamous carcinoma (4.8%) and sarcomatoid carcinoma (31.8%) [153]. Both *MET* mutation and *MET* amplification were independent prognostic factors that predicted poorer survival by multivariate analysis [153]. *MET* exon 14 skipping was detected in 1.3% of the Chinese patients with NSCLC (1.9% of never smoking lung adenocarcinoma) [154].

Recently, novel *MET* mutations were reported in some lung adenocarcinoma patients. Thus, Plenker and coworkers identified two *MET* fusion genes (total frequency of about 0.5%) in two stage IV lung adenocarcinomas: *KIF5B-MET* and *STARD3NL-MET*; crizotinib treatment led to a clinical response in these patients [155]. Furthermore, they identified also a MET kinase domain duplication occurring as a resistance mechanism in a patient with a lung carcinoma with an *EML4-ALK* rearrangement undergoing treatment with ceritinib [155].

Mutated MET represents a target for the development of a specific therapy using TKi. Case report studies of patients with lung adenocarcinoma and *MET* exon 14 alterations have documented a clinical response in patients treated with the multikinase inhibitors crizotinib and cabozantinib [reviewed in 156]. In a phase I clinical study the Met inhibitor capmatinib (INC280) was investigated, reporting a partial response in two patients bearing MET-mutated NSCLC (one large cell carcinoma and one squamous lung carcinoma) [151]. Some specific trials designed to evaluate the safety and the therapeutic effects of new MET inhibitors such as tepotinib or MGCD 265 are ongoing and their results are expected soon.

In patients with advanced lung adenocarcinoma who develop resistance to a combinatorial therapy based on gefitinib and a type I MET-TKI, new acquired *MET* mutations have been reported, such as Y1248H and D1246N: the mutations confer resistance to MET TKIs [157].

*MET* amplifications are observed in 21% of NSCLC, while point mutations are rare. *MET* amplifications are potentially oncogenic in that they can drive the proliferation of lung cancer cells. In these patients, amplifications of the *MET* gene were shown to correlate with poor clinical prognosis and increased *MET* gene copy numbers in general were shown to be independently as a negative prognostic factor. Furthermore, activation of the MET-HGF signal pathway is one of the main factors contributing to resistance to therapy with EGFR inhibitors. A recent study evaluated the occurrence of MET amplifications in 693 patients with NSCLC and showed that 39% of them have gains in *MET* gene copy numbers: 3% displayed high levels of *MET* amplification, 6% intermediate gains and 24% low-levels gains [158]. However, while the definition of patients with high levels of amplification is clear and reproducible, the definition of the other two subsets of *MET* amplification is largely based on arbitrary criteria. *MET* amplification is significantly correlated with lymph node metastasis, but not with differentiation or tumor grade [158]. *MET* amplification occurred equally in squamous and lung adenocarcinomas, without or with *EGFR* or *KRAS* mutations [158]. In addition to the *MET* amplifications occurring in untreated lung adenocarcinomas, *MET* amplifications were reported with high frequency in EGFR-mutant lung adenocarcinomas treated with TKIs. In fact, high *MET* overexpression with a high immunoscore index by immunohistochemistry was found in 27% [159] or in 42% [160] of EGFR-mutated lung adenocarcinomas with acquired resistance to EGFR TKI. MET increased gene copy number is an independent negative prognostic factor in surgically resected NSCLC: MET-positive patients have shorter survival than MET-negative patients [161].

Few data are available about the clinical characteristics and outcome of patients with *EGFR*-mutated lung cancers with MET-driven resistance to EGFR TKIs. The optimal treatment of these patients remains to be determined. Preclinical studies and case reports support the view that *MET*-amplified *EGFR*-mutated lung adenocarcinomas are addicted to both EGFR and MET and the combination of both EGFR and MET TKIs is required to overcome resistance mechanisms [162, 163]. Recently, Baldacci et al have reported the outcome of 42 patients with metastatic *EGFR*-mutated NSCLC patients displaying high MET expression or MET amplification, detected after progression on EGFR TKI; in 12 of these patients treated with a MET TKI, only one responding patient was observed; in 2 of these patients treated with a combination of MET and EGFR TKIs, one responding patient was observed [164].

In a phase I clinical study, preliminary evidence was obtained that the MET inhibitor capmatinib exerts anti-tumor activity in patients with *EGFR*-WT NSCLC and with a high level of *MET* amplification [165]. A study of capmatinib+gefitinib in patients with *EGFR*-mutant, *MET*-positive NSCLC reported an overall response rate of 50% in patients with high *MET* copy number [166]. In a study of crizotinib in *MET*-amplified NSCLC, anti-tumor activity was seen in patients with high copy number *MET* amplification [167]. The meta-analysis of seven different studies, enrolling a total of 2577 patients showed that: in the whole population analysis of NSCLC patients, patients who received an additional MET inhibitor did not show significantly improved progression-free survival and overall survival; in the subgroup analysis, patients with MET-high NSCLC tended to show longer survival when treated with an additional MET inhibitor than those in the placebo group [168]. Interestingly, a recent study showed that PDL1 expression in lung adenocarcinomas directly correlated with *MET* amplification [169]. Thus, *MET*-amplified lung adenocarcinomas were characterized by frequent *TP53* mutations and high PDL1 expression [169]. According to these findings, it was suggested that combined therapy with an immune check inhibitor and a MEK inhibitor could be of potential benefit for the treatment of *MET*-amplified lung adenocarcinomas [169].

Recent studies have shown a role of *MET* amplification in osimertinib resistance [170]. Among patients who developed osimertinib resistance, those with *MET* amplification displayed a reduced progression-free survival (3.5 months versus 9.9 months) and overall survival (15.6 months versus 30.7 months) than those without *MET* amplification [171]. Some patients with osimertinib resistance and *MET* amplification responded to the treatment with a first/third generation EGFR TKI and crizotinib [171]. In experimental models, the development of resistance to the 3^rd^ generation EGFR TKI naquotinib is accompanied by *MET* and *NRAS* amplification: interestingly, the association of niquotinib with a MET inhibitor elicited a beneficial effect both on niquotinib-resistant and osimertinib-resistant cells [172].

14. Lung Adenocarcinoma Associated with LKB1 Inactivation

The tumor suppressor gene *LKB1* is inactivated by point mutations or deletions in at least 15-35% of NSCLC and the concurrent KRAS and LKB1 mutation is observed in 5-10% of NSCLC [173]. More recently, using chromogenic *in situ* hybridization the chromosomal status of the *LKB1* locus was assessed in 64 NSLC cases: loss of one copy of chromosome 19p was observed in 62% of cases, while loss of both copies in 28% of cases [174]. In mouse models of *KRAS* mutant-driven lung tumorigenesis, *LKB1* loss cooperated with mutant *KRAS* in promoting lung carcinoma development and stimulated tumor progression and metastasis formation [175]. The stimulatory effect of *LKB1* loss on lung cancer progression and metastasis is promoted through an increased expression of the integrin signaling adaptor protein NEDD9: *NEDD9* silencing inhibited lung tumor progression; in clinical specimens, high NEDD9 expression was associated with malignant progression and metastasis [176]. LKB1 is the major upstream kinase activating the energy-sensing kinase AMPK; LKB1-deficient cells are unable to appropriately sense metabolic stress [177]. Metabolic drugs, such as phenformin, a mitochondrial inhibitor and analog of the diabetes drug metformin selectively induces apoptosis of *LKB1*-deficient tumor cells [177]. Furthermore, this drug prolonged the survival of mice in a model of *KRAS*-mutated and *LKB1*-loss lung cancer [177]. Glutamate dehydrogenase 1 (GDH1), a glutaminolytic enzyme is required for the metastatic activity of *LKB1*-deficient lung adenocarcinomas; interestingly, targeting of GDH1 with specific inhibitors attenuated the metastatic activity of *LKB1*-deficient lung cancers [178]. Studies in primary tumor cells supported the relevance of GDH1 in *LKB1*-deficient lung adenocarcinomas [178]. These observations suggest a therapeutic potential of glutaminolysis inhibitors in *LKB1*-deficient lung adenocarcinomas.

*KRAS*-mutant lung adenocarcinomas can be subdivided according to gene expression into three different clusters; interestingly, cluster 2 (defined as KL cluster) is enriched in *LKB1* mutations, while cluster 3 (defined as KP cluster) is enriched in *TP53* mutations; in the large majority of cases, these two co-occurring mutations, *LKB1* and *TP53*, are mutually exclusive [179]. KL tumors display low levels of immune markers, including PD-L1, while KP tumors are associated with higher levels of somatic mutations, inflammatory markers, immunecheckpoint effector molecules [179]. A clinical trial directly evaluated the response of *KRAS*-mutant lung adenocarcinomas to anti-PD-1/PD-L1 therapy; in KL patients 9% of partial responses and 16% of stable diseases, compared in KP patients to 33% of partial responses and 20% stable diseases, were observed, thus indicating that *LKB1* co-mutations in KRAS-mutant lung tumors predict *de novo* resistance to PD-1/PD-L1 blockade [180].

The study of the *KRAS/LKB1* mouse model of lung adenocarcinoma allowed to determine an important role of LKB1 as a negative regulator of the Focal Adhesion Kinase (FAK) [181]. Treatment of early lung tumors developed in mice *KRAS/LKB1* with FAK pharmacological inhibitors resulted in a striking inhibition of tumor progression, invasion and tumor-associated collagen; chronic treatment improved mice survival [181]. In line with these observations, increased FAK and collagen-associated tumor invasion were detected in human lung adenocarcinomas [181].

Recent studies indicate that *LKB1* inactivation not only accelerates *KRAS* mutant-induced lung adenocarcinoma formation in mice, but drives also a partial lineage switching from the adeno to squamous type [182]. In fact, a stepwise mouse model of lung tumorigenesis indicated that established adenocarcinoma cells can transition to squamous cell fate upon an additional genetic perturbation, such as *LKB1* deletion. Following *LKB1* deletion, de-repression of squamous genes through loss of Polycomb-mediated gene repression was observed [182].

A recent study showed that *KRAS*-mutant *LKB1*-deficient, but not KRAS-mutant TP53-mutant lung adenocarcinoma cells are particularly sensitive to the ChK1 inhibitor that induces killing of these tumor cells in cooperation with the anti-cancer drug gemcitabine [183].

15. KEAP1 Abnormalities in Lung Adenocarcinoma

The transcription factor NRF2 (NF-E2-related factor 2) is a master cell regulator of environmental stress. NRF2 exerts this important function through the induction of detoxification and antioxidant enzymes and block the induction of inflammatory cytokine genes. KEAP1 (Kolch-like ECH-associated protein 1), an adaptor subunit of Cullin-3-based E3 ubiquitin ligase, regulates the activity of NRF2 and acts as a sensor for oxidation and electrophilic stresses. Particularly, NRF2 is anchored in the cytoplasm through binding of KEAP1, which in turn, facilitates the ubiquitination and subsequent degradation of NRF2. *KEAP1* is frequently mutated in lung adenocarcinomas. As above discussed, *KEAP1* mutations are particularly frequent at the level of a tumor subgroup, the unknown mitogenic mutation set [184]. KEAP1 is also frequently mutated (29%) in lung adenocarcinomas characterized by the presence of *KRAS* mutations [185]. Importantly, co-mutation of KRAS and KEAP1 is an independent prognostic factor, predicting shorter overall survival, duration of response to initial platinum-based chemotherapy and survival from the start of immunotherapy, independent of tumor mutational burden [185].

Berger et al have performed a detailed throughput analysis of somatic mutations occurring in lung adenocarcinomas. In this context, they identified and characterized mutations occurring at the level of *KEAP1* and *NRF2* genes. They observed a heterogeneity of *KEAP1* mutations occurring in lung adenocarcinomas, with some mutants retaining KEAP1 activity and two other groups of mutants exhibiting either severe (R470S, R470H, P278S and E117K) or moderate loss-of-function [186]; interestingly, many *KEAP1* mutants observed in lung adenocarcinomas were accompanied by loss-of-heterozygosity, contributing to an additional decrease of KEAP1 activity [186].

Mutations in *KEAP1* cause the aberrant activation of NRF2, which leads to oncogenesis and drug resistance in lung adenocarcinomas. Nuclear NRF2 localization (underlying NRF2 activation) was observed in 18% lung adenocarcinomas and was associated with negative prognosis [187]. A *NRF2*-related gene signature was identified in lung adenocarcinoma samples and higher expression of this signature conferred a poor prognosis in independent cohorts of NSCLC patients [188].

Recently, Frank and coworkers have reported the results of a large genetic screening on 1391 NSCLC patients, showing that *KEAP1* mutations occurred with a frequency of 11.3% and NRF2 mutations with a frequency of 3.5%; both the tumor subtypes mostly affected smokers [189]. Only very rarely these two mutations occurred simultaneously. KEAP1 mutations were more frequent in lung adenocarcinomas, while *NRF2* mutations were more frequent in LSQCC [211]. *TP53* (45% in *KEAP1*-mutant and 41% in *NRF2*-mutant NSCLC) and *KRAS* (40.5% in *KEAP1*-mutant and 22.5% in *NRF2*-mutant NSCLC) were the most frequent co-occurring mutation; *MET* amplification (19% in *KEAP1*-mutant and 26% in *NRF2*-mutant NSCLC) and FGFR1 amplification (6% in *KEAP1*-mutant and 20.6% in *NRF2*-mutant NSCLC) were among the most frequent co-occurring copy number alteration s [189]. The response rate of these patients to systemic treatment was very low [189].

Various lines of evidence indicate that KEAP1 inhibits the response of lung cancer cells to different types of anti-cancer drugs. Thus, Krall and coworkers provided evidence that loss of KEAP1 inhibited the response to BRAF, MEK, EGFR, and ALK inhibition in *BRAF-, NRAS-, KRAS-, EGFR-* and *ALK*-mutant lung cancer cells [173]. Particularly, loss of *KEAP1* abrogated increased production of reactive oxygen species in lung cancer cells exposed to these inhibitors [190].

In mouse models of *KRAS*-driven lung adenocarcinoma, *KEAP1* loss hyperactivates NRF2 and promotes lung tumorigenesis [191]. A core NRF2 signature was derived from 108 *NRF2* target genes: core *NRF2* target genes were significantly upregulated in tumors from advanced-stage disease; furthermore, subjects whose tumors were most associated with NRF2 core signature had significantly poor survival than the rest of patients [191]. Similarly, it was identified a *KEAP1*-mutant signature and tumors more matching this signature were enriched in advanced-stage disease and were associated with a worse prognosis [191]. A genetic screening and metabolomic analyses showed that *KEAP1/NRF2*-driven lung cancers are dependent on increased glutaminolysis and this property can be exploited through the pharmacological inhibition of glutaminase [191]. *KEAP1*-mutant, but not *KEAP1*-WT tumor cells were sensitive to glutaminase inhibitors; pre-treatment with glutamine rescued the sensitivity to glutaminase inhibitor [191]. The ensemble of these observations suggests that glutaminase suppresses the growth of *KEAP1*-mutant lung cancer cells though a block of anaplerosis [191]. Importantly, glutaminase inhibitors strongly decrease the growth of *KEAP1*-mutant lung tumor cells xenografts in mice, but not of *KEAP1*-WT lung cancer cells [191].

The study of a mouse model of *KEAP1*-mutant NSCLC has recently led to the identification of some abnormalities of these tumors potentially amenable to drug treatment. Inactivation of *KEAP1* and *PTEN* in the mouse lung promoted adenocarcinoma formation [214]. Particularly, metabolites identified in the plasma of these double mutant mice supported the view that tumorigenesis is associated with reprogramming of the pentose phosphate pathway [192]. Importantly, the immune milieu was markedly changed by *KEAP1* and *PTEN* deletion, and tumor regression was induced by treatment with immune check inhibitors [192]. Thus, metabolic and immune characteristics can be exploited for the treatment of lung tumors harboring *KEAP1/NRF2* pathway alterations [192].

Proteomic studies have led to the identification of druggable proteins that are selectively expressed bin KEAP1-mutant lung adenocarcinomas [193]. Among the identified proteins, NR0B1 seems to play a relevant role forming a multimeric transcriptional complex regulating the transcriptional activity of *KEAP1*-mutant tumor cells [215]. Interestingly, small molecules able to bind at the level of cysteine residues of the NRB01 protein disrupt NRB01 complexes and inhibit the oncogenic activity of mutant *KEAP1* [193].

These studies indicate the need to develop efficient NRF2 inhibitors suitable first for pre-clinical and then for clinical studies. However, no potent NRF2 inhibitors are clinically available to date. A recent study, based on the screening of a library of 4,000 clinical compounds identified clobetasol propionate as the most potent NRF2 inhibitor; this compound prevented the nuclear accumulation of NRF2 and promoted its degradation [194]. This compound potently inhibited the growth of *KEAP1*-mutant, but not of KEAP1-WT, tumors [194]. In *KEAP1*-mutant lung tumors, clobetasol propionate acted in synergism with rapamycin to strongly inhibit tumor cell growth [194].

Using a high throughput screening of the sensitivity of various lung cancer models to 200,000 chemical compounds, it was discovered that *KEAP1/KRAS* double mutant NSCLC cells were selectively sensitive to the benzothiozole, SW157765, due to the convergent consequences of KRAS and KEAP1-induced regulation of metabolism and of xenobiotic regulatory mechanisms [195]. GLUT8 was identified as a mechanistic target of SW157765 and it was shown that it is selectively required to support the conversion of glucose to serine biosynthesis in these tumor cells [195]. These findings open the way to the development of experimental clinical studies for a targeted therapy of *KEAP1/KRAS* double mutant NSCLCs.

16. Lung Adenocarcinoma with TRAF6 Amplification

Recent studies provided evidence about the frequent (9% of NSCLC) recurring amplification of chromosome 11p13 occurring in non-small-cell lung cancer [218]. Examination of the genes present within the amplified region on 11p13 showed that TNF receptor-associated factor 6 (TRAF6) is a key candidate oncogene [196]. Overexpression and gene inactivation studies have shown that TRAF6 acts as an oncogene inducing NF-kB activation and tumorigenic growth [196]. Particularly, in these models it was shown that RAS requires TRAF6 for its oncogenic activities: TRAF6 expression was usually high in KRAS mutated lung cancers and in >30% of these tumors was amplified [196]. These observations are important because provide a molecular explanation for the need of constitutive NF-kB activation in RAS-driven lung cancers and suggest also a possible therapeutic target for KRAS-mutated lung cancers [196].

References

1. Pao, W.; Miller, V.; Zakowski, M.;  [Doherty, J](https://www.ncbi.nlm.nih.gov/pubmed/?term=Doherty%20J%5BAuthor%5D&cauthor=true&cauthor_uid=15329413).; [Politi, K](https://www.ncbi.nlm.nih.gov/pubmed/?term=Politi%20K%5BAuthor%5D&cauthor=true&cauthor_uid=15329413).; [Sarkaria, I](https://www.ncbi.nlm.nih.gov/pubmed/?term=Sarkaria%20I%5BAuthor%5D&cauthor=true&cauthor_uid=15329413).; [Singh, B](https://www.ncbi.nlm.nih.gov/pubmed/?term=Singh%20B%5BAuthor%5D&cauthor=true&cauthor_uid=15329413).; [Heelan, R](https://www.ncbi.nlm.nih.gov/pubmed/?term=Heelan%20R%5BAuthor%5D&cauthor=true&cauthor_uid=15329413).; [Rusch, V](https://www.ncbi.nlm.nih.gov/pubmed/?term=Rusch%20V%5BAuthor%5D&cauthor=true&cauthor_uid=15329413).; [Fulton, L](https://www.ncbi.nlm.nih.gov/pubmed/?term=Fulton%20L%5BAuthor%5D&cauthor=true&cauthor_uid=15329413).; et al. EGFR gene mutations are common in lung cancers from “never smokers” and are associated with sensitivity to tumors to gefitinib and erlotinib. *Proc. Natl. Acad. Sci. USA* **2004**, 101, 13306-13311.
2. Kerr, K.M.; Dafni, U.; Thunissen, E.; Budendorf, L.; Hager, H., Finn, S.; Biernat, W.; Vliegen, L.; Losa, J.H., Marchetti, A.; et al. Prevalence and clinical association of gene mutations through multiplex mutation testing with NSCLC: results from the ETOP Lungscape project. *Ann. Oncol.* **2018**, 29, 200-208.
3. Aisner, D.L.; Sholl, L.M.; Berry, L.D.; Rossi, M.R.; Chen, H.; Fujiomoto, J.; Moreira, A.L.; Ramalingam, S.S.; rtuzaruz, L.C.; Otterson, G.A.; et al. The impact of smoking and TP53 mutations in lung adenocarcinoma patients with targetable mutations – the Lung Cancer Mutation Consortium (LCMC2). *Clin. Cancer Res.* **2018**, in press.
4. Liao, B.C.; Lin, C.C.; Yang, J.C. Second and third-generation epidermal growth factor receptor tyrosine kinase inhibitors in advanced non small cell lung cancer. *Curr. Opin. Oncol*. **2015**, 27, 94-101.
5. Yang, J.C.; Wu, Y.L.; Schuler, M.; Sebastian, M.; Popat, S.; Yamamoto, N.; Zhou, C.; Hu, C.P.; O'Byrne, K.; Feng, J.; et al. Afatinib versus cisplatin-based chemotherapy for EGFR mutation-positive lung adenocarcinoma (LUX-Lung3 and LUX-Lung6): analysis of overall survival data from two randomized, phase 3 trials. *Lancet Oncol*. **2015**, 16, 141-151.
6. Wu, Y.L.; Cheng, Y.; Zhou, X.; Lee, K.H.; Nakagawa, K.; Niho, S.; Tsuij, F.; Linke, R.; Rosell, R.; Corral, J.; et al. Dacomitinib versus gefitinib as first-line treatment for patients with EGFR-mutation-positive non-small-cell lung cancer (ARCHER 1050): a randomised, open-label, phase 3 trial. *Lancet Oncol.* **2017**, 18, 1454-1466.
7. Janne, P.A.; Yang, J.C.; Kim, D.W., Planchard, D.; Ohe, Y.; Ramalingam, S.S.; Ahn, M.J.; Kim, S.W.; Su, W.C.; et al. AZD9291 in EGFR inhibitor-resistant non-small-cell lung cancer. *N. Engl. J. Med.* **2015**, 372, 1689-1699.
8. Mok, T.S.; Wu, Y.L.; Garassino, M.C.; Kim, H.R.; Ramalingam, S.S.; Shepherd, F.A.; He, Y.; Akamatsu, H.; Theelen, W.S.; et al. Osimertinib or platinum-pemetrexed in EGFR T790M-positive lung cancer. *N. Engl. J. Med.* **2017**, 376, 629-640.
9. Yang, J.C.; Ahn, M.J.; Kim, D.W.; Ramalingam, S.S.; Sequist L.V.; Su, W.C.; Kim, S.W.; Kim, Planchard, D.; Felip, E.; et al. Osimertinib in pretreated T790M-positive advanced non-small-cell lung cancer: AURA study phase II extension component. *J. Clin. Oncol.* **2017**, 35, 1288-1296.
10. Soria, J.C.; Ohe, Y.; Vansteenkiste, J.; Reungwetwattana, T.; Chewaskulyong, B.; Lee, K.H.; Dechaphunkul, A.; Imamura, F.; Nogami, N.; Kurata, T.; et al. Osimertinib in untreated EGFR-mutated advancer non-small-cell lung cancer. *N.Engl. J. Med.* **2018**, 378, 113-125.
11. Oztan, A.; Fischer, S.; Schrock, A.B.; Erlich, R.L.; Lovly, C.M.; Stephens, P.J.; Ross, J.S.; Miller, V.; Ali, S.M.; Ou, S.H.; Raez, L.E. Emergence of EGFR G724S mutation in EGFR-mutant lung adenocarcinoma post progression on osimertinib. *Lung Cancer* **2017**, 111, 84-87.
12. Zhong, W.Z.; Wang, Q.; Mao, W.M.; Xu, S.T.; Wu, L.; Shen, Y.; Yu, Y.; Liu, Y.Y.; Chen, C.; Cheng, Y.; et al. Gefitinib versus vinarelbine plus cisplatin as adjuvant treatment for stage II-IIIA (N1-N2) EGFR-mutant NSCLC (ADJUVANT/CTON G1104): a randomized, open-label, phase 3 study. *Lancet Oncol.* **2018**, 19, 139-148.
13. Kelly, K.; Altorti, N.K., Eberhardt, W.E.E.; et al. Adjuvant erlotinib versus placebo in patients with stage IB-IIIA non-small-cell lung cancer (RADIANT): a randomized, double-blind, phase III trial. *J. Clin. Oncol.* **2015**, 33, 4007-4014.
14. Chaudhuri, A.A.; Chabon, J.J.; Lovejoy, A.F. Early detection of molecular residual disease in localized lung cancer by circulating tumor DNA profiling. *Cancer Discover.* **2018**, in press.
15. Gallant, J.N.; Sheehan, J.H.; Shaver, T.M.; Bailey, M.; Lipson, D.; Chandramohan, R.; Red Brewer, M.; York, S.J.; Kris, M.G.; Pietenpol, J.A.; et al. EGFR kinase domain duplication (EGFR-KDD) is a novel oncogenic driver in lung cancer that is clinically responsive to Afatinib. *Cancer Discover.* **2015**, 5, 1155-1163.
16. Konduri, K.; Gallant, J.N.; Chae, Y.K.; Giles, F.J.; Gitlitz, B.J.; Gowen, K.; Ichihara, E.; Owonikoko, T.K.; Peddareddigari, V.; Ramalingam, S.S.; et al. EGFR fusions as novel therapeutic targets in lung cancer. *Cancer Discover.* **2016**, 6, 601-611.
17. Parry, E.M.; Gable, D.L.; Stanley, S.E.; Khalil, S.E.; Antonescu, V.; Florea, L.; Armonios, M. Germline mutations in DNA repair genes in lung adenocarcinoma. *J. Thorac. Oncol.* **2017**, 12, 1673-1678.
18. Bell, D.W.; Gore, I.; Okimoto, R.A.; Godin-Heymann, N.; Sordella, R.; Mulloy, R.; Sharma, S.V.; Brannigan, B.W.; Mohapatra, G.; Settleman, J.; et al. Inherited susceptibility to lung cancer may be associated with the T790M drug resistance in EGFR. *Nat. Genet.* **2005**, 37, 1315-1316.
19. Hu, Y.; Alden, R.S.; Odegaard, J.I.; Fairclough, S.R.; Chen, R.; Heng, J.; Feeney, N.; Nagy, R.J.; Shah, J.; Ulrich, B.; et al. Discrimination of germline EGFR T790M mutations in plasma cell-free DNA allows study of prevalence across 31,414 cancer patients. *Clin. Cancer Res.* **2017**, 23, 7351-7359.
20. Oxnard, G.R.; Heng, J.C.; Rod, E.J.; Rainville, I.R.; Sable-Hunt, A.L.; Shone-Carson, K.P.; et al. Initial results of a prospective, multicenter trial to study inherited lung cancer risk associated with germline EGFR T790M: INHERIT EGFR. *J. Clin. Oncol.* **2015**, 33, 1505.
21. Arcila, M.E.; Nafa, K.; Chaft, J.E.; Rekhtman, N.; Lau, C.; Reva, B.A.; Zakowski, M.F.; Kris, M.G.; Ladanyi, M. EGFR exon 20 insertion mutations in lung adenocarcinomas: prevalence, molecular heterogeneity, and clinicopathologic characteristics. *Mol. Cancer Ther.* **2013**, 12, 220-229.
22. Kosaka, T.; Tanizaki, J.; Paranal, R.M.; Endoh, H.; Lydon, C.; Capelletti, M.; Repellin, C.E.; Choi, J.; Ogino, A.; Callòes, A.; et al. Response heterogeneity of EGFR and HER2 exon 20 insertions to covalent EGFR and HER2 inhibitors. *Cancer Res.* **2017**, 77, 2712-2721.
23. Robichaux, J.P.; Elamin, Y.Y.; Tan, Z.; Carter, B.W.; Zhang, S.; Liu, S.; Li, S.; Chen, T.; Poteete, A.; Estrada-Bernal, A.; et al. Mechanisms and clinical activity of an EGFR and HER2 exon 20-selective kinase inhibitor in non-small cell lung cancer. *Nat. Med.* **2018,** in press.
24. Blakely, C.M.; Watkins, T.B.K.; Wu, W.; Gini, B,M Chabon, J.J.; McCoach, C.E.; McGranahan, N.; Wilson, G.A.; Birkbak, N.J.; Olivas, V.R.; et al. Evolution and clinical impact of co-occurring genetic alterations in advanced-stage EGFR-mutant lung cancers. *Nat. Genet.* **2017**, 49, 1693-1704.
25. VanderLaan, P.A.; Rangachari, D.; Mockus, S.M.; Spotlow, V.; Reddi, H.V.; Malcolm, J.; Huberman, M.S.; Joseph, L.J.; Kobayashi, S.S.; Costa, D.B. Mutations in TP53, PIK3CA, PTEN and other genes in EGFR mutated lung cancers: correlation with clinical outcomes. *Lung Cancer* **2017**, 106, 17-21.
26. Canale, M.; Petracci, E.; Delmonte, A.; chiadini, E.; Dazzi, C.; Papi, M.; Capelli, L.; Casanova, C.; De Luigi, N.; Mariotti, M.; et al. Impact of TP53 mutations on outcome in EGFR-mutated patients treated with first-line tyrosine kinase inhibitors. *Clin. Cancer Res.* **2017**, 23, 2195-2202.
27. Nahar, R.; Zhai, W.; Takano, A.; Khng, A.J.; Lee, Y.Y.; Lin, C.H.; Koh, T.; Aung, Z.W.; Lim, T.K.; et al. Elucidating the genomic architecture of Asian EGFR-mutant lung adenocarcinoma through multiregion exome sequencing. *Nat. Commun.* **2018**, 9, 216.
28. Sato, S.; Nagahashi, M.; Koike, H.; Ichikawa, H.; Shimada, Y.; Watanabe, S.; Kikuchi, T.; Takada, K.; Nakanishi, R.; Oki, E.; et al. Impact of concurrent genomic alterations detected by comprehensive genomic sequencing on clinical outcomes is East-Asian patients with EGFR-mutated lung adenocarcinoma. *Scient. Rep.* **2018**, 8, 1005.
29. Su, K.Y.; Chen, H.Y.; Li, K.C.;  [Kuo, M.L](https://www.ncbi.nlm.nih.gov/pubmed/?term=Kuo%20ML%5BAuthor%5D&cauthor=true&cauthor_uid=22215752).; [Yang, J.C](https://www.ncbi.nlm.nih.gov/pubmed/?term=Yang%20JC%5BAuthor%5D&cauthor=true&cauthor_uid=22215752).; [Chan, W.K](https://www.ncbi.nlm.nih.gov/pubmed/?term=Chan%20WK%5BAuthor%5D&cauthor=true&cauthor_uid=22215752).; [Ho, B.C](https://www.ncbi.nlm.nih.gov/pubmed/?term=Ho%20BC%5BAuthor%5D&cauthor=true&cauthor_uid=22215752).; [Chang, G.C](https://www.ncbi.nlm.nih.gov/pubmed/?term=Chang%20GC%5BAuthor%5D&cauthor=true&cauthor_uid=22215752).; [Shih, J.Y](https://www.ncbi.nlm.nih.gov/pubmed/?term=Shih%20JY%5BAuthor%5D&cauthor=true&cauthor_uid=22215752).; [Yu, S.L](https://www.ncbi.nlm.nih.gov/pubmed/?term=Yu%20SL%5BAuthor%5D&cauthor=true&cauthor_uid=22215752).; et al. Pretreatment epidermal growth factor receptor (EGFR) T790M mutation predicts shorter EGFR tyrosine kinase inhibitor response duration in patients with non-small-cell lung cancer. *J. Clin. Oncol*. **2012**, 30, 433-440.
30. Hate, A.N.; Niederst, M.; Archibald, H.L.; Gomez-Caraballo, M.; Siddiqui, F.M.; Mulvey, H.E.; Maruvka, Y.E.; Ji, F.; Bhong, H.; Radhakrishina, V.K.; Siravagna, G.; et al. Tumor cells can follow distinct evolutionary paths to become resistant to epidermal growth factor receptor inhibition. *Nat. Med.* **2016**, 22, 262-269.
31. Sequist, L.V.; Waltman, B.A.; Dias-Santagata, D.;  [Digumarthy, S](https://www.ncbi.nlm.nih.gov/pubmed/?term=Digumarthy%20S%5BAuthor%5D&cauthor=true&cauthor_uid=21430269).; [Turke, A.B](https://www.ncbi.nlm.nih.gov/pubmed/?term=Turke%20AB%5BAuthor%5D&cauthor=true&cauthor_uid=21430269).; [Fidias, P](https://www.ncbi.nlm.nih.gov/pubmed/?term=Fidias%20P%5BAuthor%5D&cauthor=true&cauthor_uid=21430269).; [Bergethon, K](https://www.ncbi.nlm.nih.gov/pubmed/?term=Bergethon%20K%5BAuthor%5D&cauthor=true&cauthor_uid=21430269).; [Shaw, A.T](https://www.ncbi.nlm.nih.gov/pubmed/?term=Shaw%20AT%5BAuthor%5D&cauthor=true&cauthor_uid=21430269).; [Gettinger, S](https://www.ncbi.nlm.nih.gov/pubmed/?term=Gettinger%20S%5BAuthor%5D&cauthor=true&cauthor_uid=21430269).; [Cosper, A.K](https://www.ncbi.nlm.nih.gov/pubmed/?term=Cosper%20AK%5BAuthor%5D&cauthor=true&cauthor_uid=21430269).; et al. Genotypic and histological evolution of lung cancers acquiring resistance to EGFR inhibitors. *Science Transl. Med*. **2011**, 3, 75ra26.
32. Niederst, M.J.; Sequist, L.V.; Poirier, J.T.; [Mermel, C.H](https://www.ncbi.nlm.nih.gov/pubmed/?term=Mermel%20CH%5BAuthor%5D&cauthor=true&cauthor_uid=25758528).; [Lockerman, E.L](https://www.ncbi.nlm.nih.gov/pubmed/?term=Lockerman%20EL%5BAuthor%5D&cauthor=true&cauthor_uid=25758528).; [Garcia, A.R](https://www.ncbi.nlm.nih.gov/pubmed/?term=Garcia%20AR%5BAuthor%5D&cauthor=true&cauthor_uid=25758528).; [Katayama, R](https://www.ncbi.nlm.nih.gov/pubmed/?term=Katayama%20R%5BAuthor%5D&cauthor=true&cauthor_uid=25758528).; [Costa, C](https://www.ncbi.nlm.nih.gov/pubmed/?term=Costa%20C%5BAuthor%5D&cauthor=true&cauthor_uid=25758528).; [Ross, K.N](https://www.ncbi.nlm.nih.gov/pubmed/?term=Ross%20KN%5BAuthor%5D&cauthor=true&cauthor_uid=25758528).; [Moran, T](https://www.ncbi.nlm.nih.gov/pubmed/?term=Moran%20T%5BAuthor%5D&cauthor=true&cauthor_uid=25758528).; et al. RB loss in resistant EGFR mutant lung adenocarcinomas that transform to small-cell lung cancer. *Nature Cummun.* **2015**, 6, 6377.
33. Oser, M.G.; Niederst, M.J.; Sequist, L.V.; Engelman, J.A. Transformation from non-small-cell lung cancer to small-cell lung cancer: molecular drivers and cells of origin. *Lancet Oncol.* **2015**, 16, e165-e172.
34. Jacobsen, K.; Bertran-Alamillo, J.; Molina, M.A.; Teixido, C.; Karachaliou, N.; Pedersen, M.H.; Castellvì, J.; Garzon, M.; Codony-Servat, C.; Gimenez-Capitan, A.; et al. Convergent activation drives acquired EGFR inhibitor resistance in lung cancer. *Nat. Commun.* **2017**, 8, 410.
35. Karachaliou, N.; Chaib, I.; Cardona, A.F.; Berenguer, J.; Wilhelmina, J.; Bracht, P.; Yang, J.; Cai, X.; Wang, Z.; Hu, C.; et al. Common co-activation of AXL and CDCP1 in EGFR-mutation-positive non-small cell lung cancer associated with poor prognosis. *EBioMedicine* **2018**, in press.
36. Yamaguchi, T.; Yanagisawa, K.; Sugiyama, R.; [Hosono, Y](https://www.ncbi.nlm.nih.gov/pubmed/?term=Hosono%20Y%5BAuthor%5D&cauthor=true&cauthor_uid=22439932).; [Shimada, Y](https://www.ncbi.nlm.nih.gov/pubmed/?term=Shimada%20Y%5BAuthor%5D&cauthor=true&cauthor_uid=22439932).; [Arima, C](https://www.ncbi.nlm.nih.gov/pubmed/?term=Arima%20C%5BAuthor%5D&cauthor=true&cauthor_uid=22439932).; [Kato, S](https://www.ncbi.nlm.nih.gov/pubmed/?term=Kato%20S%5BAuthor%5D&cauthor=true&cauthor_uid=22439932).; [Tomida, S](https://www.ncbi.nlm.nih.gov/pubmed/?term=Tomida%20S%5BAuthor%5D&cauthor=true&cauthor_uid=22439932).; [Suzuki, M](https://www.ncbi.nlm.nih.gov/pubmed/?term=Suzuki%20M%5BAuthor%5D&cauthor=true&cauthor_uid=22439932).; [Osada, H](https://www.ncbi.nlm.nih.gov/pubmed/?term=Osada%20H%5BAuthor%5D&cauthor=true&cauthor_uid=22439932).; et al. NKX2-1/TITF1/TTF-1-induced ROR1 is required to sustain EGFR survival signaling in lung adenocarcinoma. *Cancer Cell* **2012**, 21, 348-361.
37. Sangodkar, J.; Dhawan, N.; Melville, H.;  [Singh, V.J](https://www.ncbi.nlm.nih.gov/pubmed/?term=Singh%20VJ%5BAuthor%5D&cauthor=true&cauthor_uid=22653055).; [Yuan, E](https://www.ncbi.nlm.nih.gov/pubmed/?term=Yuan%20E%5BAuthor%5D&cauthor=true&cauthor_uid=22653055).; [Rana, H](https://www.ncbi.nlm.nih.gov/pubmed/?term=Rana%20H%5BAuthor%5D&cauthor=true&cauthor_uid=22653055).; [Izadmehr, S](https://www.ncbi.nlm.nih.gov/pubmed/?term=Izadmehr%20S%5BAuthor%5D&cauthor=true&cauthor_uid=22653055).; [Farrington, C](https://www.ncbi.nlm.nih.gov/pubmed/?term=Farrington%20C%5BAuthor%5D&cauthor=true&cauthor_uid=22653055).; [Mazhar, S](https://www.ncbi.nlm.nih.gov/pubmed/?term=Mazhar%20S%5BAuthor%5D&cauthor=true&cauthor_uid=22653055).; [Katz, S](https://www.ncbi.nlm.nih.gov/pubmed/?term=Katz%20S%5BAuthor%5D&cauthor=true&cauthor_uid=22653055).; et al. Targeting the FOXO1/KLF6 axis regulates EGFR signaling and treatment response. *J. Clin. Invest*. **2012**, 122, 2637-2641.
38. Chen, Z.Y.; Zhong, W.Z.; Zhang, X.C.; Su, J.; Yang, X.N.; Chen, Z.H.; Yang, J.J.; Zhou, Q.; Yan, H.H.; An, S.J. ; et al. EGFR mutation heterogeneity and the mixed response of EGFR tyrosine kinase inhibitors of lung adenocarcinomas. *The Oncologist* **2012**, 17, 978-985.
39. Noh, K.W.; Lee, M.S.; Lee, S.E.; Song, J.Y.; Shin, H.T.;b Kim, Y.J.; Oh, D.Y.; Jung, K.; Sung, M.; Kim, M.; et al. Molecular breakdowns: a comprehensive view of anaplastic lymphoma kinase (ALK)-rearranged non-small cell lung cancer. *J. Pathol.* **2017**, 243, 307-319.
40. Soda, M.; Choi, Y.L.; Enomoto, M.;  [Takada, S](https://www.ncbi.nlm.nih.gov/pubmed/?term=Takada%20S%5BAuthor%5D&cauthor=true&cauthor_uid=17625570).; [Yamashita, Y](https://www.ncbi.nlm.nih.gov/pubmed/?term=Yamashita%20Y%5BAuthor%5D&cauthor=true&cauthor_uid=17625570).; [Ishikawa, S](https://www.ncbi.nlm.nih.gov/pubmed/?term=Ishikawa%20S%5BAuthor%5D&cauthor=true&cauthor_uid=17625570).;  [Fujiwara, S](https://www.ncbi.nlm.nih.gov/pubmed/?term=Fujiwara%20S%5BAuthor%5D&cauthor=true&cauthor_uid=17625570).; [Watanabe, H](https://www.ncbi.nlm.nih.gov/pubmed/?term=Watanabe%20H%5BAuthor%5D&cauthor=true&cauthor_uid=17625570).; [Kurashina, K](https://www.ncbi.nlm.nih.gov/pubmed/?term=Kurashina%20K%5BAuthor%5D&cauthor=true&cauthor_uid=17625570).; [Hatanaka, H](https://www.ncbi.nlm.nih.gov/pubmed/?term=Hatanaka%20H%5BAuthor%5D&cauthor=true&cauthor_uid=17625570).; et al. Identification of the transforming EML4-ALK fusion gene in non-small-cell lung cancer. *Nature* **2007**, 448, 561-566.
41. Zhao, F.; Xu, M.; Lei, H.;  [Zhou, Z](https://www.ncbi.nlm.nih.gov/pubmed/?term=Zhou%20Z%5BAuthor%5D&cauthor=true&cauthor_uid=25706305).; [Wang, L](https://www.ncbi.nlm.nih.gov/pubmed/?term=Wang%20L%5BAuthor%5D&cauthor=true&cauthor_uid=25706305).; [Li, P](https://www.ncbi.nlm.nih.gov/pubmed/?term=Li%20P%5BAuthor%5D&cauthor=true&cauthor_uid=25706305).; [Zhao, J](https://www.ncbi.nlm.nih.gov/pubmed/?term=Zhao%20J%5BAuthor%5D&cauthor=true&cauthor_uid=25706305).; [Hu, P](https://www.ncbi.nlm.nih.gov/pubmed/?term=Hu%20P%5BAuthor%5D&cauthor=true&cauthor_uid=25706305). Clinicopathological characteristics of patients with non-small-cell lung cancer who harbor EML4-ALK fusion gene: a meta-analysis. *PLos ONE* **2015**, 10, e0117333.
42. Heuckmann, J.M.; Balke-Whut, H.; Malchers, F.;  [Peifer, M](https://www.ncbi.nlm.nih.gov/pubmed/?term=Peifer%20M%5BAuthor%5D&cauthor=true&cauthor_uid=22912387).; [Sos, M.L](https://www.ncbi.nlm.nih.gov/pubmed/?term=Sos%20ML%5BAuthor%5D&cauthor=true&cauthor_uid=22912387).; [Koker, M](https://www.ncbi.nlm.nih.gov/pubmed/?term=Koker%20M%5BAuthor%5D&cauthor=true&cauthor_uid=22912387).; [Meder, L](https://www.ncbi.nlm.nih.gov/pubmed/?term=Meder%20L%5BAuthor%5D&cauthor=true&cauthor_uid=22912387).; [Lovly, C.M](https://www.ncbi.nlm.nih.gov/pubmed/?term=Lovly%20CM%5BAuthor%5D&cauthor=true&cauthor_uid=22912387).; [Heukamp, L.C](https://www.ncbi.nlm.nih.gov/pubmed/?term=Heukamp%20LC%5BAuthor%5D&cauthor=true&cauthor_uid=22912387).; [Pao, W](https://www.ncbi.nlm.nih.gov/pubmed/?term=Pao%20W%5BAuthor%5D&cauthor=true&cauthor_uid=22912387).; et al. Differential protein stability and ALK inhibitor sensitivity of EML4-ALK fusion variants. *Clin. Cancer Res.* **2012**, 18, 4682-4690.
43. Zhou, J.X.; Yang, H.; Deng, Q.; Gu, X.; He, P.; Lin, Y.; Zhao, M.; Jiang, J.; Chen, H.; Lin, Y.; et al. Oncogenic driver mutations in patients with non-small-cell lung cancer at various clinical stages. *Annals Oncol*. **2013**, 24, 1319-25.
44. Rosenbaum, J.N.; Bloom, R.; Forys, J.T.; Hiken, J.; Armstrong, J.R.; Branson, J.; McNulty, S.; Velu, P.D.; Pepin, K.; Abel, H., et al. Genomic heterogeneity of ALK fusion breakpoints in non-small-cell lung cancer. *Mod. Pathol.* **2018**, in press.
45. Lin, J.L.; Zhu, V.; Yoda, S. ; Yeap, B.Y. ; Schrock, A.B. ; Dagogo-Jack, I. ; Jessopp, N.A. ; Jiang, G.J. ; Le, L.P. ; Gowen, K. ; et al. Impact of SLM4-ALK variant on resistance mechanisms and clinical outcomes in ALK-positive lung cancer. *J.Clin. Oncol.* **2018**, 36, 1199-1206.
46. Solomon, B.J.; Mok, T.; Kim, D.W.;  [Nakagawa, K](https://www.ncbi.nlm.nih.gov/pubmed/?term=Nakagawa%20K%5BAuthor%5D&cauthor=true&cauthor_uid=25470694).; [Mekhail, T](https://www.ncbi.nlm.nih.gov/pubmed/?term=Mekhail%20T%5BAuthor%5D&cauthor=true&cauthor_uid=25470694).; [Felip, E](https://www.ncbi.nlm.nih.gov/pubmed/?term=Felip%20E%5BAuthor%5D&cauthor=true&cauthor_uid=25470694).; [Cappuzzo, F](https://www.ncbi.nlm.nih.gov/pubmed/?term=Cappuzzo%20F%5BAuthor%5D&cauthor=true&cauthor_uid=25470694).; [Paolini, J](https://www.ncbi.nlm.nih.gov/pubmed/?term=Paolini%20J%5BAuthor%5D&cauthor=true&cauthor_uid=25470694).; [Usari, T](https://www.ncbi.nlm.nih.gov/pubmed/?term=Usari%20T%5BAuthor%5D&cauthor=true&cauthor_uid=25470694).; [Iyer, S](https://www.ncbi.nlm.nih.gov/pubmed/?term=Iyer%20S%5BAuthor%5D&cauthor=true&cauthor_uid=25470694).; [Reisman, A](https://www.ncbi.nlm.nih.gov/pubmed/?term=Reisman%20A%5BAuthor%5D&cauthor=true&cauthor_uid=25470694).; et al. First-line crizotinib versus chemotherapy in ALK-positive lung cancer. *N. Engl. J. Med.* **2014**, 371, 2167-2177.
47. Kim, S.; Kim, T.M.; Kim, D.W.;  [Go, H](https://www.ncbi.nlm.nih.gov/pubmed/?term=Go%20H%5BAuthor%5D&cauthor=true&cauthor_uid=23344087).; [Keam, B](https://www.ncbi.nlm.nih.gov/pubmed/?term=Keam%20B%5BAuthor%5D&cauthor=true&cauthor_uid=23344087).; [Lee, S.H](https://www.ncbi.nlm.nih.gov/pubmed/?term=Lee%20SH%5BAuthor%5D&cauthor=true&cauthor_uid=23344087).; [Ku, J.L](https://www.ncbi.nlm.nih.gov/pubmed/?term=Ku%20JL%5BAuthor%5D&cauthor=true&cauthor_uid=23344087). [Chung DH](https://www.ncbi.nlm.nih.gov/pubmed/?term=Chung%20DH%5BAuthor%5D&cauthor=true&cauthor_uid=23344087), [Heo DS](https://www.ncbi.nlm.nih.gov/pubmed/?term=Heo%20DS%5BAuthor%5D&cauthor=true&cauthor_uid=23344087). Heterogeneity of genetic changes associated with acquired crizotinib resistance in ALK-rearranged lung cancer. *J. Thorac. Oncol*. **2013**, 8, 415-22.
48. Wilson, F.H.; Johannesen, C.M.; Piccioni, F.;  [Tamayo, P](https://www.ncbi.nlm.nih.gov/pubmed/?term=Tamayo%20P%5BAuthor%5D&cauthor=true&cauthor_uid=25759024).; [Kim, J.W](https://www.ncbi.nlm.nih.gov/pubmed/?term=Kim%20JW%5BAuthor%5D&cauthor=true&cauthor_uid=25759024).; [Van Allen, E.M](https://www.ncbi.nlm.nih.gov/pubmed/?term=Van%20Allen%20EM%5BAuthor%5D&cauthor=true&cauthor_uid=25759024).;  [Corsello, S.M](https://www.ncbi.nlm.nih.gov/pubmed/?term=Corsello%20SM%5BAuthor%5D&cauthor=true&cauthor_uid=25759024).; [Capelletti, M](https://www.ncbi.nlm.nih.gov/pubmed/?term=Capelletti%20M%5BAuthor%5D&cauthor=true&cauthor_uid=25759024).; [Calles, A](https://www.ncbi.nlm.nih.gov/pubmed/?term=Calles%20A%5BAuthor%5D&cauthor=true&cauthor_uid=25759024).; [Butaney, M](https://www.ncbi.nlm.nih.gov/pubmed/?term=Butaney%20M%5BAuthor%5D&cauthor=true&cauthor_uid=25759024).; et al. A functional landscape of resistance to ALK inhibition in lung cancer. *Cancer Cell* **2015**, 27, 397-408.
49. Lovly, C.M.; McDonald, N.; Chen, H.;  [Ortiz-Cuaran, S](https://www.ncbi.nlm.nih.gov/pubmed/?term=Ortiz-Cuaran%20S%5BAuthor%5D&cauthor=true&cauthor_uid=25173427).; [Heukamp, L.C](https://www.ncbi.nlm.nih.gov/pubmed/?term=Heukamp%20LC%5BAuthor%5D&cauthor=true&cauthor_uid=25173427).; [Yan, Y](https://www.ncbi.nlm.nih.gov/pubmed/?term=Yan%20Y%5BAuthor%5D&cauthor=true&cauthor_uid=25173427).; [Florin, A](https://www.ncbi.nlm.nih.gov/pubmed/?term=Florin%20A%5BAuthor%5D&cauthor=true&cauthor_uid=25173427).; [Ozretić, L](https://www.ncbi.nlm.nih.gov/pubmed/?term=Ozreti%C4%87%20L%5BAuthor%5D&cauthor=true&cauthor_uid=25173427).; [Lim, D](https://www.ncbi.nlm.nih.gov/pubmed/?term=Lim%20D%5BAuthor%5D&cauthor=true&cauthor_uid=25173427).; [Wang, L](https://www.ncbi.nlm.nih.gov/pubmed/?term=Wang%20L%5BAuthor%5D&cauthor=true&cauthor_uid=25173427).; et al. Rationale for co-targeting IGF-1R and ALK in ALK fusion-positive lung cancer. *Nature Med*. **2014**, 20, 1027-1034.
50. Shaw, A.T.; Kim, D.W.; Mehra, R.;  [Tan, D.S](https://www.ncbi.nlm.nih.gov/pubmed/?term=Tan%20DS%5BAuthor%5D&cauthor=true&cauthor_uid=24670165).; [Felip, E](https://www.ncbi.nlm.nih.gov/pubmed/?term=Felip%20E%5BAuthor%5D&cauthor=true&cauthor_uid=24670165).; [Chow, L.Q](https://www.ncbi.nlm.nih.gov/pubmed/?term=Chow%20LQ%5BAuthor%5D&cauthor=true&cauthor_uid=24670165).; [Camidge, D.R](https://www.ncbi.nlm.nih.gov/pubmed/?term=Camidge%20DR%5BAuthor%5D&cauthor=true&cauthor_uid=24670165).; [Vansteenkiste, J](https://www.ncbi.nlm.nih.gov/pubmed/?term=Vansteenkiste%20J%5BAuthor%5D&cauthor=true&cauthor_uid=24670165).; S[harma, S](https://www.ncbi.nlm.nih.gov/pubmed/?term=Sharma%20S%5BAuthor%5D&cauthor=true&cauthor_uid=24670165).; [De Pas, T](https://www.ncbi.nlm.nih.gov/pubmed/?term=De%20Pas%20T%5BAuthor%5D&cauthor=true&cauthor_uid=24670165).; et al. Ceritinib in ALK-rearranged non-small-cell lung cancer. *N. Engl. J. Med*. **2014**, 370, 1189-1197.
51. Gainor, J.F.; Tan, D.S.; De Pas, T.;  [Solomon, B.J](https://www.ncbi.nlm.nih.gov/pubmed/?term=Solomon%20BJ%5BAuthor%5D&cauthor=true&cauthor_uid=25724526).; [Ahmad, A](https://www.ncbi.nlm.nih.gov/pubmed/?term=Ahmad%20A%5BAuthor%5D&cauthor=true&cauthor_uid=25724526).; [Lazzari, C](https://www.ncbi.nlm.nih.gov/pubmed/?term=Lazzari%20C%5BAuthor%5D&cauthor=true&cauthor_uid=25724526).;  [de Marinis, F](https://www.ncbi.nlm.nih.gov/pubmed/?term=de%20Marinis%20F%5BAuthor%5D&cauthor=true&cauthor_uid=25724526).; [Spitaleri, G](https://www.ncbi.nlm.nih.gov/pubmed/?term=Spitaleri%20G%5BAuthor%5D&cauthor=true&cauthor_uid=25724526).; [Schultz, K](https://www.ncbi.nlm.nih.gov/pubmed/?term=Schultz%20K%5BAuthor%5D&cauthor=true&cauthor_uid=25724526).; [Friboulet, L](https://www.ncbi.nlm.nih.gov/pubmed/?term=Friboulet%20L%5BAuthor%5D&cauthor=true&cauthor_uid=25724526).; et al. Progression-free and overall survival in ALK-positive NSCLC patients treated with sequential Crizotinib and Cerotinib. *Clin. Cancer Res*. **2015**, 21, 2745-52.
52. Gainor, J.F.; Dardaei, L.; Yoda, S.; Friboulet, L.; Leschiner, I.; Katayama, R.; Dagogo-Jack, I.; Gadgeel, S.; Schultz, K.; Singh, M.; et al. Molecular mechanisms of resistance to first- and second-generation ALK inhibitors in ALK-rearranged lung cancer. *Cancer Discover.* **2016**, 6, 1118-1133.
53. Soria, J.C.; Tan, D.S.W.; Chiari, R.; Wu, Y.L.; Paz-Perez, L.; Wolf, J.; Geater, S.L.; Orlov, S.; Cortinovis, D.; Yu, C.J.; et al. First-line ceritinib versus platinum-based chemotherapy in advanced ALK-rearranged non-small-cell lung cancer (ASCEND-4): a randomized, open-label, phase 3 study. *Lancet* **2017**, 389, 917-929.
54. Shaw, A.T.; Kim, T.M.; Crinò, L.; Gridelli, C.; Kiura, K.; Liu, G.; Novello, S.; Bearz, A.; Gautschi, O.; Mok, T.; et al. Ceritinib versus chemotherapy in patients with ALK-rearranged non-small-cell lung cancer previously given chemotherapy and crizotinib (ASCEND-5): a randomized, controlled, open-label, phase 3 trial. *Lancet Oncol.* **2017**, 18, 874-886.
55. Hida, T.; Nokihara, H.; Kondo, M.; Kim, Y.H.; Azuma, K.; Seto, S.; Takiguchi, Y.; Nishio, M.; Yoshioka, H.; Imamura, E.; et al. Alectinib versus crizotinib in patients with ALK-positive non-small-cell lung cancer (J-ALEX): an open-label, randomized phase 3 trial. *Lancet* **2017**, 390, 29-39.
56. Peters, S.; Camidge, D.R.; Shaw, A.T.; Gadgeel, S.; Ahn, J.S.; Kim, D.W.; Ou, S.I.; Perol, M.; Dziadziusko, R.; Rosell, R.; et al. Alectinib versus crizotinib in untreated ALK-positive non-small-cell lung cancer. *N. Engl. J. Med.* **2017,** 377, 829-838**.**
57. Novello, S.; Mazieres, J.; Oh, I.J.; deCastro, J.; Migliorino, M.R.; Helland, A.; Dziadziuszko, R.; Griesinger, F.; Kotb, A.; Zeaiter, A.; et al. Alectinib versus chemotherapy in crizotinib-pretreated anaplastic lymphoma kinase (ALK)-positive non-small-cell lung cancer: results from the phase III ALUR study. *Ann. Oncol.* **2018**, in press.
58. Gettingger, S.N.; Bazhenova, L.A.; Langer, C.J.; Salgia, R.; Gold, K.A.; Rosell, R.; Shaw, A.T.; Weiss, G.J.; Tugnait, M. Narasimhan, N.I.; et al. Activity and safety of brigantinib in ALK-rearranged non small-cell lung cancer and other malignancies: a single-arm, open-label, phase 1-2 trial. *Lancet Oncol.* **2016**, 17, 1683-1696.
59. Kim, D.W.; Tiseo, M.; Ahn, M.J.; Reckamp, K.L.; Hansen, K.H.; Kim, S.W.; Huber, R.M.; West, H.L.; Groen, H.J.M.; Hochmair, M.J.; et al. Brigantinib in patients with crizotinib-refractory anaplastic lymphoma kinase-positive non small-cell lung cancer: a randomized, multicenter phase II trial. *J. Clin. Oncol.* **2017**, 35, 2490-2498.
60. Shaw, A.T.; Felip, E.; Bauer, T.M.; Besse, B.; Navarro, A.; Postel-Vinay, S.; Gainor, J.F.; Johnson, M.; Dietrich, J.; James, L.P.; et al. Lorlatinib in non-small-cell lung cancer with ALK or ROS1 rearrangement: an international, multicenter, open-label, single-arm first-in-man phase 1 trial. Lancet Oncol. 2017, 18, 1590-1599.
61. Yoda, S.; Lin, J.J.; Lawrence, M.S.; Burke, B.J.; Friboulet, L.; Langenbucher, A.; Dardaei, L.; Prutisto-Chang, K.; Dagogo-Jack, I.; Timofeevski, S.; et al. Sequential ALK inhibitors can select for Lorlatinib-resistant compound ALK mutations in ALK-positive lung cancer. *Cancer Res.* **2018**, in press.
62. Horn, L.; Infante, J.R.; Reckamp, K.L.; Blumenshein, G.R.; Leal, T.A.; Waqar, S.N.; Gitlitz, B.J.; Sanborn, R.E.; Whisenant, J.G.; Du, L.; et al. Ensartinib (X-396) in ALK-positive non-small cell lung cancer: results from a first-in-human phase I/II, multicenter study. *Clin. Cancer Res.* **2018**, in press.
63. McCoach, C.E.; Le, A.T., Gowan, K.; Jones, K.; Schubert, A.; Doak, A.; Estrada-Bernal, A.; Davies, K.D.; Merrick, D.T.; Bunn, P.A.; et al. The resistance mechanisms to targeted therapies in ROS1^+^ and ALK^+^ non-small cell lung cancer. *Clin. Cancer Res.* **2018**, in press.
64. Dardaei, L.; Wang, H.Q.; Singh, M.; Fordjour, P.; Shaw, K.X.; Yoda, S.; Kerr, G.; Yu, K.; Lieng, J.; Cao, Y.; et al. SHP2 inhibition restores sensitivity in ALK-rearranged non-small-cell lung cancer resistant to ALK inhibitors. *Nat. Med.* **2018**, 24, 512-517.
65. Yun, M.R.; Lim, S.M.; Kim, S.K.; Choi, H.M.; Pyo, K.H.; Kim, S.K.; Lee, J.M.; Choi, J.W.; Kim, H.R.; Hong, M.H.; et al. Enhancer remodeling and microRNA alterations are associated with acquired resistance to ALK inhibitors. *Cancer Res.* **2018**, in press.
66. McCoach, C.E.; Blakely, C.M.; Banks, K.C.; Levy, B.; Chue, B.M.; Raymond, V.M.; Le, A.; Lee, C.E.; Diaz, J.; Wagar, S.N.; et al. Clinical utility of cell-free DNA for the detection of ALK fusions and genomic mechanisms of ALK inhibitor resistance in non-small cell lung cancer. *Clin. Cancer Res.* **2018**, in press.
67. Dagogo-Jack, I.; Brannon, A.R.; Ferris, L.A.; Campbell, C.D.; Lin, J.J.; Schultz, K.R.; Ackil, J.; Stevens, S.; Dardaei, L.; Yoda, S.; et al. Tracking the evolution of resistance to ALK tyrosine kinase inhibitors through longitudinal analysis of circulating tumor DNA. *JCO Precis. Oncol.* **2018**, in press.
68. Bergethon, K.; Shaw, A.T.; Ou, S.H.; [Katayama, R](https://www.ncbi.nlm.nih.gov/pubmed/?term=Katayama%20R%5BAuthor%5D&cauthor=true&cauthor_uid=22215748).; [Lovly, C.M](https://www.ncbi.nlm.nih.gov/pubmed/?term=Lovly%20CM%5BAuthor%5D&cauthor=true&cauthor_uid=22215748).; [McDonald, N.T](https://www.ncbi.nlm.nih.gov/pubmed/?term=McDonald%20NT%5BAuthor%5D&cauthor=true&cauthor_uid=22215748).; [Massion, P.P](https://www.ncbi.nlm.nih.gov/pubmed/?term=Massion%20PP%5BAuthor%5D&cauthor=true&cauthor_uid=22215748).; [Siwak-Tapp, C](https://www.ncbi.nlm.nih.gov/pubmed/?term=Siwak-Tapp%20C%5BAuthor%5D&cauthor=true&cauthor_uid=22215748).; [Gonzalez, A](https://www.ncbi.nlm.nih.gov/pubmed/?term=Gonzalez%20A%5BAuthor%5D&cauthor=true&cauthor_uid=22215748).; [Fang, R](https://www.ncbi.nlm.nih.gov/pubmed/?term=Fang%20R%5BAuthor%5D&cauthor=true&cauthor_uid=22215748).; et al. ROS1 rearrangements define a unique molecular class of lung cancers. *J. Clin. Oncol*. **2012**, 30, 863-870.
69. Davies, K.D.; Le, A.T.; Theodoro, M.; [Skokan, M.C](https://www.ncbi.nlm.nih.gov/pubmed/?term=Skokan%20MC%5BAuthor%5D&cauthor=true&cauthor_uid=22919003).; [Aisner, D.L](https://www.ncbi.nlm.nih.gov/pubmed/?term=Aisner%20DL%5BAuthor%5D&cauthor=true&cauthor_uid=22919003).; [Berge, E.M](https://www.ncbi.nlm.nih.gov/pubmed/?term=Berge%20EM%5BAuthor%5D&cauthor=true&cauthor_uid=22919003).; [Terracciano, L.M](https://www.ncbi.nlm.nih.gov/pubmed/?term=Terracciano%20LM%5BAuthor%5D&cauthor=true&cauthor_uid=22919003).; [Cappuzzo, F](https://www.ncbi.nlm.nih.gov/pubmed/?term=Cappuzzo%20F%5BAuthor%5D&cauthor=true&cauthor_uid=22919003).; [Incarbone, M](https://www.ncbi.nlm.nih.gov/pubmed/?term=Incarbone%20M%5BAuthor%5D&cauthor=true&cauthor_uid=22919003).; [Roncalli, M](https://www.ncbi.nlm.nih.gov/pubmed/?term=Roncalli%20M%5BAuthor%5D&cauthor=true&cauthor_uid=22919003).; et al. Identifing and targeting ROS1 gene fusions in non-small cell lung cancer. *Clin. Cancer Res.* **2012**, 18, 4570-4579.
70. Lee, S. E.; Lee, B.; Hong, M.; Song, J.Y.; Lira, M.E.; Mao, M.; Han, J.; Kim, J.; Yan-La, C. *et al*. Comprehensive analysis of RET and ROS1 rearrangement in lung adenocarcinoma. *Mod. Pathol.* **2015**, 28, 468-479.
71. Li, Z.; Shen, L.; Ding, D.; Huang, J.; Zhang, J.; Chen, Z.; Lu, S. Efficacy of crizotinib among different types of ROS1 fusion partners in patients with ROS1-rearranged non-small-cell lung cancer. *J Thor. Oncol.* **2018**, in press.
72. Wiesweg, M.; Eberhardt, W.; Reis, H.; Ting, S.; Savviudou, N.; Skiba, C.; Herold, T.; Christoph, D.C.; Meiler, J., Worm, K.; Kasper, S.; et al. High prevalence of concomitant oncogene mutations in prospectively identified patients with ROS1-positive metastatic lung cancer. *J. Thorac. Oncol.* **2016**, 1, 54-64.
73. Lin, J.J.; Ritterhouse, L.L.; Ali, S.M.; Bailey, M.; Schrock, A.B.; Gainor, J.F.; Ferris, L.A.; Mino-Kenudson-Kenudson, M.; Miller, V.A.; Iafrate, A.J.; et al. ROS1 fusions rarely overlap with other oncogenic drivers in non-small cell lung cancer. *J. Thorac. Oncol.* **2017**, 12, 872-877.
74. Shaw, A.T.; Ou, S.H.; Bang, Y.J.;  [Camidge, D.R](https://www.ncbi.nlm.nih.gov/pubmed/?term=Camidge%20DR%5BAuthor%5D&cauthor=true&cauthor_uid=25264305).; [Solomon, B.J](https://www.ncbi.nlm.nih.gov/pubmed/?term=Solomon%20BJ%5BAuthor%5D&cauthor=true&cauthor_uid=25264305).; [Salgia, R](https://www.ncbi.nlm.nih.gov/pubmed/?term=Salgia%20R%5BAuthor%5D&cauthor=true&cauthor_uid=25264305).; [Riely, G.J](https://www.ncbi.nlm.nih.gov/pubmed/?term=Riely%20GJ%5BAuthor%5D&cauthor=true&cauthor_uid=25264305).; [Varella-Garcia, M](https://www.ncbi.nlm.nih.gov/pubmed/?term=Varella-Garcia%20M%5BAuthor%5D&cauthor=true&cauthor_uid=25264305).; [Shapiro, G.I](https://www.ncbi.nlm.nih.gov/pubmed/?term=Shapiro%20GI%5BAuthor%5D&cauthor=true&cauthor_uid=25264305).; [Costa, D.B](https://www.ncbi.nlm.nih.gov/pubmed/?term=Costa%20DB%5BAuthor%5D&cauthor=true&cauthor_uid=25264305).; et al. Crizotinib in ROS1-rearranged non-small-cell lung cancer. *N. Engl. J. Med.* **2014**; 37:1963-71.
75. Song, A.; Kim, T.M.; Kim, D.W.; Kim, S.; Keam, B.; Lee, S.H.; Heo, D. Molecular changes associated with acquired resistance to crizotinib in ROS1-rearranged non-small cell lung cancer (NSCLC). *Clin. Cancer Res*. **2015**, 21,2379-87.
76. Katayama, R.; Kobayashi, Y.; Friboulet, L.;  [Lockerman, E.L](https://www.ncbi.nlm.nih.gov/pubmed/?term=Lockerman%20EL%5BAuthor%5D&cauthor=true&cauthor_uid=25351743).; [Koike, S](https://www.ncbi.nlm.nih.gov/pubmed/?term=Koike%20S%5BAuthor%5D&cauthor=true&cauthor_uid=25351743).; [Shaw, A.T](https://www.ncbi.nlm.nih.gov/pubmed/?term=Shaw%20AT%5BAuthor%5D&cauthor=true&cauthor_uid=25351743).; [Engelman, J.A](https://www.ncbi.nlm.nih.gov/pubmed/?term=Engelman%20JA%5BAuthor%5D&cauthor=true&cauthor_uid=25351743).; [Fujita, N](https://www.ncbi.nlm.nih.gov/pubmed/?term=Fujita%20N%5BAuthor%5D&cauthor=true&cauthor_uid=25351743). Cabozantinib overcomes crizotinib resistance in ROS1 fusion-positive cancer. *Clin. Cancer. Res.* **2015**, 21, 166-174.
77. Zou, H.; Li, Q.; Engstrom, L.D.; West, M.; Appleman, V.; Wong, K.A.; McTigue, M.; Deng, Y.L.; Liu, W.; Brooun, A.; et al. PF-06463922 is a potent and selective next-generation ROS1/ALK inhibitor capable of blocking crizotinib-resistant ROS1 mutations. *Proc. Natl. Acad. Sci. USA* **2015**, 112, 3493-3498.
78. Wu, Y.L.; Yang, J.C.; Kim, D.W.; Lu, S.; Zhou, J. ; Seto, T. ; Yang, J.J. ; Yamamoto, N. ; Ahn, M.J. ; Takahashi, T. ; Phase II study of crizotinib in East Asian patients with ROS1-positive advanced non-small-cell lung cancer. *J. Clin. Oncol.* **2018**, 36, 1405-1411.
79. Gainor, J.F.; Tseng, D.; Yoda, S.; Dagogo-Jack, I.; Friboulet, L.; Lin, J.J.; Hubbeling, H.G.; Dardaei, L.; Fargo, A.F.; Schultz, A.R.; et al. Patterns of metastatic spread and mechanisms of resistance to crizotinib in ROS1 -positive non-small-cell lung cancer. *J.C.O. Precis. Oncol.* **2017**, 10.1200.
80. Awad, M.M.; Katayama, R.; Mc Tigue, M.; Liu, W.; Deng, Y.L.; Brooun, A.; et al. Acquired resistance to crizotinib from a mutation in CD74-ROS1. *N. Engl. J. Med.* **2013**, 368, 2395-2401.
81. Drilon, A.; Somwar, R.; Wagner, J.P.; Vellone, N.A.; Eide, C.A.; Zabriskie, M.S. A novel crizotinib-resitant solvent-front mutation responsive to cabozantinib therapy in a patient with ROS1-rearranged lung cancer. *Clin. Cancer Res.* **2016**, 22, 2351-2358.
82. Facchinetti, F.; Loriot, Y.; Kuo, M.S.; Mahjoubi, L.; Lacroix, L.; Planchard, D.; Besse, B.; Farace, F.; Auger, N.; Remon, J.; et al. Crizotinib-resistant ROS1 mutations reveal a predictive kinase inhibitor sensitivity model for ROS1- and ALK-rearranged lung. *Clin. Cancer Res.* **2016**, 22, 5983-5991.
83. Vaishnavi, A.; Schubert, L.; Rix, U.; Marek, L.A.; Le, A.T.; Keysar, S.B.; Glogowska, M.J.; Smith, M.A., Kako, S.; Sumi, N.J.; et al. EGFR mediates responses to small-molecule drugs targeting oncogenic fusion kinases. *Cancer Res.* **2017**, 77, 3551-3563.
84. Drilon, A.; Siena, S.; Ou, S-I-; Patel, M.; Ahn, M.J.; Lee, J.; Bauer, T.M.; Farago, A.F.; Wheler, J.J.; Liu, S.V.; et al. Safety and antitumor activity of the multitargeted pan-TRK, ROS1, and ALK1 inhibitor Entrectinib: combined results from two phase I trials (ALKA-372-001 and STRARK-1). *Cancer Discov.* **2017**, 7, 400-409.
85. Lim, S.M.; Kim, H.R.; Lee, J.S.; Kim, K.H.; Lee, Y.G.; Min, Y.J.; Cho, E.K.; Lee, S.S.; Choi, M.Y.; Shim, H.S.; et al. Open-label, multicenter, phase II study of ceritinib in patients with non-small-cell lung cancer harboring ROS1 rearrangement. *J. Clin. Oncol.* **2017**, 35, 2613-2618.
86. Chen, Y.F.; Hsieh, M.S.; Wu, S.G.; Chang, Y.L.; Yu, C.J.; Yang, J.C.; Yang, P.C.; Shih, J.Y. Efficacy of pemetrexed-based chemotherapy in patients with ROS1 fusion-positive lung adenocarcinoma compared with in patients harboring other driver mutations in East Asian populations. *J. Thorac. Oncol.* **2016**, 11, 1140-1152.
87. Song, Z.; Su, H.; Zhang, Y. Patients with ROS1 rearrangement-positive non-small-cell lung cancer benefit from pemetrexed-based chemotherapy. *Cancer Med.* **2016**, 5, 2688-2693.
88. Zhang, L.; Jiang, T.; Zhao, C.; Li, W.; Li, X.; Zhao, S.; Liu, X.; Jia, Y.; Yang, H.; Ren, S.; Zhou, C. Efficacy of crizotinib and pemetrexed-based chemotherapy in Chinese NSCLC patients with ROS1 rearrangement. *Oncotarget* **2016**, 7, 75145-75154.
89. Lin, J.J.; Shaw, A.T. Recent advances in targeting ROS1 in targeting ROS1 in lung cancer. *J. Thor. Oncol.* **2017**, 12, 1611-1625.
90. Lipson, D.; Capelletti, M.; Yelensky, R.;  [Otto, G](https://www.ncbi.nlm.nih.gov/pubmed/?term=Otto%20G%5BAuthor%5D&cauthor=true&cauthor_uid=22327622).; [Parker, A](https://www.ncbi.nlm.nih.gov/pubmed/?term=Parker%20A%5BAuthor%5D&cauthor=true&cauthor_uid=22327622).; [Jarosz, M](https://www.ncbi.nlm.nih.gov/pubmed/?term=Jarosz%20M%5BAuthor%5D&cauthor=true&cauthor_uid=22327622).; [Curran, J.A](https://www.ncbi.nlm.nih.gov/pubmed/?term=Curran%20JA%5BAuthor%5D&cauthor=true&cauthor_uid=22327622).; [Balasubramanian, S](https://www.ncbi.nlm.nih.gov/pubmed/?term=Balasubramanian%20S%5BAuthor%5D&cauthor=true&cauthor_uid=22327622).; [Bloom, T](https://www.ncbi.nlm.nih.gov/pubmed/?term=Bloom%20T%5BAuthor%5D&cauthor=true&cauthor_uid=22327622).; [Brennan, K.W](https://www.ncbi.nlm.nih.gov/pubmed/?term=Brennan%20KW%5BAuthor%5D&cauthor=true&cauthor_uid=22327622).; et al. Identification of new ALK and RET gene fusions from colorectal and lung cancer biopsies. *Nature Med*. **2012**, 18, 382-384.
91. Wang, R.; Hu, H.; Pan, Y.;  [Li, Y](https://www.ncbi.nlm.nih.gov/pubmed/?term=Li%20Y%5BAuthor%5D&cauthor=true&cauthor_uid=23150706).; [Ye, T](https://www.ncbi.nlm.nih.gov/pubmed/?term=Ye%20T%5BAuthor%5D&cauthor=true&cauthor_uid=23150706).; [Li, C](https://www.ncbi.nlm.nih.gov/pubmed/?term=Li%20C%5BAuthor%5D&cauthor=true&cauthor_uid=23150706).; [Luo, X](https://www.ncbi.nlm.nih.gov/pubmed/?term=Luo%20X%5BAuthor%5D&cauthor=true&cauthor_uid=23150706).; [Wang, L](https://www.ncbi.nlm.nih.gov/pubmed/?term=Wang%20L%5BAuthor%5D&cauthor=true&cauthor_uid=23150706).; [Li, H](https://www.ncbi.nlm.nih.gov/pubmed/?term=Li%20H%5BAuthor%5D&cauthor=true&cauthor_uid=23150706).; [Zhang, Y](https://www.ncbi.nlm.nih.gov/pubmed/?term=Zhang%20Y%5BAuthor%5D&cauthor=true&cauthor_uid=23150706).; et al. RET fusions define a unique molecular and clinicopathologic subtype of non-small-cell lung cancer. *J. Clin. Pathol*. **2012**, 30, 4352-4359.
92. Michels, S.; Scheel, A.H.; Scheffer, M. Clinicopathological characteristics of RET rearranged lung cancer in European patients. *J. Thorac. Oncol.* **2016**, 11, 122-127.
93. Kim, J.O.; Lee, J.; Shin, Y.J. The clinical characteristics of RET rearranged lung adenocarcinoma patients (abstract). *J. Clin. Oncol.* **2015**, 33(suppl.), e18529.
94. Mukhopahyay, S.; Pennell, N.A.; Ali, S.M.; Ross, J.S.; Am, P.C.; Velcheti, V*.* RET-rearranged lung adenocarcinomas with lymphangitic spread, psammoma bodies, and clinical responses to cobanzitinib. *J. Thorac. Oncol*. **2014**, 9, 1714-1719.
95. Drilon, A.; Rekhtman, N.; Arcila, M.; Wang, L.; Ni, A.; Albano, M.; Van Voorthuysen, M.; Somawar, R.; Smith, R.S.; Montecalvo, J.; et al. Cobanzantinib in patients with advanced RET-rearranged non-small-cell lung cancer: an open-label, single-centre, phase 2, single-arm trial. *Lancet Oncol.* **2016**, 17, 1563-1660.
96. Gautschi, O.; Filleron, T.; Wolf, J.; Carbone, D.P.; Owen, D.; Camidge, R.; Narayan, V.; Doebele, R.C.; Besse, B.; Remon-Masip, J.; et al. *J. Clin. Oncol.* **2017**, 35, 1403-1410.
97. Drilon, A.; Hu, Z.I.; Lai, G.G.Y.; Tan, D.S.W. Targeting RET-driven cancers: lessons from evolving preclinical and clinical landscapes. *Nat. Rev. Clin. Oncol.* **2017**, 15, 151-167.
98. Plenker, D.; Riedel, M.; Bragelmann, J.; dammert, M.A.; Chauhan, R.; Knowles, P.P.; Lorenz, C.; Keul, M.; Buhrmann, J.; Pagel, O.; et al. Mechanistic insight into RET kinase inhibitors targeting the DFG-out conformation in RET-rearranged cancer. *Sci. Transl. Med.* **2017**, 9, 10.1126.
99. Nakaoku, T.; Kohno, T.; Araki, M.; Niho, S.; Chauan, R.; Knowles, P.P.; Tsuchinara, K.; Matsumoto, S.; Shimada, Y. Mimaki, S.; et al. A secondary RET mutation in the activation loop conferring resistance to Vandetinib. *Nat. Commun.* **2018**, 9, 625.
100. Subbiah, V.; Gainor, J.F.; Rahal, R.; Brubaker, J.D.; Kim, J.L.; Maynard, M.; Hu, W.; Cao, Q.; Sheets, M.P.; Wilson, D.; et al. Precision targeted therapy with BLU-667 for RET-driven cancers. Cancer Discov. **2018**, in press.
101. Calles, A.; Sholl, L.M.; Rodig, S.J.;  [Pelton, A.K](https://www.ncbi.nlm.nih.gov/pubmed/?term=Pelton%20AK%5BAuthor%5D&cauthor=true&cauthor_uid=25737507).; [Hornick, J.L](https://www.ncbi.nlm.nih.gov/pubmed/?term=Hornick%20JL%5BAuthor%5D&cauthor=true&cauthor_uid=25737507).; [Butaney, M](https://www.ncbi.nlm.nih.gov/pubmed/?term=Butaney%20M%5BAuthor%5D&cauthor=true&cauthor_uid=25737507).; [Lydon, C](https://www.ncbi.nlm.nih.gov/pubmed/?term=Lydon%20C%5BAuthor%5D&cauthor=true&cauthor_uid=25737507).; [Dahlberg, S.E](https://www.ncbi.nlm.nih.gov/pubmed/?term=Dahlberg%20SE%5BAuthor%5D&cauthor=true&cauthor_uid=25737507).; [Oxnard, G.R](https://www.ncbi.nlm.nih.gov/pubmed/?term=Oxnard%20GR%5BAuthor%5D&cauthor=true&cauthor_uid=25737507).; [Jackman, D.M](https://www.ncbi.nlm.nih.gov/pubmed/?term=Jackman%20DM%5BAuthor%5D&cauthor=true&cauthor_uid=25737507).; et al. Immunohistochemical loss of LKB1 is a biomarker for more aggressive biology in KRAS mutant lung adenocarcinoma. *Clin. Cancer Res.* **2015**, 21, 2851-60.
102. Paik, P.K.; Johnson, M.L.; D’Angelo, S.; [Sima, C.S](https://www.ncbi.nlm.nih.gov/pubmed/?term=Sima%20CS%5BAuthor%5D&cauthor=true&cauthor_uid=22605530).; [Ang, D](https://www.ncbi.nlm.nih.gov/pubmed/?term=Ang%20D%5BAuthor%5D&cauthor=true&cauthor_uid=22605530).; [Dogan, S](https://www.ncbi.nlm.nih.gov/pubmed/?term=Dogan%20S%5BAuthor%5D&cauthor=true&cauthor_uid=22605530).; [Miller, V.A](https://www.ncbi.nlm.nih.gov/pubmed/?term=Miller%20VA%5BAuthor%5D&cauthor=true&cauthor_uid=22605530).; [Ladanyi, M](https://www.ncbi.nlm.nih.gov/pubmed/?term=Ladanyi%20M%5BAuthor%5D&cauthor=true&cauthor_uid=22605530).; [Kris, M.G](https://www.ncbi.nlm.nih.gov/pubmed/?term=Kris%20MG%5BAuthor%5D&cauthor=true&cauthor_uid=22605530).; [Riely, G.J](https://www.ncbi.nlm.nih.gov/pubmed/?term=Riely%20GJ%5BAuthor%5D&cauthor=true&cauthor_uid=22605530). Driver mutations determine survival in smokers and never-smokers with stage IIIB/IV lung adenocarcinomas. *Cancer* **2012**, 118, 5840-7.
103. Dogan, S.; Shen, F.; Ang, D.C.; [Johnson, M.L](https://www.ncbi.nlm.nih.gov/pubmed/?term=Johnson%20ML%5BAuthor%5D&cauthor=true&cauthor_uid=23014527).; [D'Angelo, S.P](https://www.ncbi.nlm.nih.gov/pubmed/?term=D%27Angelo%20SP%5BAuthor%5D&cauthor=true&cauthor_uid=23014527).; [Paik, P.K](https://www.ncbi.nlm.nih.gov/pubmed/?term=Paik%20PK%5BAuthor%5D&cauthor=true&cauthor_uid=23014527).; [Brzostowski, E.B](https://www.ncbi.nlm.nih.gov/pubmed/?term=Brzostowski%20EB%5BAuthor%5D&cauthor=true&cauthor_uid=23014527).; [Riely, G.J](https://www.ncbi.nlm.nih.gov/pubmed/?term=Riely%20GJ%5BAuthor%5D&cauthor=true&cauthor_uid=23014527).; [Kris, M.G](https://www.ncbi.nlm.nih.gov/pubmed/?term=Kris%20MG%5BAuthor%5D&cauthor=true&cauthor_uid=23014527).; [Zakowski, M.F](https://www.ncbi.nlm.nih.gov/pubmed/?term=Zakowski%20MF%5BAuthor%5D&cauthor=true&cauthor_uid=23014527).; et al. Molecular epidemiology of EGFR mutations in 3026 lung adenocarcinomas: higher susceptibility of women to smoking-related KRAS mutant cancers. *Clin. Cancer Res.* **2012**, 18, 6169-77.
104. Ohashi, K.; Sequist, L.V.; Arcila, M.E.;  [Lovly, C.M](https://www.ncbi.nlm.nih.gov/pubmed/?term=Lovly%20CM%5BAuthor%5D&cauthor=true&cauthor_uid=23515407).; [Chen, X](https://www.ncbi.nlm.nih.gov/pubmed/?term=Chen%20X%5BAuthor%5D&cauthor=true&cauthor_uid=23515407).; [Rudin, C.M](https://www.ncbi.nlm.nih.gov/pubmed/?term=Rudin%20CM%5BAuthor%5D&cauthor=true&cauthor_uid=23515407).; [Moran, T](https://www.ncbi.nlm.nih.gov/pubmed/?term=Moran%20T%5BAuthor%5D&cauthor=true&cauthor_uid=23515407).; [Camidge, D.R](https://www.ncbi.nlm.nih.gov/pubmed/?term=Camidge%20DR%5BAuthor%5D&cauthor=true&cauthor_uid=23515407).; [Vnencak-Jones, C.L](https://www.ncbi.nlm.nih.gov/pubmed/?term=Vnencak-Jones%20CL%5BAuthor%5D&cauthor=true&cauthor_uid=23515407).; [Berry, L](https://www.ncbi.nlm.nih.gov/pubmed/?term=Berry%20L%5BAuthor%5D&cauthor=true&cauthor_uid=23515407).; et al. Characteristics of lung cancer harboring NRAS mutations. *Clin. Cancer Res.* **2013**,19, 2584-2591.
105. Manchado, E.; Weissmueller, S.; Morris, J.P.; Chen, C.C.; Wullenkord, R.; Lujanubio, A.; de Stanchina, E.; Poirier, J.T.; Gainor, J.F.; Carcoran, R.B.; et al. A combinatorial strategy for treating KRAS-mutant lung cancer. *Nature* **2016**, 534, 647-651.
106. Sanclemente, M.; Francor, S.; Esteban-Burgos, L.; Bousquet-Mur, E.; Djrac, M.; Lopez-Casas, P.; Hidalgo, M.; Guerra, C.; Drosten, M.; Musteanu, M.; et al. c-RAF ablation induces regression of advanced KRas/Trp53 mutant lung adenocarcinomas by a mechanism independent of MAPK signaling. *Cancer Cell* **2018**, 33, 217-228.
107. Ambrogio, C.; Kohler, J.; Zhou, Z.W.; Wang, H.; Paranal, R.; Li, J.; Capelletti, M.; Caffara, C.; Li, S.; Lv, Q.; et al. KRAS dimerization impacts MEK inhibitor sensitivity and oncogenic activity of mutant KRAS. *Cell* **2018**, 172, 857-868.
108. Hayashi, T.; Desmeules, P.; Smith, R.S.; Drilon, A.; Somwar, R.; Lodanyi, M. RASA1 and NF1 are preferentially co-mutated and define a distinct genetic subset of smoking-associated non-small cell lung carcinomas sensitive to MEK inhibition. *Clin. Cancer Res.* **2018,** 24, 1436-1447.
109. Wang, R.; Wang, L.; Li, Y.;  [Hu, H](https://www.ncbi.nlm.nih.gov/pubmed/?term=Hu%20H%5BAuthor%5D&cauthor=true&cauthor_uid=24850843).; [Shen, L](https://www.ncbi.nlm.nih.gov/pubmed/?term=Shen%20L%5BAuthor%5D&cauthor=true&cauthor_uid=24850843).; [Shen, X](https://www.ncbi.nlm.nih.gov/pubmed/?term=Shen%20X%5BAuthor%5D&cauthor=true&cauthor_uid=24850843).; [Pan, Y](https://www.ncbi.nlm.nih.gov/pubmed/?term=Pan%20Y%5BAuthor%5D&cauthor=true&cauthor_uid=24850843).; [Ye, T](https://www.ncbi.nlm.nih.gov/pubmed/?term=Ye%20T%5BAuthor%5D&cauthor=true&cauthor_uid=24850843).; [Zhang, Y](https://www.ncbi.nlm.nih.gov/pubmed/?term=Zhang%20Y%5BAuthor%5D&cauthor=true&cauthor_uid=24850843).; [Luo, X](https://www.ncbi.nlm.nih.gov/pubmed/?term=Luo%20X%5BAuthor%5D&cauthor=true&cauthor_uid=24850843).; et al. FGFR1/ tyrosine kinase fusions define a unique molecular subtype of non-small cell lung cancer. *Clin. Cancer Res*. **2014**, 20, 4107-4114.
110. Capelletti, M.; Dodge, M.E.; Ercan, D.; Hammerman, P.S.; Park, S.I.; Kim, J.; Sasaki, H.; Jablons, D.M.; Lipson, D.; Young, L.; et al. Identification of recurrent FGFR3-TACC3 fusion oncogenes from lung adenocarcinoma. *Clin. Cancer Res.* **2014**, 20, 6551-6558.
111. Chandrani, P.; Prabhash, K.; Prasad, R.; Sethunath, V.; Ranjar, M.; Iyer, P.; Aich, J.; Dhamne, H.; Iyer, D.N.; Upadhay, P.; et al. Drug-sensitive FGFR3 mutations in lung adenocarcinoma. *Annal. Oncol.* **2017**, 28, 597-603.
112. Imielinski, M.; Berger, A.H.; Hammerman, P.S.; Hernandez, B.; Pugh, T.J.; Hodis, E.; Cho, J.; Suh, J.; Capelletti, M.; Sivachenko, A.; et al. Mapping the hallmarks of lung adenocarcinoma with massively parallel sequencing. *Cell* **2012**; 150, 1107-1120.
113. Chaft, J.E.; Arcila, M.E.; Paik, P.; Lou, C.; Riely, G.; Pietanza, C.; Zakowski, M.; Rusch, V.; Sima, C.S.; Ladanyi, M.; et al. Coexistence of PIK3CA and of the oncogene mutation in LUADS-rationale for comprehensive mutation profiling. *Mol. Cancer Ther.* **2011**, 11, 485-491.
114. Wang, L.; Hu, H.; Pan, Y.; Wang, R.; Li, Y.; Shen, L.; Yu, Y.; Li, H.; Cai, D.; Sun, Y.; et al. PI3KCA mutations frequently coexist with EGFR/KRAS mutations in non-small cell lung cancer and suggest poor prognosis in EGFR/KRAS wild-type subgroup. *PLOS ONE* **2014**, 9, e88291.
115. Wu, S.G.; Chang, Y.L.; Yu, C.J.; Yang, P.C.; Shih, J.Y. The role of PI3KCA mutations among lung adenocarcinoma patients with primary and acquired resistance to EGFR tyrosine kinase inhibition. *Scient. Rep.* **2016**, 6, 35249.
116. Villartuz, L.C.; Socinski, M.A.; Abberbock, S.;  [Berry, L.D](https://www.ncbi.nlm.nih.gov/pubmed/?term=Berry%20LD%5BAuthor%5D&cauthor=true&cauthor_uid=25273224).; [Johnson, B.E](https://www.ncbi.nlm.nih.gov/pubmed/?term=Johnson%20BE%5BAuthor%5D&cauthor=true&cauthor_uid=25273224).; [Kwiatkowski, D.J](https://www.ncbi.nlm.nih.gov/pubmed/?term=Kwiatkowski%20DJ%5BAuthor%5D&cauthor=true&cauthor_uid=25273224).; [Iafrate, A.J](https://www.ncbi.nlm.nih.gov/pubmed/?term=Iafrate%20AJ%5BAuthor%5D&cauthor=true&cauthor_uid=25273224).; [Varella-Garcia, M](https://www.ncbi.nlm.nih.gov/pubmed/?term=Varella-Garcia%20M%5BAuthor%5D&cauthor=true&cauthor_uid=25273224).; [Franklin, W.A](https://www.ncbi.nlm.nih.gov/pubmed/?term=Franklin%20WA%5BAuthor%5D&cauthor=true&cauthor_uid=25273224).; [Camidge, D,R](https://www.ncbi.nlm.nih.gov/pubmed/?term=Camidge%20DR%5BAuthor%5D&cauthor=true&cauthor_uid=25273224).; et al. Clinicopathologic features and outcomes of patients with lung adenocarcinomas harboring BRAF mutations in the lung cancer mutation consortium. *Cancer* **2014**, 121, 448-56.
117. Salimian, K.J.; Fazeli, R.; Zheng, G.; Ettinger, D.; Maleki, Z. V600E BRAF versus non-V600E BRAF mutated lung adenocarcinomas: cytomorphology, coexistence of other driver mutations and patient characetristics. *Acta Cytol.* **2018**, 62, 79-84.
118. Nguyen-Nyac, T.; Bouchaub, H.; Adje, A.A.; Peters, S. BRAF alterations as therapeutic targets in non-small-cell lung cancer. *J. Thorac. Oncol.* **2015**, 10, 1396-1403.
119. Ilei, P.B.; Belchis, D.; Tseng, L.H.; Nguyen, D.; De Marchi, F.; Haley, L.; Riel, S.; Beierl, K.; Zheng, G.; Brahmer, J.R.; et al. Clinical mutational profiling of 1006 lung cancers by next generation sequencing. *Oncotarget* **2017**, 8, 96684-96696.
120. Chang, M.T. Identifying recurrent mutations in cancer reveals widespread lineage diversity and mutational specificity. *Nat. Biotechnol.* **2016**, 34, 155-163.
121. Carter, J. A retrospective identified BRAF^D594G^ as the most frequent BRAF alteration occurring in lung adenocarcinoma patientys. *Am. J. Pathol.* **2015**, 144, 620-628.
122. Nieto, P.; Ambrogio, C.; Esteban-Burgos, L.; Gomez-Lopez, G.; Blasco, M.T.; Yao, Z.; Marais, R.; Rosen, N.; Chiarle, R.; Pisano, D.; et al. Braf kinase-inactive mutant induces lung adenocarcinoma. *Nature* **2017**, 548, 239-243.
123. Yao, Z.; Yager, R.; Rodrik-Outmezguine, V.; Tao, A.; Torres, N.; Chang, M.; Drosten, M.; Zhao, H.; Cecchi, F.; Hembrough, T.; et al. Tumours with class 3 BRAF mutants are sensitive to the inhibition of activated RAS. *Nature* **2017**, 548, 234-238.
124. Marchetti, A.; Felicioni, L.; Malatesta, S.; Grazia Sciarrotta, M.; Guetti, L.; Chella, A. Clinical features and outcome of patients with non-small-cell lung cancer harboring BRAF mutations. *J. Clin. Oncol.* **2011**, 29, 3574-3579.
125. Planchard, D.; Kim, T.M.; Mazieres, J.; Quoix, E.; Riely, G.; Barlesi, F.; Souquet, P.J.; Smit, E.F.; Groen, H.J.; Kelly, R.J. et al. Dabrafenib in patients with BRAF(V600E)-positive advanced non-small-cell lung cancer: a single-arm, multicenter, open-label, phase 2 trial. *Lancet Oncol.* **2016**, 17, 642-650.
126. Planchard, D.; Besse, B.; Green,H.J.M.; Souquet, P.J.; Quoix, E.; Baik, C.S.; Barlesi, F.; Kim, T.M.; Mazieres, J.; Novello, S., et al. Dabrafenib plus trimetinib in patients with previously treated BTAF(V600E)-mutant metastatic non-small cell lung cancer: an open-label, multicenter phase 2 trial. *Lancet Oncol.* **2016**, 17, 984-993.
127. Planchard, D.; Smit, E.F.; Groen, H.J.M.; Mazieres, J.; Besse, B.; Helland, A.; Giannone, V.; D’Amelio, A.M.; Zhang, P.; Mookerjee, B.; et al. Debrafenib plus trametinib in patients with previously untreated BRAF<sup>V600E</sup>mutant metastatic non-small-cell lung cancer: an open-label, phase 2 trial. *Lancet Oncol.* **2017**, 18, 1307-1316.
128. Kotani, H.; Adachi, Y.; Kitai, H.; Tomida, S.; Baudo, H.; Faber, A.C.; Yoshino, T.; Voon, D.C.; Yano, S.; Ebi, H. Distinct dependencies on receptor tyrosine kinases in the regulation of MAPK signaling between BRAF V600E and non-V600E mutant. *Oncogene* **2018**, in press.
129. Xue, Y.; Martellotto, L.; Baslan, T.; Vides, A.; Solomon, M.; Mai, T.T.; Chaudhary, N.; Riely, G.J.; Li, B.T.; Scott, K.; et al. An approach to suppress the evolution of resistance in BRAF^V600E^-mutant cancer. *Nat. Med.* **2017**, 23, 929-937.
130. Arcila, M.E.; Drilan, A.; Sylvester, B.;  [Lovly, C.M](https://www.ncbi.nlm.nih.gov/pubmed/?term=Lovly%20CM%5BAuthor%5D&cauthor=true&cauthor_uid=25351745).; [Borsu, L](https://www.ncbi.nlm.nih.gov/pubmed/?term=Borsu%20L%5BAuthor%5D&cauthor=true&cauthor_uid=25351745).; [Reva, B](https://www.ncbi.nlm.nih.gov/pubmed/?term=Reva%20B%5BAuthor%5D&cauthor=true&cauthor_uid=25351745).; [Kris, M.G](https://www.ncbi.nlm.nih.gov/pubmed/?term=Kris%20MG%5BAuthor%5D&cauthor=true&cauthor_uid=25351745).; [Solit, D.B](https://www.ncbi.nlm.nih.gov/pubmed/?term=Solit%20DB%5BAuthor%5D&cauthor=true&cauthor_uid=25351745).; [Ladanyi, M](https://www.ncbi.nlm.nih.gov/pubmed/?term=Ladanyi%20M%5BAuthor%5D&cauthor=true&cauthor_uid=25351745). MAP2K1 (MEK1) mutations define a distinct subset of lung adenocarcinoma associated with smoking. *Cancer Res.* **2015**, 21, 1935-43.
131. Trejo, C.L.; Juan, J.; Vicent, S.;  [Sweet-Cordero, A](https://www.ncbi.nlm.nih.gov/pubmed/?term=Sweet-Cordero%20A%5BAuthor%5D&cauthor=true&cauthor_uid=22511580).; [McMahon, M](https://www.ncbi.nlm.nih.gov/pubmed/?term=McMahon%20M%5BAuthor%5D&cauthor=true&cauthor_uid=22511580). MEK1/2 inhibition elicits regression of autochthonous lung tumors induced by KRAS^G412D^ or BRAF^V600E^. *Cancer Res*. **2012**, 72, 3048-3059.
132. Janne, P.A.; Shaw, A.T.; Pereira, J.R.; [Jeannin, G](https://www.ncbi.nlm.nih.gov/pubmed/?term=Jeannin%20G%5BAuthor%5D&cauthor=true&cauthor_uid=23200175).; [Vansteenkiste, J](https://www.ncbi.nlm.nih.gov/pubmed/?term=Vansteenkiste%20J%5BAuthor%5D&cauthor=true&cauthor_uid=23200175).; [Barrios, C](https://www.ncbi.nlm.nih.gov/pubmed/?term=Barrios%20C%5BAuthor%5D&cauthor=true&cauthor_uid=23200175).; [Franke, F.A](https://www.ncbi.nlm.nih.gov/pubmed/?term=Franke%20FA%5BAuthor%5D&cauthor=true&cauthor_uid=23200175).; [Grinsted, L](https://www.ncbi.nlm.nih.gov/pubmed/?term=Grinsted%20L%5BAuthor%5D&cauthor=true&cauthor_uid=23200175).; [Zazulina, V](https://www.ncbi.nlm.nih.gov/pubmed/?term=Zazulina%20V%5BAuthor%5D&cauthor=true&cauthor_uid=23200175).; [Smith, P](https://www.ncbi.nlm.nih.gov/pubmed/?term=Smith%20P%5BAuthor%5D&cauthor=true&cauthor_uid=23200175).; et al. Selumetinib plus docetaxel for KRAS-mutant advanced non-small-cell lung cancer: a randomized, multicenter, placebo-controlled, phase 2 study. *Lancet Oncol*. **2013**, 14, 38-47.
133. Janne, P.A.; van den Heuvel, M.M.; Barlesi, F.; Cobo, M.; Mazieres, J.; Crinò, L.; Orlov, S.; Blackhall, F.; Wolf, J.; Garrido, P.; et al. Selumetinib plus docetaxel compared with docetaxel alone and progression-free survival in patients with KRAs-mutant advanced non-small cell lung cancer: the SELECT-1 randomized clinical trial. *JAMA* **2017**, 317, 1844-1853.
134. Arcila, M.; Chaft, J.; Nafa, K.;  [Roy-Chowdhuri, S](https://www.ncbi.nlm.nih.gov/pubmed/?term=Roy-Chowdhuri%20S%5BAuthor%5D&cauthor=true&cauthor_uid=22761469).; [Lau, C](https://www.ncbi.nlm.nih.gov/pubmed/?term=Lau%20C%5BAuthor%5D&cauthor=true&cauthor_uid=22761469).; [Zaidinski, M](https://www.ncbi.nlm.nih.gov/pubmed/?term=Zaidinski%20M%5BAuthor%5D&cauthor=true&cauthor_uid=22761469).; [Paik, P.K](https://www.ncbi.nlm.nih.gov/pubmed/?term=Paik%20PK%5BAuthor%5D&cauthor=true&cauthor_uid=22761469).; [Zakowski, M.F](https://www.ncbi.nlm.nih.gov/pubmed/?term=Zakowski%20MF%5BAuthor%5D&cauthor=true&cauthor_uid=22761469).; [Kris, M.G](https://www.ncbi.nlm.nih.gov/pubmed/?term=Kris%20MG%5BAuthor%5D&cauthor=true&cauthor_uid=22761469).; [Ladanyi, M](https://www.ncbi.nlm.nih.gov/pubmed/?term=Ladanyi%20M%5BAuthor%5D&cauthor=true&cauthor_uid=22761469). Prevalence, clinicopathologic associations, and molecular spectrum of ERBB2 (HER2) tyrosine kinase mutations in lung adenocarcinomas. *Clin. Cancer Res.* **2012**, 18, 4910-4918.
135. Suzuki, M.; Shiraishi, K.; Yoshida, A.;  [Shimada, Y](https://www.ncbi.nlm.nih.gov/pubmed/?term=Shimada%20Y%5BAuthor%5D&cauthor=true&cauthor_uid=25468202).; [Suzuki, K](https://www.ncbi.nlm.nih.gov/pubmed/?term=Suzuki%20K%5BAuthor%5D&cauthor=true&cauthor_uid=25468202).; [Asamura, H](https://www.ncbi.nlm.nih.gov/pubmed/?term=Asamura%20H%5BAuthor%5D&cauthor=true&cauthor_uid=25468202).; [Furuta, K](https://www.ncbi.nlm.nih.gov/pubmed/?term=Furuta%20K%5BAuthor%5D&cauthor=true&cauthor_uid=25468202).; [Kohno, T](https://www.ncbi.nlm.nih.gov/pubmed/?term=Kohno%20T%5BAuthor%5D&cauthor=true&cauthor_uid=25468202).; [Tsuta, K](https://www.ncbi.nlm.nih.gov/pubmed/?term=Tsuta%20K%5BAuthor%5D&cauthor=true&cauthor_uid=25468202). HER2 gene mutations in non-small cell lung carcinomas: concurrence with HER2 gene amplification and Her2 protein expression and phosphorylation. *Lung Cancer* **2015**, 87, 14-22.
136. Kim, E.K.; Kim, K.A.; Lee, C.Y.; Shim, H.S. The frequency and clinical impact of HER2 alterations in lung adenocarcinoma. *PLoS ONE* **2017**, 12, e0171280.
137. Pillai, R.N.; Bahera, M.; Berry, L.D.; Rossi, M.R.; Kris, M.G.; Johnson, B.E.; bunn, P.A.; Ramalingam, S.S.; Khuri, F.R. HER2 mutations in lung adenocarcinomas: a report from the Lung Cancer Mutation Consortium. *Cancer* **2017**, 123, 4099-4105.
138. Li, B.; Ross, D.S.; Aisner, D.L.; Chaft, J.E.; Hsu, M.; Kako, S.; Kris, M.G.; Varella-Garcia, M.; Arcila, M.E. HER2 amplification and HER2 mutation are distinct molecular targets in lung cancer. *J. Thorac. Oncol.* **2015**, 11, 414-419.
139. Mazieres, J.; Peters, S.; Lepage, B.; Cortot, A.B.; Barlesi, F.; Beau-Faller, M.; Besse, B.; Blons, H.; Mausuet-lupo, A.; Urban, T.; et al. Lung cancer that harbors an HER2 mutation: epidemiologic characteristics and therapeutic perspectives. *J. Clin. Oncol.* **2013**, 31, 1997-2003.
140. Kris, M.G.; Camidge, D.R.; Giaccone G. Targeting HER2 aberrations as actionable drivers in lung cancers: phase II trial of the pan-HER tyrosine kinase inhibitor dacatomitinib in patients with HER2-mutant or amplified tumors. *Ann. Oncol.* **2015**, 26, 1421-1427.
141. Mezieres, J.; Barlesi, F.; Filleron, T.; Besse, B.; Monnet, I.; Beau-Faller, M.; Peters, S.; Dausin, E.; Fruh, M.; Pless, M.; et al. Lung cancer patients with HER2 mutations treated with chemotherapy and HER2-targets drugs: results from the European EUHER2 cohort. *Ann. Oncol.* **2016**, 27, 281-286.
142. Wang, Y.; Zhang, S.; Wu, F.; Zhao, J.; Li, X.; Zhao, C.; Ren, S.; Zhou, C. Outcomes of pemetrexed-based chemotherapies in HER2-mutant lung cancers. *BMC Cancer* **2018**, 18, 326.
143. Greulich, H.; Kaplan, B.; Mertins, P.; [Chen, T.H](https://www.ncbi.nlm.nih.gov/pubmed/?term=Chen%20TH%5BAuthor%5D&cauthor=true&cauthor_uid=22908275).; [Tanaka, K.E](https://www.ncbi.nlm.nih.gov/pubmed/?term=Tanaka%20KE%5BAuthor%5D&cauthor=true&cauthor_uid=22908275).; [Yun, C.H](https://www.ncbi.nlm.nih.gov/pubmed/?term=Yun%20CH%5BAuthor%5D&cauthor=true&cauthor_uid=22908275).; [Zhang, X](https://www.ncbi.nlm.nih.gov/pubmed/?term=Zhang%20X%5BAuthor%5D&cauthor=true&cauthor_uid=22908275).; [Lee, S.H](https://www.ncbi.nlm.nih.gov/pubmed/?term=Lee%20SH%5BAuthor%5D&cauthor=true&cauthor_uid=22908275).; [Cho, J](https://www.ncbi.nlm.nih.gov/pubmed/?term=Cho%20J%5BAuthor%5D&cauthor=true&cauthor_uid=22908275).; [Ambrogio, L](https://www.ncbi.nlm.nih.gov/pubmed/?term=Ambrogio%20L%5BAuthor%5D&cauthor=true&cauthor_uid=22908275).; et al. Functional analysis of receptor tyrosine kinase mutations in lung cancer identifies oncogenic extracellular domain mutations of ERBB2. *Proc. Natl. Acad. Sci. USA* **2012**, 109, 14476-14481.
144. Hyman, D.M.; Piha-Paul, S.; Won, H.; Radan, J.; Saura, C.; Shapiro, G.I.; Juric, D.; Quinn, D.I.; Moreno, V.; Doger, B.; et al. HER kinase inhibition in patients with HER2- and HER-3-mutant cancers. *Nature* **2018**, 554, 189-194.
145. Ren, S.; Zhou, C.; Gao, G.; Su, C.; Chen, X.; Wu, F.Y.; Li, X.; Zhao, C.; Cai, W. Preliminary results of a phase II study about the efficacy and safety of pyrotinib in patients with HER2 mutant advanced NSCLC. *J. Thor. Oncol.* **2017**, 12, MA0403 abst.
146. Li, B.; Shen, R.; Buonocore, D.; Olah, Z.T.; Ni, A.; Ginsberg, M.; Ulaner, G.; Weber, W.; Ladanyi, M.; Won, H.H.; et al. Ado-trastuzumab emtansine in patients with HER2 mutant lung cancers: results from a phase II basket trial. *J. Clin. Oncol.* **2017**, 35, 8510 abst.
147. Liu, S.; Li, S.; Hai, J.; Wang, X.; Chen, T.; Quinn, M.M.; Gao, P.; Zhang, Y.; Ji, H.; Cross, D.; et al. Targeting HER2 aberrations in non-small cell lung cancer with osimertinib. *Clin. Cancer Res.* **2018**, 24, 1-11.
148. Weiss, J.; Sos, M.L.; Seidel, D.;  [Peifer, M](https://www.ncbi.nlm.nih.gov/pubmed/?term=Peifer%20M%5BAuthor%5D&cauthor=true&cauthor_uid=21160078).; [Zander, T](https://www.ncbi.nlm.nih.gov/pubmed/?term=Zander%20T%5BAuthor%5D&cauthor=true&cauthor_uid=21160078).; [Heuckmann, J.M](https://www.ncbi.nlm.nih.gov/pubmed/?term=Heuckmann%20JM%5BAuthor%5D&cauthor=true&cauthor_uid=21160078).; [Ullrich, R.T](https://www.ncbi.nlm.nih.gov/pubmed/?term=Ullrich%20RT%5BAuthor%5D&cauthor=true&cauthor_uid=21160078).; [Menon, R](https://www.ncbi.nlm.nih.gov/pubmed/?term=Menon%20R%5BAuthor%5D&cauthor=true&cauthor_uid=21160078).; [Maier, S](https://www.ncbi.nlm.nih.gov/pubmed/?term=Maier%20S%5BAuthor%5D&cauthor=true&cauthor_uid=21160078).; [Soltermann, A](https://www.ncbi.nlm.nih.gov/pubmed/?term=Soltermann%20A%5BAuthor%5D&cauthor=true&cauthor_uid=21160078).; et al. Frequent and focal FGFR1 amplification associates with therapeutically tractable FGFR1 dependency in squamous cell lung cancer. *Sci. Transl. Med*. **2010**, 2, 6220293.
149. Drilon, A.; Wang, L.; Arcila, M.E.; [Balasubramanian, S](https://www.ncbi.nlm.nih.gov/pubmed/?term=Balasubramanian%20S%5BAuthor%5D&cauthor=true&cauthor_uid=25567908).; [Greenbowe, J.R](https://www.ncbi.nlm.nih.gov/pubmed/?term=Greenbowe%20JR%5BAuthor%5D&cauthor=true&cauthor_uid=25567908).; [Ross, J.S](https://www.ncbi.nlm.nih.gov/pubmed/?term=Ross%20JS%5BAuthor%5D&cauthor=true&cauthor_uid=25567908).; [Stephens, P](https://www.ncbi.nlm.nih.gov/pubmed/?term=Stephens%20P%5BAuthor%5D&cauthor=true&cauthor_uid=25567908).; [Lipson, D](https://www.ncbi.nlm.nih.gov/pubmed/?term=Lipson%20D%5BAuthor%5D&cauthor=true&cauthor_uid=25567908).; [Miller, V.A](https://www.ncbi.nlm.nih.gov/pubmed/?term=Miller%20VA%5BAuthor%5D&cauthor=true&cauthor_uid=25567908).; [Kris, M.G](https://www.ncbi.nlm.nih.gov/pubmed/?term=Kris%20MG%5BAuthor%5D&cauthor=true&cauthor_uid=25567908).; et al. Broad, hybrid-capture-based next generation sequencing identifies actionable genomic alterations in lung adenocarcinomas otherwise negative for such alterations by other genomic testing approaches. *Clin. Cancer Res*. **2015**, 21, 3631-9.
150. Sousa, V.; Reis, D.; Silva, M.; Alarçao, A.M.; Laideeirinha, A.E.; d’Aguiar, M.J.; Ferreira, T.; Carmujo-Balseiro, S.; Carvalho, L. Amplification of FGFR1 gene and expression of FGFR1 protein is found in different histological types of lung carcinoma. *Virchows Arch.* **2016**, 469, 173-182.
151. Frampton, G.; Ali, S.; Rosenzweig, M.; Chmielcki, J.; Lu, X.; Bauer, T.; Akimov, M.; Bufill, J.; Lee, C.; Jentz, D.; et al. Activation of MET via diverse exon 14 splicing alteration occurs in multiple tumor types and confers clinical sensitivity to MET inhibitors. *Cancer Discov.* **2015**, 5, 850-859.
152. Awad, M.M.; Oxnard, G.R.; Jachman, D.M.; Savukovski, D.O.; Hall, D.; Shivdasani, P.; Heng, J.C.; Dahlberg, S.E.; Janne, P.A.; Verma, S.; et al. MET exon 14 mutations in non-small-cell lung cancer are associated with advanced age and stage-dependent MET genomic amplification and c-Met overexpression. *J. Clin. Oncol.* **2016**, 34, 721-730.
153. Tong, J.H.; Yeung, S.F.; Chan, A.; Chung, L.; Chan, S.; Lung, R.; Tong, C.; Chow, C.; Tin, E.; Yu, Y.; et al. MET amplification and exon 14 splice site mutation define unique molecular subgroups of non-small cell lung carcinoma with poor prognosis. *Clin. Cancer Res.* **2016**, 22, 3048-3056.
154. Zheng, D.; Wang, R.; Ye, T.; Yu, S.; Hu, H.; Shen, X.; Li, Y.; Ji, H.; Sun, Y.; Chen, H. MET exon 14 skipping defines a unique molecular of non-small cell lung cancer. *Oncotarget* **2016**, 7, 41691-41702.
155. Plenker, D.; Bertrand, M.; deLangen, A.; Riedel, R.; Lorenz, C.; Scheel, A.; Muller, J.; Bragelmann, J.; Dabler-Plamker, J.; Kobe, C.; et al. Structural alterations of MET trigger response to MET kinase inhibition in lung adenocarcinoma patients. *Clin. Cancer Res.* **2017**, 24, 1-7.
156. Salgia, R. MET in lung cancer: biomarker selection based on scientific rationale. *Mol. Cancer Ther.* **2017**, 16, 555-565.
157. Li, A.; Yang, J.J.; Zhang, X.C.; Zhang, Z.; Su, J.; Gou, L.Y.; Bai, Y.; Zhou, Q.; Yang, Z.; Han-Zhang, H.; et al. Acquired METY1248H and D1246N mutations mediate resistance to MET inhibitors in non-small cell lung cancer. *Clin. Cancer Res.* **2017**, 23, 4929-4937.
158. Schildhaus, H.U.; Schultheis, A.M.; Rucshoff, J.;  [Binot, E](https://www.ncbi.nlm.nih.gov/pubmed/?term=Binot%20E%5BAuthor%5D&cauthor=true&cauthor_uid=25492085).; [Merkelbach-Bruse, S](https://www.ncbi.nlm.nih.gov/pubmed/?term=Merkelbach-Bruse%20S%5BAuthor%5D&cauthor=true&cauthor_uid=25492085).; [Fassunke, J](https://www.ncbi.nlm.nih.gov/pubmed/?term=Fassunke%20J%5BAuthor%5D&cauthor=true&cauthor_uid=25492085).; [Schulte, W](https://www.ncbi.nlm.nih.gov/pubmed/?term=Schulte%20W%5BAuthor%5D&cauthor=true&cauthor_uid=25492085).; [Ko, Y.D](https://www.ncbi.nlm.nih.gov/pubmed/?term=Ko%20YD%5BAuthor%5D&cauthor=true&cauthor_uid=25492085).; [Schlesinger, A](https://www.ncbi.nlm.nih.gov/pubmed/?term=Schlesinger%20A%5BAuthor%5D&cauthor=true&cauthor_uid=25492085).; [Bos, M](https://www.ncbi.nlm.nih.gov/pubmed/?term=Bos%20M%5BAuthor%5D&cauthor=true&cauthor_uid=25492085).; et al. MET amplification status in therapy-naïve adeno- and squamous cell carcinomas of the lung. *Clin. Cancer Res*. **2015**, 21,907-915.
159. Gou, L.Y.; Li, A.N.; Yang, J.J.; Zhang, X.C.; Su, J.; Yan, H.H.; Xie, Z.; Lou, N.N.; Liu, S.Y.; Dong, Z.Y.; et al. The coexistence of MET over-expression and an EGFR T790M mutation is related to acquired resistance to EGFR tyrosine kinase inhibitors in advanced non-small cell lung cancer. *Oncotarget* **2016**, 7, 51311-51319.
160. Suryavanshi, M.; Shah, A.; Kumar, D.; Panigrahi, M.K.; Metha, A.; Batra, U. MET amplification and response to MET inhibitors in stage IV lung adenocarcinoma. *Oncol. Res. Treat.* **2017**, 40, 198-202.
161. Cappuzzo, F.; Marchetti, A.; Skokan, M.; Rossi, E.; Gajapathy, S.; Felicioni, L.; Del Grammastro, M.; Sciarrotta, M.G.; Buttitta, F.; Incarbone, M.; et al. Increased MET gene copy number negatively affects survival of surgically resected non-small-cell lung cancer patients. *J. Clin. Oncol.* **2009**, 27, 1667-1674.
162. Gainor, J.F.; Niederst, M.J.; Lennerz, J.K.; Dagogo-Jack, I.; Stevens, S.; Shaw, A.T.; Sequist, L.V.; Engelman, J.A. Dramatic response to combination erlotinib and crizotinib in a patient with advanced, EGFR-mutant lung cancer harboring de novo Met amplification. *J. Thorac. Oncol.* **2016**, 11, e83-85.
163. Bahcall, M.; Sim, T.; Paweletz, C.P.; Patel, J.D.; Alden, R.S.; Kuang, Y.; Sacher, A.G.; Kim, N.D.; Lydon, C.; Awad, M.M.; et al. Acquired MET D1228V mutation and resistance to MET inhibition in lung cancer. *Cancer* *Discov.* **2016**, 6, 1335-1341.
164. Baldacci, S.; Mazieres, J.<; Tomasini, P.; Girard, N.; Guisier, F.; Audigier-Valette, C.; Monnet, I.; Wislez, M.; Perol, M.; Do, P.; et al. Outcome of EGFR-mutated NSCLC patients with MET-driven resistance to EGFR tyrosine kinase inhibitors. *Oncotarget* **2017**, 8, 105103-105114.
165. Schuler, M.H.; Berardi, R.; Lim, W.; Gell, R.V.; DeJong, M.J.; Bauer, T.M. Phase I study of the safety and efficacy of the cMET inhibitor capmatinib (INC280) in patients with advanced cMET+ non-small cell lung cancer (NSCLC). *J. Clin. Oncol.* **2016**, 34, 9067.
166. Wu, Y.; Kim, D.; Felip, E.; Zhong, L.; Liu, X.; Zhou, C.C. Phase II safety and efficacy of a single arm ph Ib/II study of capmatinib (INC280) + gefitinib in patients (pts) with EGFR-mutated (mut), cMET-positive (cMET+) non-small cell lung cancer (NSCLC). *J. Clin. Oncol.***2016**, 34, 9020.
167. Camidge, D.R.; Ou, S.I.; Shapiro, G.; Otterson, G.A.; Villaruz, L.C.; Villanova-Calero, M.A. Efficacy and safety of crizotinib in patients with advanced c-MET-amplified non-small cell lung cancer. *J. Clin. Oncol.* **2014**, 32, 8001.
168. Kim, J.H.; Kim, H.S.; Kim, B.J. MET inhibitors in advanced non-small-cell lung cancer: a meta-analysis and review. *Oncotarget* **2017**, 8, 75500-75508.
169. Albitar, M.; Sudarsanam, S.; Ma, W.; Jiang, S.; Chen, W.; Funari, V.; Blocker, F.; Agersborg, S. Correlation of MET gene amplification and TP53 mutation with PD-L1 expression in non-small cell lung cancer. *Oncotarget* **2018**, 9, 13682-13693.
170. Piotrowska, Z. MET amplification (amp) as a resistance mechanism to osimertinib. *J. Clin. Oncol.* **2017**, 35, suppl.; abstr 9020.
171. Wang, Y.; Li, L.; Jiao, L.; Zheng, J.; He, Y. Clinical analysis by next-generation sequencing for NSCLC patients with MET amplification resistant to osimertinib. *Lung Cancer* **2018**, 118, 105-110.
172. Ninomiya, K.; Ohashi, K.; Makimoto, G.; Tomida, S.; Higo, H.; Kayatani, H.; Ninomiya, T.; Kub o, T.; Ichihara, E.; Hotta, K.; et al. MET or NRAs amplification is an acquired resistance mechanism to the third-generation EGFR inhibitor naquotinib. *Scient. Rep.* **2018**, 8, 1955.
173. Sanchez-Cespedes, M.; Parrella, P.; Esteller, M.; [Nomoto, S](https://www.ncbi.nlm.nih.gov/pubmed/?term=Nomoto%20S%5BAuthor%5D&cauthor=true&cauthor_uid=12097271).; [Trink, B](https://www.ncbi.nlm.nih.gov/pubmed/?term=Trink%20B%5BAuthor%5D&cauthor=true&cauthor_uid=12097271).; [Engles, J.M](https://www.ncbi.nlm.nih.gov/pubmed/?term=Engles%20JM%5BAuthor%5D&cauthor=true&cauthor_uid=12097271).; [Westra, W.H](https://www.ncbi.nlm.nih.gov/pubmed/?term=Westra%20WH%5BAuthor%5D&cauthor=true&cauthor_uid=12097271).; [Herman, J.G](https://www.ncbi.nlm.nih.gov/pubmed/?term=Herman%20JG%5BAuthor%5D&cauthor=true&cauthor_uid=12097271).; [Sidransky, D](https://www.ncbi.nlm.nih.gov/pubmed/?term=Sidransky%20D%5BAuthor%5D&cauthor=true&cauthor_uid=12097271). Inactivation of LKB1/STK11 is a common event in adenocarcinomas of the lung. *Cancer Res*. **2002**, 62, 3659-3662.
174. Gill, R.K.; Yang, S.H.; Mezzaman, D.; [Mechanic, L.E](https://www.ncbi.nlm.nih.gov/pubmed/?term=Mechanic%20LE%5BAuthor%5D&cauthor=true&cauthor_uid=21532627).; [Bowman, E.D](https://www.ncbi.nlm.nih.gov/pubmed/?term=Bowman%20ED%5BAuthor%5D&cauthor=true&cauthor_uid=21532627).; [Jeon, H.S](https://www.ncbi.nlm.nih.gov/pubmed/?term=Jeon%20HS%5BAuthor%5D&cauthor=true&cauthor_uid=21532627).; [Roy Chowdhuri, S](https://www.ncbi.nlm.nih.gov/pubmed/?term=Roy%20Chowdhuri%20S%5BAuthor%5D&cauthor=true&cauthor_uid=21532627).; [Shakoori, A](https://www.ncbi.nlm.nih.gov/pubmed/?term=Shakoori%20A%5BAuthor%5D&cauthor=true&cauthor_uid=21532627).; [Dracheva, T](https://www.ncbi.nlm.nih.gov/pubmed/?term=Dracheva%20T%5BAuthor%5D&cauthor=true&cauthor_uid=21532627).; [Hong, K.M](https://www.ncbi.nlm.nih.gov/pubmed/?term=Hong%20KM%5BAuthor%5D&cauthor=true&cauthor_uid=21532627).; et al. Frequent homozygous deletion of the LKB1/STK11 gene in non-small cell lung cancer. *Oncogene* **2011**, 30, 3784-3791.
175. Ji, H.; Ramsey, E.R.; Haynes, D.N.;  [Fan, C](https://www.ncbi.nlm.nih.gov/pubmed/?term=Fan%20C%5BAuthor%5D&cauthor=true&cauthor_uid=17676035).; [McNamara, K](https://www.ncbi.nlm.nih.gov/pubmed/?term=McNamara%20K%5BAuthor%5D&cauthor=true&cauthor_uid=17676035).; [Kozlowski, P](https://www.ncbi.nlm.nih.gov/pubmed/?term=Kozlowski%20P%5BAuthor%5D&cauthor=true&cauthor_uid=17676035).; [Torrice, C](https://www.ncbi.nlm.nih.gov/pubmed/?term=Torrice%20C%5BAuthor%5D&cauthor=true&cauthor_uid=17676035).; [Wu, M.C](https://www.ncbi.nlm.nih.gov/pubmed/?term=Wu%20MC%5BAuthor%5D&cauthor=true&cauthor_uid=17676035).; [Shimamura, T](https://www.ncbi.nlm.nih.gov/pubmed/?term=Shimamura%20T%5BAuthor%5D&cauthor=true&cauthor_uid=17676035).; [Perera, S.A](https://www.ncbi.nlm.nih.gov/pubmed/?term=Perera%20SA%5BAuthor%5D&cauthor=true&cauthor_uid=17676035).; et al. LKB1 modulates lung cancer differentiation and metastasis. *Nature* **2007**, 448, 807-810.
176. Feng, Y.; Wang, Y.; Wang, Z.; [Fang, Z](https://www.ncbi.nlm.nih.gov/pubmed/?term=Fang%20Z%5BAuthor%5D&cauthor=true&cauthor_uid=23074285).; [Li, F](https://www.ncbi.nlm.nih.gov/pubmed/?term=Li%20F%5BAuthor%5D&cauthor=true&cauthor_uid=23074285).; [Gao, Y](https://www.ncbi.nlm.nih.gov/pubmed/?term=Gao%20Y%5BAuthor%5D&cauthor=true&cauthor_uid=23074285).; [Liu, H](https://www.ncbi.nlm.nih.gov/pubmed/?term=Liu%20H%5BAuthor%5D&cauthor=true&cauthor_uid=23074285).; [Xiao, T](https://www.ncbi.nlm.nih.gov/pubmed/?term=Xiao%20T%5BAuthor%5D&cauthor=true&cauthor_uid=23074285).; [Li, F](https://www.ncbi.nlm.nih.gov/pubmed/?term=Li%20F%5BAuthor%5D&cauthor=true&cauthor_uid=23074285).; [Zhou, Y](https://www.ncbi.nlm.nih.gov/pubmed/?term=Zhou%20Y%5BAuthor%5D&cauthor=true&cauthor_uid=23074285).; et al. The CRCT1-NEDD9 signaling axis mediates lung cancer progression caused by LKB1 loss. *Cancer Res*. **2012**, 72, 6502-6511.
177. Shackelford, D.B.; Abt, E.; Gerken, L.;  [Vasquez, D,S](https://www.ncbi.nlm.nih.gov/pubmed/?term=Vasquez%20DS%5BAuthor%5D&cauthor=true&cauthor_uid=23352126).; [Seki, A](https://www.ncbi.nlm.nih.gov/pubmed/?term=Seki%20A%5BAuthor%5D&cauthor=true&cauthor_uid=23352126).; [Leblanc, M](https://www.ncbi.nlm.nih.gov/pubmed/?term=Leblanc%20M%5BAuthor%5D&cauthor=true&cauthor_uid=23352126).; [Wei, L](https://www.ncbi.nlm.nih.gov/pubmed/?term=Wei%20L%5BAuthor%5D&cauthor=true&cauthor_uid=23352126).; [Fishbein, M.C](https://www.ncbi.nlm.nih.gov/pubmed/?term=Fishbein%20MC%5BAuthor%5D&cauthor=true&cauthor_uid=23352126).; [Czernin, J](https://www.ncbi.nlm.nih.gov/pubmed/?term=Czernin%20J%5BAuthor%5D&cauthor=true&cauthor_uid=23352126).; [Mischel, P.S](https://www.ncbi.nlm.nih.gov/pubmed/?term=Mischel%20PS%5BAuthor%5D&cauthor=true&cauthor_uid=23352126).; et al. LKB1 inactivation dictates therapeutic response of non-small cell lung cancer to the metabolism drug phenformin. *Cancer Cell* **2013**, 23, 143-158.
178. Jin, L.; Chum, J.; Kumar, A.; Zhang, G.; Ha, Y.; Li, D.; Alesi, G.N.; Kang, Y.; Zhou, L.; Yu, Y.M.; et al. The PLAG1-GDH1 axis promotes anoikis, resistance and tumor metastasis through CamKK2-AMPK signaling in LKB1-deficient lung cancer. *Mol. Cell* **2018**, 69, 87-99.
179. Skoulidis, F.; Byers, L.; Diao, L.; Papadimitrakopoulos, V.; Tong, P.; Izzo, J.; Behrens, C.; Codora, H.; Parra, E.R.; Rodriguez Canales, J.; et al. Co-occurring genomic alterations define major subsets of KRAS-mutant lung adenocarcinoma wi distinct biology, immune profiles, and therapeutic vulnerabilities. *Cancer Discover.* **2015**, 5, 860-877.
180. Skoulidis, F.; Hellmann, M.D.; Awad, M.M.; Rizvi, H.; Carter, B.W.; Denning, W. STK11/LKB1 co-mutations to predict for de novo resistance to PD-1/PD-L1 axis blockade in KRAS-mutant lung adenocarcinoma. *J. Clin. Oncol.* **2017**, 35, abst. 9016.
181. Gilbert-Ross, M.; Konen, J.; Koo, J.; Shupe, J.; Robinson, B.S.; Wiles IV, W.; Huang, C.; Martin, W.D.; Behera, M.; Smith, G.H.; et al. Targeting adhesion signaling in KRAS, LKB1 mutant lung adenocarcinoma. *JCI Insight* **2017**, 2, e90487.
182. Zhang, H.; Brainson, C.F.; koyama, S.; Redig, A.; Chen, T.; Li, S.; Gupta, M.; Garcia-de-Alba, C.; Paschini, M.; Herter-Sprie, G.S.; et al. Lkb1 inactivation drives lung cancer lineage switching governed by Polycomb repressive complex. *Nat. Commun.* **2017**, 8, 14922.
183. Liu, Y.; Li, Y.; Wang, X.; Liu, F.; Gao, P.; Quinn, M.M.; li, F.; Merlino, A.A.; Benes, C.; Liu, Q.; et al. Gemcitabine and Chk1 inhibitor AZD7762 synergistically suppress the growth of LKB1-deficient lung adenocarcinoma. *Cancer Res.* **2017**, 77, 5068-5076.
184. Levy, M.A.; Lovly, C.M.; Pao, W. Translating genomic information into clinical medicine: lung cancer as a paradigm. *Genome Res.* **2012**, 22, 2101-8.
185. Arbour, K.C.; Jordan, E.; Kim, H.R.; Dienstag, J.; Yu, H.A.; Sanchez-Vega, F.; Lito, P.; Berger, M.; Solit, D.B.; Hellmann, M.; et al. Effects of co-occurring genomic alterations on outcomes in patients with KRAS-mutant non-small cell lung cancer. *Clin. Cancer Res.* **2017**, 24, 334-340.
186. Berger, A.H.; Brooks, A.N.; Wu, X.; Shrestha, Y.; Chovinard, C.; Piccioni, F.; Bagul, M.; Kamburov, A.; Imielinski, M.; Hoystrom, L.; et al. High-throughput phenotyping of lung cancer somatic mutations. *Cancer* *Cell* **2016**, 30, 214-228.
187. Solis, L.M.; Behrens, C.; Dong, W.; Suraokar, M.; Ozburn, N.C.; Moran, C.A.; Corvalan, A.; Biswal, S.; Swisher, S.G.; Bekele, B.N.; et al. Nrf2 and Keap1 abnormalities in non-small cell lung carcinoma and association with clinicopathologic features. *Clin. Cancer Res.* **2010**, 16, 3743-3753.
188. Namani, A.; Cui, Q.Q.; Wu, Y.; Wang, H.; Wang, X.J.; Tang, X. NRF2-regulated metabolic gene signature as a prognostic biomarker in non-small cell lung cancer. *Oncotarget* **2017**, 8, 69847-69862.
189. Frank, R.; Scheffler, M.; Merkelbach-Bruse, S.; Ihle, M., Kron, A.; Rauer, M.; Ueckertoh, F.; Konig, K.; Michels, S.; Fischer, R.; et al. Clinical and pathological charcateristics of KEAP1- and NFE2L2-mutated non-small cell lung adenocarcinoma (NSCLC). *Clin. Cancer Res.* **2018**, in press.
190. Krall, E.B.; Wang, B.; Munoz, D.M.; Ilic, N.; Raghwan, S.; Niederst, M.; Yu, K.; Ruddy, D.A.; Aguirre, A.J.; Kim, J.W.; et al. KEAP1 loss modulates sensitivity to kinase targeted therapy in lung cancer. *eLIFE* **2017**, 6, e18970.
191. Romero, S.P.; Sayin, V.I.; Davidson, S.M.; Bauer, M.R.; Singh, S.X.; LeBoeuf, S.E.; Karakousi, T.R.; Ellis, D.C.; Bhutkar, A.; Sanchez-Rivera, F.J.; et al. Keap1 promotes KRas-driven lung cancer and results in dependence bon glutaminolysis. *Nat. Med.* **2017**, 23, 1362-1368.
192. Beast, S.A.; De Souza, D.P.; Kersbergen, A.; Policheni, A.N.; Dayalan, S.; Tull, D.; Rathi, V.; Gray, D.H.; Ritchie, M.E.; McConville, M.J.; Sutherland, K.D. Synergy between the KEAP1/NRF2 and PI3K pathways drives non-small-cell lung cancer with an altered immune microenvironment. *Cell Met.* **2018**, 27, 935-943.
193. Bar-Peled, L.; Kempler, E.K.; Suciu, R.M.; Vinogradova, E.; Backus, K.M.; Horning, B.; Paul, T.A.; Ichu, T.A.; Svensson, R.U.; Olucha, J.; et al. Chemical proteomics identifies druggable vulnerabities in a genetically defined cancer. *Cell* **2017**, 171, 696-709.
194. Choi, E.J.; Jung, B.J.; Lee, S.H.; Yoo, H.S.; Shin, E.A.; Ko, H.J.; Chang, S.; Kim, S.Y.; Jean, S.M.A clinical drug library screen identifies clobetasol propionate as an NRF2 inhibitor with potential therapeutic efficacy in KEAP1 mutant lung cancer. *Oncogene* **2017**, 36, 5285-5295.
195. McMillan, E.A.; Ryu, M.J.; Diep, C.H.; Mendiratta, S.; Clemenceau, J.R.; Vaden, R.M.; Kim, J.H.; Motoyaji, T.; Covington, K.R.; Peyton, M.; et al. Chemistry-first approach for nomination of personalized treatment in lung cancer. *Cell* **2018**, 173, 864-878.
196. Starczynowski, D.T.; Lockwood, W.W.; Deléhouzée, S.;  [Chari, R](https://www.ncbi.nlm.nih.gov/pubmed/?term=Chari%20R%5BAuthor%5D&cauthor=true&cauthor_uid=21911935).; [Wegrzyn, J](https://www.ncbi.nlm.nih.gov/pubmed/?term=Wegrzyn%20J%5BAuthor%5D&cauthor=true&cauthor_uid=21911935).; [Fuller, M](https://www.ncbi.nlm.nih.gov/pubmed/?term=Fuller%20M%5BAuthor%5D&cauthor=true&cauthor_uid=21911935).; [Tsao, M.S](https://www.ncbi.nlm.nih.gov/pubmed/?term=Tsao%20MS%5BAuthor%5D&cauthor=true&cauthor_uid=21911935).; [Lam, S](https://www.ncbi.nlm.nih.gov/pubmed/?term=Lam%20S%5BAuthor%5D&cauthor=true&cauthor_uid=21911935).; [Gazdar, A.F](https://www.ncbi.nlm.nih.gov/pubmed/?term=Gazdar%20AF%5BAuthor%5D&cauthor=true&cauthor_uid=21911935).; [Lam, W.L](https://www.ncbi.nlm.nih.gov/pubmed/?term=Lam%20WL%5BAuthor%5D&cauthor=true&cauthor_uid=21911935).; et al. TRAF6 is an amplified oncogene bridging the RAS and NF-kB pathways in human lung cancer. *J. Clin. Invest*. **2011**, 121, 4095-4105.
